# Extreme genomic erosion after recurrent demographic bottlenecks in the highly endangered Iberian lynx

**DOI:** 10.1186/s13059-016-1090-1

**Published:** 2016-12-14

**Authors:** Federico Abascal, André Corvelo, Fernando Cruz, José L. Villanueva-Cañas, Anna Vlasova, Marina Marcet-Houben, Begoña Martínez-Cruz, Jade Yu Cheng, Pablo Prieto, Víctor Quesada, Javier Quilez, Gang Li, Francisca García, Miriam Rubio-Camarillo, Leonor Frias, Paolo Ribeca, Salvador Capella-Gutiérrez, José M. Rodríguez, Francisco Câmara, Ernesto Lowy, Luca Cozzuto, Ionas Erb, Michael L. Tress, Jose L. Rodriguez-Ales, Jorge Ruiz-Orera, Ferran Reverter, Mireia Casas-Marce, Laura Soriano, Javier R. Arango, Sophia Derdak, Beatriz Galán, Julie Blanc, Marta Gut, Belen Lorente-Galdos, Marta Andrés-Nieto, Carlos López-Otín, Alfonso Valencia, Ivo Gut, José L. García, Roderic Guigó, William J. Murphy, Aurora Ruiz-Herrera, Tomas Marques-Bonet, Guglielmo Roma, Cedric Notredame, Thomas Mailund, M. Mar Albà, Toni Gabaldón, Tyler Alioto, José A. Godoy

**Affiliations:** 1 Structural Biology and Biocomputing Programme, Spanish National Cancer Research Centre (CNIO), Madrid, 28029 Spain; 2 CNAG-CRG, Centre for Genomic Regulation (CRG), Barcelona Institute of Science and Technology (BIST), Baldiri i Reixac 4, 08028 Barcelona, Spain; 3 Department of Integrative Ecology, Doñana Biological Station (EBD), Spanish National Research Council (CSIC), C/ Americo Vespucio, s/n, 41092 Sevilla, Spain; 4 Evolutionary Genomics Group, Research Programme on Biomedical Informatics (GRIB), Hospital del Mar Research Institute (IMIM), Dr. Aiguader 88, 08003 Barcelona, Spain; 5 Bioinformatics and Genomics Programme, Centre for Genomic Regulation (CRG), Dr. Aiguader 88, 08003 Barcelona, Spain; 6 Universitat Pompeu Fabra (UPF), Dr. Aiguader 88, 08003 Barcelona, Spain; 7 Bioinformatics Research Centre, Aarhus University, C.F. Møllers Allé 8, 8000 Aarhus, Denmark; 8 Departamento de Bioquímica y Biología Molecular, Instituto Universitario de Oncología (IUOPA), Universidad de Oviedo, 33006 Oviedo, Spain; 9 Institut de Biologia Evolutiva (UPF-CSIC), Universitat Pompeu Fabra, PRBB, Doctor Aiguader, 88, 08003 Barcelona, Spain; 10 Department of Veterinary Integrative Biosciences, College of Veterinary Medicine, Texas A&M University, College Station, TX 77843 USA; 11 Servei de Cultius Cel.lulars (SCC, SCAC), Universitat Autònoma de Barcelona, Barcelona, Spain; 12 National Bioinformatics Institute (INB), Spanish National Cancer Research Centre (CNIO), Madrid, 28029 Spain; 13 Bioinformatics Core Facility, Centre for Genomic Regulation (CRG), Dr. Aiguader 88, 08003 Barcelona, Spain; 14 Department of Environmental Biology, Center for Biological Research (CIB), Spanish National Research Council (CSIC), Ramiro de Maeztu 9, 28040 Madrid, Spain; 15 Institut de Biotecnologia i de Biomedicina, Universitat Autònoma de Barcelona, 08193 Cerdanyola del Vallès, Spain; 16 Computational Genomics Group, Research Programme on Biomedical Informatics (GRIB), Hospital del Mar Research Institute (IMIM), Dr. Aiguader 88, 08003 Barcelona, Spain; 17 Departament de Biologia Cel.lular, Fisiologia i Immunologia, Universitat Autònoma de Barcelona, 08193 Cerdanyola del Vallès, Spain; 18 Institució Catalana de Recerca i Estudis Avançats (ICREA), Pg. Lluís Companys 23, 08010 Barcelona, Spain

**Keywords:** Conservation genomics, Genetic diversity, Inbreeding, Genetic drift, Lynx

## Abstract

**Background:**

Genomic studies of endangered species provide insights into their evolution and demographic history, reveal patterns of genomic erosion that might limit their viability, and offer tools for their effective conservation. The Iberian lynx (*Lynx pardinus*) is the most endangered felid and a unique example of a species on the brink of extinction.

**Results:**

We generate the first annotated draft of the Iberian lynx genome and carry out genome-based analyses of lynx demography, evolution, and population genetics. We identify a series of severe population bottlenecks in the history of the Iberian lynx that predate its known demographic decline during the 20th century and have greatly impacted its genome evolution. We observe drastically reduced rates of weak-to-strong substitutions associated with GC-biased gene conversion and increased rates of fixation of transposable elements. We also find multiple signatures of genetic erosion in the two remnant Iberian lynx populations, including a high frequency of potentially deleterious variants and substitutions, as well as the lowest genome-wide genetic diversity reported so far in any species.

**Conclusions:**

The genomic features observed in the Iberian lynx genome may hamper short- and long-term viability through reduced fitness and adaptive potential. The knowledge and resources developed in this study will boost the research on felid evolution and conservation genomics and will benefit the ongoing conservation and management of this emblematic species.

**Electronic supplementary material:**

The online version of this article (doi:10.1186/s13059-016-1090-1) contains supplementary material, which is available to authorized users.

## Background

Species are becoming extinct at rates unprecedented in recent history as a consequence of human activity [1]. Surviving populations of vertebrate species have decreased in size by an average of 58% from 1970 to 2012 [2] and 15–40% of living species are predicted to go extinct by 2050 [3]. While the primary causes of these declines are usually known and have been the main targets of conservation efforts, genetic changes accumulated during the decline can compromise the recovery of endangered populations and limit their long-term viability. Indeed, endangered populations typically show patterns of low genetic diversity and high inbreeding that can result in loss of adaptive potential, reduced rates of reproduction and survival, and increased extinction risk [4]. Genomic approaches are expected to improve our understanding of how the interaction between genetic drift, mutation, recombination, and natural selection shapes the genome of endangered populations and to contribute to a more effective conservation by facilitating the identification and subsequent management of deleterious variants. The fulfillment of these expectations requires, however, genomic studies in well-characterized and actively managed endangered species to serve as models [5].

The Iberian lynx (*Lynx pardinus*) is one of the four extant lynx species that share a short bobbed tail, spotted coat, muscular body, long legs, and characteristic tufted ears and beard-resembling ruffs. The Iberian and Eurasian lynx are sister species and the two extant lynxes in Eurasia, having diverged around 1.1 million years ago (Mya) [6, 7]. In contrast to the large, generalist and widespread Eurasian lynx, the Iberian lynx is smaller and a habitat- and prey-specialist, being restricted to the Mediterranean region in the Iberian Peninsula where they prey almost exclusively on rabbits. Supposed to be once fairly abundant and widely distributed across the Iberian peninsula, a steep decline during the second half of the 20th century left less than 100 lynx (less than 62 mature) distributed in the two isolated populations of Doñana and Andújar (Sierra Morena) in Andalusia, southern Spain, leading to its recognition as the most endangered felid in the world [8] and to its classification as “critically endangered” in the 2002, 2006, and 2008 IUCN red lists. Active conservation in the last 14 years, including in situ management of habitat, prey, and non-natural mortality, captive breeding, translocation, and reintroduction programs, has recovered lynx numbers to over 300 (156 mature) individuals in 2012, leading to its downlisting to “endangered” in the 2015 IUCN red list [9].

Previous studies using microsatellite markers documented low genetic diversity, a high inbreeding rate, and a high genetic differentiation between the two populations [10]. The following evidence suggests that these genetic factors are limiting current reproduction and survival rates (inbreeding depression): (i) an increase in the proportion of abnormal sperm with individual inbreeding [11]; (ii) a recent decrease in litter size and survival in Doñana [12]; (iii) a high incidence of membranous glomerulonephritis and lymphoid depletion [13, 14]; and (iv) several deleterious traits with likely genetic bases segregate at moderate to high frequencies in the captive population, including cryptorchidism and an idiopathic epilepsy [15]. This has prompted the translocation of individuals to reconnect the two remnant populations and their mixing in captivity, which has likely contributed to improved reproductive and survival rates. These circumstances make of the Iberian lynx a good model for the emerging field of conservation genomics [16].

We have sequenced, assembled, and annotated a draft genome of an Iberian male named *Candiles*, and re-sequenced another ten Iberian and one Eurasian lynx genomes. In addition, to obtain gene expression data and to assist gene annotation we have characterized the transcriptome of 11 lynx tissues. We use these resources to analyze the marks left by recurrent demographic bottlenecks on the dynamics of transposable elements (TEs), the rates and patterns of nucleotide substitution, and the efficiency of purifying selection. We characterize the genetic diversity in the two remnant Iberian lynx populations and discuss the interplay between demographic history, GC-biased gene conversion, genetic drift, recombination, and selection in a species on the brink of extinction.

## Results and discussion

### The Iberian lynx reference genome

We assembled the first draft of the Iberian lynx genome (LYPA 1.0) by combining a fosmid-pool sequencing approach [17] with shotgun sequencing of whole-genome fragment libraries on Illumina and 454 platforms (Additional file 1: Sections 1 and 2). With a contig N50 of 68 kb, our Iberian lynx assembly is more contiguous than other felid genome assemblies, including those of the domestic cat, tiger, and cheetah (Additional file 1: Table S4). However, due to the limited amount of long-range linkage information we were able to produce, we could not achieve as high a scaffold N50 (1.52 Mb). Regardless, the completeness of the gene space as assessed by the Core Eukaryotic Genes Mapping Approach (CEGMA) [18] was 95% (98% including partial genes). We annotated the reference genome with protein-coding genes and other structural and functional genomic features, including TEs and small non-coding RNAs (sncRNAs) and long non-coding RNAs (lncRNAs) (Additional file 1: Section 3). More than 98% of the 21,257 protein coding genes identified were functionally annotated with InterPro, KEGG, or Blast2GO features (Additional file 1: Section 4). We also performed a focused annotation of the degradome, the complete repertoire of proteases in the organism, with computer-assisted manual methods (Additional file 1: Section 5; Additional file 2: Datasheet S1) and analyzed expression patterns of protein-coding genes (Additional file 1: Section 6) and lncRNAs (Additional file 1: Section 7). A small percentage of these genes (<1%) were expressed in at least one tissue in the Iberian lynx but did not show homology to genes in other mammals or vertebrates (Additional file 1: Section 8; Additional file 2: Datasheet S2). These features, along with other layers of genomic information that include synteny, gene expression, and genomic variation, can be accessed interactively through a dedicated genome browser (http://denovo.cnag.cat/genomes/iberian_lynx; Additional file 1: Section 25).

### Evolutionary and demographic history

**Fig. 1:**
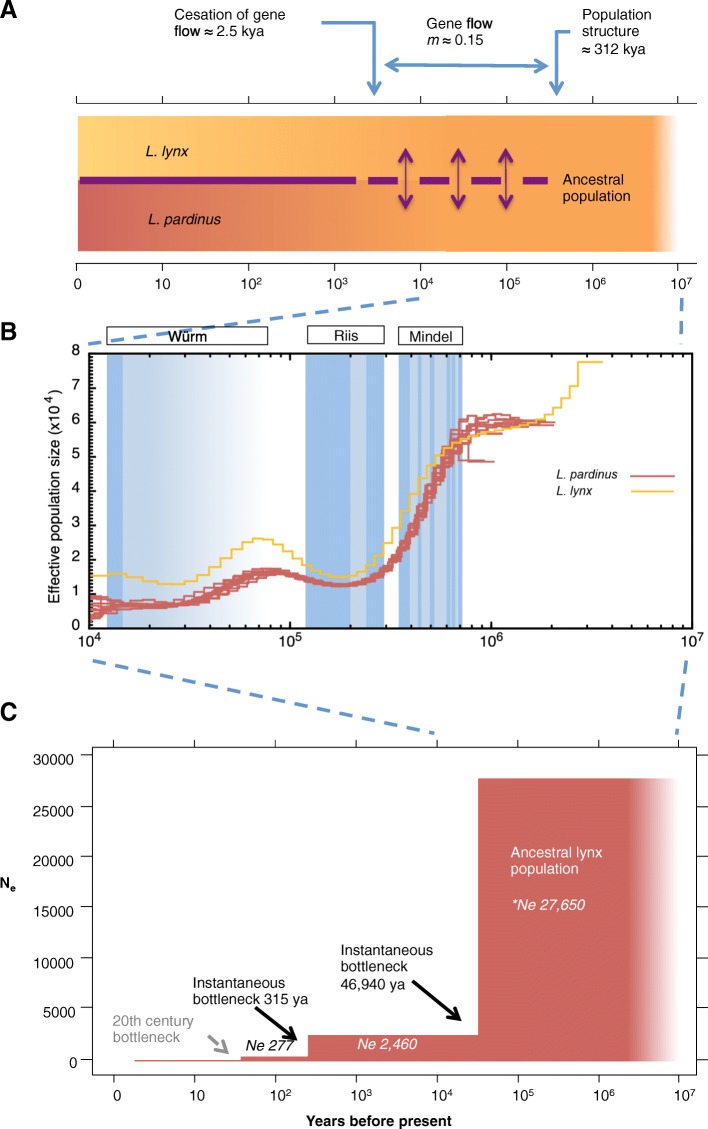
Evolutionary and demographic history of Iberian and Eurasian lynx. **a** Eurasian and Iberian lynx divergence adjusted to a model of divergence with gene flow. Results suggest a relatively recent divergence of the two lynx species followed by a period of gene flow that lasted until recently (circa 2473 years ago). **b** Effective population size through time estimated for each of the 11 Iberian lynx and a single Eurasian individual using PSMC. Demographic histories are similar for the ten Iberian lynx and slightly different for the Eurasian lynx, although both species follow largely parallel fluctuations of population size probably related to glacial cycles; glacial periods are shaded in blue with glacial maxima in darker tone. **c** Based on the allele frequency spectrum, ∂a∂*i* infers a model with two successive abrupt bottlenecks, one around 47 kya, coincident with the last important decline observed in PSMC output, and a second one at 315 years ago, both reducing to approximately one-tenth the previous population size. The most recent documented bottleneck occurring during the 20th century is not recovered by these methods

Phylogenomic analyses confirmed the evolutionary relationships among mammals and felids inferred with smaller datasets [6, 7] and estimated the Eurasian and Iberian lynx divergence as 1.5 Mya (95% credibility interval = 0.9–2.2 Mya; Additional file 1: Section 13). We further investigated the divergence of Iberian and Eurasian lynx using CoalHMM [19, 20] (“Methods”; Additional file 1: Section 9) and their demographic history using PSMC [21] and *∂a∂i* [22] (“Methods”; Additional file 1: Section 10). The former analysis yielded higher support for a divergence model with a period of limited gene flow before complete genetic isolation [20] than for an instantaneous speciation model (isolation with no further gene flow [19] (∆AIC = 4 × 10^3^; Fig. 1a). We found that the demographic history of the Iberian lynx is punctuated by four bottlenecks (Fig. 1b, c; Additional file 1: Section 10). PSMC inferred a first and most drastic population decline that affected both Iberian and Eurasian lynx 700–100 thousand years ago (kya; Fig. 1b); this contraction may have separated the two species, resulting in the onset of the population structure detected by CoalHMM (312.2 kya; 95% confidence interval 323.1–179.4 kya). Subsequently, the demography of both lynx species followed parallel histories with fluctuations apparently associated with glacial cycles and with Eurasian lynx showing slightly larger population sizes than Iberian. A transient increase in effective population sizes during a period of milder climate (130–60 kya; Riss–Würm interglacial) might have favored a range expansion and the interbreeding of Eurasian and Iberian lynxes, causing the signal of gene flow detected in the divergence analyses (*m* = 0.15 migrants per generation in each direction). The subsequent period of progressive cooling (Würm glaciation) apparently caused a second population contraction that may have re-isolated both lynx species. However, genetic interchange apparently did not cease until recently (2.473 kya; 95% confidence interval 126.8–0 kya; Fig. 1a). Certainly, opportunities for interbreeding did exist in the recent past as the two species co-occurred within the Iberian peninsula in historical times [23] and their ranges probably overlapped during the Holocene in southern France and northern Italy, where they may have hybridized [24]. Post-speciation gene flow is becoming progressively recognized as a frequent phenomenon and it has been fairly common among felids in general and lynxes in particular [6], including current occasional hybridization at sites of range overlap (e.g. *Lynx rufus* and *Lynx canadensis* [25]). Therefore, evidence for post-divergence gene flow is not sufficient by itself to question the species-level taxonomic status of the Iberian lynx; further characterization of the patterns, timing, and outcome of admixture will be needed to assess the level of post-zygotic reproductive isolation between these two lynxes.

The analysis with *∂a∂i* was able to capture a third, more recent decline that reduced the Iberian lynx effective population size to less than 300 individuals around 315 years ago (Fig. 1c). A similarly dated bottleneck was detected previously using microsatellite marker data [10] and has also been suggested for the Eurasian lynx [26]. This period is characterized by human population expansions leading to increased persecution of large carnivores, forest destruction, and expansion of agricultural land across Europe [27]. The fourth and most recent bottleneck occurred during the 20th century. This population contraction, attributed to increased direct prosecution and decreases in main prey caused by two successive viral outbreaks, is probably too recent to be recovered by these methods but is well documented in the literature.

### Genome evolution

The Iberian lynx karyotype is identical to that of other felids in terms of diploid number (2n = 38) and G-banding [28] (Additional file 1: Section 11). A finer-scale analysis based on genome alignments identified five potential intra-chromosomal and ten potential inter-chromosomal rearrangements between domestic cat and lynx and up to 37 inversions. Alignments to the dog genome indicated that 20 of the inversions and six of the inter/intra-chromosomal rearrangements occurred in the Iberian lynx lineage. PCR amplification and Sanger sequencing empirically validated 8 of the 15 putative chromosomal rearrangements (five lynx-specific; Additional file 1: Section 12; Additional file 2: Datasheet S3).

We investigated the evolution of lynx genes through several complementary approaches. First, we reconstructed the molecular phylogeny of every lynx protein-coding gene in the context of 15 other mammalian species to establish orthology relationships and to detect and date duplication events [29] (Additional file 1: Section 13). We observed a significant enrichment in genes related to sensory perception of smell among the genes specifically expanded in the lynx lineage and also among those duplicated in the most recent common ancestor of all felids (Additional file 2: Datasheet S4), which is consistent with the importance of smell perception for these carnivores. Besides, and in contrast to humans, felids harbor multiple functional paralogs of the cysteine protease genes *CTSL* and *CTSL2*, ranging from five to ten, which are involved in extracellular matrix homeostasis [30] and immune regulation [31] (Additional file 1: Section 5; Additional file 2: Datasheet S1). Four of these new *CTSL-*like genes have pseudogenized in lynx but not in tiger or cat (Additional file 2: Datasheet S1). Up to 85 additional lynx genes were conservatively identified as putative pseudogenes in lynx (Additional file 2: Datasheet S5).

Second, we used the branch-site test [32] on a set of 9695 one-to-one orthologs from eight mammalian species (*Panthera tigris*, *Felis catus*, *Lynx lynx*, *L. pardinus*, *Ailuropoda melanoleuca*, *Canis lupus familiaris*, *Homo sapiens*, and *Mus musculus*) to identify genes that may have undergone positive selection in lynx (Additional file 1: Section 14). Following extensive manual inspection of the alignments and using strict criteria to minimize alignment errors, we identified 100 genes likely to have accumulated adaptive substitutions in the lynx lineage (Additional file 2: Datasheet S7). Felids possess outstanding hearing [33] and lynx in particular are attributed an exceptionally acute vision and hearing. We found two genes involved in hearing—*CACNA1D* (LYPA23A015140P1) and *MYO1F* (LYPA23A022113P1)—and two genes related to vision—*OPTC* (LYPA23A008195P1) and *GUCY2F* (LYPA23A015393P1) [34]—among those with signatures of positive selection in the lynx lineage.

**Fig. 2:**
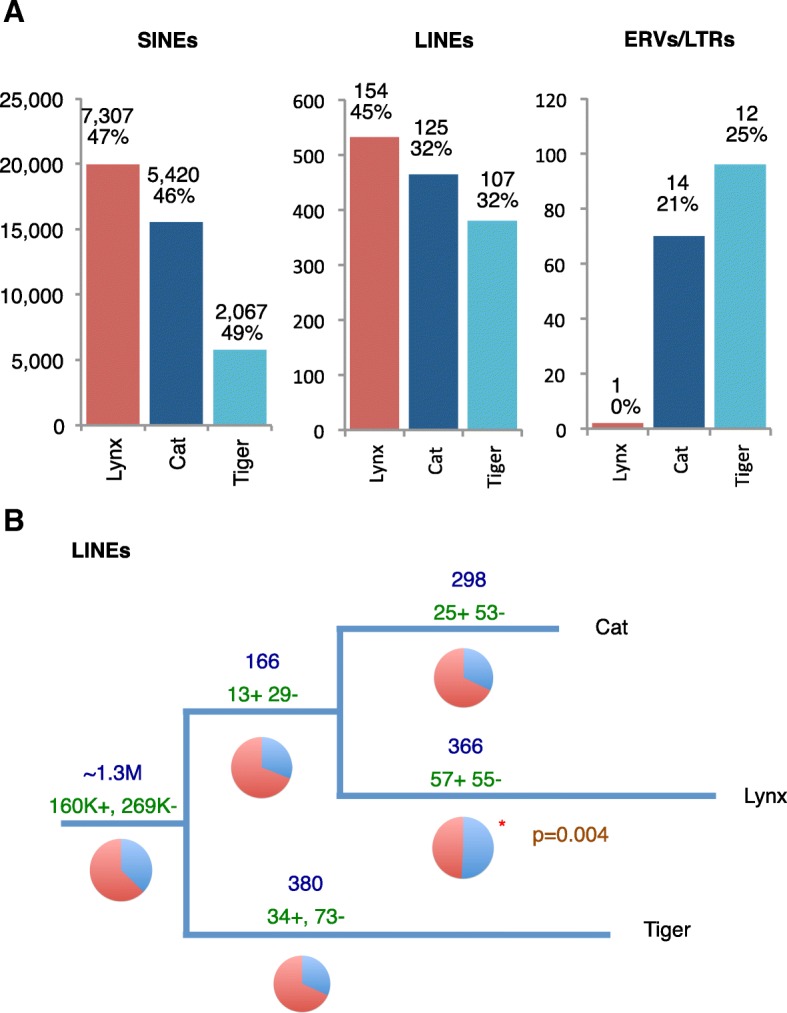
Comparison of transposable element dynamics. **a** Absolute number of species-specific SINE, LINE, and ERV insertions estimated for lynx, cat, and tiger. For SINEs and LINEs, the number of TE insertions was calculated based on pairwise comparisons between domestic cat and lynx and between domestic cat and tiger. The numbers shown represent the accumulation of insertions in each species since the last common ancestor of lynx, cat, and tiger (Additional file 1: Section 15). The number of TE insertions within genes and the percentage of insertions in sense with respect to the gene are shown on top of each bar. *LTR* long terminal repeat. **b** Phylogenetic tree depicts the number of LINE insertions mapped onto each branch (in *blue above branch*) and the number of insertions that are in sense (*+*) or antisense (-) orientation with respect to the genes (in *green below branch*). Relative proportions are shown as *pie charts*, with those significantly departing from the average genome-background frequency of 37% (Lynx) indicated by an asterisk. Branch lengths were set manually to reflect the number of LINE insertions on each branch

Population bottlenecks increase inbreeding, reduce diversity, and make purifying selection less effective. Most models predict an increased fixation rate of TEs and a reduced rate of new TE invasions in bottlenecked and inbred populations, although the net effect might depend on the mechanisms of transposition and the relative importance of negative selection and ectopic recombination [35–37]. Our analysis revealed greater expansions of short interspersed elements (SINEs) and long interspersed elements (LINEs) in lynx than in cat and tiger; on the other hand, and in contrast with cat and tiger, we found no clear evidence of recent invasion by new endogenous retroviruses (ERVs) in lynx (Fig. 2a; Additional file 1: Section 15). Our results strongly suggest that the demographic history of the lynx had a strong impact on the TE fixation rate, in accordance with patterns reported for the human lineage [38] and for *Arabidopsis lyrata* [39].

Insertion of TEs within genes is expected to be under stronger purifying selection and more so when it occurs in sense orientation with respect to the gene because sense insertion may disrupt protein translation [40]. Consistent with this and with patterns reported for other genomes [40], LINEs and ERVs, but not SINEs, are particularly depleted within introns in felids and, when present, tend to be in antisense orientation with respect to the gene. Interestingly, lynx-specific LINEs show an increased proportion of insertion within genes in sense orientation (57 out of 112; 51%) compared to the genome-background frequency (37%; *p* = 0.004, Fisher’s exact test) (Fig. 2b). A higher proportion of in-sense LINE insertions suggests a less effective purifying selection.

Demographic bottlenecks also reduce heterozygosity and hence are expected to reduce the opportunities for GC-biased gene conversion (gBGC) [41]. The demographic history of Iberian lynx makes it an ideal case to assess the influence of demography on gBGC. We mapped nucleotide substitutions onto the phylogeny of the domestic cat and the Iberian and Eurasian lynx using tiger as outgroup. The weak-to-strong substitution bias (from A/T to G/C; hereafter W → S), a characteristic signature of gBGC, is generally weaker in the lynx ancestor than in the domestic cat lineage and becomes drastically reduced after the evolutionary split between the Iberian and the Eurasian lynx (Additional file 1: Section 16). This drastic reduction supports a remarkable role for gBGC in shaping the evolution of genomes. Interestingly, the rate of evolution was significantly reduced in both lynx species, suggesting that under population contractions genome stasis may be increased through a decrease in gBGC.

**Fig. 3:**
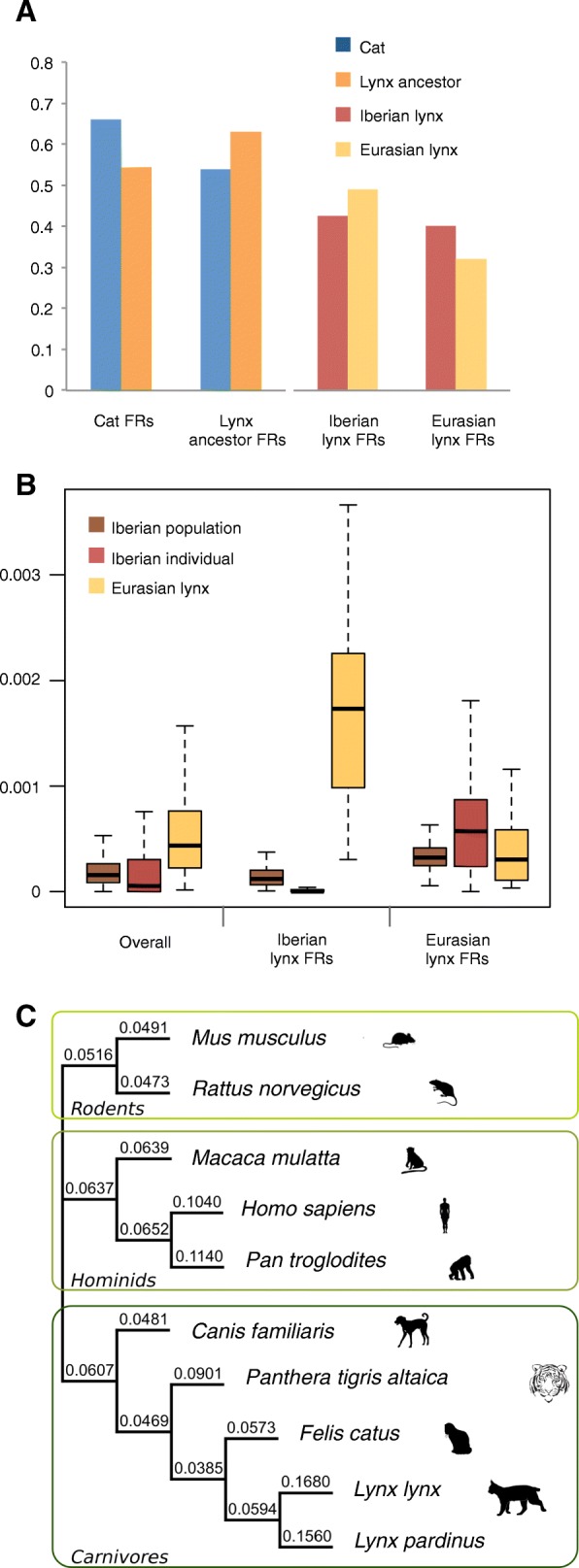
Patterns of genome evolution. **a** Magnitude of W → S bias within faster evolving regions (*FRs*) identified in pairwise comparisons between cat and the lynx ancestor and between Iberian and Eurasian lynx. **b** Heterozygosity in regions defined as FRs in Eurasian or Iberian lynx. Iberian lynx values are reported for the whole species and for the reference individual. Whereas FRs in cat and the lynx ancestor are associated with higher W → S biases, FRs in both lynx species are associated with reduced heterozygosity and fixation of ancestral polymorphisms. **c** *dN*/*dS* ratios estimated for different mammalian lineages. Increased ratios in lynx indicate the relaxation of purifying selection following the divergence of Iberian and Eurasian lynx

Regions diverging faster (FRs) identified between the cat and the lynx ancestor showed higher W → S biases in the faster evolving species (Fig. 3) and were similarly abundant in the two lineages. However, whereas FRs are distributed homogeneously along chromosomal regions in cat, in the lynx ancestor they are concentrated in subtelomeric regions (Additional file 1: Figure S32), as observed in human [42]. In contrast, FRs identified between Iberian and Eurasian lynx showed a lower W → S bias in the faster evolving species; they are instead characterized by a reduced heterozygosity (Fig. 2b). We found that these FRs are the result of the differential rate of fixation of ancestral polymorphisms in the two lynx species, which is also supported by an inverse correlation between interspecific ratios of substitution rates and of heterozygosity along the genome (*r* = −0.32, *p* value <2.2 × 10^−16^). The higher number of FRs (117 versus 46) and fixed ancestral polymorphisms within FRs (2049 versus 233) in Iberian lynx is consistent with smaller populations sizes and more severe bottlenecks in the Iberian lynx. Seventeen of the Eurasian lynx FRs versus none of the Iberian’s were located in subtelomeric regions. Since these 17 FRs did not show high W → S biases, the difference is probably not due to gBGC but to differential loss of heterozygosity in these highly polymorphic regions (see the “Genomic variation” section below).

Ratios of non-synonymous to synonymous substitution rates (*dN*/*dS*) are useful means of measuring the strength of purifying selection. Unfortunately, comparison across species is usually not possible because gene annotations differ in their qualities, diminishing the reliability of the alignments. Here, we have developed a new method to select sites aligned with the highest reliability, allowing us to conservatively filter a concatenated alignment of 8117 one-to-one orthologs from different felids, rodents, and hominids (Additional file 1: Section 13). Increased ratios in Iberian (*dN*/*dS* = 0.16) and Eurasian lynx (*dN*/*dS* = 0.17) after their divergence from their most recent common ancestor, which has a ratio similar to cat (*dN*/*dS* = 0.06), are consistent with the relaxation of purifying selection in both species. These ratios are higher than those estimated for other bottlenecked species like humans and chimpanzees (0.10 and 0.11, respectively; Fig. 3c). As most non-synonymous changes are likely to be deleterious, such high *dN*/*dS* ratios indicate a high rate of fixation of mildly deleterious mutations in both lynx species since their separation from their most recent common ancestor.

### Genomic variation

**Fig. 4:**
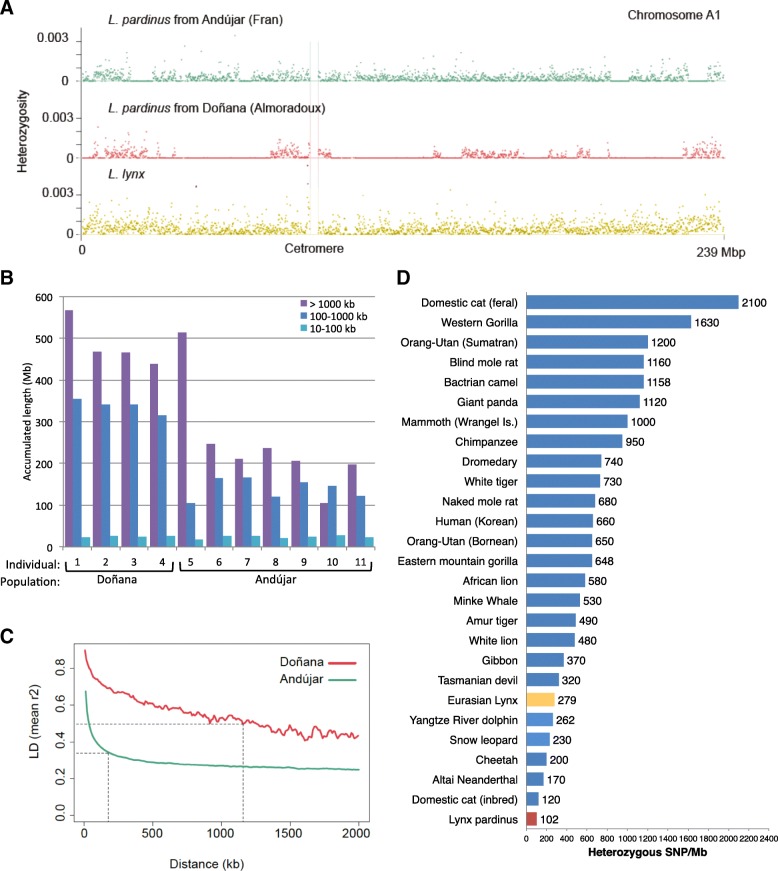
Patterns of genomic variation in 11 Iberian and one Eurasian lynx. **a** Average heterozygosity in non-overlapping syntenic 100-kb windows in one Iberian lynx from Doñana, one from Andújar (Sierra Morena) and one Eurasian lynx; chromosome A1 is shown as an illustrative example. Long runs of homozygosity are evident in the Iberian individuals. **b** Length of the genome covered by runs of homozygosity of different sizes in each Iberian lynx individual. Both large and medium size ROH are more abundant in Doñana, indicating higher inbreeding and a longer recent history of low effective size. **c** Linkage disequilibrium (*LD*) decay in Iberian lynx populations. Doñana has remained small (50–80 lynxes) and isolated at least since the 1950s, whereas Andújar was part of a large and well connected population until the 1960s; then it became progressively contracted and isolated and reached its lowest size at around 60 animals by 2002. **d** Heterozygous SNP rates in genome-sequenced mammals. Modified from Cho et al. [43] and updated with the addition of data for Altai Neanderthal [114], cheetah and feral domestic cat [115], Yangtze river dolphin [116], gibbon [117], minke whale [118], Eastern mountain gorilla [46], dromedary and Bactrian camel [119], Wrangle Is. mammoth [120], and blind mole rat [121]. The Iberian lynx genome- and species-wide SNP rate and heterozygosity are the lowest reported to date

Recent studies have revealed genome-wide signatures of inbreeding, low diversity, and accumulation of potentially deleterious variation in extinct and endangered species, with levels varying extensively, sometimes with little relationship to current demography or conservation status (e.g., [43–47]). To investigate the patterns of genomic variation we identified SNPs using whole-genome shotgun re-sequencing data for 11 Iberian and one Eurasian lynx (“Methods”; Additional file 1: Section 18). Individual Iberian lynx genomes are characterized by the abundance of long runs of homozygosity (ROH; Fig. 4a; Additional file 1: Section 19). The longest ROH (>1 Mb), which are indicative of recent inbreeding, are more abundant in the Doñana population than in the Andújar population (Fig. 4b), resulting in higher average inbreeding coefficients (*F*_*ROH-Doñana*_ = 0.32; *F*_*ROH-Andújar*_ = 0.16). Medium-length (100 kb–1 Mb) ROH are also more abundant in Doñana, consistent with its lower effective size since the two populations became effectively isolated. Finally, the extent of the genome covered by shorter ROH (10–100 kb) is similar in all individuals, suggesting a shared history of bottlenecks or low population sizes in a more distant past, when the two populations were probably part of a single ancestral population (Fig. 4b).

In line with the analysis of ROH, we found that the linkage disequilibrium (LD), measured as the squared correlation coefficient between genotypes in each individual (*r* ^*2*^), extends to long distances in Iberian lynx (Additional file 1: Appendix, Section 24). *r* ^*2*^ reaches 50% of its maximum value at a distance of 185 kb in Andújar (Fig. 4c), almost twice the average of domestic cat breeds (96 kb) and close to that observed for the highly inbred Burmese cat (249–380 kb) [48]. An even longer extent of LD was estimated for Doñana (1.2 Mb), a result that cannot be solely attributed to its smaller sample size. It must be noted that extensive LD, an additional characteristic signature of small or inbred populations, can hinder the purging of deleterious recessive alleles [49] and may thus have contributed to the accumulation of mildly deleterious mutations in Iberian lynx that we observe.

The two remnant Iberian lynx populations are strongly differentiated (*F*_ST_ = 0.22) and differ in levels of genetic diversity, with Doñana lynxes showing about half the genetic diversity detected in Andújar lynxes (Table 1; Additional file 1: Section 20). Similar patterns were recovered with polymorphic TE insertions (*PhiPT* = 0.261; *H*_*E_Andújar*_ = 0.240; *H*_*E_Doñana*_ = 0.195; Additional file 1: Section 15) and copy-number variants (Additional file 1: Section 17) and are consistent with previous studies based on microsatellite markers [10]. The average genome-wide heterozygosity rate in the Iberian lynx (102 SNPs/Mb) is the lowest reported for any mammal and is about one-third (36.6%) of that present in the Eurasian lynx (279 SNPs/Mb). Note that this figure is similar to that observed in a highly inbred domestic cat (121 SNPs/Mb) and lower than those of other highly endangered mammals [43] (Fig. 4d) or birds [44], including the endangered crested ibis (*Nipponia nippon*; 430 SNPs/Mb) or the white-tailed eagle (*Haliaeetus albicilla*; 400 SNPs/Mb) [44]. Accordingly, we also observed values of average genome-wide nucleotide diversity and synonymous nucleotide diversity that are to our knowledge the lowest reported for any organism [50] (Table 1; Additional file 1: Sections 20 and 21). At the same time, the ratio of non-synonymous to synonymous diversity is high (π_N_/π_S_ = 0.286), similar to those observed in other bottlenecked populations, such as humans (π_N_/π_S_ = 0.241) [51] or the Galápagos giant tortoise, *Chelonoidis nigra* (π_N_/π_S_ = 0.310) [52], indicating a relative abundance of potentially deleterious mutations segregating at moderate to high frequencies.

**Table 1:** Iberian lynx genetic diversity

	Doñana	Andújar	All
*N* (chromosomes)	8	14	22
Number of SNPs	625,552	1,383,709	1,587,509
*H*_*O*_ per SNP	0.178	0.317	0.266
Watterson’s Θ (%)^a^	0.012	0.022	0.022
*H*_*E*_ per SNP	0.167	0.316	0.336
*H*_*E*_ per site (π) (%)^a^	0.013	0.025	0.026
π_*S*_ (%)^b^	0.014	0.026	0.028
π_*N*_/π_*S*_^b^	0.287	0.286	0.287

^a^Per site statistics consider the universe of callable sites (2,021,732,768).

^b^Coding sequence estimates are based on 14,028 coding sequences larger than 200 nucleotides.

*H*_*O*_ observed heterozygosity, *H*_*E*_ expected heterozygosity under Hardy–Weinberg equilibrium, π nucleotide diversity, π_*S*_ nucleotide diversity at synonymous sites, *π*_*N*_ nucleotide diversity at non-synonymous sites

To assess whether different parts of the genome might have become differentially affected by genetic drift, we analyzed genetic diversity in non-overlapping 100-kb-long windows along the genome. The X chromosome has been especially depleted of genetic variation: the average X chromosome-to-autosomal normalized diversity ratios at intergenic sites were 0.35 (standard error (SE) = 0.02), 0.29 (SE = 0.02), and 0.38 (SE = 0.03) for the global, Andújar, and Doñana populations, respectively, and ratios were even smaller for coding sequence (Additional file 1: Section 22). Ratios substantially lower than the 0.75 expected at equilibrium are predicted by theory and often observed in recently bottlenecked populations [53].

We also identified regions showing the highest differences in standardized heterozygosity between Eurasian and Iberian lynx (*∆Z*_*H*_ = *Z*_*H-Eurasian*_ − *Z*_*H-Iberian*_; Additional file 1: Section 23). Windows within both the 2.5% largest negative (N = 718) and 2.5% largest positive *∆Z*_*H*_ (N = 671) were significantly depleted of genes (43.2 and 41.1% of outlier windows with genes, respectively, against 50.4% overall; Fisher’s exact tests, *p* < 0.0001) and windows with a large negative *∆Z*_*H*_ (*Z*_*H-Eurasian*_ < < *Z*_*H-Iberian*_) were more likely to be in subtelomeric regions (15 versus 6% overall; Fisher’s exact test, *p* < 0.0001). A comparatively high diversity in subtelomeric regions supports a role for recombination in maintaining genetic diversity in small or declining populations [54], either through an associated increase in mutation rates or by reducing the number of sites affected by hitch-hiking during positive or purifying selection. Regarding gene functions, a gene ontology enrichment analysis indicated that windows with the largest positive and the largest negative *∆Z*_*H*_ are both enriched for genes related to olfactory perception and G-protein signal transduction, while windows with the largest negative *∆Z*_*H*_ (*Z*_*H-Eurasian*_ < < *Z*_*H-Iberian*_) were also enriched in genes involved in pheromone reception, amino acid and glucose transmembrane transport, and the regulation of triglyceride biosynthesis genes, among others (Additional file 2: Datasheet S8). The olfactory receptor family is the largest and one of the most genetically diverse multigene families in vertebrates and its evolution has been suggested to be under the influence of balancing selection [55]. Thus, despite the extreme global loss of diversity, functional variation may have been preserved at specific loci (e.g., olfactory receptor genes) by on-going balancing selection, a hypothesis that deserves further investigation.

## Conclusions

Our analyses provided novel insights into the evolutionary and demographic history of the Iberian lynx, revealing a recent divergence and continued admixture with the Eurasian lynx and several drastic population bottlenecks in the last millennia. Such demography has shaped the patterns of nucleotide substitution and increased the fixation rate of transposable elements, whereas the predominance of genetic drift and the concomitant decrease in the efficiency of purifying selection have resulted in extremely low levels of genetic diversity and a high genetic load, indicating a severe level of genomic erosion in Iberian lynx.

The consequences of such low levels of genetic diversity for the viability of the species are hard to predict, but they are likely to limit the capacity of the Iberian lynx to adapt to environmental changes, whereas the excess of deleterious variants in combination with high inbreeding may reduce individual fitness (i.e., inbreeding depression), as suggested by recent evidence [11–15]. Current conservation efforts, including both ex situ and in situ programs, are addressing these threats by promoting the admixture of the two populations and through the genetic management of captive breeding, translocations, and reintroductions. These actions have likely contributed to the recent modest recovery of the population by generating less inbred and more genetically diverse populations that are potentially more fit.

However, these strategies cannot restore the diversity that has been definitively lost, which may limit the species adaptive potential to environmental change. Increasing the adaptability of the species would demand the careful consideration of novel but controversial genetic restoration approaches such as facilitated adaptation, genome editing, or assisted adaptive introgression [56, 57]. Although the evidence for recent natural introgression could encourage the use of Eurasian lynx as a source for the latter, such drastic measure should require the careful evaluation of hybrid fitness and the associated risks of maladaptation and hybrid swarm [58]. In any case, existing examples of species with long-term persistence and widespread distribution despite depleted genetic diversity [59] allow for some measure of hope and argue for the maintenance of current conservation efforts.

The Iberian lynx draft genome, along with the other resources generated in this study, will support the species conservation by providing more informative and efficient genetic markers for genetic monitoring and management, which is currently based on 36 microsatellite markers. A selected set of highly informative SNPs will provide more reliable and cost-effective tools for genetic monitoring from non-invasive samples (e.g., scats or hairs) and more accurate estimates of relatedness and inbreeding for an integral genetic management of the species that should cover ongoing captive breeding, translocations, and reintroductions. Most importantly, these resources will facilitate the identification and eventual management of deleterious alleles of highest impact on Iberian lynx reproduction or survival.

## Methods

### Samples

Eleven Iberian and one Eurasian lynx were sequenced in this project (Additional file 1: Table S1). A moderately inbred male born in Andújar in 2006 and kept as a founder of the captive population since then (Candiles) was selected to provide the reference genome for the species. Ten additional Iberian and one Eurasian male were sampled for whole-genome resequencing, four of them from the population in Doñana and six from Andújar. These two populations differ in their recent demography: Doñana has remained small and isolated at least since the 1950s, while Andújar is the result of the progressive contraction of the large and more connected population of Sierra Morena [60]. The Eurasian lynx is a male born in captivity in 2007 at the Zoológico de Córdoba (Spain) with no recent history of close inbreeding. Samples for DNA sequencing were obtained from blood and DNA was extracted following standard protocols. Ten organs (brain, heart, kidney, liver, lung, muscle, pancreas, spleen, stomach, and testes) were sampled for RNA sequencing from one of the Doñana Iberian lynx (Almoradux) immediately after its euthanization. Organ samples were immediately frozen in liquid nitrogen and kept at −80 °C. Total RNA was extracted by the RiboPure™ RNA Purification Kit (Ambion®).

### Genome sequencing and assembly

Genomic DNA from a single captive male lynx (*Candiles*; studbook no. 0029) was isolated and shotgun-sequenced using Illumina and 454 technology in libraries of insert sizes 500 bp, 4.5 kb, and 5.2 kb. At the time, larger-insert mate-pair libraries, while desirable, were difficult to obtain and we opted for a fosmid-pool strategy [17]. Ninety fosmid pools of 1200 clones prepared using the NxSeq 40 kb Mate-Pair Cloning Kit (Lucigen Corporation, USA) were used for fosmid-end and fosmid-pool sequencing. The 115,000 clones represented an approximately 1.6-fold physical coverage of the genome. Each pool was shotgun sequenced to greater than 100× depth and assembled independently. The resulting contigs were then merged to obtain an assembly representing approximately 67% of the estimated size of the genome. The remaining portion of the genome was assembled using whole-genome shotgun (WGS) paired-end (PE) data. Both partial assemblies were combined by scaffolding with WGS PE and mate-pair data, followed by extra scaffolding steps using RNA-seq and fosmid end data. The CEGMA pipeline was used to determine the state of the gene space as an indicator of genome completeness [18] (Additional file 1: Sections 1 and 2).

### Transcriptome analyses

Total RNA was extracted by the RiboPure™ RNA Purification Kit (Ambion®) from ten organs (brain, heart, kidney, liver, lung, muscle, pancreas, spleen, stomach, and testes) sampled immediately after the euthanasia of one Iberian lynx (*Almoradux*) and from the blood of *Candiles*. Sequence libraries were prepared using the mRNA-Seq sample preparation kit (Illumina Inc., catalog number RS-100-0801). Reads were aligned to the reference assembly using GEMTools RNAseq pipeline v.1.6.2. Flux Capacitor v.1.2.4 [61] was used to quantify genes, transcripts, exons, and splice junctions in each sample separately. Expression levels were obtained in pure read counts and in reads per kilobase per million mapped reads (RPKM) [62]. Differential gene expression (DGE) analysis across tissues was performed with Bioconductor package edgeR v.3.4.2 (R v.3.0.2) [63] using classic pairwise comparison. A comparative gene expression analyses was performed using data from Brawand et al. [64] and RNA-seq of testis transcriptome from the domesticated cat, *F. catus* (Sequence Read Archive experiment ID SRX193575) using NOISeq Bioconductor package v.2.6.0 [65] (Additional file 1: Section 6).

### Genome annotation

Transposable elements and other repeats were annotated with RepeatMasker (version open-4.0.1) [66], using rmblastn v2.2.27 search engine and RM database v20120418, with the *F. catus* library of repeats and the sensitive search option. Low-complexity regions were identified with DustMasker v.2.2.28 [67] with default parameters. Protein-coding genes were then annotated by combining transcriptome evidence with homology-based and ab initio gene prediction methods (Additional file 1: Section 3). Ab initio gene predictions were performed on the TE-masked assembly using *Genid*, *SGP2*, *GlimmerHMM*, and *Augustus*. A combination of the Program to Assemble Spliced Alignments (PASA r2012-06-25) and Evidence Modeler (EVM r2012-06-25) [68] was used to obtain consensus coding sequence (CDS) models using three main sources of evidence: aligned transcripts, aligned proteins, and gene predictions. Small structured non-coding RNAs were detected using the CMsearch tool from the Infernal package (version 1.1rc2) [69] against the Rfam database (version 11) [70]. Long non-coding RNAs (lncRNAs) were predicted by homology using the strategy reported in [71, 72] and by ab initio approaches using *Geneid* to generate a final set consisting of transcripts that are either expressed or conserved in at least one species (Additional file 1: Section 7).

### Functional annotation

We used our own automatic functional annotation pipeline based on Interproscan [73], KEGG [74], and Reactome [75] and Blast2GO [76] to assign a description (e.g., the protein name) and relevant annotation through sequence similarity and Gene Ontology-based data mining (Additional file 1: Section 4). SignalP [77] was used to predict the presence and location of signal peptide cleavage sites. Finally, in order to organize, store, and facilitate the access to the entire set of annotations we have developed a MySQL (http://www.mysql.com/) relational database. The modules implemented in APPRIS (http://appris.bioinfo.cnio.es/docs/appris.html) were used to map a range of conserved protein features to the splice variants annotated for each gene and to determine which of these is the main (principal) gene variant. The number of genes annotated with protein features is similar to that of the human genome, but less Iberian lynx genes align full length and without gaps to orthologs in other species or contain signal sequences. The main protein isoform could be identified for the majority of lynx genes with multiple variants (3408 of 5218 genes, 64.9%) and 8066 variants were tagged as alternative. A computer-assisted manual annotation of the degradome [78], the complete repertoire of proteases in the organism, found almost all of the 635 expected proteases, of which 306 were completely annotated, confirming a good gene coverage of the Iberian lynx genome (Additional file 1: Section 5).

### Orphan genes

We developed a pipeline to identify lynx orphan protein-coding genes (Additional file 1: Section 8). First, we discarded any proteins that had homologs in any of 23 non-mammalian eukaryotic species, using gene protein coding annotations from Ensembl. To search for homologs we used BlastP (2.2.23+) [79] with an E-value threshold of 10^−4^ and the filter for low complexity regions activated. Second, we discarded any proteins for which we could indirectly trace homology to other species through a second protein in lynx. This could happen, for example, if the protein had evolved very rapidly after a gene duplication event [80]. For these searches we used BlastP with the same parameters as previously except that we used a BLOSUM80 matrix instead of the default BLOSUM62, as we were searching for sequences that had diverged relatively recently. Third, we classified the remaining proteins as lynx-specific or mammalian-specific depending on the presence of homologs in the annotated genes from *F. catus*, *C. lupus familiaris*, *A. melanoleuca*, *Mustela putorius furo*, *H. sapiens*, *M. musculus*, *Bos taurus*, *Equus ferus caballus*, and *Myotis lucifugus* (Ensembl version 72). Fourth, we only selected those genes expressed in at least one tissue using a RPKM threshold of 0.3. This resulted in the identification of 323 lynx-specific genes. The current gene catalogs are likely to be incomplete and this means that some of these 323 putatively lynx-specific genes may correspond to not yet annotated genes in other mammals. We thus employed published RNA-seq data for different tissues and mammalian species [64] to have a more comprehensive set of transcripts to compare our genes with. We ran TopHat2 v.2.0.8 [81] for pooled-tissue reads from human, mouse, chimpanzee, macaque, and orangutan. Next, all long expressed transcripts (length >200 nucleotides) were assembled using Cufflinks (v.2.0.2) [81] for each species and tissue separately, not using information from gene annotations (no reference GTF file). We used Cuffmerge to obtain a comprehensive set of transcripts for each species and Cuffcompare to classify the transcripts into already known transcripts (annotated, using GTF files corresponding to Ensembl v.60) and novel transcripts (non-annotated). Finally, we ran tBlastX with an E-value threshold of 10^−6^ to search for homologs of the 323 putative lynx orphan genes among these transcripts. After discarding any gene that had at least one match, the list of lynx orphan genes was reduced to 204 (206 transcripts).

### Evolutionary and demographic history

We extracted the autosomal contigs and obtained maximum likelihood estimates for either the isolation model of Mailund et al. [19] or the initial migration model of Mailund et al. [20] (Additional file 1: Section 9). As in Mailund et al. [20] we used Akaike’s information criterion (AIC) to determine the most probable model. To estimate parameters we used a Nelder-Mead optimization as implemented in scipy’s optimization module. The scripts used were “isolation-model.py” and “initial-migration-model.py” from https://github.com/mailund/IMCoalHMM. We split the autosomal contigs into 44 sets each covering ~100 Mbp and estimated the uncertainty in the parameter estimates using a leave-one-out jackknife approach. We used two complementary approaches to infer the demographic history of Iberian lynx (Additional file 1: Section 10). The first uses a pairwise sequentially Markov coalescent (PSMC) model applied to complete diploid genome sequences of single individuals to reconstruct the demographic history of the species from the distribution of the local density of heterozygous sites [21]. The method seems to work well for periods between 10,000 to 1 million years b.p. but tends to overestimate recent population sizes and to spread sudden changes in population size over several preceding tens of thousands of years. For the second approach we used the maximum likelihood inference method implemented in the software *∂a∂i*, which searches for the most recent demographic history that better fits the observed allelic frequency spectrum (AFS) [22]. In *∂a∂i* we evaluated either a single or two demographic changes, allowing them to be instantaneous or exponential and chose the best-fit model using the AIC.

### Karyotype

Cells from a primary Iberian lynx fibroblast cell line were harvested at early passages and chromosomal preparations were obtained following standard protocols. Metaphases were stained homogenously with Leishman solution for the analysis of diploid number (2n) and the number of autosomal chromosome arms (NFa) and then G-banded with Wright’s stain following the methods described by [82] for karyotyping. For each staining, at least 30 metaphase spreads were analyzed. The karyotype of the Iberian lynx was arranged following the cat chromosomal nomenclature [83] and compared to previously published karyotypes for domestic cat and Eurasian lynx [28, 84]. For telomere detection fluorescence in situ hybridization (FISH) analysis was performed using a peptide nucleic acid (PNA) probe complementary to the telomere G-rich strand (TelC; Panagene, Yuseong-gu, Daejeon, Korea) according to the manufacturer’s protocol (Additional file 1: Section 11).

### Synteny

We built several pairwise alignments between lynx, cat, tiger, and dog genomes using LAST v.458 [85]. For each scaffold, we sorted all the best-hit alignments of length >1000 bp based on the corresponding cat genome coordinates. Then, all the alignments that were less than 20 kb apart and lay on the same strand were merged with bedtools [86]. We retained only those chained alignments that were at least 15 kbp long and for which at least 40% of the sites in the region were aligned. Finally, we explored the resulting chained alignments to detect inversions and inter/intra-chromosomal rearrangements. We used the dog genome as outgroup to determine whether the potential rearrangements took place in the cat or in the lynx branches. To filter rearrangements that may be assembly artifacts we required that at least one scaffold derived from fosmid sequencing crossed the predicted breakpoint. We tested the scaffold integrity by performing long-range PCRs with primers flanking the inferred breakpoint on the reference genome, followed by Sanger sequencing. Out of 15 potential rearrangements tested, eight were empirically validated by this approach (Additional file 2: Datasheet S3; Additional file 1: Section 12).

### Phylogenomics

The Iberian lynx phylome (i.e., the complete collection of phylogenetic trees for each gene encoded in the genome) was reconstructed using the PhylomeDB pipeline [87]. We used 15 mammalian species for expansion and pseudogene analyses and 17 for *dN*/*dS* estimation (Additional file 1: Section 13). Maximum likelihood (ML) trees were reconstructed based on the codon alignments using codonPhyML v.1.0 [88] with GY as codon substitution model and F3X4 as model for defining the codon frequency from the alignment. The resulting phylome was used to infer orthology and paralogy relationships. For each tree, ETE v.2 [89] was used to identify duplication and speciation nodes along the trees using a species overlap approach and a species overlap score of 0, as described by Huerta-Cepas et al. [90]. All orthology and paralogy relationships are available through PhylomeDB [91]. Gene Ontology enrichment analysis was performed using FatiGO [92]. To find putative pseudogenized genes in lynx, domestic cat genes showing no homologs in the Iberian lynx genome, even when relaxing the overlap threshold to 20%, and that had homologs in at least four additional species were searched against the lynx genome using tBlastn [79]. Cat proteins with significant (e-value <10^−5^) hits in the lynx genome aligning over 30% of their length were selected for further inspection. The genomic region determined by the blast search was extended by 10,000 nucleotides at both sides and exonerate-based gene prediction [93] was performed on the region using the cat protein as a seed; 85 predictions interrupted by stop codons were considered as putative pseudogenes.

We used 8117 sets of one-to-one orthologs comprising proteins from five carnivore species (*L. pardinus*, *L. lynx*, *F. catus*, *P. tigris*, and *C. familiaris*), three primates (*H. sapiens*, *P. troglodytes*, and *M. mulatta*), and two rodents (*M. musculus* and *R. norvegicus*) to estimate *dN*/*dS* ratios for different branches of the extended reference species tree (Fig. 3c, main text). To reduce the impact of alignment errors, the trimmed alignments used to reconstruct single gene trees were further filtered using an automated script (selective_trimming_for_dNdS_analyses.based_neighbours.py) available at the official trimAl repository in GitHub (https://github.com/scapella/trimal). Firstly, codon columns containing gaps were removed. Secondly, we scanned the corresponding translated alignments looking for columns with at least one amino acid replacement and only those surrounded by two previous and two posterior fully conserved sites were retained. Resulting alignments were concatenated and the number of nonsynonymous substitutions per nonsynonymous site (*dN*), synonymous substitutions per synonymous site (*dS*), and the corresponding *dN*/*dS* ratio (ω) were estimated for each branch using the ML method implemented in the CodeML program of PAML v.4.4. [32]. For this analysis, we used a (1) fixed topology according to the extended species tree, (2) F3X4 as model of codon frequency, and (3) a free-omega model (model = 1) so an independent ratio for each branch is assumed.

We also produced a dated Felidae phylogeny based on filtered whole-genome alignments of available felid genomes (domestic cat, Iberian lynx, Eurasian lynx, tiger, lion, snow leopard, and cheetah; Additional file 1: Section 13.8). We analyzed the alignment using *Saguaro* to identify chromosomal regions with discrete phylogenetic signals that are different from the background signal [94] (Additional file 2: Datasheet 6), constructed a maximum likelihood tree in RAxML [95], and used the topology matching the species tree, which was also the most frequent one, to estimate divergence times from whole-genome alignment with *MCMCtree* [32].

### Positive selection

We looked for signatures of positive selection in the lynx lineage using a set of 9695 one-to-one orthologs generated in the phylogenomics analyses (Additional file 1: Section 14). We selected eight different species: *P. tigris*, *F. catus*, *L. lynx*, *L. pardinus*, *A. melanoleuca*, *C. lupus familiaris*, *H. sapiens*, and *M. musculus*. We performed multiple sequence alignments with the software PRANK [96] and conducted a branch-site test of positive selection (PS) [32] using information from Timetree (http://www.timetree.org/) for the input tree. We filtered out cases with more than one site with a probability of being under positive selection higher than 0.99 by the Bayes empirical Bayes (BEB) approach as they typically corresponded to non-homologous stretches [97]. We manually validated 100 lynx positive selection candidates (96 for *Lynx* sp. and four for *L. lynx*; Additional file 2: Datasheet S7). We used Gitools [98] and annotations from Ensembl version 73 [99] to perform an enrichment analysis in the set of positively selected genes.

### Transposable elements

Genomes of lynx, cat, and tiger were pairwise aligned using LAST [85] with the aim of identifying orthologous regions between them (Additional file 1: Section 15). We analyzed unambiguously aligned regions for each pair of species (lynx–cat, lynx–tiger, cat–tiger) to identify strongly supported gaps. Every gap in which a particular TE covered at least 95% of the gap, 99% of that TE was within the gap, and in which target-site duplications (TSDs) were detected at each gap boundary, was considered as a species-specific TE insertion. TSD were defined by obtaining −25/+15 and −15/+25 bp around the start and end site coordinates of the TE, respectively. Then, both sequences were compared to each other with BLAST and we required that L*P/100 was greater than 6, where L is the length of the alignment and P the percentage of identity. This procedure allowed the identification of short interspersed element (SINE) and long interspersed element (LINE) insertions, as they leave clear TSDs of size ~20 bp. To analyze the accumulation of TEs along the branches of the tree that relates lynx, cat, and tiger, we relied on the pairwise comparisons between lynx and cat and between tiger and cat. Every TE insertion was mapped onto the domestic cat genome to analyze the patterns of insertion within genes.

To determine the activity of endogenous retroviruses (ERVs) in lynx, we relied on a combined approach based on synteny analyses and phylogenetic reconstruction. First, we annotated the set of endogenous retroviruses in lynx, cat, and tiger. To reconstruct full ERVs we post-processed RepeatMasker results and searched for pairs of long-terminal repeats (LTRs) that (1) were of the same type and (2) were on the same strand and (3) for which at least 50% of the LTR-enclosed sequence corresponded to ERV fragments of the same family and orientation. Finally, when ERV candidates overlapped, we retained only one of them. By doing this, we were able to reconstruct 1776, 1895, and 1940 full-ERV candidates in lynx, cat, and tiger, respectively. We built a phylogenetic tree for all these ERVs using the BioNJ method [100] (Additional file 1: Section 15).

### Substitution patterns

To identify and polarize substitutions in Eurasian and Iberian lynxes we called variants with the RubioSeq pipeline [101] using the genome of domestic cat (version 6.2, felcat5) as reference (Additional file 1: Section 16). Based on the genotype of each lynx species, we selected all those sites, either variant or invariant with respect to cat, which were reliably predicted in both species in homozygosis (heterozygous sites were treated separately). The resulting dataset encompassed 2.15 billion genotyped base pairs. To infer ancestral character states, we focused on the set of sites lying on regions for which orthology was successfully established between lynx, cat, and tiger; we excluded sites lying on repeats and/or low-complexity regions. The final dataset contained 1,062,208,795 genotyped sites and included: 712,201 and 707,025 variants specific for Iberian (*L. pardinus*) and Eurasian (*L. lynx*) lynx, respectively; 9,687,075 variants shared by the two (substitutions occurring since the divergence of cat and lynxes until the divergence of Iberian and Eurasian lynxes); and 1,051,102,494 shared invariant sites. Identified substitutions were used to estimate substitution rates, non-synonymous to synonymous substitution ratios (*dN*/*dS*), and weak-to-strong (mutations from A/T to G/C; hereafter W → S) substitution biases. We translated cat genome coordinates to lynx scaffold coordinates (based on the genome alignments) to annotate the effect of substitutions on lynx protein-coding genes using SnpEff v.3.5 [102] and based on the principal transcript isoforms identified with APPRIS [103]. Substitutions were condensed into non-overlapping 100-kbp windows containing at least 10,000 informative sites to analyze the patterns of evolution along chromosomes.

### Segmental duplications

We detected segmental duplications (SD) in the genomes of one Eurasian lynx (*L. lynx*) and 11 Iberian lynxes (*L. pardinus*) both from Sierra Morena (7) and from Doñana (4) (Additional file 1: Section 17). Illumina 100-bp reads were mapped to the repeat-masked Fca-6.2 (UCSC felCat5) domestic cat assembly using BWA [104] (using as parameters “bwa -q 15”) and duplicated reads were removed with SAMtools [105]. Successfully mapped reads in the resulting BAM files were then used to recover the original FASTQ files using the bam2fastq tool (http://gsl.hudsonalpha.org/information/software/bam2fastq). The final set of 100-bp reads were clipped to 36-bp fragments but only retaining positions in the read with high quality, which was assessed with fastqc (http://www.bioinformatics.babraham.ac.uk/projects/fastqc/). The resulting 36-bp reads were then mapped to the reference assembly using mrFast [106] (using as mapping parameters “–e 2”). mrCaNaVaR (v.3.0.1) [107] was used to estimate the copy number along the genome from the mapping read depth. Mean read depth per base pair was calculated in 1-kbp non-overlapping windows and a control read depth distribution was obtained by iteratively excluding windows with extreme read depth values relative to the normal distribution. The mean read depth in these control regions was considered to correspond to copy number equal to two and was used to convert the read depth value in each window into a GC-corrected absolute copy number in each sample. We called SDs in each individual as genomic regions in which the predicted copy number significantly exceeded diploidy, while accounting for the technical variation in the copy number predictions across samples. Finally, we filtered out SDs shorter than 10 kbp and with >85% of their size overlapping with repeats.

### Variant calling

To generate variation data for population genomics and species divergence analyses we re-sequenced ten Iberian (mean depth 26.4×) and one Eurasian lynx (64×) (Additional file 1: Section 18). Variant discovery and genotyping were performed using a mapping-based approach as implemented in the RUbioSeq suite [101], using either the Iberian lynx or the domestic cat genome as references and different sets of samples. In addition, we applied the reference-free and assembly-based strategy implemented in *Cortex_var* [108]. The two procedures yielded largely concordant results but, as expected by its higher sensitivity to detect singletons, the mapping-based calling dataset yielded more variants, slightly higher diversity estimates, and more reliable estimates of LD and homozygosity blocks and was the one used for population genomic analyses.

### Population genomics

We used VCFTools v.0.1.10 [109] to identify runs of homozygosity (ROH) in each Iberian lynx individual, to estimate individual inbreeding coefficients from the extent of ROH larger than 1 Mbp (*F*_*roh*_) and from the observed homozygosities and allele frequencies (*F*_h_) (Additional file 1: Section 19), and to compute diversity and differentiation parameters (Additional file 1: Section 20). Estimates were averaged for all variants and converted to per-site averages using the number of reliably called invariant sites. For coding sequences we counted synonymous and non-synonymous variants as annotated by SnpEff v.3.5 [102] (*P*_*s*_, *P*_*n*_) and calculated the per-site synonymous and non-synonymous nucleotide diversity (π_S_, π_N_) by assuming that three-quarters of all of the sites are non-synonymous. The genomic averages of π_S_ and π_N_ were calculated by averaging across CDS with more than 200 callable sites weighted by the number of sites; genomic ratios were calculated from these averages (Additional file 1: Section 21). To study the distribution of diversity along and across chromosomes, we considered genomic regions with conserved synteny to the domestic cat genome (felCat 6.2) so that we could assign lynx regions to specific chromosomal locations and obtain estimates of cat–lynx divergence (Additional file 1: Sections 22 and 23). We excluded repeats, low complexity regions, centromeres, and telomeres along with 2 Mb of flanking regions, and the pseudoautosomal region 1 (PAR1) in the X chromosome (first 6 Mb), and defined non-overlapping 100-kb-long syntenic windows. For each of these windows we estimated the nucleotide diversity (π, nucleotide diversity) and the divergence to cat (*D*, the observed fraction of fixed differences), and the ratio between the two (π/*D*) was used as a measure of diversity normalized by mutation rate. Standard errors were calculated by bootstrapping over windows or CDS as implemented in the *boot* package for R [110, 111] to account for the correlation among nearby sites due to LD (Additional file 1: Section 22).

To characterize genomic patterns of diversity in the Iberian lynx genome in comparison to the Eurasian lynx genome, for each window we calculated the Z-transformed per-site average of the observed heterozygosity in Eurasian (*Z*_*H-EL*_) and in Iberian lynx (*Z*_*H-IL*_) and its difference (*∆Z*_*H*_ *= Z*_*H-EL*_ *− Z*_*H-IL*_). Windows with a *∆Z*_*H*_ value higher than the 99.9th or equal to or lower than the 0.1th percentile were identified as outliers. We then tested whether outlier windows were preferentially located in subtelomeric regions (within 5 Mb from the end of chromosomes) or were significantly enriched for particular cellular components, biological processes, or molecular functions by performing a Gene Ontology analyses using FatiGO [112], as implemented in Babelomics 4.3 [113].

## Additional files

Additional file 1:
**Supplemental information.** Additional details on methods and results, including additional tables (Tables S1–S37) and figures (Figures S1–S45). **Section 1:** Samples, libraries and sequencing. **Section 2**: Genome assembly. **Section 3:** Genome annotation. **Section 4:** Functional annotation. **Section 5:** Manual annotation and comparative analysis of lynx protease genes. **Section 6:** Transcriptome characterization. **Section 7:** Evolutionary profiling and expression of lncRNAs. **Section 8**: Lynx orphan genes. **Section 9:** Eurasian and Iberian lynx divergence **Section 10:** Demographic history. **Section 11:** Karyotype. **Section 12:** Genome alignments and synteny analysis. **Section 13:** Phylogenomics. **Section 14:** Positive selection. **Section 15:** Transposable elements dynamics. **Section 16:** Substitution patterns. **Section 17:** Segmental duplications. **Section 18:** Variant discovery and genotype calling. **Section 19:** Runs of homozygosity (ROH) and individual inbreeding. **Section 20:** Genomic averages of population genetics parameters. **Section 21:** Variation and divergence at coding sequences. **Section 22:** X chromosome versus autosomes genetic diversity. **Section 23:** Patterns of diversity across the genome. **Section 24:** Linkage disequilibrium. **Section 25:** The Iberian lynx genome browser. **Section 26:** References. (PDF 10 MB)
Additional file 2:
**Supplemental datasheets. Datasheet S1:** Summary of the annotation of the human and felid degradomes. **Datasheet S2:** List of orphan genes with expression levels in different organs. **Datasheet S3:** List of potential chromosomal rearrangements with respect to domestic cat. **Datasheet S4:** Gene Ontology terms enriched in proteins that duplicated at specific points in the lynx, cat, and tiger phylogeny. **Datasheet S5:** List of putative pseudogenes. **Datasheet S6:** Partitions identified by Saguaro as yielding alternative topologies from a whole genome alignment of Felidae. **Datasheet S7:** List of genes with signatures of positive selection. **Datasheet S8:** List of Gene Ontology terms significantly enriched in windows of high and low diversity in Iberian lynx populations or of high of low difference with respect to Eurasian. (XLSX 340 kb)
