# Supplementary material for: Extreme genomic erosion after recurrent demographic bottlenecks in the highly endangered Iberian lynx
Source: Genome Biol. 2016 Dec 14;17:251. doi: 10.1186/s13059-016-1090-1 (PMC5155386; doi:10.1186/s13059-016-1090-1)
Supplement: Additional file 1: — Supplemental information. Additional details on methods and results, including additional tables (Tables S1–S37) and figures (Figures S1–S45). Section 1: Samples, libraries and sequencing. Section 2: Genome assembly. Section 3: Genome annotation. Section 4: Functional annotation. Section 5: Manual annotation and comparative analysis of lynx protease genes. Section 6: Transcriptome characterization. Section 7: Evolutionary profiling and expression of lncRNAs. Section 8: Lynx orphan genes. Section 9: Eurasian and Iberian lynx divergence Section 10: Demographic history. Section 11: Karyotype. Section 12: Genome alignments and synteny analysis. Section 13: Phylogenomics. Section 14: Positive selection. Section 15: Transposable elements dynamics. Section 16: Substitution patterns. Section 17: Segmental duplications. Section 18: Variant discovery and genotype calling. Section 19: Runs of homozygosity (ROH) and individual inbreeding. Section 20: Genomic averages of population genetics parameters. Section 21: Variation and divergence at coding sequences. Section 22: X chromosome versus autosomes genetic diversity. Section 23: Patterns of diversity across the genome. Section 24: Linkage disequilibrium. Section 25: The Iberian lynx genome browser. Section 26: References. (PDF 10 MB) [file 13059_2016_1090_MOESM1_ESM.pdf]

Supplemental Information

Extreme genomic erosion after recurrent demographic bottlenecks in the highly endangered Iberian lynx

Federico Abascal[1,*], André Corvelo[2,*], Fernando Cruz[3,2,*], Jose L. Villanueva-Cañas[4], Anna Vlasova[5,6], Marina Marcet-Houben[5,6], Begoña Martínez-Cruz[3], Jade Yu Cheng[7], Pablo Prieto[5,6], Víctor Quesada[8], Javier Quilez[9] , Gang Li[10], Francisca García[11], Miriam Rubio-Camarillo[1], Leonor Frias[2], Paolo Ribeca[2], Salvador Capella-Gutiérrez[5,6], José M. Rodríguez[12,1], Francisco Câmara[5,6], Ernesto Lowy[13], Luca Cozzuto[13], Ionas Erb[5,6], Michael L. Tress[1], Jose L. Rodriguez-Alés[5,6], Jorge Ruiz-Orera[4], Ferran Reverter[5,6], Mireia Casas-Marce[3], Laura Soriano[3], Javier R. Arango[8], Sophia Derdak[2], Beatriz Galán[14], Julie Blanc[2], Marta Gut[2], Belen Lorente-Galdos[9] , Marta Andrés-Nieto[15], Carlos López-Otín[8], Alfonso Valencia[1,12] , Ivo Gut[2], José L. García[14], Roderic Guigó[5,6,16], William J. Murphy[10], Aurora Ruiz-Herrera[15,17], Tomás Marques-Bonet[2,9,18] , Guglielmo Roma[13], Cedric Notredame[5,6], Thomas Mailund[7], M.Mar Albà[4,6,18], Toni Gabaldón[5,6,18], Tyler Alioto[2], José A. Godoy[3]

[1] Structural Biology and Biocomputing Programme, Spanish National Cancer Research Centre (CNIO), Madrid 28029, Spain
[2] CNAG-CRG, Centre for Genomic Regulation (CRG), Barcelona Institute of Science and Technology (BIST), Baldiri i Reixac 4, 08028 Barcelona, Spain
[3] Department of Integrative Ecology, Doñana Biological Station (EBD), Spanish National Research Council (CSIC), C/ Americo Vespucio, s/n, 41092 Sevilla, Spain
[4] Evolutionary Genomics Group, Research Programme on Biomedical Informatics (GRIB), Hospital del Mar Research Institute (IMIM), Dr. Aiguader 88, 08003 Barcelona, Spain
[5] Bioinformatics and Genomics Programme, Centre for Genomic Regulation (CRG), Dr. Aiguader 88, 08003 Barcelona, Spain.
[6] Universitat Pompeu Fabra (UPF), Dr. Aiguader 88, 08003 Barcelona, Spain.
[7] Bioinformatics Research Centre, Aarhus University, C.F. Møllers Allé 8, 8000 Aarhus, Denmark
[8] Departamento de Bioquímica y Biología Molecular, Instituto Universitario de Oncología (IUOPA), Universidad de Oviedo, 33006 Oviedo, Spain
[9] Institut de Biologia Evolutiva (UPF-CSIC), Universitat Pompeu Fabra, PRBB, Doctor Aiguader, 88, 08003 Barcelona, Spain.
[10] Department of Veterinary Integrative Biosciences, College of Veterinary Medicine, Texas A&M University, College Station, TX 77843, USA
[11] Servei de Cultius Cel.lulars (SCC, SCAC), Universitat Autònoma de Barcelona, Barcelona, Spain
[12] National Bioinformatics Institute (INB), Spanish National Cancer Research Centre (CNIO), Madrid 28029, Spain
[13] Bioinformatics Core Facility, Centre for Genomic Regulation (CRG), Dr. Aiguader 88, 08003 Barcelona, Spain.
[14] Department of Environmental Biology, Center for Biological Research (CIB), Spanish National Research Council (CSIC), Ramiro de Maeztu 9, 28040 Madrid, Spain
[15] Institut de Biotecnologia i de Biomedicina, Universitat Autònoma de Barcelona, 08193 Cerdanyola del Vallès, Spain
[16] Computational Genomics Group, Research Programme on Biomedical Informatics (GRIB), Hospital del Mar Research Institute (IMIM), Dr. Aiguader 88, 08003 Barcelona, Spain
[17] Departament de Biologia Cel.lular, Fisiologia i Immunologia. Universitat Autònoma de Barcelona, 08193 Cerdanyola del Vallès, Spain
[18] Institució Catalana de Recerca i Estudis Avançats (ICREA), Pg. Lluís Companys 23, 08010 Barcelona, Spain

TABLE OF CONTENTS

1 Samples, libraries and sequencing 4

2 Genome Assembly 12

3 Genome annotation 26

4 Functional annotation 32

5 Manual annotation and comparative analysis of lynx protease genes 43

6 Transcriptome characterization 45

7 Evolutionary profiling and expression of lncRNAs 55

8 Lynx orphan genes 59

9 Eurasian and Iberian lynx divergence 64

10 Demographic history 68

11 Karyotype 74

12 Genome alignments and synteny analysis 77

13 Phylogenomics 80

14 Positive selection 88

15 Transposable elements dynamics 89

16 Substitution patterns 99

17 Segmental duplications 107

18 Variant discovery and genotype calling 111

19 Runs of Homozygosity (ROH) and individual Inbreeding 121

20 Genomic averages of population genetics parameters 124

21 Variation and divergence at coding sequences 128

22 X chromosome versus autosomes genetic diversity 131

23 Patterns of diversity across the genome 133

24 Linkage disequilibrium 139

25 The Iberian lynx genome browser 141

26 References 142

1 Samples, libraries and sequencing

1.1 Samples

Eleven Iberian and one Eurasian lynx were sampled for this project (Table S1). A moderately inbred male born in Andújar in 2006 and kept as a founder of the captive population since then (Candiles) was selected to provide the reference genome for the species. Ten additional Iberian and one Eurasian male were sampled for whole genome resequencing. The two extant populations of Iberian lynx in Doñana and Andújar are represented by four and seven individuals, respectively. These two populations differ in their recent demography: Doñana has remained small and isolated at least since the 1950s, while Andújar is the result of the progressive contraction of the large and more connected population of Sierra Morena [1]. Sampled animals represent the composition of these populations prior to their intentional admixture, initiated in 2007 with the translocation of one male from Andújar to Doñana. Animals were chosen to avoid the inclusion of close relatives based on microsatellite genotypes. All Iberian lynx were wild-born individuals and most were held in captivity as founders of the captive population, so extensive phenotypic information is already available and repeated sampling is possible. The Eurasian lynx is a male born in captivity in 2007 at the Zoológico de Córdoba (Spain) with no recent history of close inbreeding. Samples for DNA sequencing were obtained from blood and DNA was extracted following standard protocols.

Ten organs (brain, heart, kidney, live, lung, muscle, pancreas, spleen, stomach, and testes) were sampled for RNA sequencing from one of the Doñana Iberian lynx (Almoradux) immediately after its euthanization, which was motivated by the severe status of a chronic renal disease caused by hipervitaminosis. Organ samples were immediately frozen in liquid nitrogen and kept at -80ºC. Total RNA was extracted by the RiboPure™ RNA Purification Kit (Ambion®). All samples

were collected by expert veterinarians and under permissions of the competent administration; CITES permits were obtained when necessary.

Table S1. Samples used in this study.

Name	Species	Birth pop	Birth year	Sex	Founder
Almoradux	*L. pardinus*	Doñana	2004	Male	Y
Arcex	*L. pardinus*	Andújar	2004	Male	Y
Beta	*L. pardinus*	Andújar	2005	Male	Y
Borja	*L. pardinus*	Doñana	1987	Male	N
Candiles	*L. pardinus*	Andújar	2006	Male	Y
Daman2	*L. pardinus*	Andújar	2007	Male	Y
Fran	*L. pardinus*	Andújar	2002	Male	Y
Gazpacho	*L. pardinus*	Doñana	2010	Male	Y
Jeme	*L. pardinus*	Andújar	2004	Male	Y
Jub	*L. pardinus*	Andújar	2000	Male	Y
Pavon	*L. pardinus*	Doñana	2003	Male	N
Ambar	*L. lynx*	Captivity	2007	Male	-

1.2 Genomic libraries

1.2.1 Immortalization of fibroblast lynx cells

Fibroblast cells from Iberian lynx (*Lynx pardinus*) obtained by skin fine-needle aspiration biopsy were cultured by standard procedures in F12 medium supplemented with 15% serum. The primary lynx fibroblast cell line named Lyp221 was transformed using the T22 plasmid that contains the T antigen under the control of SV40 promoter (kindly supplied by Dr S. Rodríguez de Cordoba at CIB-CSIC, Madrid, Spain), which transforms the cells into tumor cell lines [2]. Lipofectamine reagent was used for the transformation. Four transformed cell lines were able to grow up during at least 15 passes. Using the transformed clonal cell lines we can obtain about 250,000 cells per plate. These cells were used as a source of genomic DNA. The primary lynx fibroblast cell line was used to obtain the lynx karyotype (Section 11).

1.2.2 Construction of fosmid library

A fosmid library was prepared using the NxSeq 40 kb Mate-Pair Cloning Kit (Lucigen Corporation, USA). Lynx genomic DNA was extracted from fibroblast cultured cells using the DNeasy Blood and Tissue Kit (Quiagen, Germany). Genomic DNA fragments of lynx prepared by random shearing, end-repair (DNATerminator End Repair reaction), and size selection to 35–45 kb were inserted by blunt-end ligation into the blunt and dephosphorylated pre-cut *Pml*I site of pNGS™ FOS vector according to the supplier instructions (Lucigen Corporation, USA). The fosmid ligation reaction was packaged in lambda phages according to the packaging extract instructions of the Gigapack III XL-11 kit (Agilent Technologies, USA). Phage transfection was performed using the Lucigen's Replicator FOS strain which contains an inducible *trfA* gene, which is required for induction of the *oriV* origin, resulting in amplification of the pNGS FOS clones. The fosmid library (about 120,000 clones) was plated on 95 agar plates containing about 1,200 colonies per plate. The colonies of each plate were removed *en masse* and cultivated on 300 ml of Terrific Broth plus 12.5 mg/mL chloramphenicol. Clones with stable inserts can be grown overnight with shaking at 37 ºC in the presence of 1X arabinose Induction Solution (supplied in the Kit). Each pool of fosmids DNA was purified using the Large Construct Kit 10 (Quiagen, Germany). The purified DNAs from each pool of fosmids were individually sequenced by Illumina technology.

1.2.3 Preparation of fosmid libraries for paired-end sequencing

Paired-end sequencing of the fosmid library was performed using the NxSeq 40 kb Mate-Pair Cloning Kit (Lucigen Corporation, USA). Twenty five of the purified fosmids pools obtained above were pooled together in a single large fosmids pool (about 40,000 clones). The large fosmids pool was digested to completion with the restriction enzyme *Cvi*QI that recognizes 4-bp sites but do not cleave the vector backbone and thus, only the termini of the fosmids inserts remain attached to the vector. The open vector containing the termini of the fosmids inserts was purified by gel-extraction (DNA band of about 8-9 kb) and recircularized by DNA ligation. The re-ligated vector pool was amplified with Illumina specific primers for sequence analysis. The expected result is a smear of

DNA fragments ranging from 0-1,000 bp. For Illumina sequencing the DNA fragments were size-selected (around 500 bp) by gel-extraction. Three different large fosmids pools (i.e., about 120,000 fosmids in total) were used for paired-end sequencing.

1.2.4 Construction of a BAC library

A BAC library was prepared using the CopyRight v2.0 BAC Cloning kit (Lucigen Corporation, USA). The library was constructed on the *Bam*HI pre-cut and dephosphorylated pSMART BAC vector (Lucigen Corporation, USA). The lynx *Bam*HI DNA fragments (100 – 150 Kb) were prepared by pulse field gel electrophoresis (PFGE, CHEF chamber, BioRad, USA) according to the method described by Zhang HB, Scheuring CF, Zhang MP, Zhang Y, Wu CC, Dong JJ and Li YN [3] using the lynx fibroblast cultured cells as source of genomic DNA. After ligation of the purified *Bam*HI large DNA fragments and the vector, BAC-optimized Replicator v2.0 electrocompetent cells supplied in the cloning kit were used to transform the BAC ligation mixture. About 2,500 BAC clones were obtained by micro-liter of BAC ligation mixture per transformation. At the moment of writing the library contains about 15,000 lynx BAC clones.

1.3 Sequencing

1.3.1 Whole Genome Sequencing

Whole genome sequencing was performed using the Illumina Genome Analyzer IIx and HiSeq2000 sequencing instruments. The standard Illumina protocol with minor modifications was followed for the creation of short-insert paired-end libraries (Illumina Inc., Cat. # PE-930-1001). In brief, 2.0 µg of genomic DNA was sheared on a Covaris™ E220 in order to reach the fragment size of ~500 bp. The fragmented DNA was end-repaired, adenylated and ligated to Illumina specific paired-end adaptors. To obtain a library of very precise insert size (500bp) with a size deviation of +/- 25 bp, the DNA with adaptor-modified ends was size selected and purified using the E-gel agarose electrophoresis system

(Invitrogen). After the size selection the library was PCR amplified using 10 PCR cycles.

Each library was run in one lane of a GAIIx flowcell in a paired end mode of 2x114 bp or on a HiSeq2000 flowcell v1.5 in 2x101 bp read length run, both, according to standard Illumina operation procedures. Primary data analysis was carried out with the standard Illumina pipeline.

Mate-pair (MP) libraries with an average 4.5kb fragment length (estimated at 4.1kb mapping distance on final assembly) were constructed at the Centre for Genomic Regulation. Modifications to the standard Illumina protocol included incorporation of a biotinylated linker sequence normally used for 454 mate pair library construction. Illumina specific paired-end adaptors were ligated and the library was then amplified. Each library was run in one lane of a HiSeq2000 flowcell v1.5 in 2x101 bp read length run, according to standard Illumina operation procedures. Primary data analysis was carried out with the standard Illumina pipeline. Post-processing of sequence reads involved trimming of the linker sequence. Only pairs for which at least one mate was trimmed (i.e. contained the linker and was thus a true mate-pair and not paired-end contamination) were kept for scaffolding. In addition, 454 Life Sciences/Roche kindly contributed four 5.2 kb 454 paired-end libraries sequenced in separate GS FLX Titanium runs. Empirical size distributions of the different fragment libraries used are depicted in Figure S1.

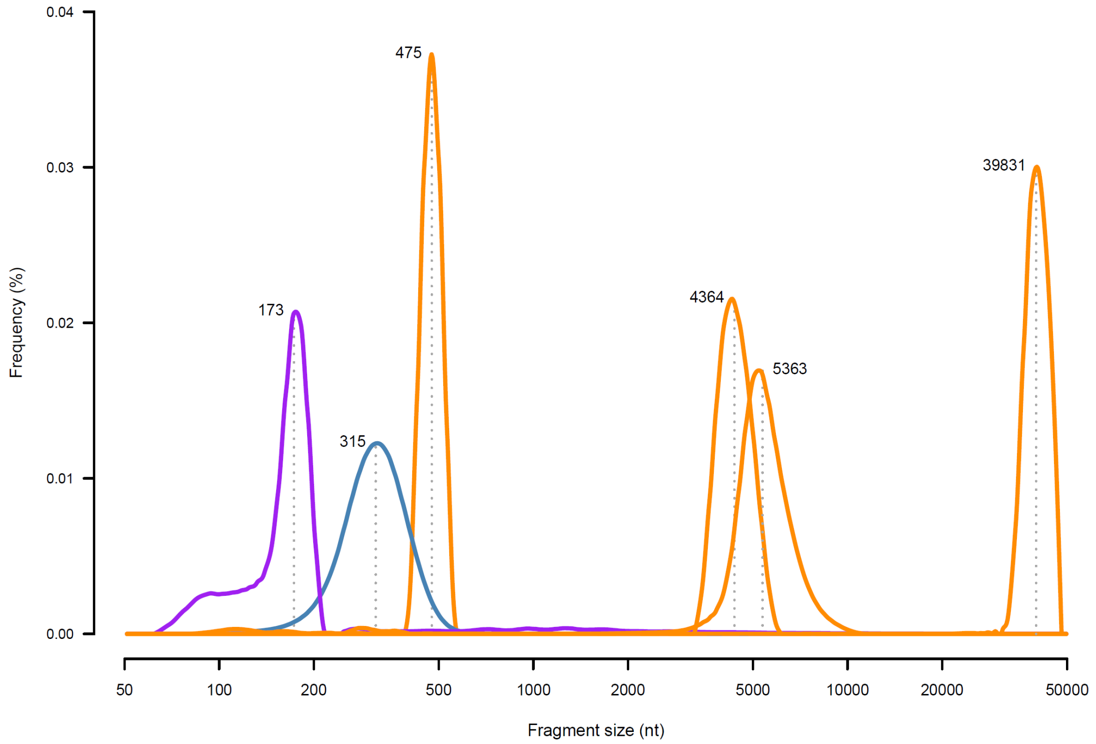

Figure S1. Library size distribution. WGS sequencing (yellow), fosmid pool sequencing (blue) and RNA-seq (purple) library fragments sizes (estimated by mapping distance) are plotted with the x-axis in log-scale.

1.3.2 mRNA sequencing

Total RNA isolated from different organs of *L. pardinus* was sequenced on the GAIIx sequencing system (Illumina, Inc).

The libraries were prepared using the mRNA-Seq sample preparation kit (Illumina Inc., Cat. # RS-100-0801) according to manufacturer's protocol with minor modifications. Briefly, 3 µg of total RNA was used for poly-A based mRNA enrichment selection using oligo-dT magnetic beads followed by fragmentation by divalent cations at elevated temperature. First strand cDNA synthesis by random hexamers and reverse transcriptase was followed by the second strand cDNA synthesis performed using RNAseH and DNA Pol I. Double stranded cDNA was end repaired, 3´adenylated and the 3´-"T" nucleotide at the Illumina adaptor was used for the adaptor ligation. The ligation products were size selected and purified using the E-gel agarose electrophoresis system (Invitrogen) targeting

the library size in the range of ~300 bp that will result in an insert size of about 200 bp. Following the gel purification, the adapter ligated cDNA was amplified with 15 cycles of PCR.

Each library was sequenced in paired end mode, 2x76 bp, in one lane of a Genome AnalyzerIIx sequencer following the manufacturer's protocol. Images from the instrument were processed using the manufacturer's software to generate FASTQ sequence files.

1.3.3 Fosmid Ends Sequencing

The CopyRight pNGS Fosmid Clonning kit from Lucigen, which contains Illumina sequencing primers flanking the insert, was used to construct a library of ~160,000 clones (also used above, for fosmid pool sequencing) which was then split into 90 pools. Aliquots of these pools were digested with a 4-cutter restriction enzyme (*Cvi*QI) that does not cut in the vector and then self-ligated and used as a PCR template to generate the end fragments using the SL1-SR4 primer pairs. This PCR gives fragments between 200 and 1000 bp. The PCR product was purified with the Ilustra TM GFXTM PCR DNA and Gel Band purification kit. The final fosmid end library was run on HiSeq2000 paired end mode, 105+7+101 bp, in one sequencing lane following Illumina instructions for the custom recipe with 4 initial dark cycles in order to overcome possible sequencing errors due to the presence of leftover of the restriction site situated after the Illumina sequencing primer position. Primary data analysis was carried out with the standard Illumina pipeline.

1.3.4 Fosmid Pool Sequencing

Illumina sequencing libraries were prepared from 90 fosmid clone pools. Each pool contained approximately 1200 fosmid clones. Sample preparation for each fosmid pool was performed according to Illumina TruSeq DNA sample preparation guide (Illumina Inc., Catalog # PE-940-2001, Rev. C) with some modifications. In brief, 2.0 μg of genomic DNA was sheared on a Covaris™ E220 instrument. The fragment size (300-450 bp) and quantity were confirmed with the Agilent 2100 Bioanalyzer 7500 chip. The fragmented DNA was size selected with AMPureXP beads and PEG concentration. The size selected DNA was end-

repaired, adenylated and ligated to Illumina indexed paired-end adaptors. The final libraries were sequenced multiplexed by 8 in one sequencing lane on an Illumina HiSeq2000 instrument with paired end run of 2x101 bp following the manufacturer's protocol. Primary data analysis was carried out with the standard Illumina pipeline.

1.4 Whole genome resequencing data

We re-sequenced 10 Iberian lynx (*Lynx pardinus*) individuals, all of them males from the extant populations in Doñaña (DON, n=4) and Andújar, Sierra Morena (SMO, n=6) (Section 1) (Table S2). For each Iberian individual 2-3 billion pair-end reads of length 100 bp and Phred Quality score above 30 were generated using the GAIIx Illumina platform, yielding average sequencing depths ranging from 24.3X to 28.4X (assuming a genome size of 2.86 Gbp, as estimated for *Lynx lynx* [4]). Additionally, the reads of the individual providing the reference genome (Candiles), which was sequenced with much higher depth (~90X), were subsampled to a total depth similar to the re-sequenced Iberian individuals to avoid biases in variant detection and genotype calling (Section 18). Finally, a single Eurasian lynx was sequenced to 64x and used for variant detection and to generate a consensus genome sequence for the species for phylogenomics (Section 13) and interspecific divergence analyses (Section 9).

Table S2. Samples resequenced for variant calling and population genomic analyses

Sample ID	Name	Population /Species	Number of reads	Read length (bp)	Sequence Coverage[2]
0/A001	Candiles*	SMO	695,810,114	114	27.78
1/A002	Beta	SMO	703,607,624	100	24.64
2/A003	Gazpacho	DON	757,217,440	100	26.51
3/A005	Arcex	SMO	748,427,854	100	26.21
4/A006	Daman2	SMO	811,608,424	100	28.42
5/A007	Almoradoux	DON	713,466,052	100	24.98
6/A008	Fran	SMO	748,151,854	100	26.20
7/A009	Pavon	DON	746,529,288	100	26.14
8/A010	Borja	DON	708,414,840	100	24.81
9/B991	Jub	SMO	753,388,788	100	26.38
10/B992	Jeme	SMO	809,014,178	100	28.33
Llynx	Ambar	Eurasian	1,796,761,054	100	64.17

* Subsampled from PE reads obtained for the reference genome assembly

2 Genome Assembly

2.1 Whole genome assembly

2.1.1 Sequence data and strategy

Approximately 160,000 fosmid clones were divided into 90 pools – each containing an average of 1200 clones – that were shotgun sequenced and assembled independently. The resulting contigs were then merged to obtain an assembly representing approximately 67% of the genome. The remaining portion of the genome was assembled using whole genome shotgun (WGS) PE data (Table S3). Both partial assemblies were combined by scaffolding with WGS PE and MP data, followed by an extra scaffolding step using RNA-seq and fosmid end data (Figure S2).

Table S3. Sequencing data used.

Type	Platform	Fragment size (nt)	Read Length (nt)	Yield (Gb)	Coverage (x)
Illumina WGS PE	GAIIx	464 bp	2 x 114	149	52.1
Illumina WGS PE	GAIIx	490 bp	2 x 114	146	51.0
Illumina WGS MP	HiSeq2000	4.1 kb	2 x 101	50	17.5
454 WGS MP	454 Titanium	5.2 kb	1 x ~330 (2 x ~167)	1.42	0.5
Fosmid Ends	HiSeq2000	39.7 kb	2 x 101	22	1000x depth (1.6x physical)
Illumina fosmid pool PE (mean per pool)	HiSeq2000	301 ± 9 bp	2 x 101	5.0 ± 0.97	90x depth (1.6x physical)

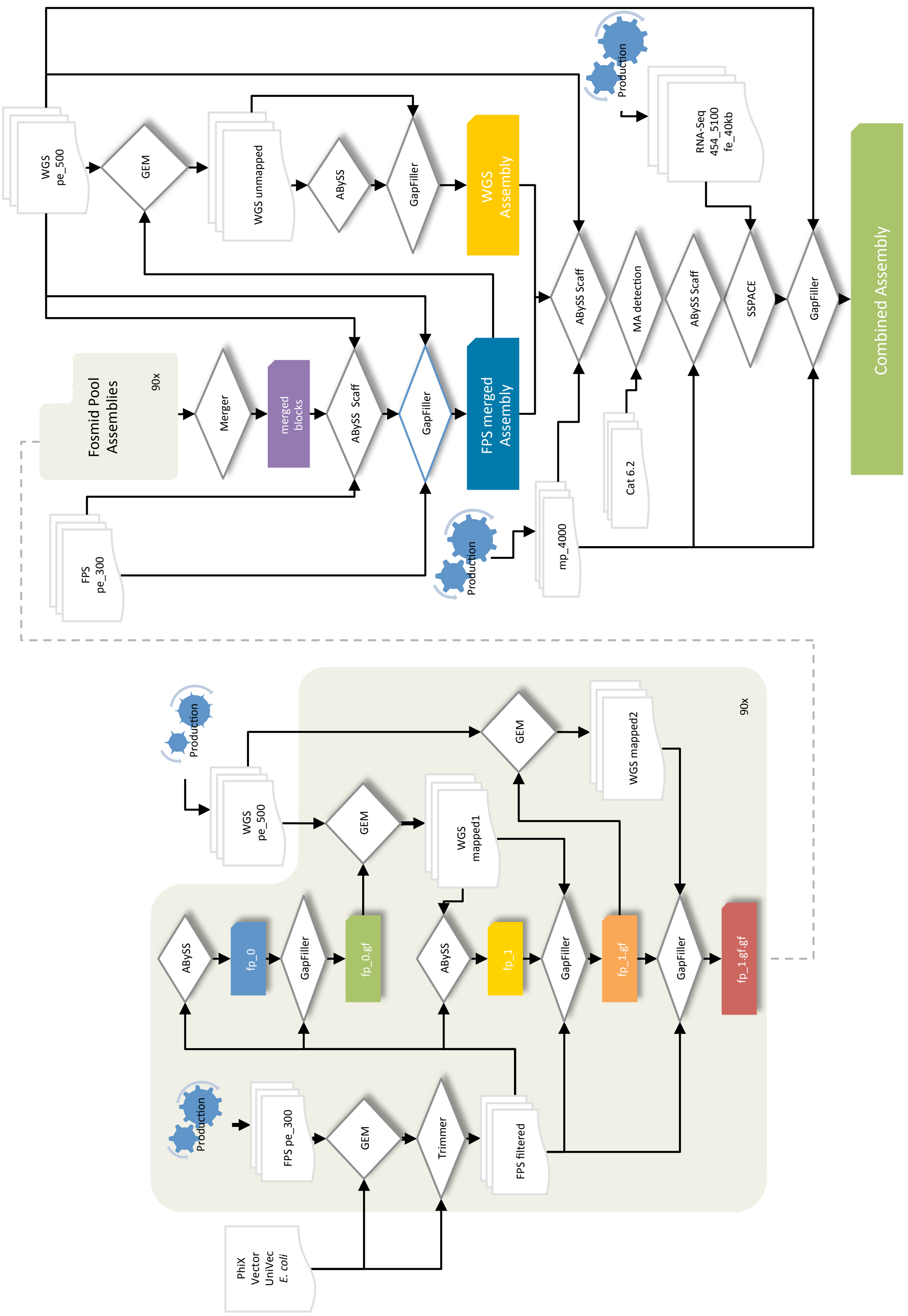

Figure S2. Genome assembly flowchart. Fosmid pool assemblies (left) were performed for each of 90 pools.

2.1.2 Fosmid pool assembly

Reads from each pool were initially assembled using ABySS [5] into scaffolds using –s=200, n=6, k=64, l=36, and q=10 as parameters. The resulting assemblies were then gap filled using GapFiller [6] with the parameters m=36, o=2, r=0.7, n=10, d=100, t=15, g=0, and i=3. The average contig N50 was 10.5 kb and the scaffold N50 26.9 kb. To overcome the short insert size of the paired-end libraries used, WGS PE reads (fragment size ~ 500bp) were mapped to each pool assembly using GEM [7] requiring both ends to map uniquely with up to 4% mismatches. For each pool, the mapped WGS PE reads were used along with the pool reads to generate a second assembly. In a second mapping round, WGS PE reads remapped to each pool assembly, this time requiring only one of the two ends to uniquely map. These additional read pairs were then used to close remaining gaps using GapFiller [6]. This enrichment procedure resulted in a significant increase in both contig and scaffold length (to an average of 22 kb and 33.5 kb N50, respectively) with respect to the primary assemblies. Contiguity statistics are given in Figure S3 and S4.

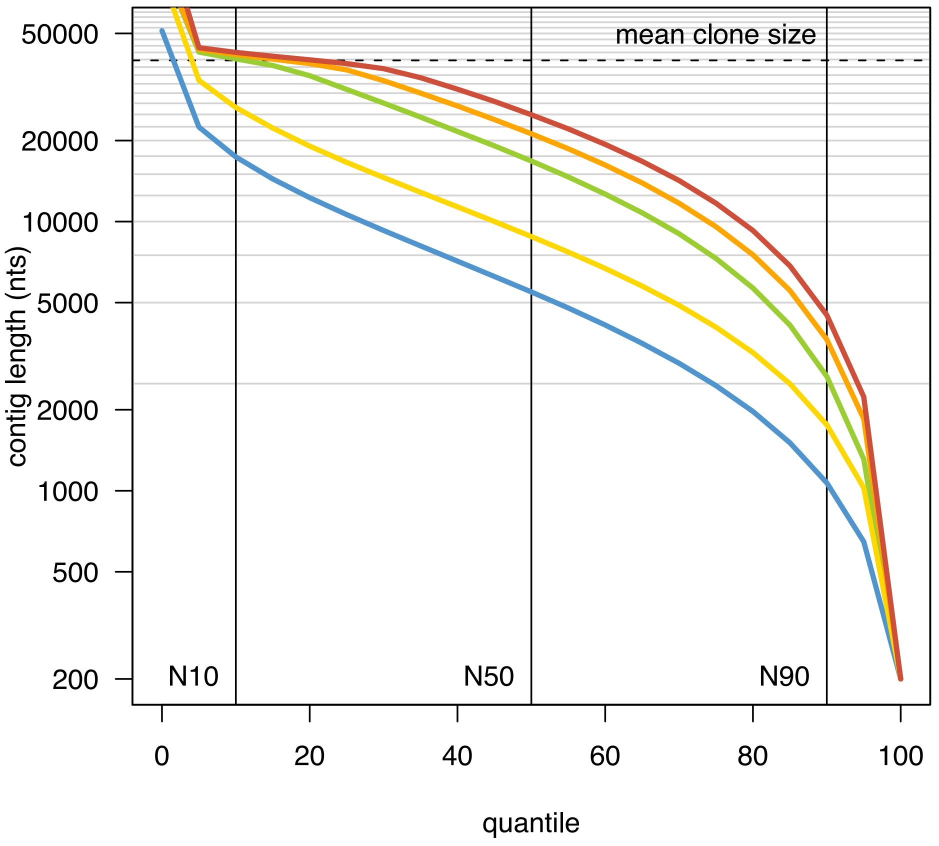

Figure S3. Contiguity statistics for the five main fosmid pool assembly steps. The plot represents the spectrum of N statistics with the vertical lines showing the N10, N50 and N90 metrics for all 90 assemblies pooled together. The base assembly that was generated using FPS PE data only is shown in blue and green (gapfilled). The WGS enriched assembly is shown in yellow, orange (first gapfilling step with conservative double-end mapped WGS PE reads), and in red (second gapfilling step including standard anchor-gap mappings of WGS PE data).

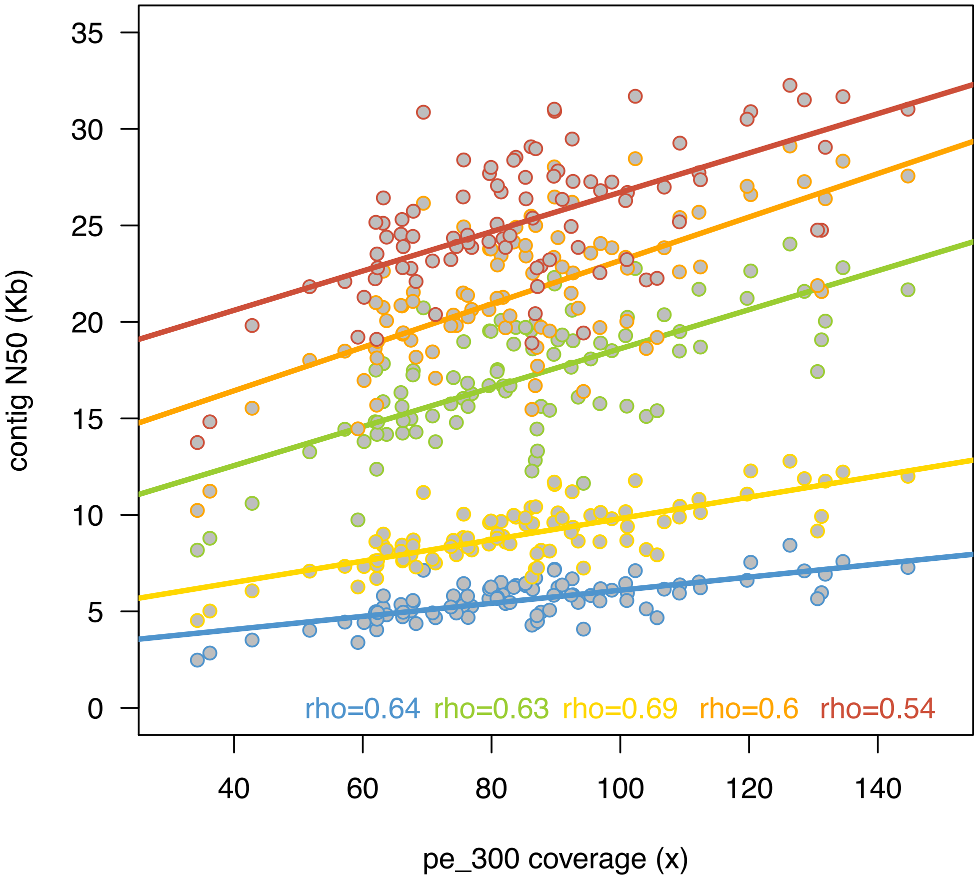

Figure S4. Contiguity (N50) with respect to estimated coverage. The N50 is plotted for each of the 90 fosmid pool assemblies according to the stage of assembly using the color codes given in Figure S3.

2.1.3 Fosmid pool merging

Contigs from all 90 pools were merged using in-house software based on an Overlap-Layout-Consensus strategy that allows for indel errors or differences between haplotypes and where path ambiguities are solved by using pairing information from PE-data. This resulted in a 1600 Mb assembly with a contig N50 of 38.2kb. The length of this assembly, at this stage, is in agreement with the ~70% real genome coverage expected from 108,000 clones for this genome size. Moreover, about 66% of the WGS reads mapped to this assembly, suggesting that haplotypes were smoothly merged and that repeats have not been extensively collapsed.

2.1.4 WGS genome assembly

To assemble the remaining portion of the genome, we selected WGS-PE read pairs that did not map to the fosmid pool assembly. These were assembled using ABySS followed by a gap closure step using GapFiller. Though some classes of repeats may be underrepresented in read set used (repeats already represented in the fosmid pool assembly), the resulting assembly was of approximately 800 Mb in length, with roughly 33% of the WGS PE read data mapping to it.

2.1.5 FPS-WGS joining by scaffolding and gap filling

Contigs from both assemblies were then scaffolded together using all the WGS PE data and the 4kb mate-pair library and gaps were filled using GapFiller, which resulted in an assembly with a contig N50 of 49.4 kb and scaffold N50 of 266 kb.

2.1.6 Homology-based coarse misassembly detection

To detect and correct potential misassemblies at this stage, all scaffolds were mapped against the cat genome (*Felis_catus* v6.2) using BLAT [8]. Out of the resulting alignment blocks, a set of non-redundant best-hits was selected and assembled into syntenic chains by a dynamic programming algorithm designed to maximize the total length of aligned bases within each chain, and synteny breakpoints were determined. Though breaks in synteny may result from real structural rearrangements occurring since the cat-Iberian lynx split, rather than from misassemblies in the Iberian lynx assembly, the assembly was fragmented at these locations and re-scaffolded using the 4kb mate-pair library, with the rationale that true structural rearrangements would be rescued in this later step. The resulting assembly had a scaffold N50 of 621Kb, suggesting that several misassemblies had been corrected, allowing for considerably more consistent, and therefore longer, scaffolds. The aforementioned procedure was also used to produce Iberian lynx-tiger alignment chains. Both chains are available for visualization as tracks in the Iberian lynx genome browser (http://denovo.cnag.cat/genomes/iberian_lynx).

2.1.7 RNA-seq scaffolding

In order to obtain a more connected assembly, in particular over genic regions, we further scaffolded our assembly using the RNA-seq data that had been sampled from 11 different tissues (described above). RNA-seq read pairs have the potential to link very distant genome locations given that they can bridge over splice junctions of introns, which can span up to hundreds of kilobases. This suggests their applicability as pseudo-long-insert libraries. The drawback is that, for a given pair, it is impossible to estimate *a priori* its “genomic” insert size (in this case the distance between ends in the genome). For this, we have used the mapping distance in a closely related genome as proxy for the pair distance in the Iberian lynx. Accordingly, RNA-seq pairs were mapped using GEM to the domestic cat genome (*Felis catus* v6.2) using iterative trimming, and the distances between consistently paired mappings were first compared to the ones obtained against the Iberian lynx assembly (Figure S5). The high correlation values observed (rho=0.98, N=5M, Spearman Rank correlation) suggest that RNA-seq pair distances in cat can be used for scaffolding with a fidelity comparable to the normally used mean or median MP library insert size. Taking the aforementioned into account, we have selected RNA-seq pairs spanning between 2kb and 150kb in the cat and binned them according to their distance. Each bin was then used as an independent scaffolding library (see Figure S6 for details). As a result the scaffold N50 increased to 897 kb and gene space completeness increased from 90% to 93% according to the CEGMA pipeline [9].

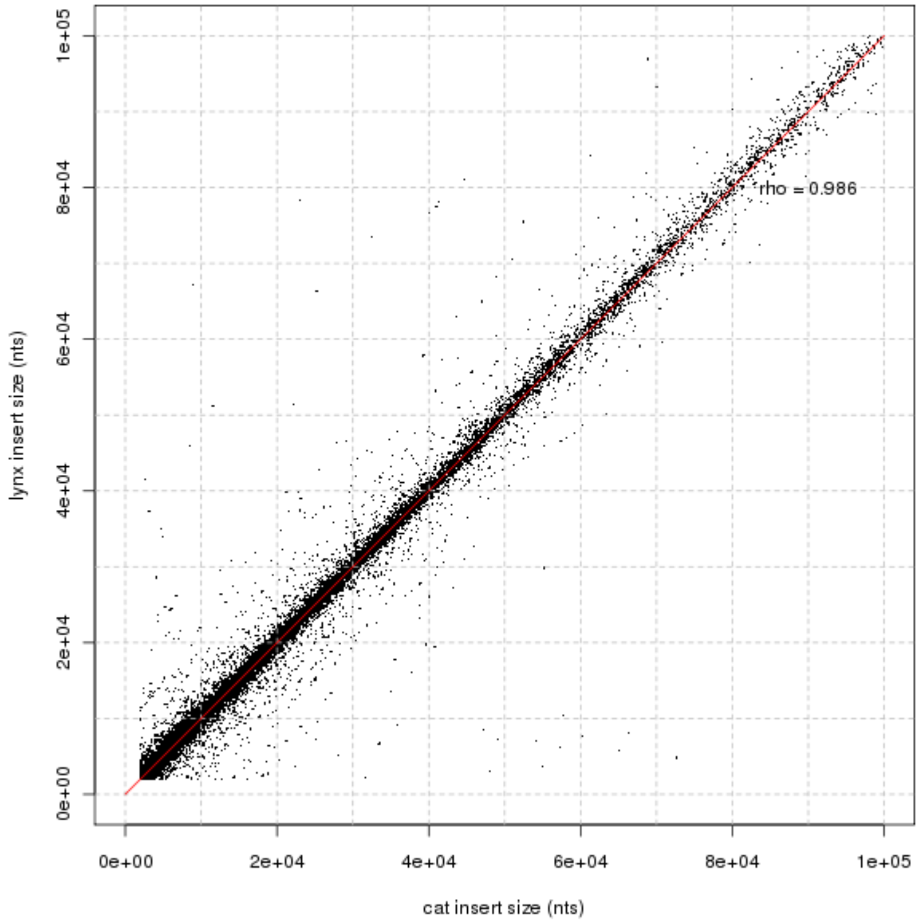

Figure S5. Correlation between RNA-seq read pair mapping distances on the genomes of cat vs. Iberian lynx.

2.1.8 Fosmid-end scaffolding

As a final scaffolding step, we have used the ends from all fosmid clones (~160000) and the 5.2kb insert 454 mate-pairs using the SSPACE Basic 2.0 scaffolder. The final contiguity achieved was 68 kb contig N50 and 1.52 Mb scaffold N50, and the CEGMA pipeline found 95% complete and 98% at least partial core orthologous genes (COGs; see below).

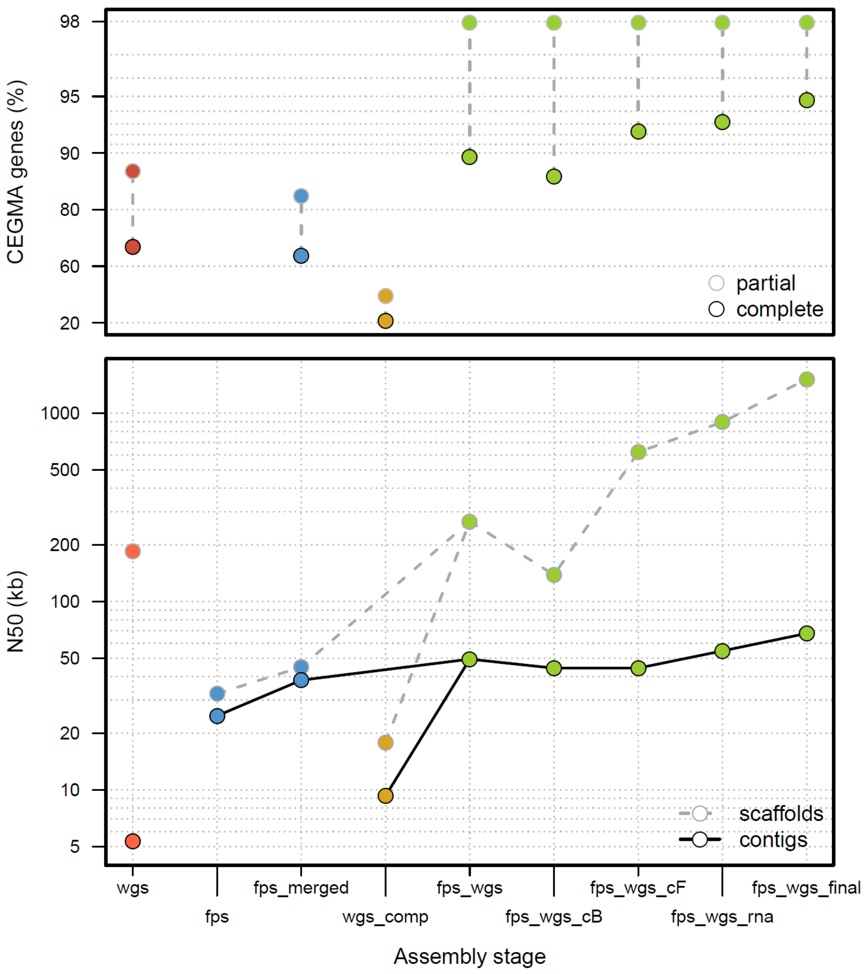

Figure S6. Contiguity and gene space completeness for major stages of assembly. CEGMA results are shown in the top panel. Contig N50 (solid line) and scaffold N50 (dotted line) are shown in the bottom panel. For reference the whole genome shotgun pilot, "wgs," is shown. Other assemblies are as follows: the average fosmid pool assemblies (fps), the merged pools (fps_merged), the assembly of WGS reads not mapping to fps_merged (wgs_comp), the scaffolded assembly including fps_merged and wgs_comp (gps_wgs), the assembly segmented at colinearity breaks in respect to the cat genome (gfs_wgs_cB), the rescaffolded or "fixed" assembly (fps_wgs_cF), the RNA-seq scaffolded assembly (fps_wgs_rna) and the final assembly lp23 (fps_wgs_final).

2.1.9 Contamination screening

Despite the fact that all read libraries had been decontaminated prior to the assembly, small traces of fosmid vector and E. coli sequence were detected by BLAST and removed from the assembly.

2.1.10 Polishing

In order to correct potential erroneous base calls and/or to have represented in the final consensus the most frequent allele, WGS PE reads were mapped to the final assembly using GEM and genotype calls were performed using SNAPE [10]. Positions with a confident genotype call were changed to the highest frequency allele if different from the reference. This final assembly version is referred to as lp23.

2.2 Evaluation of the gene space completeness

Even though sequence coverage and the N50 are useful for characterizing a genome in terms of "base pairs", it doesn't always describe the state of the gene space or whether we will be able to easily and accurately detect and identify genes in the genomic sequence. The CEGMA pipeline has been used to determine the state of the gene space (*i.e.* another indicator of genome completeness) of several species in the last few years [11]. This pipeline looks at a set of "core" orthologous genes (COGs) that were deemed to be highly conserved and exist in low-copy numbers in the large majority of higher eukaryotes. The latest COG set of genes includes 248 sets of "COG" proteins from six different species (including 248 *H. sapiens* proteins). Parra *et al* estimated that most (nearly) complete assemblies (using WGS technology) contained 90% of the 248 COG proteins.

In the case of the *L. pardinus* lp23 assembly 94.76% of the conserved COGs were fully mapped to the assembly (235 out of 248) by the CEGMA pipeline. This number in itself is an indicator that the assembly, in terms of its gene space, can be considered as being of very high quality for a draft genome. Furthermore, 97.98 % of the COG proteins could be mapped at least partially to the assembly (243 out of 248). The percent of partial matches to the COGs (3.2%) suggests that even though the assembly in itself is very complete a number of genes (with an average length in lynx of approximately 36 Kbases) are "split" into more than one of the several smaller-size scaffolds, as only 3,834 scaffolds (out of the 41,700 that make up the assembly) are longer than the estimated average length of a gene in *L. pardinus*.

2.3 Comparison with other Felid assemblies

In order to evaluate the quality of the lp23 assembly (publicly named LYPA1.0) we have compared several statistics between all the felid assemblies existing at the time (Table S4). Although in terms of scaffold contiguity our assembly is the lowest, it shows the highest contig N50 and largest scaffold is similar to that of the recently published cheetah genome [12]. This implies that we have produced highly contiguous blocks with the trade-off of being conservative in establishing connections among them (scaffolding). The existence of large contigs is reflected in the gene completeness, as estimated with CEGMA, being closer to FelCat6.0 the highest-quality assembly in this comparison.

Table S4. Assembly statistics of felid genomes.

Species	Assembly	Length[1]	Largest scaffold[1]	Scaffold N50[1]	Largest contig[1]	Contig N50[1]	Total No. Gaps	Total gap length[1]	GC content (%)	CEGMA complete genes (%)	CEGMA partial genes (%)
Lynx pardinus	LYPA1.0[2]	2,413,209,059	13,188,378	1,519,745	545,385	67,711	74,938	52,002,211	0.415	95.16	97.98
Acinonyx jubatus	aciJub1	2,372,553,907	13,046,067	3,122,036	304,265	28,271	183,260	42,058,837	0.413	94.35	98.79
Panthera tigris	PanTig1.0	2,391,082,183	41,607,841	8,860,407	287,365	30,032	155,553	58,232,500	0.414	93.15	98.79
Felis catus	Felis_catus-6.2[3]	2,455,541,136	*239,302,903*	*148,491,654*	318,754	20,621	197,583	91,244,929	0.401	95.97	99.6

[1] Units of length are in base pairs

[2] Public release of the lp23 assembly

[3] The chromosomal version of this assembly was created by aligning marker sequences associated with a radiation hybrid (RH) map (Davis et al. 2009) to the assembled genome sequence using Cross_match (P. Green, unpublished).

2.4 Mitochondrial genome assembly

The alignment of the mitochondrial genome was assisted by *de novo* assemblies generated from 454 reads obtained from long-PCR products. Control region RS2 and RS3 repeats, which were not properly assembled de novo, were obtained from Sanger Iberian lynx PCR product sequences (RS2) or Eurasian lynx published sequences (RS3) and manually inserted to generate a whole mitochondrial genome to be used as reference for a mapping based assembly [13]. Iberian and Eurasian lynx mitochondrial genomes were *de novo* assembled based on Illumina reads mapped to this reconstructed sequence. This first assembly was used as base for a second round of mapping and assembly. The final sequences were gap-filled and manually edited to match coordinates and orientations to previously reported felid sequences.

2.5 Y chromosome assembly and annotation

Iberian lynx Y chromosome scaffolds were identified by first aligning all assembly scaffolds to the female-derived domestic cat genome assembly (felCat 5.0). Scaffolds that failed to produce a significant BLAST hit were placed in a separate file and further evaluated for Y chromosome content by querying these scaffolds via BLAST, using 1) the domestic cat Y chromosome single-copy and partial multicopy assembly from Li et al. (2013)[14], and 2) single copy and multicopy Y chromosome cDNA sequences from Murphy et al. (2006) [15] and Wilkerson et al. (2008) [16]. We used a BLAST score cuttoff of 1e-20. In total, we identified 927,793-bp of single copy Y chromosome sequence, and 419,877 of ampliconic/multicopy gene sequence. The latter number is an underestimate due to probable collapse of multicopy gene families into single scaffolds, or smaller contigs. We identified orthologues of nearly every known domestic cat Y chromosome transcript within these scaffolds (Table S5).

As a second approach we queried the *L. pardinus* testis *de novo* transcriptome assembly with a published set of domestic cat Y chromosome cDNA sequences. We were able to confirm multiple testis-expressed transcript isoforms per gene (Table S5) and validate our initial BLAST-based gene search results using the

assembled scaffolds. We found no evidence of stop codons or disrupted open reading frames in the *L. pardinus* Y chromosome transcripts. This suggests that the functional single-copy transcript content is conserved between the *Felis* and *Lynx* genera.

Table S5. Identified Y chromosome scaffolds, gene content and expression potential

**Single copy genes/ low copy gene families**	**BLAST result**	***Lynx pardinus* scaffolds**	**Testis expressed**
UTY	+	scaffold00015426, scaffold00082875, scaffold00058506, scaffold00058504, scaffold00058500, scaffold00065408	+
DDX3Y	+	scaffold00015426	+
USP9Y	+	scaffold00081870, scaffold00060902, scaffold00028606, scaffold00015427	+
AMELY	+	scaffold00050315	NA*
EIF1AY	+	scaffold00027549	+
EIFS23Y	+	scaffold00028466	+
UBE1Y	+	scaffold00049695	+
SMCY	+	scaffold00038387	+
SRY	+	scaffold00052116	+
HSFY	+	scaffold00079824	+
CYORF15	+	8, mixed with *CYORF15* fusion gene	+
CYORF/EIF1AYfusion	+		+
ZFY	+	scaffold00038448, scaffold00038447	+
**Ampliconic gene families**			
CUL4BY	+	scaffold00081220, scaffold00081221, scaffold00085099, scaffold00081227, scaffold00086513, scaffold00007750, scaffold00085216, scaffold00051752, scaffold00016428, scaffold00031284, scaffold00016411	+

FLJ36031Y	+	scaffold00008144, scaffold00009667, scaffold00032702, scaffold00036788, scaffold00041249, scaffold00042544, scaffold00063415, scaffold00074578, scaffold00075763, scaffold00085806, scaffold00087907	+
TETY1	+	scaffold00019867, scaffold00019690, scaffold00041653	+
TETY2	+	scaffold00053523, scaffold00041264, scaffold00067193	low expression
TSPY	+	25 scaffolds	+
* *AMELY* only expressed in developing tooth buds			

3 Genome annotation

3.1 Protein-coding genes

3.1.1 *Ab initio* gene prediction

Ab initio gene predictions were performed on the lp23 assembly masked for repeats found with RepeatMasker [17] with *Felis catus* given as the species. Low complexity repeats were left unmasked for this purpose. Four different approaches were used and finally combined to generate consensus gene models.

Genid. Geneid is an *ab initio* gene prediction program used to find potential protein-coding genes in anonymous genomic sequences. In the context of Geneid, training basically consists of computing position weight matrices (PWMs) or Markov models of order 1 for splice sites and start codons, and deriving a model of coding DNA, which, in this case, is a Markov model of order 5. Furthermore, once a preliminary species-specific matrix is obtained it is further optimized by adjusting two internal matrix parameters: -the cutoff of the scores of the predicted exons (eWF) and the ratio of signal to coding statistics information to be used (oWF). Geneid using its *H. sapiens*/mammal-specific parameter file has

been used in the past to accurately generate gene predictions in several different mammalian genomes (for example, [18, 19]).

SGP2. SGP2 is a syntenic gene prediction tool that combines *ab initio* gene prediction (Geneid) with TBLASTX searches between two or more genome sequences to provide both sensitive and specific gene predictions, and it tends to improve Geneid's performance, especially by reducing the number of false-positives. SGP2 requires a reference genome to which the target genome (in this case *L. pardinus*) is compared. We decided to use the genome of *M. musculus* (assembly mm7) as reference to develop our *L. pardinus* parameter file for SGP2 because it seems to be at an appropriate evolutionary distance from *L. pardinus* so that it is mostly the coding regions of the genes that seem to be significantly conserved between these two genomes. Using *H. sapiens* as the reference genome generated similar, albeit slightly inferior performance (data not shown).

Obtaining of the *L. pardinus*-specific SGP2 parameter file was based on the methodology used by Parra *et al.* [20] to generate a human SGP2 parameter file using mouse homology evidence. The starting point to obtain a parameter file for SGP2 is the Geneid matrix used to predict genes in mammalian genomes. The SGP2 parameter file was then optimized using the *M. musculus* "mm7" RefSeq annotation (obtained from the UCSC "goldenpath" genome database). We optimized the SGP2 matrix by modifying not only the eWF internal parameter (as previously for the Geneid parameter file) but also two SGP2-specific internal parameters ("NO_SCORE" and "HSP_factor"). The "NO_SCORE" parameter provides a penalty for no overlap between TBLASTX-derived HSPs (High-Scoring Pairs) and Geneid *ab initio* predictions in the same region, whereas the "HSP_factor" parameter reduces the score assigned to the HSPs in order to maximize the prediction accuracy. The optimal values of "NO_SCORE" and "HSP_factor" for *L. pardinus* were found to be 0.05 and -1.25, respectively.

GlimmerHMM. We also obtained *L. pardinus* gene predictions using the program GlimmerHMM (University of Maryland; http://www.cbcb.umd.edu/software/GlimmerHMM). GlimmerHMM is an *ab initio* program that is based on a Generalized Hidden Markov Model (GHMM). This program also incorporates splice site models adapted from the GeneSplicer

program and a decision tree adapted from GlimmerM [21]. For GlimmerHMM we simply used the mammalian matrices included with the program.

Augustus. We also produced *L. pardinus* protein-coding gene annotations using the gene prediction tool Augustus. Augustus is a program that predicts genes in eukaryotic genomic sequences and is "re-trainable". The program is based on a Hidden Markov Model and integrates a number of known methods and submodels [22]. In order to predict gene sequences in *L. pardin*us using Augustus we employed the program's pre-existing mammal/*H. sapiens* parameter file.

Accuracy of the four different approaches in predicting sequences in *L. pardinus* was evaluated on an "artificial contig" of 16 Mb consisting of 498 evaluation-set concatenated gene models with 800 nucleotides of intervening sequence between each of the genes. This artificial contig was built using one of the modules of a recently developed Geneid training tool. The protein-coding gene sequences embedded into the artificial contig were generated by selecting a subset of *F. catus* RefSeq genes (with more than 2000 nucleotides) that were mapped to the *L. pardinus* lp23 assembly using the genomic mapping and alignment tool GMAP [23]. We only selected those *F. catus* genes mapping to the lynx genome with high stringency (>98% identity over >98% of length). The performance of each approach with this artificial contig is summarized in Table S6.

Table S6. Accuracy of gene prediction on a *L. pardinus* artificial contig consisting of 498 concatenated Iberian lynx test sequences with approximately 800 nucleotides of sequence between each of the gene models using the ab initio programs Geneid, Augustus and GlimmerHMM using the pre-existing mammal-specific parameter files for each of the programs. The accuracy of SGP2 (homology evidence-based prediction tool that used the *M. musculus* genome as reference) was also tested for accuracy on the same set of sequences (SN & SP: sensitivity & specificity at nucleotide level; SNe & SPe: sensitivity & specificity at exon level; SNg & SPg: sensitivity & specificity at gene level).

**Program/param**	**SN**	**SP**	**SNe**	**SPe**	**SNg**	**SPg**
**Geneid**	0.85	0.78	0.59	0.60	0.13	0.16
**SGP2 (lynx / mouse)**	0.93	0.85	0.72	0.65	0.23	0.24
**Augustus**	0.75	0.84	0.55	0.64	0.12	0.18

The Geneid, SGP2, Augustus and GlimmerHMM mammal-specific parameter files were subsequently used to predict genes on the repeat-masked lp23 assembly of the Iberian lynx genome made up of 41,700 scaffolds. The number of predicted gene models ranged from 21,973 with Augustus to 161,352 with GlimmerHMM. Geneid and SGP2 predicted intermediate gene numbers: 47,688 and 34,762 protein-coding genes, respectively.

3.1.2 Generation of Consensus Gene Models

A combination of the Program to Assemble Spliced Alignments (PASA r2012-06-25) and Evidence Modeler (EVM r2012-06-25) [24] was used to obtain consensus coding sequence (CDS) models using three main sources of evidence: aligned transcripts, aligned proteins, and gene predictions.

Transcripts for assembly with PASA (r2012-06-25) were obtained as follows: first, RNA-seq reads from 11 tissues (described above) were aligned to the final *Lynx pardinus* assembly with GEM and transcript models generated using the standard Cufflinks [25] pipeline, resulting in 132,012 transcripts, which were then added to the PASA database. In addition, 386,206 cat and dog ESTs and mRNAs present in Genbank were also added to PASA using GMAP as the alignment engine. All of the above transcript alignments were then assembled by PASA, resulting in 104,059 PASA assembled transcripts.

We aligned the complete mammalian proteomes present in Uniprot (22 Apr 2013) to the lynx genome with SPALN [26], resulting in 522,464 proteins aligned. Gene predictions were obtained as described above. Then the transcript alignments, protein alignments and the *ab initio* gene models were combined into consensus CDS models by EVM using the weights in Table S7.

Table S7. EvidenceModeler weights.

Type	Source	Weight
**ABINITIO_PREDICTION**	Augustus	2
**ABINITIO_PREDICTION**	AugustusHints	4
**ABINITIO_PREDICTION**	GlimmerHMM	1
**ABINITIO_PREDICTION**	geneid_v1.4	2
**ABINITIO_PREDICTION**	sgp2	3
**PROTEIN**	GeneWise	6
**TRANSCRIPT**	PASA	10

The consensus CDS models were then updated with UTRs and alternative exons through two rounds of PASA's annotation updates. The resulting transcripts were grouped into genes and then systematic identifiers with prefix "LYPA" were assigned to the genes, transcripts and protein products derived from them.

Support by source of evidence at the gene and exon level was determined *a posteriori* using BEDTOOLS *intersect* and *multiinter* programs.

3.2 Non-coding RNAs

3.2.1 Small non-coding RNAs (sncRNAs)

The genome of *Iberian lynx* was analyzed for detecting small structured non-coding RNAs by using the CMsearch tool from the Infernal package (version 1.1rc2) [27]. We scanned the genome looking at every RNA model stored in the Rfam database (version 11) [28]. Overlapping hits were removed by selecting the hit with the lowest E-value. Setting an E-value cut-off of 1E-06 allows detecting 21,842 non-overlapping hits. As shown below, the majority of them are microRNAs, snRNAs and snoRNAs (Figure S7).

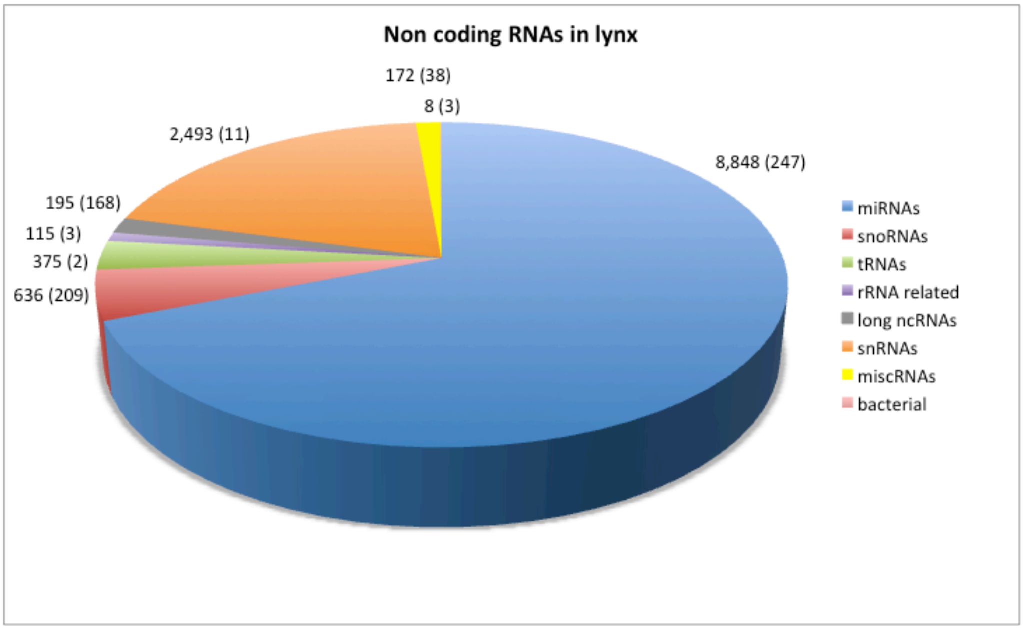

Figure S7. Number of RNAs (hits) is shown. Numbers in parenthesis are the corresponding families.

3.2.2 Long non-coding RNAs (lncRNAs)

Homology based prediction of lncRNAs

Homology based lncRNAs are predicted using the strategy reported in [29, 30]. We used genomic regions from human and mouse (gencode v17 annotation and Ensembl) coding for lncRNAs as templates and blasted them against the reference genome previously masked for interspersed repeats using RepeatMasker. The hits thus obtained were used as anchor points to guide the extraction of surrounding genomic regions. These regions were then re-aligned with the original query using exonerate in split mapping alignment mode, which allows for intron modeling between the query and the extracted genomic sequence. Hits aligned to the query but covered with more than 20% of known ancestral repeats, as estimated by RepeatMasker [17], were removed from the analysis in order to avoid the inclusion of non-related sequences. Final conservation was estimated on a T-Coffee [31] pairwise re-alignment between the query and its predicted spliced model (excluding introns).

Ab-initio prediction of lncRNAs

We used Cufflinks [32] to build transcript models on all RNA-seq samples, processed one at a time. Models containing less than 2 exons or overlapping with protein-coding gene were filtered out. We then used Cuffmerge to combine transcript models from all samples into a single set of consensus models. These were additionally filtered for ancestral repeats (less than 20% coverage).

Refinement of the lncRNA complement

We filtered out all sequences having a high GeneID [33] coding potential (GeneID likelihood ratio of 15 or higher), overlapping with GeneID predicted ORFs onto the genome, or having an ORF covering more than 25% of their length. These cut-offs were selected so as to retain the trusted predictions of non-coding genes obtained by homology. Note that the final set consisted of transcripts that are either expressed or conserved in at least one species (Table S8).

Table S8. Number of transcripts and gene loci obtained from homology and ab-initio predictions. (Note that gene numbers do not add up to the figure given in the last column due to loci containing transcripts of different types).

	homology mouse	homology human	ab-initio	all
transcripts	157	1889	2284	4330
genes (expressed)	143 (65)	1479 (845)	1608 (1608)	3179 (2475)

3.3 Transposable elements and repeats

We annotated transposable elements and other repeats with RepeatMasker (version open-4.0.1, using rmblastn v2.2.27 search engine and RM database v20120418; [17]) using the *Felis catus* library of repeats and the sensitive search option. Low-complexity regions were identified with DustMasker v2.2.28 [34], with default parameters. Overall abundance of different classes of repeats was very similar between cat and lynx (11.72% SINEs, 20.17% LINEs, 4.94% LTRs, and 2.71% DNA transposons in lynx), although a detailed analysis revealed that some subfamilies have been active in lynx and cat since their divergence (see Section 15).

4 Functional annotation

4.1 Protein annotation

We used the automatic functional annotation pipeline developed in our group for the annotation of the protein-coding gene set (genebuild lypa23B). The pipeline performs an inference of function that is based on the sequence similarity between our proteins of interest and other sequences annotated in different public repositories, the most extended approach when the number of sequences to annotate is large, as in this case. The pipeline is based on some of the most well-established tools routinely used by the scientific community for the functional annotation of proteins (Figure S8). Interproscan [35] allows to scan for matches against the InterPro [36] collection of protein signature databases; KEGG [37] and Reactome [38] allow to predict the participation of a certain protein in a specific reaction or pathway; and Blast2GO [39] allows to assign a

description (e.g. the protein name) and relevant annotation through sequence similarity and Gene Ontology based data mining. Using a combination of the results obtained with Blast2GO, InterPro and KEGG, lynx proteins were associated to unique or, whenever possible, multiple GO terms. Using these three sources of evidence of GO terms association, we managed to assign terms to a broader spectrum of lynx proteins. Moreover, SignalP [40] was used to predict the presence and location of signal peptide cleavage sites in our proteins of interest. Finally, in order to organize, store and facilitate the access to the entire set of annotations we have developed a MySQL (http://www.mysql.com/) relational database.

A total of **30,610** (98%) out of **31,121** proteins had some type of annotation feature derived from one of the annotation resources used in this work (InterPro, KEGG and Blast2GO). GO terms were assigned to 27,903 (89%) proteins. Additionally, we were capable of assigning a description (name) to 26,350 (85%) proteins using Blast2GO and a definition (name) to 14,686 (47%) proteins using KEGG. In total, 26,853 (86%) proteins had either a Blast2GO description or a KEGG definition.

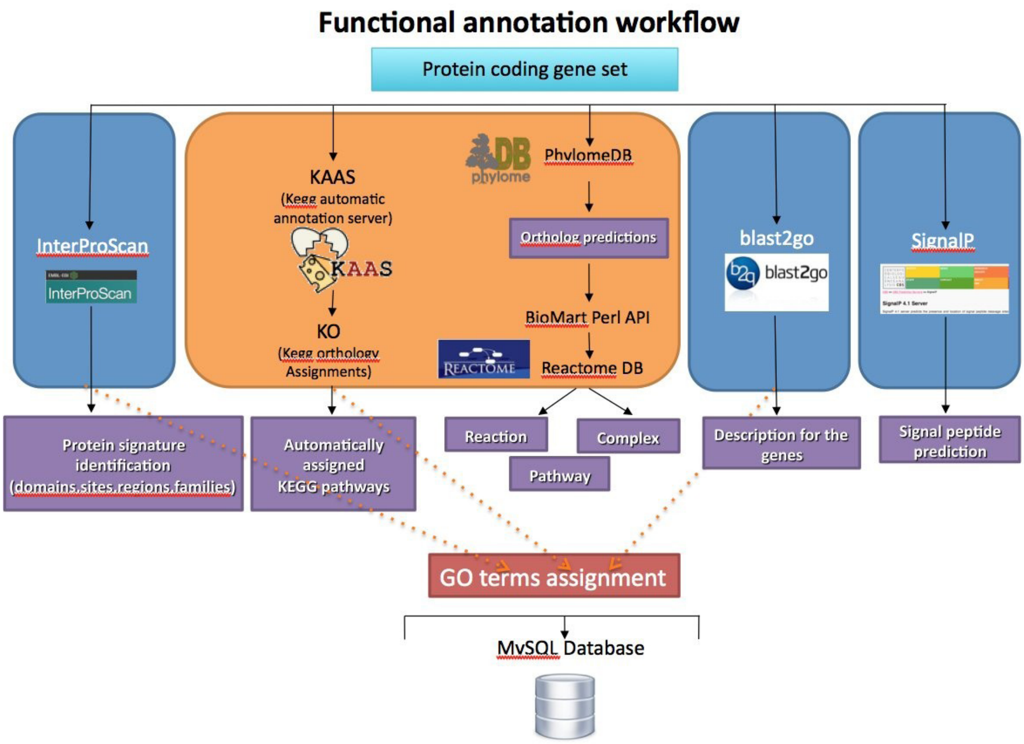

Figure S8. Functional annotation pipeline

4.1.1 Interpro-based annotation

We used InterProScan (version 5) to inspect the lynx proteins for signatures using all available InterPro databases. In total, 29,600 (95%) proteins have some type of protein signatures derived from one of the InterPro member databases. More specifically, 29,283 proteins (94%) were annotated with at least one signature coming from one of the most important InterPro databases for functional annotation (i.e. PANTHER, Pfam, TIGRFAM, HAMAP, SUPERFAMILY). Table S9 displays the number of proteins containing a signature belonging to each specific InterPro member database.

Table S9. Number of protein signatures identified by InterProScan for each of the InterPro member databases

InterPro member database	Number of proteins
PANTHER	28,509
Pfam	25,722
SUPERFAMILY	22,167
Gene3D	20,645
ProSiteProfiles	15,474
SMART	13,940
ProSitePatterns	9,054
Coils	7,482
PRINTS	7,501
PIRSF	1,879
TIGRFAM	1,559
Hamap	255

4.1.2 Blast2GO-based annotation

Blast2GO was used by running a BLAST search against the NCBI non-redundant (NR) collection of protein sequences (release 2013-04) and mapping the obtained hits to existing annotation associations. This analysis was run with the local p2gpipe version 2.5.0 with databases go_201309 (September 2013) and it assigned a description to 26,350 (85%) of the initial protein set. This description is produced by Blast2GO using the text mining functionality embedded in the Blast2GO suite that is capable of generating a consensus description extracted from all the descriptions in the Blast hits. Furthermore, Blast2GO was also used to assign GO terms to our proteins (see section 4.1.6).

4.1.3 KEGG-based annotation

Each aminoacidic sequence was mapped to KEGG orthology (KO) groups using the freely accessible KEGG Automatic Annotation Server (KAAS) [41]. For this mapping KAAS uses a bi-directional best hit (BBH) method that performs BLAST searches in both forward and reverse directions against a representative gene set from 29 different species, including *Canis lupus familiaris and Ailuropoda melanoleuca.* KO identifiers were then used to retrieve the KEGG relevant functional annotation, such as metabolic pathways and external database references, using the KEGG REST-based API service. In total 14,751 (47.4%) proteins were assigned to 7,479 different KO groups. Additionally, 14,686 (47%) proteins were annotated with a definition (name).

4.1.4 Reactome-based annotation

Reactome [www.reactome.org] is a repository of manually curated biological pathways for several organisms. Our Reactome-based annotation consisted on the derivation by an orthology-based process of the reactions and pathways in which our proteins are involved, based on the annotations that are already present in Reactome for a certain organism for which there is an ortholog to our protein. This orthology-based derivation was performed using the orthology predictions generated by the Comparative Genomics group at the CRG using PhylomeDB [42].

In order to derive the reaction annotations we used the 178,345 one-2-one orthologs identified in 13 different species that corresponded to 16,315 lynx proteins. The species used in this analysis were: *Ailuropoda melanoleuca, Bos taurus, Canis lupus familiaris, Equus ferus caballus, Felis catus, Homo sapiens, Loxodonta africana, Lynx lynx, Lynx pardinus, Monodelphis domestica, Mus musculus, Myotis lucifugus, Panthera tigris altaica, Rattus norvegicus, Sus scrofa*. Uniprot and Ensembl IDs from orthologs were used via the Reactome URL/XML query system to scan the Reactome DB (version 46, September 2013) and obtain the reactions, complexes and pathways in which a certain protein is involved. A total of 5,161 lynx proteins obtained some Reactome annotation, 5041 for Reaction (22,561 total annotations), 5041 for Pathway (30629 annotations) and 4,187 for Complex (75,835 annotations).

4.1.5 Signal peptide annotation

The presence and location of the signal peptide cleavage sites was predicted using the software SignalP with the default parameters for eukaryotes. A total of 3,049 proteins contained signal peptides with D-score (discrimination score) > 0.450.

4.1.6 GO terms association

We have four different sources of evidence to associate GO terms to our proteins: InterPro, KEGG, Blast2GO and Reactome. Using these four resources we managed to associate at least one GO term to 27,903 (89%) proteins. Figure S9 shows the overlapping among the different GO terms sources of evidence (i.e. Interpro, KEGG, Blast2GO and Reactome). Table S10 displays the number of GO terms of each specific type obtained in this work. Figure S10 illustrates the distribution of GO terms grouped by the different functional categories.

Table S10. Number of proteins, associated with each different GO term type.

Term type	Number of proteins
Biological process	23,157
Cellular component	23,782
Molecular function	25,073
Any of the above	27,903

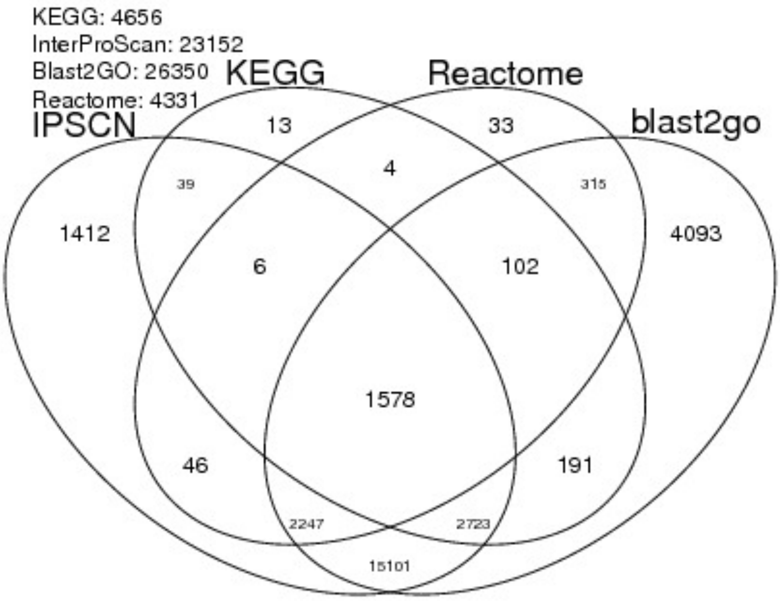

Figure S9. Number of proteins having GO terms from the four different sources of evidence used in this work (i.e. InterProScan, KEGG, Blast2GO and Reactome)

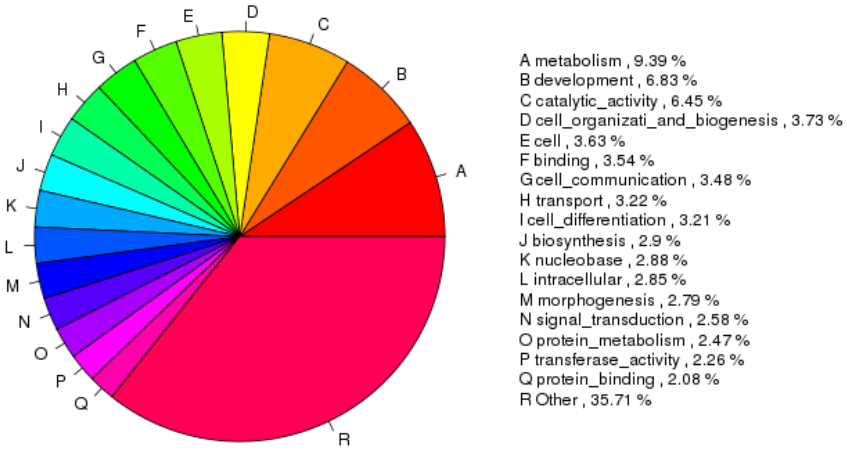

Figure S10. The GO terms mapping results into GO slims without top level categories.

4.1.7 Proteins having no annotation

A total of 511 (1.6%) out of 31,121 proteins did not have any type of functional annotation from any of the different approaches followed in this work. This percentage of proteins is not surprising and has been previously reported in other projects we have been involved in. Figure S11 illustrates the correspondence between the protein length and the number of annotated and non-annotated proteins; it is evident that the length of the non-annotated sequences tends to be smaller, this could be due to the fact that these smaller proteins could come from incomplete ORFs erroneously predicted as separate genes by the automatic annotation pipeline due to genome fragmentation.

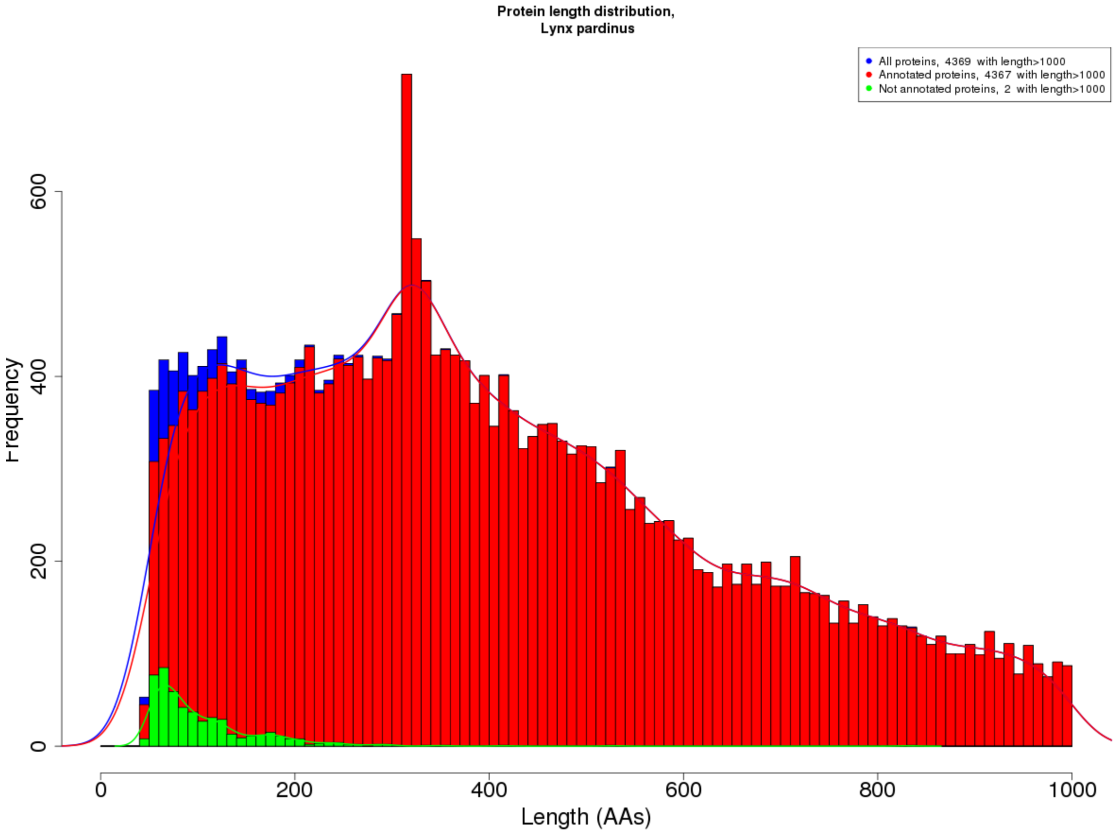

Figure S11. Number of annotated and non-annotated sequences in relation to their length. The blue color correspond to all proteins, red – annotated proteins and green to the non-annotated proteins.

4.2 Principal isoform identification

The APPRIS database (http://appris.bioinfo.cnio.es/docs/appris.html) was developed for the human genome and was based on the merged GENCODE [43] and Ensembl [44] annotations. APPRIS-Human is updated with each new release, approximately every three months [45]. Here APPRIS has been applied to the lynx genome (lp23). APPRIS for lynx is based on seven of the eight separate modules. The modules in APPRIS map a range of conserved protein features to the variants annotated for each gene. All computational methods behind each module are previously published and explained breifly below.

CORSAIR is implemented locally and uses BLAST [46] to map orthologous protein sequences from a reduced list of vertebrate species to each variant. CORSAIR counts the number of orthologs that align correctly to each alternative transcript in the BLAST searches. For each isoform the CORSAIR score approximates to the number of species with orthologues that align correctly and without gaps.

CRASH uses locally installed versions of the SignalP and TargetP programs [47] to make conservative predictions of signal peptides and mitochondrial signal sequences. The reliability of the predicted signal sequences predicted by SignalP and TargetP is based on the scores of the two programs. CRASH indicates those variants that are predicted to have reliable signal sequences.

***firestar* [48, 49]** makes reliable predictions of conserved functionally important amino acid residues in protein sequences. *firestar* is based on FireDB [50], a database of biologically relevant protein-ligand binding sites culled from the PDB [51]. *firestar* uses PSI-BLAST [46] and HHsearch [52] to align protein sequences against pre-generated sequence profiles from FireDB. The *firestar* method outputs the total number of ligand binding/catalytic residues detected for each transcript

Matador3D is a locally implemented method that employs a simple BLAST search to align PDB structures to the annotated transcripts. Alternative variants that introduce large indels into alignments between the protein isoforms and known structures are likely to have 3D structures that do not fold properly. The

Matador3D score is based on the number of exons that can be mapped to structural homologues.

SPADE uses the program Pfamscan [53] to map Pfam functional domains to the variants and identifies the number of conserved and compromised Pfam functional domains from the output. Indels in alignments between the protein sequences and functional domains are suggestive of loss of protein function. The SPADE value is the absolute numbers of Pfam domains that are detected. The numbers after the decimal indicate the numbers of partial/damaged domains.

THUMP is a trans-membrane helix prediction web service that makes conservative predictions of trans-membrane helices by analysing the output of three trans-membrane prediction methods, MemSat [54], PHOBIUS [55] and PRODIV [56]. THUMP only predicts trans-membrane helices when all three prediction methods agree. The THUMP value is the number of trans-membrane helices detected. Numbers after the decimal indicate partial/damaged trans-membrane helices.

The results of each module can be accessed via the APPRIS-lynx web site (download formats can be found at http://appris.bioinfo.cnio.es/docs/aws.html).

As part of the annotation process, APPRIS selects a single isoform as the dominant, or principal, isoform from among the isoforms annotated for each gene. The methods that make up APPRIS detect missing or damaged conserved features, or find fewer cross-species relatives, and will flag these transcripts as alternative. The principal isoform is selected by a jury of the six modules. The core of the jury system is made up of four methods (SPADE, CORSAIR, Matador3D and *firestar*) and the other methods are important where these methods cannot make a decision. Where APPRIS cannot determine the main isoform, it chooses the variant with the longest protein sequence from among those isoforms not flagged as alternative. We have compared the APPRIS principal isoforms against the annotated consensus CCDS variants for human genes [57] where the CCDS project annotates a unique transcript [45], and against the main isoform identified by proteomics experiments (internal results).

In both cases the agreement with APPRIS principal isoform is higher than 96% of the genes.

APPRIS identified the main isoform for the majority of lynx genes with multiple variants. There were 5,218 genes annotated with distinct protein sequence variants and APPRIS determined a principal isoform for 3,408 (64.9%). A total of 8,066 variants were tagged as alternative by the methods in APPRIS.

The vast majority of the alternative isoforms (94.2% of variants tagged as alternative) are likely to have substantial structure and function changes. A total of 7,598 alternative splice variants would lose important functional or structural information relative to the principal isoform. APPRIS estimates that 4,056 alternative isoforms would lose part of a folded 3D structure, 1,539 variants would have fewer functional residues and 6,060 alternative isoforms would have damaged or lost Pfam functional domains. As many as 801 variants would lose at least one trans-membrane helix, while 316 would lose a signal peptide. Almost all alternative splice variants (8,049, 99,8% of variants tagged as alternative) had fewer cross-species orthologues in the protein databases (from the results of CORSAIR).

4.3 Comparison of lynx and human gene annotations

A comparison of the numbers of Iberian lynx genes annotated with features from the APPRIS modules against the number of genes annotated in the human genome (Table S11) suggests that there are a similar number of genes with protein features in both species. Iberian lynx has slightly more genes with functional residues and slightly fewer genes with functional domains, for example. The biggest differences between the two genomes are in the number of genes that have orthologues that align well and with without gaps (CORSAIR) and in the number genes with signal sequences (CRASH). Those differences are magnified when the comparison is made as a proportion of the total annotated set of coding genes (Figure S12) because lynx is annotated with almost 3,000 extra genes.

The CORSAIR results are strikingly different from the human genome (probably the most in-depth annotated vertebrate genome). There are only 3,004 human

genes with transcripts that do not correctly align to any other species. In Iberian lynx 15,328 of 23,245 genes do not align correctly with any other species. This is due to the incomplete nature of the assembly, it is inevitable that there will be many more gene fragments, annotation errors, gene model errors and pseudogenes than in a genome like the human genome, which has had many years of hand-curation. APPRIS can be used to indicate where the lynx genome will need to be refined in future.

Table S11. The number of human and lynx genes with annotations from each individual APPRIS module.

	**Human**	**Lynx**
firestar	6,945	7,578
**Matador3D**	12,575	12,349
**SPADE**	16,229	16,051
**CORSAIR**	17,469	*9,718*
**THUMP**	4,937	4,823
**CRASH**	1,893	*1,090*
**Total genes**	20,481	23,246

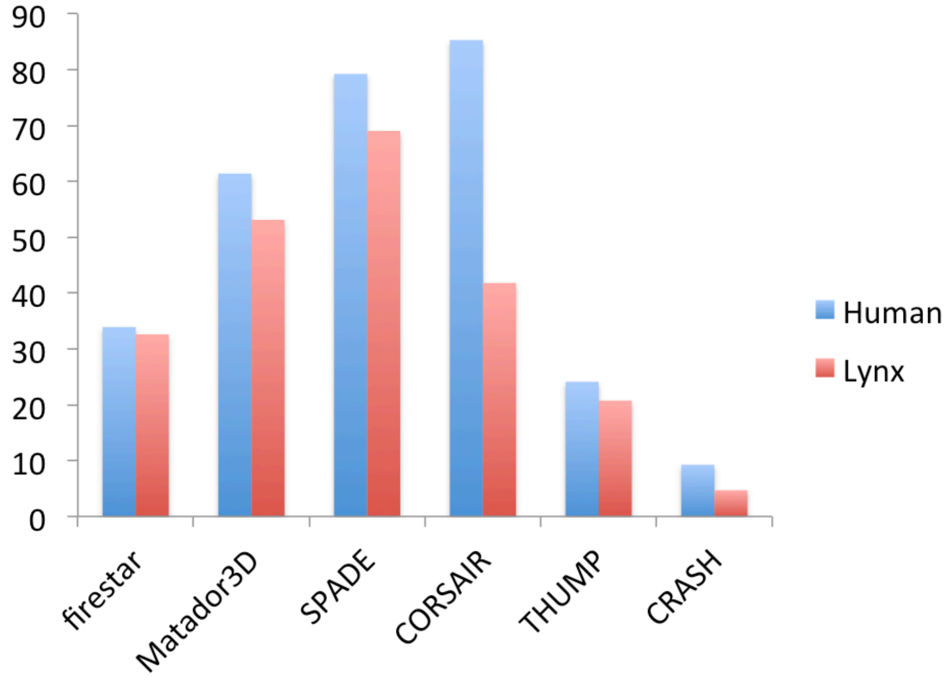

Figure S12. The percentage of human and lynx genes with annotations from each individual APPRIS module

5 Manual annotation and comparative analysis of lynx protease genes

Proteases form a diverse group of enzymes that share the ability to hydrolyse peptide bonds. The biological and pathological significance of this enzymatic activity has prompted the definition of the degradome as the complete repertoire of proteases in an organism[58]. From a genomics point of view, the degradome is highly attractive for several reasons. First, the degradome is composed of a large number of genes. For instance, the human degradome includes more than 580 protease genes, which represents about 1.7% of the total annotated human genes [59]. On the other hand, the number of known proteases allows the genomic study of the degradome with computer-assisted manual methods which can complement and validate automatic methods [60]. We have previously used this approach to discover copy number variations, gene gains or losses and inactivating mutations in proteases from different animals including mouse, rat, platypus or zebra finch [61-64]. We have also compared the human degradome with those of other primates such as chimpanzee [65] and orangutan [66]. Additionally, and because proteases have been related to a wealth of biological and pathological processes [67], we have used this accumulated knowledge on protease functions to raise hypotheses that link the genomic sequence to biological traits in a given organism. In this regard, it is remarkable that comparative genomic analyses of diverse degradomes have singled out the reproductive and immunological systems as the main protease evolution drivers [19, 68-70].

As expected, the comparative analysis of the degradome of the Iberian lynx showed a high level of similarity with those of domestic cat and Siberian tiger (Additional file 2, Datasheet S1). In some particular instances, this analysis suggests important milestones in the evolution of felids, probably related to challenges to their immune system. Thus, compared to the human degradome, felid degradomes feature multiple functional paralogues of the cysteine

proteases *CTSL* and *CTSL2*, ranging from 5 to 10. In turn, the human genome contains several tandem *CTSL*-like pseudogenes which might be phylogenetically related with the novel felid genes [71]. These lysosomal cysteine proteases are known to degrade collagen and elastin, but also include alpha-1 protease inhibitor, which might affect extracellular matrix homeostasis [72] and immune regulation [73]. Interestingly, it has been shown that CTSL plays a role in viral infection in humans [74-76]. Therefore, the conservation of multiple functional *CTSL*-like genes in felids might be related to the prevalent viral infections in these species. Notably, four of these novel *CTSL*-like genes have been pseudogenized in lynx and not in tiger or cat. These pseudogenizations have been confirmed by examining the paired-end realignment of Candiles to the final reference assembly (mean depth = 96x) and by the absence of SNPs falling within their premature codons (Table S12). However, some of the cat *CTSL*-like genes are predicted as catalytically inactive, due to variants in the active site. This suggests that *CTSL*-like genes were genomically amplified in a common ancestor to Laurasiatheria and Euarchontoglira and their function conserved in Felinae. Nevertheless, a closer inspection of the human pseudogenes suggests that their origin might be independent, since most of them share a common stop codon not present in lynx *CTSL*-like pseudogenes. The evolution of *CTSL*-like genes seems extremely dynamic with important changes between felids.

Table S12. Inspection of PE alignments and counts of SNVs in premature stop codons of four putative pseudogenes in the degradome.

Pseudogene	Order[1]	Scaffold	Start	End	Exon	Strand	Coverage in Candiles' 96x[2]			No. SNVs[3]
							1st Base	2nd Base	3rd Base	
c01l_ctsl3	1	lp23.s00351	34,603	34,605	3	-	T (116/116)	C (115/115)	A (116/116)	0
	2	lp23.s00351	34,597	34,599	3	-	T (111/112*)	T (112/112)	A (111/111)	0
c01l_ctsl4	1	lp23.s00351	82,828	82,830	1	+	T (117/117)	G (116/116)	A (117/117)	0
c01l_ctsl5	1	lp23.s10928	3,326	3,328	2	-	T (117/118*)	C (118/118)	A (117/117)	0
c01l_ctsl9	1	lp23.s37008	4,486	4,488	3	-	T (122/122)	C (121/121)	A (121/121)	0

[1] Order of appearance of the earlier stop codons in each CDS (1 and 2 referring to two consecutive ones).

[2] Coverage of PE data realigned to the reference assembly in each particular position of the codon. Each cell contains the sequenced base followed by the proportion of reads supporting in parenthesis. Note sequenced base is reported only for the forward strand.

[3] Total number of SNVs from the RubioSeq SNV dataset that are located at any of the STOP codon bases.

* Alternative base is a G in only one read with base phred quality of 11.

A second felid-specific feature which might be related to the regulation of the immune system was detected in the cluster of neutrophil granule proteases, which in humans contains the serine protease genes *AZU1*, *ELA2* and *PRTN3*. In primates, the multiple genomic changes through evolution suggest that this cluster is under strong selective pressure [77]. In felids, this cluster lacks *AZU1* and *PRTN3*, both of which seem to have been partially deleted. A second related cluster, called granzyme cluster, is also lacking one of the serine proteases (*GZMM*) in felids. Taken together, these data suggest that the immune system is an important driver of felid evolution and that it may be important for the conservation of the Iberian lynx.

In the course of this analysis, we were able to find almost all of the expected proteases. In a few cases, like the aspartyl protease *PSH2*, only a small stretch of the gene was present. Only for the metalloprotease *ADAM8* we could find no solid evidence of its presence anywhere in the lynx genome. Since this gene is very well conserved throughout evolution and clearly present in cat (ENSFCAP00000016289), this absence is likely due to the limitations of the assembly. As a proxy for assembly quality, out of the 635 genes analyzed, we have completely annotated 306. The annotation of the rest of the genes was incomplete, in principle due to absent regions in the assembly, although in a few cases this might be caused by the complexity of the genes themselves. These figures are close to those found in other projects with *de novo* assemblies, including those using the Sanger method with longer reads. In summary, the annotation of the degradome suggests that the current state of the assembly is satisfactory for gene annotation.

6 Transcriptome characterization

Reads from RNAseq samples obtained from 11 tissues (Table S13) were aligned to the reference assembly using GEMTools RNAseq pipeline v.1.6.2. This pipeline

uses GEM as a mapper [7], which performs full paired-end, quality-aware alignment and does not require preliminary filtering by quality. At the same time GEM is split-aware, and finds gapped matches, that is, it can correctly align reads into the genome originating from exon junctions. The read mappings were performed allowing less than 4 mismatches and less than 10 multimappings. On average, 79±2% of the reads were mapped across samples, 75±3% of the reads mapping uniquely.

Flux Capacitor v.1.2.4 [78] was used to quantify genes, transcripts, exons and splice junctions in each sample separately. Expression levels were obtained in pure read counts and in Read Per Kilobase per Million mapped reads (RPKM) [79].

6.1 Distribution of reads among genomic elements

Prior to the analysis, we checked whether RNAseq reads were mapped into genic regions, and specifically to exons. As expected, the majority of the RNAseq reads were mapped into exonic genomic regions, with some variability between samples (69.5-89.5%). We found that 0.3-16% of the reads mapped to intergenic regions, the maximum number belonging to the brain sample, and 5-17% of the reads mapped to introns. We also computed the proportion of detected genomic elements - genes, transcript, exons and junctions, for all samples for protein coding genes (PCG), lncRNAs and for both of them. A threshold of ≥0.3 RPKM was used to distinguish expressed from unexpressed elements. Cumulatively, we detected ~78% of the annotated genes, ~85% of transcripts and junctions, and ~92% of annotated exons for PCG across all samples. For the lncRNA we detected expression activity for ~67% of genes. The proportions for genes expressed in individual samples were similar across samples: 51-67% for PCG and 25-38% for lncRNAs. On average, ~12,000 PCG and ~1,600 putative lncRNA genes were expressed in one sample. The expression level of the lncRNA genes is very low compared to the PCGs, although 211 (4%) of these genes showed a high expression level (>10 RPKM).

6.2 Tissue expression patterns

To analyze differences in gene expression patterns, we plot the RPKM profile for each sample for the PCG and lncRNA genes independently (Figure S13). Genes have been sorted by expression values and then their RPKM was calculated. All samples, except pancreas, show smoothed curves, corresponding to the slow accumulation of the RPKM fraction with the number of genes. The top 10 PCG genes with the highest expression in pancreas tissue are capturing more than 50% of its whole transcriptomic activity, which is a larger percentage than in other tissues. All of these genes encode for enzymes, such as trypsin or pancreatic triacylglycerol lipase, and their GO terms are shifted towards the digestion and proteolysis biological process.

a)

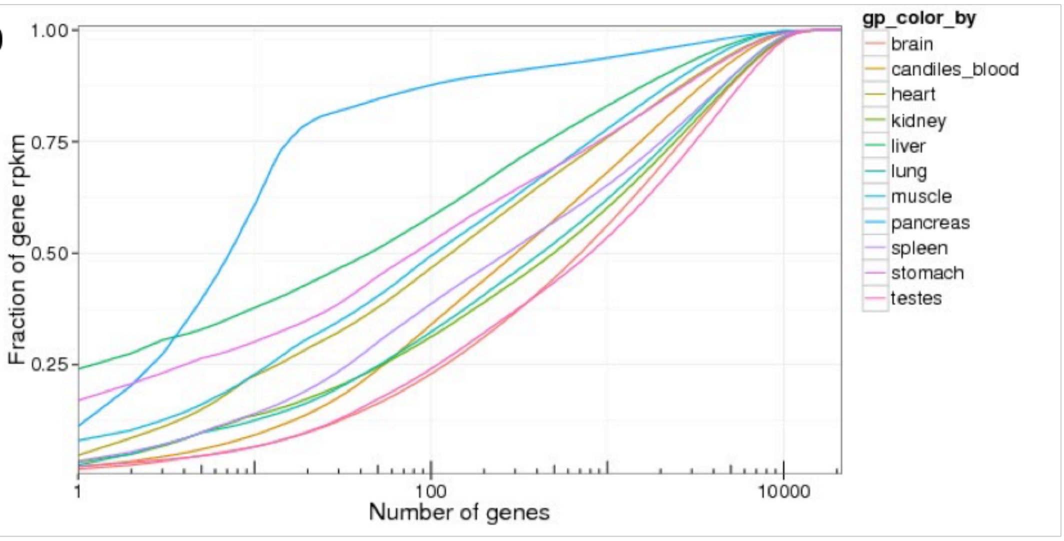

b)

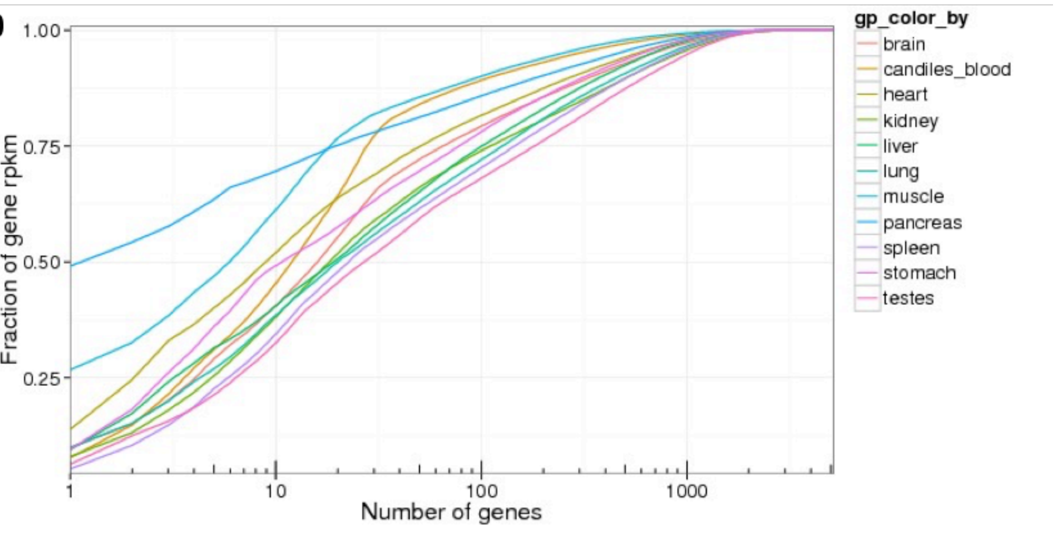

Figure S13. RPKM fraction profiling of protein-coding genes **(a)** lncRNAs **(b).**

6.2.1 Tissue specificity

We studied the patterns of gene specificity by creating a matrix of expression for the different tissues and selecting those that were exclusively expressed (RPKM>0.3) in one tissue (Table S13).

Table S13. Gene expression values and tissue specificity for the different tissues analyzed. #TSG number of Tissue Specific Genes or genes that are only expressed in that particular tissue.

		RPKM					RPKM		
**Tissue**	**#Expressed genes**	**Mean**	**Median**	**SD**	**# TSG**	**% TSG**	**Mean**	**Median**	**SD**
Liver	11,023	36	4.25	935.84	48	0,44	2.15	0.79	3.70
Muscle	11,202	32.77	4.88	350.26	44	0,39	27.61	1.57	74.93
Heart	11,748	25.13	4.45	231.23	53	0,45	2.47	0.60	5.38
Pancreas	12,042	57.59	2.89	1370.74	33	0,27	0.89	0.47	1.03
Stomach	12,679	34.25	6.06	694.37	39	0,31	0.70	0.48	0.55
Blood (Candiles)	12,922	30.29	6.40	152.76	45	0,35	2.70	0.68	10.81
Lung	13,453	23.81	7.05	146.28	62	0,46	1.51	0.59	2.41
Spleen	13,551	23.55	6.78	161.93	185	1,37	2.92	0.77	7.33
Kidney	13,720	20.34	6.36	128.88	103	0,75	1.25	0.60	2.01
Testes	13,975	19.89	7.75	81.20	168	1,20	1.60	0.66	2.52
Brain	14,310	19.26	5.87	73.69	385	2,69	1.04	0.65	0.91

As seen in other studies [80], brain had the highest number of expressed genes and also of tissue specific genes, followed by spleen and testes. In this experiment the number of tissue specific genes in brain might be a bit inflated since we do not have an evolutionary or functionally 'close' tissue to compare with. Tissue specific genes had much lower RPKM values on average.

6.2.2 Differential gene expression.

Differential gene expression (DGE) analysis across tissues was performed with Bioconductor package edgeR v.3.4.2 (R v. 3.0.2) [81] using classic pairwise comparison. For the estimation of the differential expression only genes with 1 count per million (CPM) were taken, in total 15,785. Following the recommendations by the package's authors, we chose a value for the biological coefficient of variation (BCV) of 0.4. Genes that had false discovery rate (FDR)

≤0.05 and log2 Fold Change log2(FC) ≥1 were determined as differentially expressed between tissues. In addition, we identified genes with preferential expression in one tissue (FDR ≤0.05 and log2(FC) ≥1). Similarly to TSG and DGE results, brain and testes have more genes preferentially expressed compared to the other tissues, while pancreas has a very low number. GO terms enrichment analysis of the preferentially expressed genes shows significant enrichment of the terms that have been previously associated with each particular organ. Genes that are preferentially expressed in brain have the largest number of the terms enriched (588), including terms related to neuron development and memory processes (Table S14).

Table S14. Number of GO terms enriched (p-value ≤ 1e-5) in the genes that are over expressed in a specific sample. Last column represents some of the top-terms examples, with p-value ≤ 1e-15

Sample	# preferentially expressed genes	# GO terms enriched	Some examples from the top-terms
Brain	1871	588	Neuron development, synaptic transmission, cell-cell signalling, learning or memory
Blood	821	151	Blood vessel, vasculature development, extracellular matrix organization, anatomical structure morphogenesis
Heart	332	200	muscle structure development, heart process, blood circulation, striated muscle contraction, regulation of cardiac muscle cell
Kidney	402	99	transmembrane transport, excretion, kidney development, vitamin D metabolic process
Liver	464	368	organic acid metabolic process, protein activation cascade, lipid metabolic process, regulation of humoral immune response
Lung	420	265	blood vessel development, endothelial cell proliferation, circulatory system development, angiogenesis, response to stimulus
Muscle	560	220	muscle system process, actin-myosin filament sliding, ubiquitin-dependent protein catabolic, signal transduction
Pancreas	89	31	Digestion, proteolysis, insulin secretion, hormone transport, endopeptidase activity, lipase activity
Spleen	712	403	immune system process, leukocyte activation, T cell activation, defense response, hemopoiesis, adaptive immune response
Stomach	337	26	Digestion, small molecule metabolic process, bicarbonate transport, regulation of pH, ligase activity
Testes	914	115	cilium morphogenesis, cell projection assembly, regionalization, spermatogenesis, gamete generation

6.3 Comparative gene expression analysis

6.3.1 Comparison with the Brawand et al. data

Supplementary Data files with normalized expression values from the paper Brawand et al. [80] were downloaded from the Nature web site. This dataset contains 5,628 one-to-one orthologus genes from nine mammalian species – human, chimpanzee, orangutan, gorilla, bonobo, macaque, mouse, opossum and platypus, and a bird – chicken, with their expression values in brain, cerebellum, heart, kidney, liver and testes samples. Each somatic tissue is represented by at least one male (5 for brain in human and chimpanzee) and one female samples, and testes tissue have two male samples per specie. Orthologus gene IDs are provided in the Ensembl format; expression values are provided in Read Per Kilobase per Million mapped reads (RPKMs) [82]. For comparison with lynx expression data only male samples from brain, heart, kidney, liver and testes tissues were considered; females and cerebellum samples were excluded. We refer to this data set as the Evolution Data Set (EDS).

One-to-one orthologus genes containing Ensembl IDs between lynx and human were selected from Phylome DB [83]. In total, we selected 4,191 candidate genes from which 1,888 genes were in intersection with orthologus genes obtained from Brawand et al. paper. Samples from brain, heart, kidney, liver and testes tissues were taken for downstream analysis; blood and samples from pancreas, muscle, lung, spleen and stomach tissues were excluded. Quantitative gene expression data for all lynx samples was obtained as described above. We consider a gene to be expressed with at least 0.1 RPKM in at least one of the selected samples; genes without expression were not considered in the downstream analyses. In total, we used 1,838 genes for inter-specific comparative analysis. We refer to this as the Lynx Data Set (LDS).

As a first overview of the data we compared overall levels of the gene expression in the two sets (LDS and EDS) in logarithmic scale. We found that all lynx samples show consistently lower expression levels than the EDS species, while EDS samples shows quite uniform expression distribution across species regardless of the tissue (data not shown). This can be explained by the batch

effect – the systematic error introduced in the sample preparation or raw data filtering at different places. Such a difference precludes the direct comparison between species before an additional normalization step. To remove systematic batch error we have applied upper quartile (UQ) normalization on the raw RPKMs values [84] using the function from the R Bioconductor package *preprocessCore* v. 1.24.0. This approach totally corrected the systematic biases observed in the data before normalization (data not shown).

We then performed a principal component analysis on the normalized merged data set (Figure S14). In a good concordance with previous observations, including Brawand et. al., samples are clearly separated by tissues with little variation among species. Among all samples, testes show more species variability compared to the rest of tissues, and testes sample from lynx stands out as an outlier with respect to testes from the rest of species.

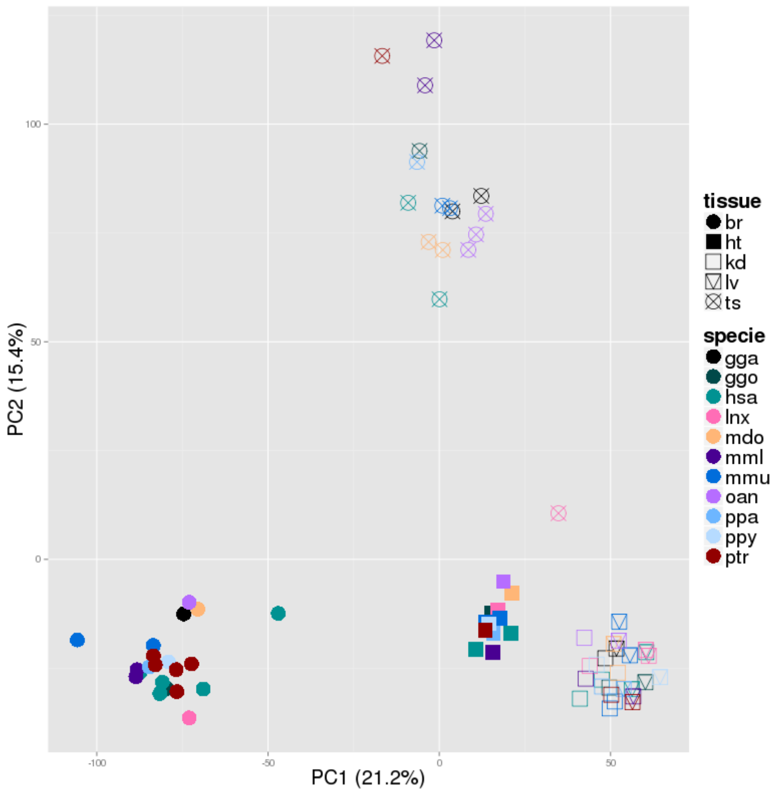

Figure S14. Principal-component analysis of the gene expression in merged data set. In parenthesis proportion of variance explained by selected components.

To identify genes differentially expressed in lynx with respect to other species we used NOISeq Bioconductor package v. 2.6.0 [85]. NOISeq is a non-parametric method with no assumption on the data distribution; it tries to discriminate whether observed differences in gene expression represent true differential expression or are likely to be part of the noise. This package does not require pure read counts and can use RPKM normalized values; also, it can generate simulated replicates from a multinomial distribution according to the specified parameters. Here we used default parameters recommended by authors for studies without replicates: pnr = 0.2 (percentage of the total reads used to simulate replicates), nss = 5 (number of replicates) and v=0.02 (variability in simulated samples). Genes are considered to be differentially expressed when q-value >=0.8.

We performed a tissue by tissue comparison of lynx expression with each of three species groups: between lynx and all other species; between lynx and mammalian species (human, chimpanzee, orang-utan, gorilla, bonobo, macaque, mouse, opossum and platypus); and between lynx and eutherian species (human, chimpanzee, orang-utan, gorilla, bonobo, macaque and mouse). We get an increasing number of differentially expressed genes with decreasing number of species in the group compared (Table S15). For example, in brain tissue we get 22 genes differentially expressed between lynx and all other species, and 52 genes between lynx and eutherian species. For each tissue the subset of genes being differentially expressed in lynx with respect to all other species is fully included into the set of genes being differentially expressed with respect to the other groups – mammalian and eutherian. This is within expectations and due to the decreasing number of samples in the selected groups.

Table S15. Differentially expressed genes for each tissue between lynx and other species. In parenthesis in header row is the number of species; in parenthesis in cells are number of up-regulated / down-regulated genes in lynx.

	**Lynx vs Eutheria (7)**	**Lynx vs Mammalia (9)**	**Lynx vs All (10)**
**Brain**	52 (8 / 44)	23 (3 / 21)	22 (2 / 20)
**Heart**	55 (18 / 37)	37 (15 / 22)	30 (10 / 20)
**Kidney**	82 (35 / 47)	78 (31 / 47)	64 (23 / 41)
**Liver**	89 (30 / 59)	57 (20 / 37)	49 (18 / 31)
**Testes**	146 (61 / 85)	115 (43 / 72)	89 (30 / 59)

To get insights on functions of genes up- and down-regulated in lynx, we performed a GO terms enrichment analysis on the smallest subset of differentially expressed genes "Lynx vs All" in each tissue. The GO terms enrichment analysis was done with the Bioconductor package topGO v.2.14.0.

Genes up-regulated in lynx brain were excluded from GO enrichment analysis because there were only two genes at the selected threshold: LYPA23B007493 (annotated as corticosteroid 11-beta-dehydrogenase) and LYPA23B008779 (annotated as MORN repeat-containing protein).

Genes down-regulated in lynx in brain tissue are enriched for receptor binding and corticosteroid regulation function terms, pval=9.0e-4. In heart sample, genes up-regulated in lynx are enriched for histamine transport, (pval=5.0e-4), and those down-regulated are enriched for processes related to vitamin A (retinol) metabolism, (pval=2.3e-5). Genes up-regulated in kidney are enriched for response to interleukin-1 (pval=1.9e-5) and down-regulated genes were for small molecules metabolic processes (pval=3.2e-6). In the liver tissue up-regulated genes are enriched for toxin and dipeptide transport activity (pval=1.8e-5), and those down regulated were for different peptidase activities (pval=2e-4). In testes genes up-regulated in lynx are enriched for tissue development processes (pval=6.5e-5), and down-regulated for sexual reproduction and spermatogenesis, (pval=1.9e-6).

6.3.2 Comparison of lynx and cat testes RNAseq data.

RNA-seq of testicle transcriptome from domesticated cat *Felis catus* was downloaded from the public Short Read Archive (SRA), experiment ID is SRX193575; this dataset contains 68 million single end Illumina v.1.9 reads with length 50 nt. Lynx RNA-seq testes data are paired end Illumina v.1.5 reads with length 75 nt, 2 x 31 million reads in total. Due to problems with the mapping of the cat data, initial reads were pre-processed –adapter sequences, reads containing Ns and bad quality reads were removed. In total, 48 M reads (70%) from the cat sample were used. RNAseq reads from both cat and lynx samples were aligned as single-end data to the reference assembly v. lp23 with STAR program [86]. We got 86% of cat reads and 89% of lynx reads mapped to the assembly, respectively, with 76.5% and 81% of the reads mapped uniquely. Flux

Capacitor [6] v.1.2.4 was used to quantify genes in each sample separately. Expression levels were obtained in pure read counts and in RPKMs. Taking into account difference in initial RNA-seq data in both samples (different number of reads, length and platform), we decided to process DGE analysis on the normalized RPKM values.

NOISeq Bioconductor package v. 2.6.0. with 5 simulated replicates was used to obtain differentially expressed genes. With qval>=0.8 we get 671 DGE where 62 genes are up-regulated in lynx sample and 609 are up-regulated in cat sample. Interestingly, the majority of DGEs were expressed only in one specie – either only in cat, 585 genes, or only in lynx, 54 genes. We consider a gene to be expressed only in one specie if it shows >=0.1 RPKM in the given specie and < 0.1 RPKM in the other. Table S16 contains results of GO terms enrichment analysis for four subset of genes – expressed only in one specie (cat or lynx) and up/down regulated genes between two species. Genes up-regulated or specifically expressed in cat are enriched for terms related to sexual reproduction and spermatogenesis. Enriched terms for genes up-regulated or specifically expressed in lynx were related to lipase activity and cell junctions. This result of GO enrichment is in an agreement with the one obtained by comparing lynx testes samples with the data from Brawand et. al. (2011).

Table S16. GO terms enrichment analysis for differentially expressed genes between cat and lynx testicle samples; number of genes is specified in parenthesis.

Genes expressed only in cat (585)	***Genes expressed only in lynx (54)***
GO:0019953 sexual reproduction<br>GO:0007338 single fertilization<br>GO:0044702 single organism reproductive process<br>GO:0007283 spermatogenesis<br>GO:0048232 male gamete generation<br>GO:0009566 fertilization<br>GO:0035036 sperm-egg recognition<br>GO:0032504 multicellular organism reproduction<br>GO:0009988 cell-cell recognition<br>GO:0000003 reproduction	GO:0070830 tight junction assembly<br>GO:0043297 apical junction assembly<br>GO:0009055 electron carrier activity<br>GO:0007043 cell-cell junction assembly<br>GO:0034329 cell junction assembly<br>GO:0007586 digestion<br>GO:0006414 translational elongation<br>GO:0022625 cytosolic large ribosomal subunit<br>GO:0033087 negative regulation of immature T cell p...
Genes up-regulated in cat (24)	***Genes up-regulated in lynx (9)***
GO:0030317 sperm motility<br>GO:0000237 leptotene<br>GO:0019244 lactate biosynthetic process	GO:0004806 triglyceride lipase activity<br>GO:0044241 lipid digestion<br>GO:0016042 lipid catabolic process

from pyruva..<br>GO:0019249 lactate biosynthetic process<br>GO:0019516 lactate oxidation	GO:0015918 sterol transport<br>GO:0030301 cholesterol transport<br>GO:0002776 antimicrobial peptide secretion<br>GO:2000478 positive regulation of metanephric glome...<br>GO:2000534 positive regulation of renal albumin abs...<br>GO:0016298 lipase activity<br>GO:0052689 carboxylic ester hydrolase activity

The gene expression patterns detected in different organs of Iberian lynx are globally similar to those reported for human and other mammalian species, while they reveal some peculiarities. Some of these could be related to species-specific adaptations, but most might just reflect differences in physiological status of the individual sampled, related to age or health status, for example. Differences in gene expression in lynx testes are concordant with lynx marked seasonality in testicular activity and the lynx being sampled in October, a period of low activity ([87], Eduardo Roldán, pers. comm.); this is in sharp contrast to cat and the other mammals analysed which are not seasonal and produce sperm during all year. It must also be noted that the sampled lynx was euthanized after a chronic renal disease causing renal failure, which was due to vitamin D toxicosis [88]. This fact might have altered gene expression in kidneys and indirectly in other organs, including a further reduction of the expression of genes related to spermatogenesis in testes, as affected individuals have been observed to have lower sperm counts and smaller testes (Eduardo Roldán, pers. comm.).

7 Evolutionary profiling and expression of lncRNAs

7.1 Evolutionary profiling

The transcripts found in lynx were compared against 11 other genomes (cat, panda, dog, horse, human, cow, pig, bat, mouse, rat, opossum) using the homology-based method described above. This way, for every transcript, the set of species in which it is conserved (as well as its % identity with the lynx

sequence) was obtained (Figure S15). Systematic phylogenomics revealed 61 lynx-specific lncRNA genes and a core set of 347 genes expressed in lynx that are conserved in all genomes.

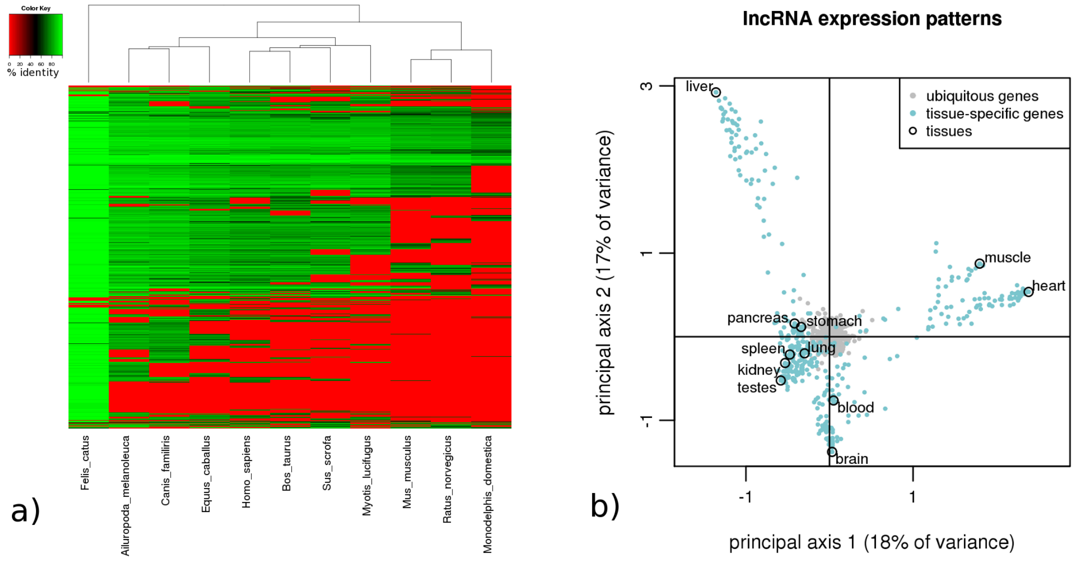

Figure S15. Sequence conservation heat map of 3118 predicted transcripts that are conserved in at least one species beyond lynx. Non-conserved transcripts are shown in red, shades of green indicate conserved transcripts. **b)** Correspondence Analysis of 2475 genes expressed in 11 tissues. Genes and tissues form the rows and columns of a data matrix of which they share the same singular values and can thus be plotted in the same coordinate system. We obtain a clustering of tissues and genes as well as associations between them. Shown are only ubiquitous (around the origin) and tissue-specific genes (close to their respective tissues), although all expressed genes enter the analysis

7.2 LncRNA expression analysis

We quantified the expression of lncRNA transcripts in all RNA-seq samples as described for expression of protein coding transcripts. Gene expressions were obtained adding transcript expressions of each locus. A non-coding gene was considered expressed if it has at least 0.1 RPKM in at least one tissue (for PCGs we chose 0.5 RPKM as a cut-off). To define ubiquitous and tissue-specific genes, a cut-off of 0.5 RPKM was chosen also for non-coding genes (Figure S15b). Pearson correlation between lncRNAs and PCGs was calculated on the log_2-transformed RPKM values (using a pseudo count of 0.1 RPKM). Correspondence Analysis was performed on log_2 RPKM values (with a pseudocount of 1 to obtain positive counts) using R with the package “ca” [89] as well as custom R scripts.

Gene expression profiling revealed 403 genes ubiquitously expressed in all tissues and 532 tissue-specific genes. Ubiquitous genes tended to be more strongly conserved than the rest of the genes, and their average expression increased with conservation (Figure S16, left panel). Interestingly, for the tissue-specific genes we observed the opposite trend, an anticorrelation of expression with conservation. These genes also tended to be more correlated with protein-coding genes (PCGs), a trend that increased with conservation (Figure S16, right panel).

To obtain the most relevant correlation of each lncRNA with PCGs, we determined the maximum absolute Pearson correlation coefficient (MAP) across tissues over all 16,143 expressed PCGs. Ubiquitous lncRNAs tended to be depleted of highly correlated genes. Against this trend, however, we found a subset of 16 lncRNAs that are both conserved in more than half of the genomes and highly correlated with PCGs (MAP>0.99), thus have hallmarks of housekeeping genes with a possible regulatory function.

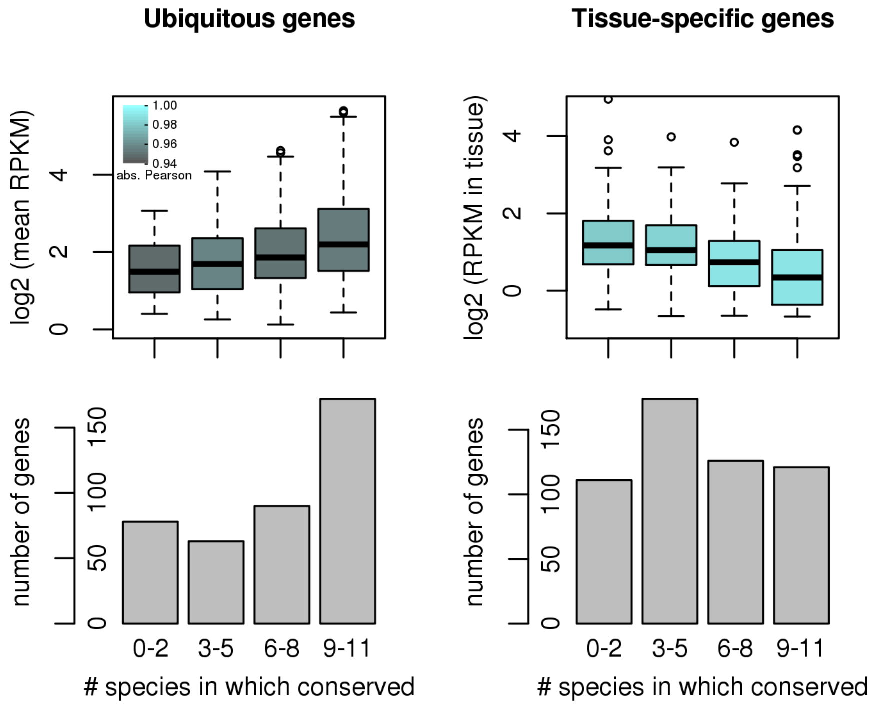

Figure S16. Ubiquitously expressed (left panels) and organ-specific genes (right panels). While ubiquitous genes tend to be highly conserved and their average expression increases with the amount of conservation, organ specific genes show the opposite trend. For them, average correlation with coding genes as measured by the group average over each lncRNA's maximum absolute Pearson across PCGs increases with conservation, consistent with a possible regulatory role of these genes.

7.3 Partial correlation network

Regularized partial correlations were determined using the procedure implemented in the R package "GeneNet" [90]. We then projected gene coordinates as weightless additional points using the principal coordinates obtained from the previously described CA of lncRNA genes to obtain an expression-driven clustering for better visualization.

To obtain the most relevant correlation of each lncRNA with PCGs, we determined the maximum absolute Pearson correlation coefficient (MAP) across tissues over all 16,143 expressed PCGs. Ubiquitous lncRNAs tend to be depleted of highly correlated genes. Against this trend, however, we find a subset of 16 lncRNAs that are both conserved in more than half of the genomes and highly correlated with PCGs (MAP>0.99), thus have hallmarks of housekeeping genes with a possible regulatory function.

Additionally, we analyzed coexpression between a list of 100 PCGs found to be positively selected in lynx (PSL) and our lncRNA genes. For this, we determined partial correlations between this set and all expressed lncRNAs. Partial correlations better reflect direct interaction between genes and are thus a preferred technique to build gene networks as compared to Pearson correlation [91]. We found 8 PSL genes with putative lncRNA partners below a 10% cut-off on local false discovery rate (Figure S17). We observe a testes-specific gene and two genes strongly expressed in kidney forming hubs with a large number of coexpressed lncRNAs.

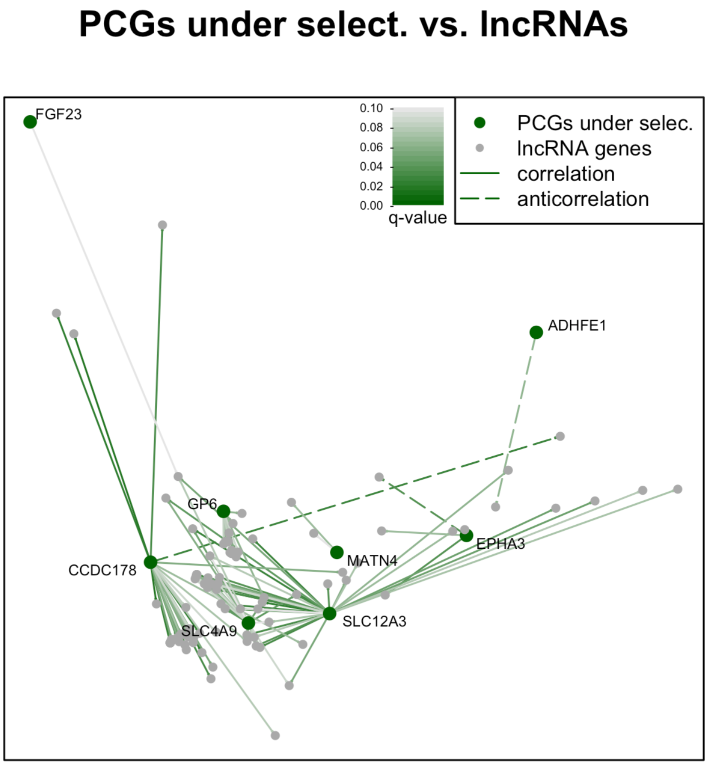

Figure S17. Coexpression network between 8 PCGs under selection showing significant partial correlation with lncRNA genes. Connections are colored according to their local false discovery rate (q-value), with the most trusted edges in darker shades of green. Only edges between lncRNAs and PCGs are shown. Genes are clustered according to their expression patterns using the principal coordinates of Figure S15b (where the PCGs are projected weightlessly). The most striking feature are the hubs formed by the testes-specific gene CCDC178 and the genes SLC4A9 and SLC12A3 (both strongly expressed in kidney).

8 Lynx orphan genes

An orphan gene is defined as a gene that lacks homologues in other lineages. Depending on which taxonomic level we are interested in, taxon-specific orphan genes (TSOGs) or species-specific orphan genes (SSOGs) can be defined [92]. Orphan genes were first discussed within the yeast genome project [93] and are thought to play an important role in adaptive processes [94, 95]. In this section we describe the identification and characterization of lynx orphan genes.

We developed a pipeline to identify lynx orphan protein-coding genes. First, we discarded any proteins that had homologues in any of 23 non-mammalian eukaryotic species indicated in Figure S18, using gene protein coding annotations from Ensembl. To search for homologs we used BlastP 2.2.23+ [96])

with an E-value threshold of 10^{-4} and the filter for low complexity regions activated. Second, we discarded any proteins for which we could indirectly trace homology to other species through a second protein in lynx. This could happen for example if the protein had evolved very rapidly after a gene duplication event [95]. For these searches we used BlastP with the same parameters as previously except that we used a BLOSUM80 matrix instead of the default BLOSUM62, as we were searching for sequences that had diverged relatively recently. Third, we classified the remaining proteins as lynx-specific or mammalian-specific depending on the presence of homologues in the annotated genes from *Felis catus, Canis lupus familiaris, Ailuropoda melanoleuca, Mustela putorius furo*, *Homo sapiens, Mus musculus, Bos taurus, Equus ferus caballus and Myotis lucifugus* (Ensembl version 72). Fourth, we only selected those genes expressed in at least one tissue using a RPKM threshold of 0.3. This resulted in the identification of 323 lynx-specific genes.

The current gene catalogs are likely to be incomplete and this means that some of these 323 putatively lynx-specific genes may correspond to not yet annotated genes in other mammals. We thus employed published RNAseq data for different tissues and mammalian species [80] to have a more comprehensive set of transcripts to compare our genes with. We run Tophat2 v2.0.8 [32] for pooled-tissues reads from human, mouse, chimpanzee, macaque and orangutan. Next, all long expressed transcripts (length > 200 nucleotides) were assembled using Cufflinks (v 2.0.2) [32] for each species and tissue separately, not using information from gene annotations (no reference GTF file). We used Cuffmerge to obtain a comprehensive set of transcripts for each species and Cuffcompare to classify the transcripts into already known transcripts (annotated, using GTF files corresponding to Ensembl v. 60) and novel transcripts (non-annotated). The number of transcripts per species is displayed in Table S17. Finally, we ran tBlastX with an E-value threshold of 10^{-6} to search for homologues of the 323 putative lynx orphan genes among these transcripts. After discarding any gene that had at least one match, the list of lynx orphan genes was reduced to 204 (206 transcripts; Additional file 2, Datasheet S2).

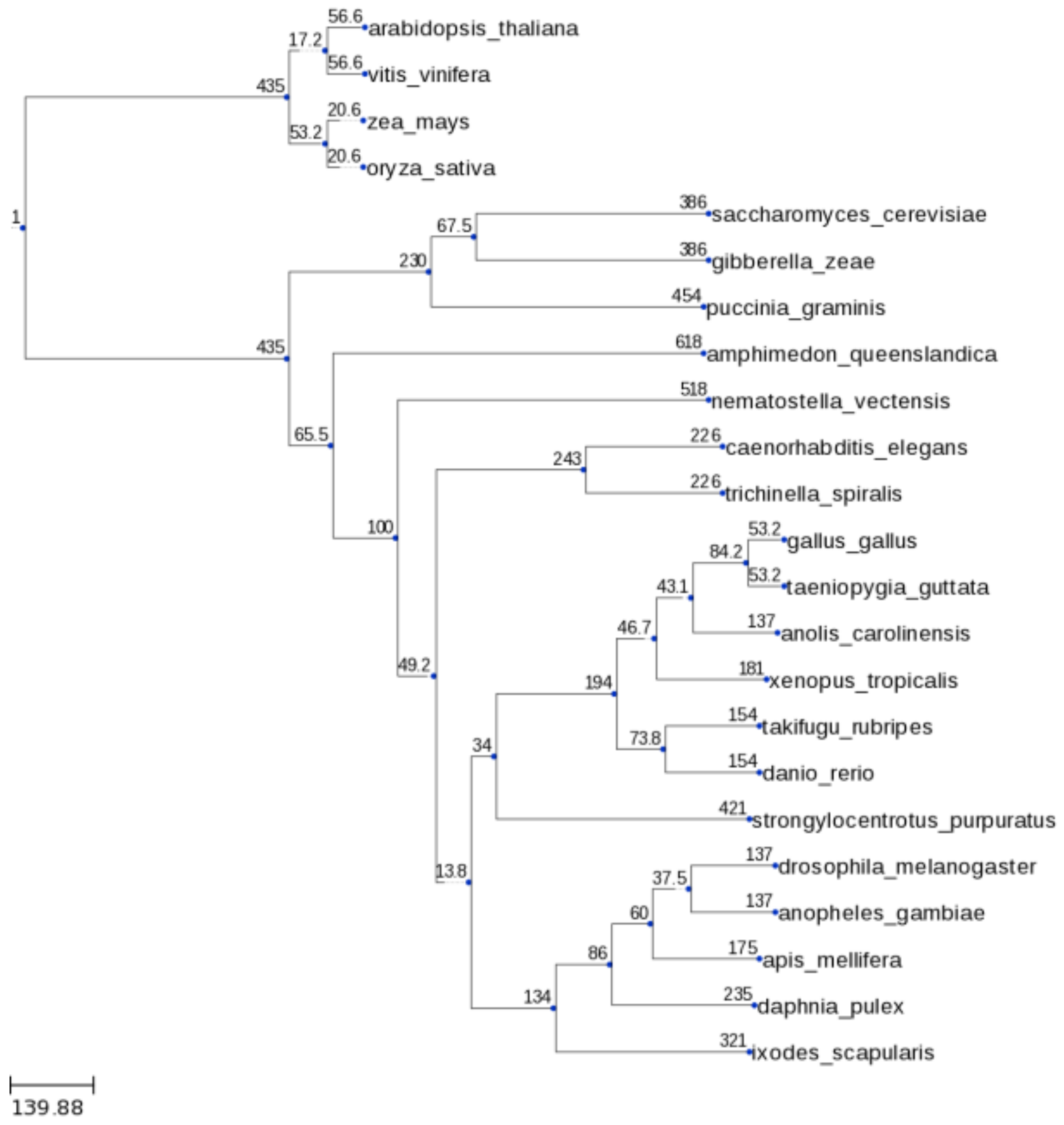

Figure S18. Tree showing the set of 23 non-mammalian species used to identify Iberian lynx orphan genes. Distances are in million years and were obtained from TimeTree.org.

Table S17. Number of assembled transcripts (annotated and novel ones) per species using RNAseq data.

	Human	Chimpanzee	Macaque	Orangutan	Mouse
**Annotated transcripts**	77,597	44,067	23,414	17,156	15,876
**Novel transcripts**	67,573	98,979	138,240	111,858	99,315

Using the RNAseq Iberian lynx transcriptomics data we investigated if there were any biases in the tissues in which the lynx orphan genes were expressed. Brain stood out as the tissue most highly enriched in expressed orphan genes (Table S18).

Table S18. Summary of expression data for 206 lynx orphan transcripts. Mean, median and SD refer to RPKM values for Expressed genes (RPKM>0.3).

			RPKM				RPKM	
**Tissue**	**Orphan Genes**	**Mean**	**Median**	**SD**	**All genes**	**Mean**	**Median**	**SD**
Brain	91	23.73	0.63	109.57	14,310	19.26	5.87	73.69
Heart	57	7.75	0.75	25.80	11,748	25.13	4.45	231.23
Kidney	71	11.74	0.74	41.53	13,720	20.34	6.36	128.88
Liver	37	28.73	0.90	87.57	11,023	36	4.25	935.84
Lung	54	15.96	0.95	57.21	13,453	23.81	7.05	146.28
Muscle	39	148.60	1.03	772.29	11,202	32.77	4.88	350.26
Pancreas	34	41.70	1.68	114.09	12,042	57.59	2.89	1370.74
Spleen	55	13.07	1.08	47.67	13,551	23.55	6.78	161.93
Stomach	50	38.92	1.45	170.69	12,679	34.25	6.06	694.37
Testes	74	14.92	0.96	55.04	13,975	19.89	7.75	81.20

In an attempt to further characterize the expression of these genes in related species and given the importance of testes in the birth of new genes [97] we reconstructed the transcriptome of cat testicle using Tophat (v2.0.8, cat genome version 75 ENSEMBL), Cufflinks to reconstruct the transcripts and cuffcompare to determine which were novel and which were already annotated in the cat (Ensembl 75, v2.1.1). We generated the fasta file from the gtf and blasted the *Lynx pardinus* orphan genes with expression (206 proteins encoded by 204 genes) against this cat reconstruction obtaining no significant hits. This means that, at least with the current data, the 74 orphan genes expressed in lynx testes would be exclusive of the lynx lineage.

Species or lineage-specific genes tend to be short and enriched in repetitive sequences [98, 99]. We confirmed that lynx orphan genes are shorter than average, with a mean length of 112.3 amino acids compared to 537.2 for the rest of genes (Table S19 and Figure S19). About half of the lynx orphan genes contain low-complexity sequences as measured with the program SEG [100], which is less than for the rest of genes (73.11 %), but these genes contain on average

nearly 40% of their sequence covered by repeats (LCRs), which is a very high portion when compared to the rest of genes (average 9.22 %) (Table S19).

Table S19. Summary of properties for the set of 206 lynx-specific expressed proteins and the rest of expressed proteins (once we removed sequences with one or more 'X').

	Number of proteins	Length of proteins (aa)			Low complexity regions (LCRs)	
		Mean	Median	SD	% of proteins with LCRs	Mean % covered
**ORPHAN GENES**	206	113.4	94	64.83	51.5	39.32
**REST OF PROTEINS**	31,818	537.2	383	581.99	73.11	9.22

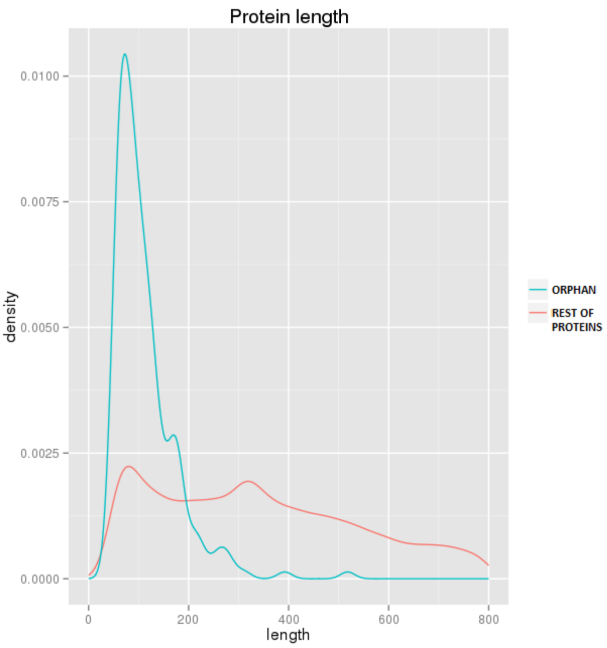

Figure S19. Distribution of lengths for the set of lynx-specific proteins compared to the rest of expressed genes.

It must be taken into account that the BLAST analysis was done with the low complexity filter activated. LCRs may result in spurious hits and this is the reason why they are normally not considered in sequence similarity searchers. However, by filtering these regions the identification of homologs is also hindered. This may have affected 106 of the 206 lynx-specific proteins (Table

S19). A case-by-case study should be done before drawing any conclusion for very highly repetitive proteins. The rest of proteins, 103, are not affected.

We crossed this list of lynx-specific proteins with the orthology information against 12 mammalian species, including 3 other felid species (*Lynx lynx, Panthera tigris, Felis catus*) used in the phylogenomics analyses (Section 13) and found that 6 proteins (LYPA23A005333P1, LYPA23A006200P1, LYPA23A006200P1, LYPA23A017426P1, LYPA23A018788P1, LYPA23A021612P1) have an ortholog in *Panthera tigris,* a species that was not used in our prior analysis.

9 Eurasian and Iberian lynx divergence

We extracted the autosomal contigs, after discarding about 2,265 scaffolds identified as best-hits to cat X chromosome and 3 to mitochondrial genome by using LAST [101] (Section 12), and obtained maximum likelihood estimates for either the isolation model of Mailund T, Dutheil JY, Hobolth A, Lunter G and Schierup MH [102] or the initial migration model of Mailund T, Halager AE, Westergaard M, Dutheil JY, Munch K, Andersen LN, Lunter G, Prufer K, Scally A, Hobolth A and Schierup MH [103]. The former model assumes a clean split model with an instantaneous speciation at some point in the past and has three parameters: the time of the speciation, the effective population size in the ancestral species and the recombination rate along the alignment. The latter model in addition allows for a period of symmetric gene flow – following an initial population split and before gene flow stops completely – and has two additional parameters: the time where the gene flow ends and the rate of gene flow during that period.

To estimate parameters we used a Nelder-Mead optimization as implemented in scipy's optimization module. The scripts used were "isolation-model.py" and "initial-migration-model.py" from https://github.com/mailund/IMCoalHMM. To estimate the uncertainty in parameter estimates we then split the autosomal

contigs into 44 sets each covering ~100 Mbp and estimated the uncertainty in the parameter estimates using a leave-one-out jackknife approach.

Estimated parameters for the isolation model and initial migration model are shown in Figure S20. For the migration model, the jackknife estimates of the two time points are very far from normally distributed but the maximum likelihood estimate is at the mode of the distributions. For the recombination rate, rho, the point estimate from the full data gives a value that falls outside the range of the jackknife samples, but this parameter is very poorly estimated in general [see Mailund T, Dutheil JY, Hobolth A, Lunter G and Schierup MH [102], Mailund T, Halager AE, Westergaard M, Dutheil JY, Munch K, Andersen LN, Lunter G, Prufer K, Scally A, Hobolth A and Schierup MH [103]] and often under-estimated by a factor of two, which is less than the difference between the mean jackknife estimate and the full data estimate. For the ancestral effective population size, the point estimate given by the full data set falls below most of the jackknife estimates and the mean jackknife estimate could potentially be a better point estimate for this parameter. Generally, though, the estimates give very tight error bars with the exception of *rho* and the migration rate.

As in Mailund T, Halager AE, Westergaard M, Dutheil JY, Munch K, Andersen LN, Lunter G, Prufer K, Scally A, Hobolth A and Schierup MH [103] we use Akaike's information criterion (AIC) to determine the most probable model. We estimate the AIC for the isolation model to be 7.3625×10^7 and for the migration model to be 7.3621×10^7. Since the migration model has the smallest AIC this is the preferred model.

The migration model estimates a structured population appearing at around the same time that the isolation model would estimate the split time, but the former model also estimates that limited gene flow has continued until very recently. It cannot distinguish between continuous gene flow and occasional admixture events, however. The exact times depends on the underlying mutation rate, μ, which is currently unknown for lynx (Figure S21). The mutation rate per site per year (μ) for Iberian lynx was estimated in 1.2×10^{-9}, based on the nucleotide divergence of lynx and cat observed in syntenic alignments (Section 12) and

assuming 7.2 Mya since the two species last shared a common ancestor [104], a value that falls within the range of mutation rates used with other felids [105].

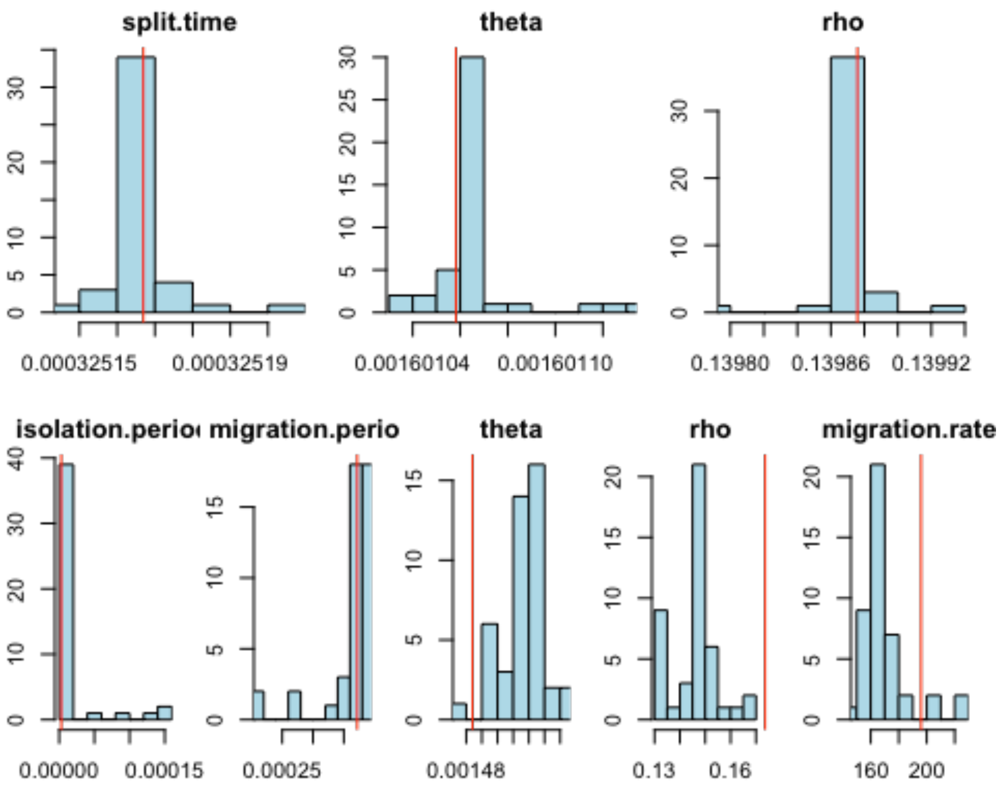

Figure S20. The plots show the results for each parameter with the maximum likelihood estimate on the full data shown as the red vertical line. The first plot shows the parameter estimates for the isolation model and the second plot the estimated parameters for the migration model.

Assuming this mutation rate we estimate that the initial population structure appeared 0.3122 Mya (95% CI: 0.1794 - 0.3231 Mya), using the maximum likelihood estimate for the full data, and gene flow ceased 2,473 ya (95% CI: 1.8×10^{-4} – 126.8 kya). For the initial split, the uncertainty in the estimates is very small and although there is a little variation between the maximum and minimum estimates this is almost certainly dominated by the uncertainty we have in the mutation rate. The final end of gene flow is poorly estimated compared to the other parameters but considering that the median estimate is only around two thousand years ago this analysis suggest that it has been maintained until recently.

The theta parameter is given as $\theta = 4Ng\mu$ where N is the effective population size in the ancestral population and g the mean generation length in years. If we

assume that $g = 5$ and a mutation rate of $\mu = 1.2 \times 10^{-9}$ mutations per nucleotide per year we get an ancestral effective population size of 62,002 (95% CI: 62,744 – 66,850).[19]

The estimated migration rate is in units of migration per substitution from one generation to the other (assumed to be symmetric). This means that $g\mu m$ is the rate of migration per generation for a single individual and $2Ng\mu m = \theta/2m$ is the migration rate for the entire population in a single generation. With this arithmetic, without assumptions about mutation rates or generation length, the scaled migration rate per generation is 0.1458 (95% CI: 0.1197 - 0.1800). This is estimated as a symmetric migration rate in each direction. The total exchange of genes is thus twice this.

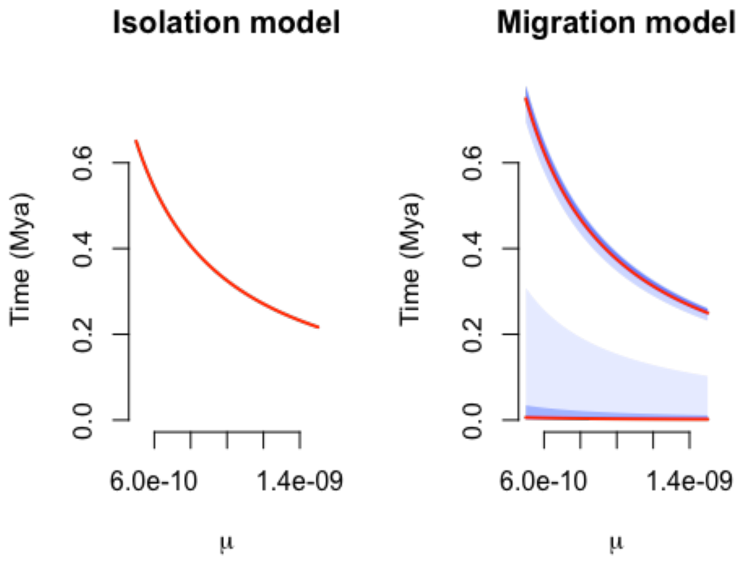

Figure S21. On the left is shown the split time estimate for the isolation model. The red line shows the maximum likelihood estimate from the full data set. There is very little variation in the jackknife estimates and the entire range of estimates for this parameter falls on the same line. On the right is shown both the initial population split and the end of gene flow for the migration model. The red lines are again the maximum likelihood estimates using the full data set. The jackknife estimates are shown as the colored area where the light blue area corresponds to the entire range of estimates and the darker blue to the 1st to 3rd quantile (middle 50% of the estimates).

[19] The point estimate falls outside the confidence in this case, as is also apparent from the histograms, since the point estimate with all data points included is lower than most of the jackknife estimates. The mean jackknife estimate is 64,721, which falls within the confidence interval.

10 Demographic history

We used two complementary approaches to infer the demographic history of Iberian lynx. The first uses a pairwise sequentially Markov coalescent (PSMC) model applied to complete diploid genome sequences of single individuals to reconstruct the demographic history of the species from the distribution of the local density of heterozygous sites [106]. The method seems to work well for periods between 10,000 to 1 million years b.p., but tends to overestimate recent population sizes and to spread sudden changes in population size over several preceding tens of thousands of years. For the second approach we used the maximum likelihood inference method implemented in the software *∂a∂i*, which searches for the most recent demographic history that better fits the observed allelic frequency spectrum (AFS) [107].

10.1 PSMC

We followed several steps to obtain the diploid consensus sequences required by PSMC for the analyses of demographic history [106]. First, the paired-end reads for each lynx genome (Table S2) were aligned against the genome assembly (*lp23*) using the BWA mapping software [108], version 0.6.1-r104. All reads were aligned using *aln* and setting the quality trimming parameter to –q = 15. The alignments were paired with the *sampe* command into a unique SAM file and subsequently filtered using flags as follows:

```
samtools view -q 20 -F 0x8 -h aln.bam |samtools view -Shb -F 0x4 -|samtools view -h -f 0x2 - | samtools view -Shb -F 0x400 - > sample.MQ20PropPairNoPCRdup.bam
```

Moreover, these BAM files were realigned around the indels using GATK (v2.5-2) [109]. The 2,265 scaffolds identified as best-hits to cat X chromosome and 3 to mitochondrial genome by using LAST [101] (Section 12) were filtered out to avoid biases on the estimates of effective population size as (N_e).

The diploid consensus was generated from the 'mpileup' command of the SAMtools software. Following the PSMC recommendation, the min depth was set to 1/3 of the average read depth and the max depth was set to twice the average [110] during the conversion. We estimated these thresholds from the average coverage for each sample. The purpose of the minimal read depth threshold is to

avoid false SNP calls, and that of the maximum read depth threshold is to exclude reads aligning to repetitive or paralogous regions.

We used the tool 'fq2psmcfa' from the PSMC package on the whole-genome consensus diploid sequences to create the input file for the HMM. We set parameters Tmax= 20, n = 64 ('4+50*1+4+6'), as done for *Sus scrofa [111]*, a species with the same generation time as Iberian lynx.

The raw PSMC outputs were scaled to time and population sizes assuming a generation time of five years and a range of mutation rates. The mutation rate per site per generation (μ) for Iberian lynx was estimated in 0.6×10^{-8} based on a rate of 1.2×10^{-9} per nucleotide per year (Section 9) and a generation time of five years.

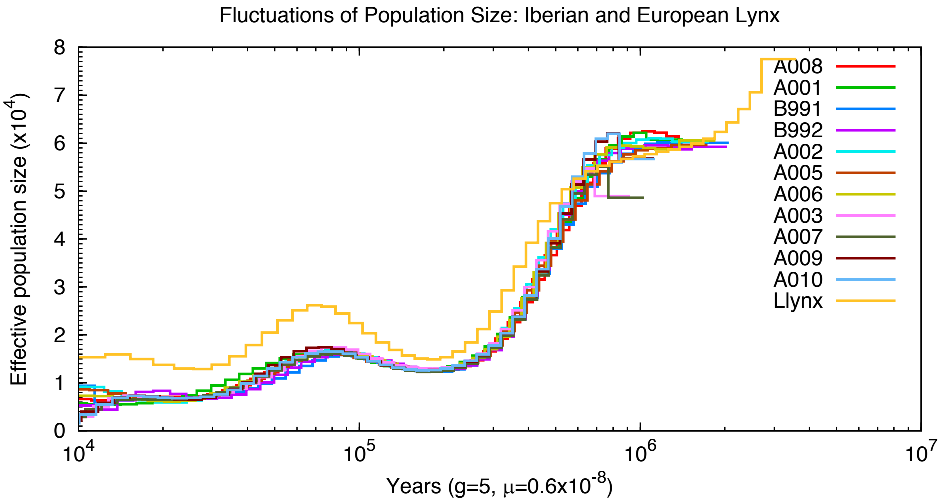

Figure S22. Demographic History of Lynx using PSMC. Effective population size through time estimated for each of the 11 Iberian lynx and a single Eurasian individual.

The demographic histories inferred for the 11 Iberian lynx are quite concordant along the reconstructed period, and only start to diverge for most recent times, probably as a result of intrinsic model limitations (Figure S22). The demographic history of lynxes shows an early population decline starting at 600 kya in around N_e = 60,000 – approximately the same value estimated for the ancestral population size in the species divergence analyses (Section 9) –, and finishing approximately 200 kya in N_e = 10,000. A moderate population expansion during

the following 130 ky raised effective population size to ca. 15,000 and was followed by a second reduction in population size starting 60 kya before present to $N_e \approx 4{,}000$, a size that remained stable at least till 10 kya. Noticeably, the Eurasian lynx showed a parallel demographic history, albeit with effective population sizes consistently above the Iberian lynx. This difference cannot be attributed to the higher sequence coverage of the Eurasian individual, since the histories obtained for the reference Iberian individual (Candiles, A001) using two different depths are almost identical (Figure S23), what also suggests the lack of biases arising from missing heterozygotes due to lower coverage in the rest of Iberian samples [112].

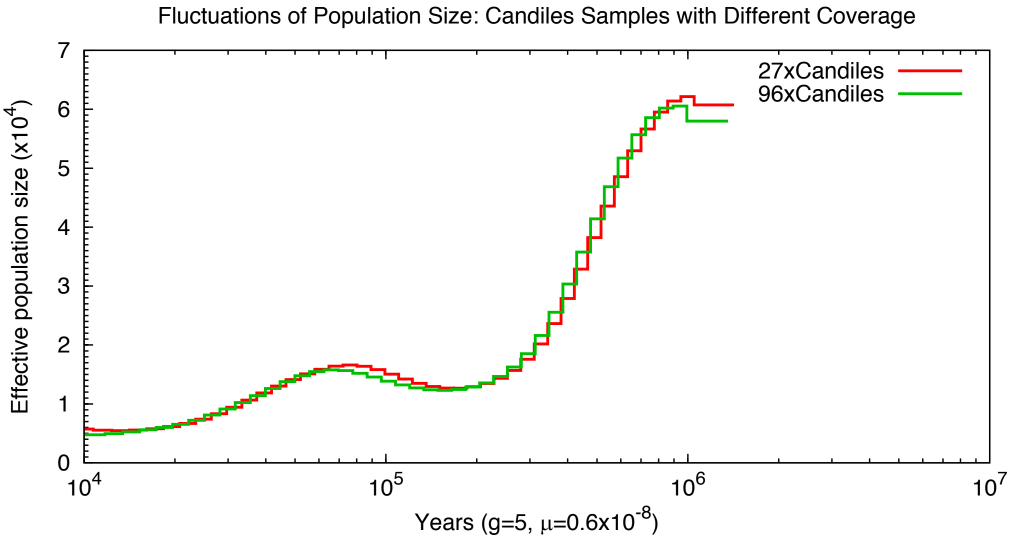

Figure S23. Effect of Sample Coverage in Demographic History Reconstruction. Below are the plots obtained using Candiles (A001) alignments with two different depths of coverage.

10.2 ∂a∂i

In order to reconstruct the more recent demographic history of the Iberian lynx we used a maximum likelihood inference method implemented in the software ∂a∂i [107]. This method implements a Wright-Fisher model through a diffusion approximation and calculates the expected allelic frequency spectrum (AFS) under the proposed demographic scenario. This methodology allows the selection of the best combination of parameters that maximize the probability of

observing the data. We started with the SNPs variants identified by *cortex_var* and excluded the SNPs in the scaffolds assigned to X- and mitochondrial chromosomes to ensure similar effective population size and unbiased demographic history. The Eurasian lynx sequences were used to infer the ancestral allelic state in the AFS. Thus, SNPs shared by the Iberian and the Eurasian lynx or that were not called in the latter were not considered. The ascertainment bias of the allelic state was statistically corrected following [113] by using the tri-nucleotide substitution matrix specific for carnivores (information kindly provided by D.G. Hwang) and neglecting SNPs with unknown flanking bases. We ended up with a total number of 1,005,086 SNPs to build up the AFS.

We tested different models with different number of parameters that could potentially accommodate the demographic history of the Iberian lynx during the last thousand millennia. Preliminary analyses considering the two Iberian lynx populations and implementing either an isolation-migration or a split-without-migration model did not reach convergence for any set of parameters tested. Similar log likelihood values were obtained for different sets of parameters tested, suggesting little statistical power with the models and data (R. Gutenkunst pers. comm.). This may be due to different reasons: the low number of samples (seven individuals from the Andújar population and four individuals from the Doñana population), the low genetic variation (especially in PND), and the conservative way of calling SNPs, all of which would hamper our ability to capture rare mutations (notably the singletons) that would capture recent demographic events [107]. Moreover, the strong drift suffered by the Doñana population during the recent times [114] may be confounding the results. For all these reasons we opted for reconstructing the demographic history of the Iberian lynx exclusively from the data on the Andújar (Sierra Morena) population, which is indeed the most representative of the recent historical population [114] and the species AFS.

We evaluated different demographic scenarios modelling either a single or two demographic changes, and allowing them to be instantaneous or exponential. We ran all models with multiple combinations of starting parameters and searching

widely in the likelihood surface to ensure that the chain was not stuck at a local maximum and that the maximum likelihood value was reached for every model. Two of the combinations of exponential and instantaneous changes (two exponential changes and one instantaneous followed by exponential) could not be optimized due to lack of convergence, so the final number of models compared was four (Figure S24). The Akaike Information Criterion (AIC) was used to select the best-fit model.

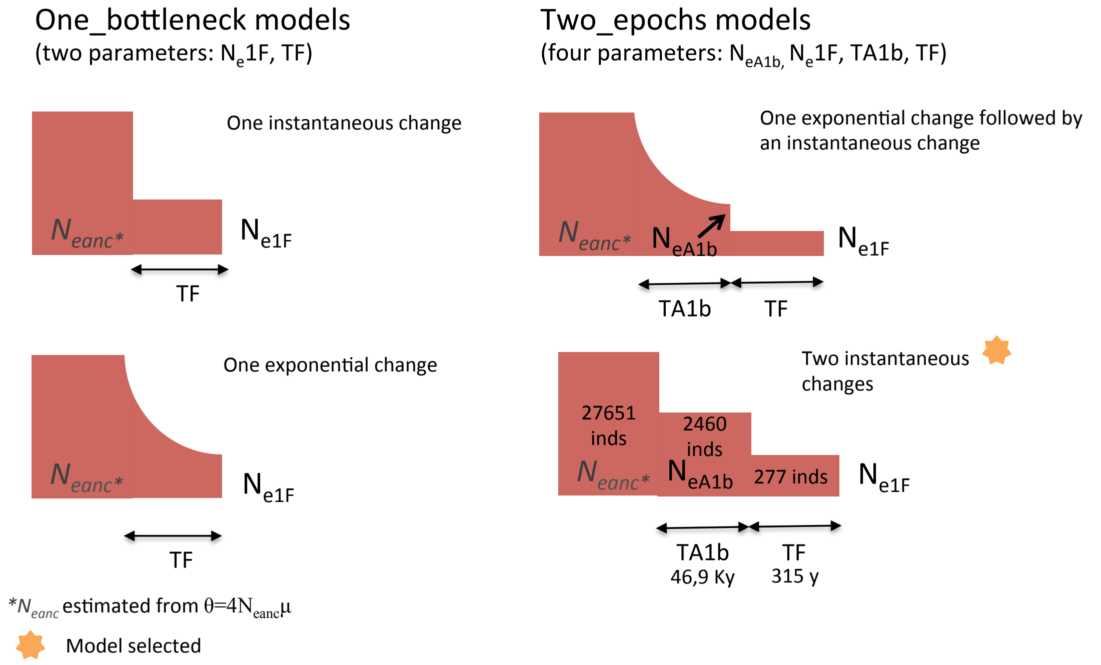

Figure S24. Demographic models evaluated for the Iberian lynx using *∂a∂i*

The parameters estimated in the models encompassed both changes in effective population size – scaled by $4N_{e(anc)}\mu$ – and time elapsed since the change – scaled by 2 N_{eanc} generations. Estimations performed under the one-demographic-change model recover two main parameters. The first is the ratio of current to ancestral population size (*Ne1F*), and the second is the time since the change in the demography, in our case the start of the population decline (*TF*). For the two-epochs model (with two demographic changes) four parameters were estimated. Two of them are the ratio of population size at one period to the previous (*NeA1b* and *NeA1F*) and the other two are the elapsed times between demographic changes, respectively (*TA1b* and *TF*) (Figure S24). To convert these values to

population sizes in individuals and time in years we assumed a rate of $0.6x10^{-8}$ mutations per site per generation, based and a generation time of five years, as used for PSMC reconstructions.

To estimate the 95% confidence intervals (95% CI) we performed 50 non-parametric bootstraps with the same number of loci than the dataset, resampling over scaffolds in order to reproduce the genome linkage structure as closely as possible. The selected model was run and optimized for each simulation. The ancestral population size, $N_{e(anc)}$, was estimated from the parameter theta in every simulation taking into consideration the different lengths of the sequence in each simulated dataset.

Table S20. Models considered in dadi. For each model we give the number of parameters (K), the AIC score and the AIC differences with respect to the one with the mínimum AIC (Δi).

Model	ln(L)	K	AIC	Δi
Two instantaneous changes	-3116.59	4	6241.18	0.00
One exponential change followed by an instantaneous change	-3292.73	4	6593.46	-352.28
One exponential change	-4583.89	2	9171.79	-2930.60
One instantaneous change	-4881.06	2	9766.12	-3524.94

The highest AIC was obtained for the two-epochs with both events being instantaneous declines of the population (Table S20). Assuming five years as the generation time for the Iberian lynx the observed allelic frequency spectrum in SMO is most consistent with a steep historical population decline around 46,941 ya (95% $CI_{bootstrap}$: 34,771-59,069) that reduced the effective population to 2,460 (95% $CI_{bootstrap}$: 1,804-3,113), slightly below one tenth of the original population, and a second decline reducing again the population to around one tenth, to 277 (95% $CI_{bootstrap}$: 199-360) occurring 315 ya (95% $CI_{bootstrap}$: 233-398) (Table S21).

Table S21. Demographic parameters estimated with *∂a∂i*. The selected model is the two_epochs model, with two instantaneous demographic changes. There are four free parameters. The ancestral population size, indicated with an asterisk in the column "Parameter symbols", was not a free parameter and was estimated from the theta value obtained from the model.

Parameters	Parameter symbols	Values	Confidence Interval (95%)	Units
Ancestral population size	$^*Ne_{Anc}$	27651	(19835, 35816)	individuals
Population size after the first bottleneck	Ne_{A1b}	2460	(1804, 3113)	individuals
Population size after the second bottleneck	Ne_{1F}	277	(199, 360)	individuals
Time of the first bottleneck	TA1b	46941	(34771, 59069)	years
Time of the second bottleneck	TF	315	(233, 398)	years

11 Karyotype

11.1 Cell culture, chromosomal preparations and G-banding

Dulbecco's Modified Eagle Medium (DMEM) supplemented with 25% fetal bovine serum, 2 mM L-glutamine, penicillin, streptomycin was used for culture of the primary Iberian lynx fibroblast cell line (Section 1.2.1). Colcemid (10 μg/ml) was added to the cell cultures for 4 hours. Cells were harvested at early passages and chromosomal preparations were obtained following standard protocols.

Metaphases were stained homogenously with Leishman solution for the analysis of diploid number (2n) and the number of autosomal chromosome arms (NFa), and then G-banded with Wright's stain following the methods described by [115] for karyotyping. For each staining, at least 30 metaphase spreads were analysed. The karyotype of the Iberian lynx was arranged following the cat chromosomal nomenclature [116].

11.2 Fluorescent *in situ* hybridization

For telomere detection fluorescence *in situ* hybridization (FISH) analysis was performed using a peptide nucleic acid (PNA) probe complementary to telomeres G-rich strand (TelC) (Panagene, Yuseong-gu, Daejeon, Korea)

according to manufacturer's protocol. Slides were dried at 67ºC for 20 min and rehydrated for 15 min in 1x PBS. Cell fixation was carried out in 4% formaldehyde in 1x PSB for 4 min. After two washes in 1x PBS, cytoplasm was removed by incubating slides 4 min in 0.005% pepsin in 0.01M HCl at 37ºC. Slides were then washed twice in PBS 1x for 3 min, dehydrated in 70%, 85% and 100% ethanol series and air-dried. 15 µl of 800 ng/ml TelC FAM-conjugated probe (Panagene, Yuseong-gu, Daejeon, Korea) in hybridization buffer (10 mM NaHPO4, 10 mM NaCl, 20 mM Tris, 70% formamide) were added on each slide. After denaturation for 5 min at 85ºC, slides were incubated for 1h 45 min at room temperature. Subsequently, slides were washed 20 min at 57ºC in PBST and 1 min at room temperature in 2x SSC 0.1% Tween-20. Before microscopic observation, nuclei were counterstained with DAPI (4',6-diamidino-2-phenylindole). Preparations were visualized using a Zeiss Axioskop epifluorescence microscope equipped with the appropriate filters and a charged coupled device camera (ProgRes® CS10plus, Jenoptik).

11.3 Iberian lynx karyotype and its comparison to Eurasian lynx and domestic cat

A G-banded karyotype of the Iberian lynx is illustrated in Figure S25A. The Iberian lynx has a diploid number (2n) of 38 chromosomes. The karyotype comprises 16 biarmed and 2 acrocentric pairs of chromosomes (Figure S25A-B), with an autosomal fundamental number (aFN) of 34. The biarmed can be divided into 11 submetacentrics (A1, A2, A3, B1, B2, B3, B4, D1, D2, D3, D4) and 5 pairs of metacentrics (C1, C2, E1, E2, E3). The X chromosome is a large submetacentric. Additionally, the FISH with a PNA telomeric probe indicated that, as is the case with other vertebrates, telomeric signals were detected at the ends of all chromosomes in the Iberian lynx (Figure S25C).

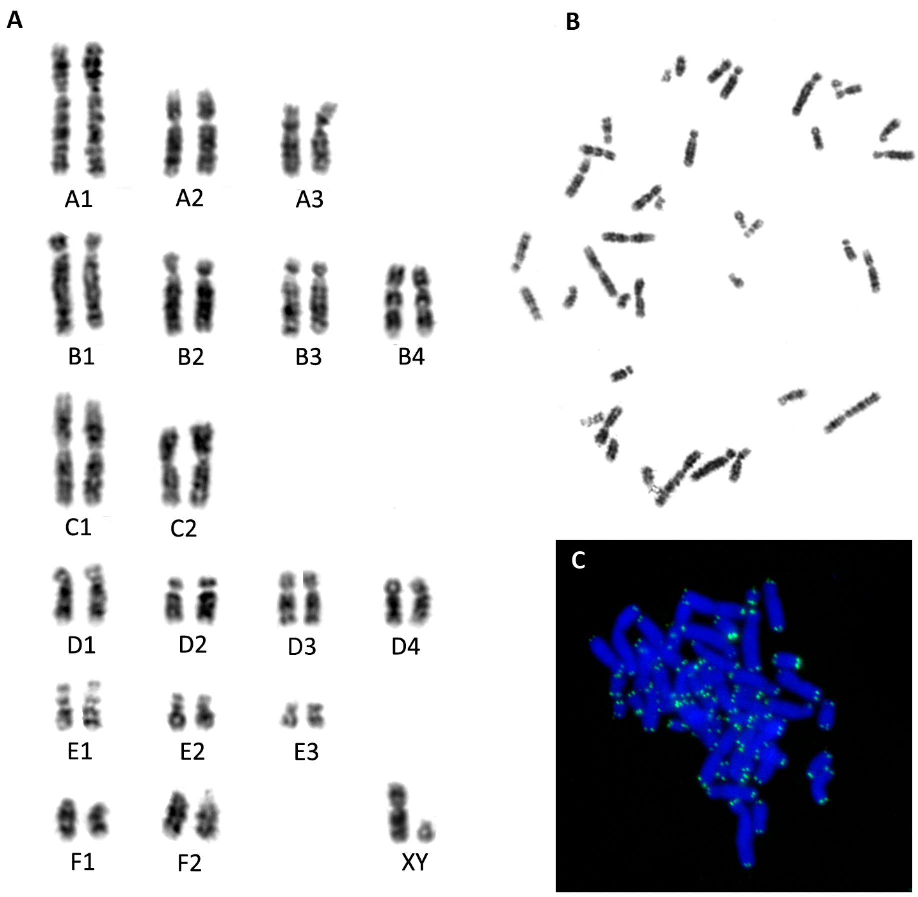

Figure S25. Chromosomal characterization of the Iberian lynx. **(A)** G-banded karyotype of the Iberian lynx (2n=38). **(B)** Example of a G-banded metaphase. **(C)** Distribution of telomeric repeats (TTAGGG) n on metaphase chromosomes of the Iberian lynx. The telomeric probe is depicted as green signals at the end of chromosomes.

Chromosomal evolution in Felidae is characterized by its conservativeness, as all species have retained (with very few modifications) the ancestral Carnivora karyotype [117]. This has been the case for the cat, tiger, lion, mainland clouded leopard, cheetah, jungle cat, puma, and caracal, all species characterized by the same diploid number (2n=38) and G-banding pattern [116, 118-120]. The present study confirms the same number of chromosomes for the Iberian lynx (Figure S25A). When comparing the Iberian lynx karyotype with those of the domestic cat and Eurasian lynx, we could observe that the G-banding patterns were well conserved between species allowing the confident assessment of homology among all chromosomes (Figure S26). In fact, no large-scale chromosomal rearrangements were detected between the three species, although we cannot discard the presence of fine-scale rearrangements (i.e., small

inversions/translocations/deletions) that scape the cytogenetic level of resolution.

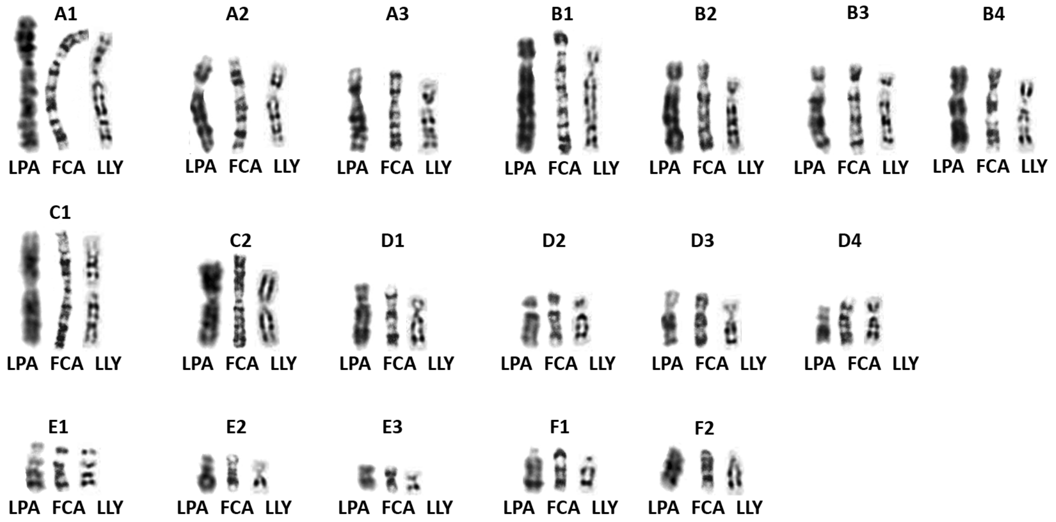

Figure S26. G-banded half karyotype comparison among the Iberian lynx (*Lynx pardinus*, LPA), the domestic cat (*Felis catus*, FCA) and the Eurasian lynx (*Lynx lynx*, LLY). FCA and LLY karyotype were adopted from Nie et al. (2012) [117] and O'Brien et al. (2006) [118], respectively.

12 Genome alignments and synteny analysis

12.1 Genome alignments

We built several pairwise whole-genome alignments using LAST v458 [121] using lynx, cat, tiger and dog genome data. In every case, genomes were soft-masked (lowercase), to reduce the number of alignment seeds while still allowing further extension of initial seed alignments over masked sequences. Masking was done with RepeatMasker for transposable elements and other repeats and with DustMasker for low complexity sequences. For simplicity, we removed unassigned cat scaffolds (e.g. chrA2_JH408375_random, chrUn_JH413745), hence relying on (almost) complete cat chromosomes as targets in the comparison.

To identify most likely orthologous genome segments, we selected only those local alignments that scored best among overlapping sets of alternative

alignments. We sorted LAST local alignments by score, and then, for each alignment, we checked whether it overlapped less than 50% with any of the previously accepted alignments, in which case it was accepted among the set of best hits. Out of 8,631,812 local alignments returned by LAST for the lynx-cat alignment, 689,518 were non-overlapping best hits and, of these, 37,521 were longer that 10,000 bps. The resulting alignments were the basis of several downstream analyses, being used to translate between lynx-scaffold and cat-chromosomes coordinates, to infer ancestral character states and polarize substitutions, and to identify chromosomal rearrangements.

Thanks to the high similarity between lynx, cat and tiger genomes, we were able to build virtual multiple alignments by bringing together lynx-cat and tiger-cat pairwise alignments. For the characterization of chromosomal rearrangements, lynx-dog alignments were also analysed.

12.2 Synteny between cat chromosomes and lynx scaffolds

We analysed the synteny between cat and lynx with several purposes: 1) to identify potential chromosomal rearrangements and inversions; 2) to define orthologous regions that would allow mapping of lynx scaffolds onto cat chromosomes (which would be the basis of our genomic population analysis); and 3), to identify specific transposable element insertions.

For each scaffold, we saved all the best-hit alignments of length >1,000 bp, and ordered these hits based on the corresponding cat genome coordinates. Then, we merged with bedtools [122] all those alignments that were less that 20Kb apart and on the same strand. We retained only those chained alignments that were at least 15 Kbp long and for which at least 40% of the sites of that region were aligned. Finally, we explored the resulting chained alignments to detect inversions and inter/intra-chromosomal rearrangements. To polarize the potential rearrangements, i.e. to label them as lynx- or cat-specific, we used the dog genome as out-group. The synteny analysis between lynx and dog required some slightly different parameters to account for the larger divergence.

Each potential rearrangement between lynx and cat was carefully inspected in the out-group. As a filter to eliminate rearrangements that may be assembly

artefacts, we required that at least one scaffold derived from fosmid sequencing crossed the predicted breakpoint. This final filtering was done manually using our genomic browser.

We identified 15 potential rearrangements, 5 intra-chromosomal and 10 inter-chromosomal. Of these, 6 corresponded to cat, 6 to lynx, and three were uncertain. Up to 37 local inversions were identified, 17 corresponding to cat, and 20 to lynx. The final list can be found in the accompanying table (Additional file 2, Datasheet S3).

In order to further validate the inferred rearrangements we tested the scaffold integrity by performing long-range PCRs with primers flanking the inferred breakpoint on the reference genome, followed by Sanger sequencing of the amplicons obtained. Out of 15 potential rearrangements tested, eight were empirically validated by this approach (Table S22).

Table S22. Potential rearrangements identified after the alignment of lynx scaffolds to cat chromosomes and their support from fosmid sequences or PCR amplification and Sanger sequencing.

cat breakpoint coords	lynx breakpoint coords	fosmid support	happened in	Empirically validated
chrX: 121299653, chrX: 123808755	lp23.s00225: 65220-69872	Weak	lynx	Yes
chrA1: 89934990, chrD4: 21523389	lp23.s00237: 50503-52502	Yes	lynx	Yes
chrE2: 8728442, chrE2: 10739143	lp23.s05401: 64744-67893	Yes/Weak	?	No
chrC2: 74597169, chrC2: 77597169	lp23.s10737: 78469-80793	Yes	?	No
chrA2: 51863039, chrC1: 152332927	lp23.s10856: 31199-32137	No	cat?	Yes
chrB4: 72340843, chrB4: 72915652	lp23.s15749: 326069-326983	Yes	lynx	Yes
chrC1: 21866728, chrC1: 21910373	lp23.s15845: 69115-70001	Yes	?	?
chrA1: 184021989, chrA1: 174341697	lp23.s20850: 1899562-1909371	Yes	cat	No
chrD1: 8038180, chrB3: 109752981	lp23.s20857: 119905-120210	Yes	cat	Yes
chrD1: 51701, chrA2: 167831369	lp23.s20915: 48478-53814	Yes	cat	No
chrE2: 10605295, chrE2: 11148490	lp23.s26299: 111002-113005	Yes	cat	No
chrB1: 104085575,	lp23.s31299: 198248-	Yes	lynx	Yes

chrB1: 104398572	199109			
chrE1: 4380477, chrE1: 4697195	lp23.s31503: 214020-214429	Yes	lynx	Yes
chrB1: 191984078, chrB1: 203274234	lp23.s36495: 2743278-2745170	Yes	lynx	No
chrB2: 44436909, chrF2: 76959744	lp23.s36540: 450100-451345	Yes	cat	Yes

13 Phylogenomics

13.1 Lynx phylome reconstruction

The Iberian lynx phylome (i.e. the complete collection of phylogenetic trees for each gene encoded in the genome) was reconstructed using the PhylomeDB pipeline [123]. Briefly, for each gene encoded in *Lynx pardinus*, a Smith-Waterman search was performed against a database containing the proteomes of the 15 species considered (Table S23). We used an e-value threshold of 1e-05 and required a continuous overlap of 50% over the query sequence. The number of hits were limited to the closest 150 homologs per gene. A multiple sequence alignment (MSA) was then reconstructed using three different programs (MUSCLE [124], MAFFT [125] and KALIGN [126]) in forward and reverse directions [127]. These six alignments were combined using M-COFFEE [128]. Considering the high levels of similarity across orthologs in the felid species we decided to back-translate the protein alignments into their respective codons, using the coding sequences. trimAl [129] was first used to trim the protein alignments (consistency cut-off of 0.16667 and -gt >0.1). Clean alignments were then back-translated from protein sequences to their corresponding codons prior to phylogenetic tree reconstruction. ML trees were reconstructed based on the codon alignments using codonPhyML v1.0 [130] using GY as codon substitution model, and F3X4 as model for defining the codon frequency from the alignment. Branch support was computed using an aLRT (approximate likelihood ratio test) parametric test based on a chi-square distribution. In all cases, a discrete gamma-distribution with three rate categories was used, estimating the gamma parameter from the data.

Table S23. List of species used in the phylome reconstruction. First column indicates taxa id, second column contains the species name and the third shows the source for the protein and the coding DNA sequences. Asterisks indicate species used in the expanded phylome.

Taxa ID	Species name	Data Source
9606	*Homo sapiens*	Ensembl v73
9615	*Canis lupus familiaris*	Ensembl v73
9646	*Ailuropoda melanoleuca*	Ensembl v73
9685	*Felis catus*	Ensembl v73
9785	*Loxodonta africana*	Ensembl v69
9796	*Equus ferus caballus*	Ensembl v73
9823	*Sus scrofa*	Ensembl v73
9913	*Bos taurus*	Ensembl v73
10090	*Mus musculus*	Ensembl v73
10116	*Rattus norvegicus*	Ensembl v73
13125	*Lynx lynx*	Lynx sequencing project
13616	*Monodelphis domestica*	Ensembl v73
59463	*Myotis lucifugus*	Ensembl v73
74533	*Panthera tigris altaica*	http://tigergenome.org/
191816	*Lynx pardinus*	Lynx sequencing project
9544*	*Macaca mulatta*	Quest For Orthologs: r2011/02
9598*	*Pan troglodytes*	Ensembl v77

13.2 Prediction of orthology and paralogy relationships

Orthology and paralogy relations between lynx and the other species considered were predicted based on phylogenetic evidence from the lynx phylome. For each tree, ETEv2 [131] was used to infer duplication and speciation relationships using a species overlap approach and a species overlap score of 0. Briefly, the method, as described in Huerta-Cepas et al. [132], considers a node as a speciation node when there are no overlapping species at any of child partitions, and a duplication node otherwise. Orthologs are inferred according to the original orthology definition, i.e orthologous genes are those whose last common ancestor is represented by a speciation event and paralogous genes those that diverge from duplication events [133]. All orthology and paralogy relationships are available through PhylomeDB [134].

13.3 Gene duplications

The phylome was scanned to detect genes that had undergone duplications in different specific lineages and the relative age of the duplication was estimated

by phylostratigraphy, i.e. by using information on the species that diverged prior and after the duplication node [135]. Trees that contained putative transposable elements were not considered. Gene Ontology (GO) enrichment analysis was performed using FatiGO [136] by comparing annotations of the proteins related to gene duplications occurring at a given age against all the other proteins encoded in the lynx genome (Additional file 2, Datasheet S4).

13.4 Detection of putative pseudogenized genes

1,731 proteins annotated in the cat genome had no homologs to lynx in the phylome. Most differences are likely resulting from different false positives and false negatives in the two independent annotations. To find putative pseudogenized genes in the lynx, we applied several filters. First the filtering threshold was relaxed so that homologs with only 20% overlap were accepted. Those genes that still had no homologs in lynx but had homologs in at least four additional species included in the analyses were searched against the lynx genome using tBlastn [96]. Cat proteins with significant (e-value < 10^{-5}) hits in the lynx genome aligning over 30% of their length were selected for further inspection. The genomic region determined by the blast search was extended by 10,000 nucleotides at either side and exonerate-based gene prediction [137] was performed on the region using the cat protein as a seed. 85 predictions interrupted by stop codons were considered as putative pseudogenes (Additional file 2, Datasheet S5). This set can be considered a first step, but further research is needed to identify bona-fide pseudogenized genes (i.e. confirming stop-codons). No functional GO term was found to be enriched among these genes.

13.5 Species tree reconstruction

1,470 genes that had one-to-one orthologs in each of the species considered were selected and their trimmed alignments, as constructed in the phylome, were concatenated. The alignment was further trimmed to delete positions with more than 50% gaps using trimAl v1.3 [129]. The final alignment contained 2,795,619 nucleotidic positions. RAxML version 7.2.6 [138] was used to reconstruct the maximum likelihood species tree (model PROTGAMMALG).

Bootstrap supports were calculated by creating 100 alignments using PHYLIP's SeqBoot [139]. For each alignment, a tree was reconstructed using RAxML as explained before. A consensus tree was finally derived using the consensus tool implemented in PHYLIP (Figure S27).

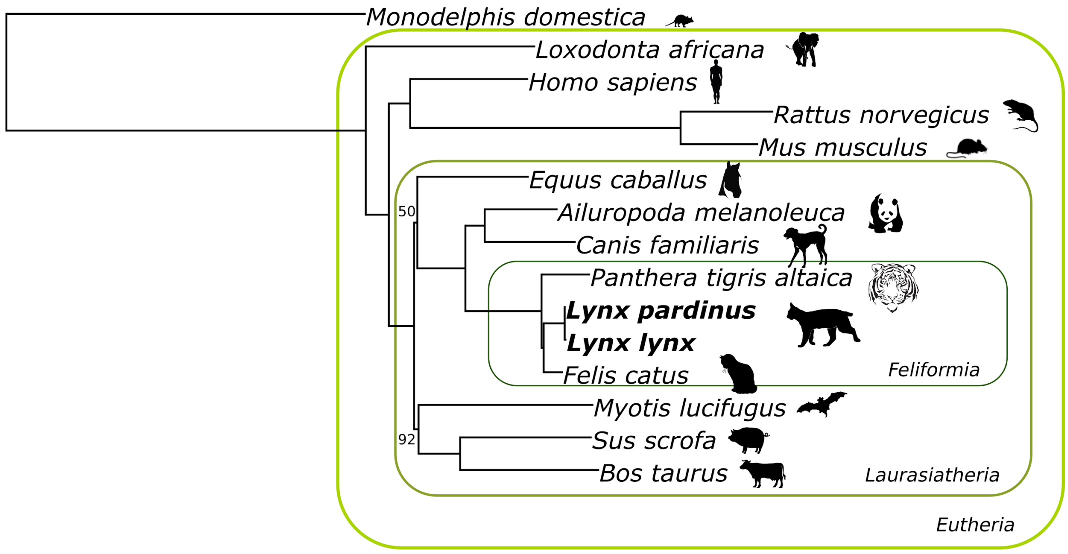

Figure S27. Species tree obtained from the concatenation of 1,470 widespread single gene trees. Species names in bold indicate genomes that have been sequenced in this study. Bootstraps below 100 are indicated in the tree.

A super-tree was also inferred from all trees in the second phylome (19,843 trees) by using a Gene Tree Parsimony approach as implemented in the dup-tree algorithm [140] (Figure S28). This procedure finds the species topology that minimizes the number of duplications across the collection of gene family trees, i.e. the phylome.

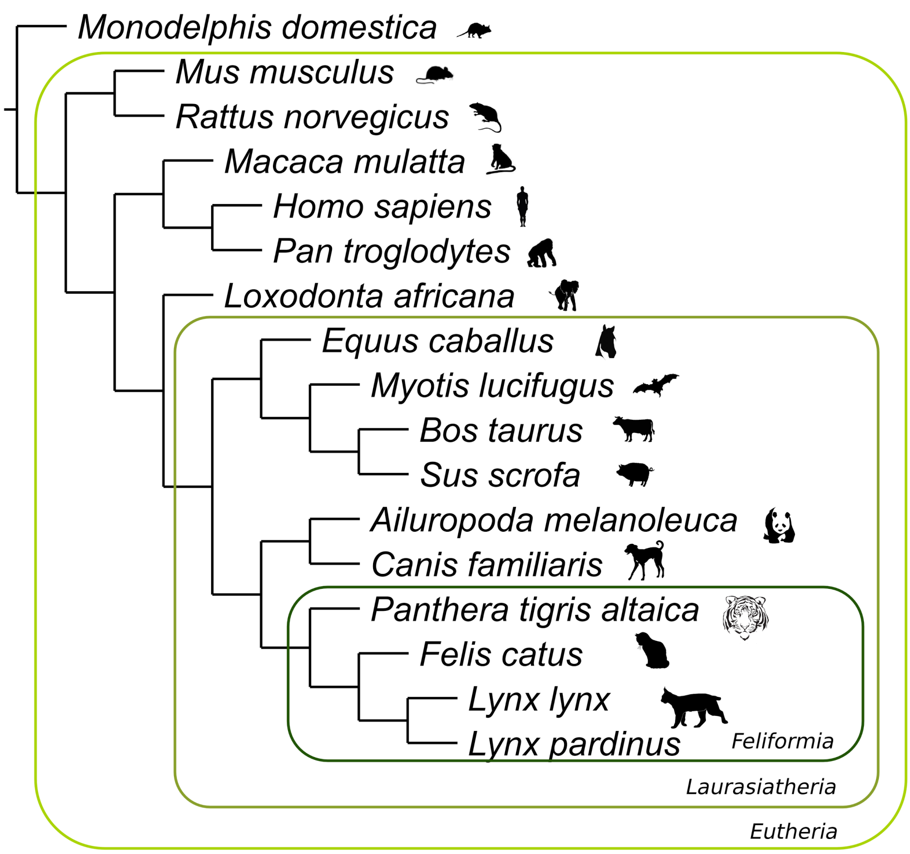

Figure S28. Species tree obtained derived from the combination of 19,843 single gene trees reconstructed for the second phylome which includes two additional hominids species. This tree does not have either branch support or length since the duptree algorithm found a unique tree which minimizes the total number of duplications.

13.6 Divergence time estimation

The first reconstructed species tree was used to estimate divergence times between several lineages. Calibration points were taken from the TimeTree website [141]. The divergence between human and cat was set at 94.2 MyA (average obtained from 35 studies, standard deviation = 19.8 MyA), between cat and dog at 55.1 MyA (average obtained from 16 studies, standard deviation = 6.1 MyA) and between pig and cow at 63.1 MyA (average obtained from 12 studies, standard deviation = 6.9 MyA). PL-r8s [142] was used to estimate the divergence time between cat and lynx and between cat and tiger (Figure S29). The smoothing parameter was estimated using cross-validation.

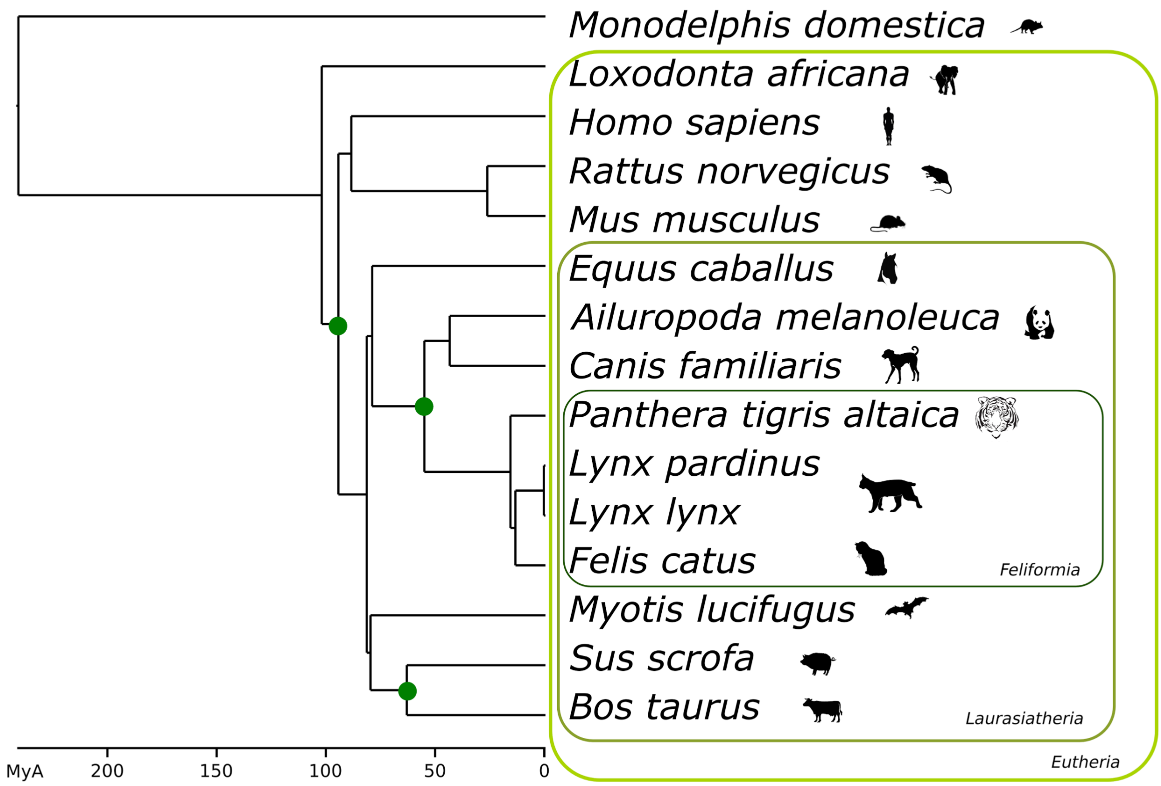

Figure S29. Chronogram depicting divergence times of the species included in the analysis. Green dots represent calibration points.

13.7 Estimation of *dN/dS* ratios

8,117 sets of one-to-one orthologs comprising proteins from five carnivore species (*L. pardinus*, *L. lynx*, *F. catus*, *P. tigris* and *C. familiaris*), three hominids (*H. sapiens*, *P. troglodytes* and *M. mulatta*), and two rodents (*M. musculus* and *R. norvegicus*) were used to estimate the *dN/dS* ratio for different branches of the extended reference species tree (Figure 3C, main text). We expected to have a high degree of misaligned residues considering the variable genome-data quality among the species used and the heuristic nature of the methods to reconstruct multiple sequence alignments. To reduce the impact of such errors in the computation of the dN/dS ratio, the trimmed alignments used to reconstruct single gene trees were further scanned. Firstly, codon columns containing gaps were removed. Secondly, we scanned the corresponding translated alignments looking for columns with at least one amino acid replacement, and only those surrounded by two previous and two posterior fully conserved sites were retained. An automated script

(selective_trimming_for_dNdS_analyses.based_neighbours.py) implementing this approximation is available at the official trimAl repository in GitHub: https://github.com/scapella/trimal. Resulting alignments were concatenated and the number of nonsynonymous substitutions per nonsynonymous site (*dN*), synonymous substitutions per synonymous site (*dS*), and the corresponding *dN/dS* ratio (ω) were estimated for each branch in the reference species tree using the ML method implemented in the CodeML program of PAML version 4.4 [143]. For this analysis, we used a 1) fixed topology according to the extended species tree, 2) F3X4 as model of codons frequency, and 3) a free-omega model (model = 1) so an independent ratio for each branch is assumed.

13.8 Phylogenomics of Felidae

We generated a genome wide alignment between seven felid species (domestic cat, Iberian lynx, Eurasian lynx, tiger, lion, snow leopard, and cheetah) by mapping Illumina short reads from each wild species ([12, 105], this study) to the repeat-masked domestic cat genome assembly [144] using BWA [108] with the following settings: bwa aln –n 0.08 [145]. We used SAMtools [146] and *ANGSD* [147] to call raw single nucleotide variants (SNV) and filtered them based on mapping quality (>30). We excluded variants from regions with read depth variation over 150% or below 50% of the genome-wide average. We further filtered the whole genome alignment by removing sites identified by CNVnator [148] as duplicated or deleted in at least one of the lineages considered, or that were located on the chromosomally unassigned scaffolds. The resulting 2-Gb alignment was analyzed by *Saguaro*, an HMM based partitioning approach that identifies chromosomal regions with discrete phylogenetic signals that are different from the background signal [149] (Additional file 2, Datasheet S6). We then constructed a maximum likelihood tree in RAxML [150] using a GTR+gamma model of sequence evolution, based on partitions matching the species tree topology [144], which was also identified as the most frequent topology (Table S24; Figure S30). We used this topology to estimate divergence times from whole genome alignment with *MCMCtree* [143] using an auto-correlated rates model with soft calibrations, node calibrations following Li *et al.*

[144] (Table S25 and Figure S30). The topology and time trees are concordant with previous studies of felid phylogeny [104, 144]. The initial divergence time estimate for the Iberian-Eurasian lynx is 1.5 My (95% credibility interval=900 Kya—2.2 Mya), which is younger than most felid sister species pairs and consistent with the scenario described here of a recent divergence followed by gene flow (Figure 1, main text).

Table S24. Topology frequency information based on sliding window analysis. The ten most frequent topologies reconstructed from *Saguaro* partions are shown.

Raxml_topology	Frequecy	Topology
1	1423	(PLE,((PUN,PTI),((AJU,FCA),(LYP,LLY))));
2	1299	(PLE,((PUN,PTI),((FCA,(LYP,LLY)),AJU)));
3	708	(PLE,((PUN,PTI),((AJU,(LYP,LLY)),FCA)));
4	210	(PLE,(PUN,(PTI,((AJU,FCA),(LYP,LLY)))));
5	181	(PLE,(PTI,(PUN,((AJU,FCA),(LYP,LLY)))));
6	115	(PLE,(PUN,(PTI,((FCA,(LYP,LLY)),AJU))));
7	109	(PLE,(PTI,(PUN,((FCA,(LYP,LLY)),AJU))));
8	102	(PLE,(PTI,(PUN,((AJU,(LYP,LLY)),FCA))));
9	81	(PLE,(PUN,(PTI,((AJU,(LYP,LLY)),FCA))));
10	5	(PLE,(PUN,((AJU,FCA),(PTI,(LYP,LLY)))));

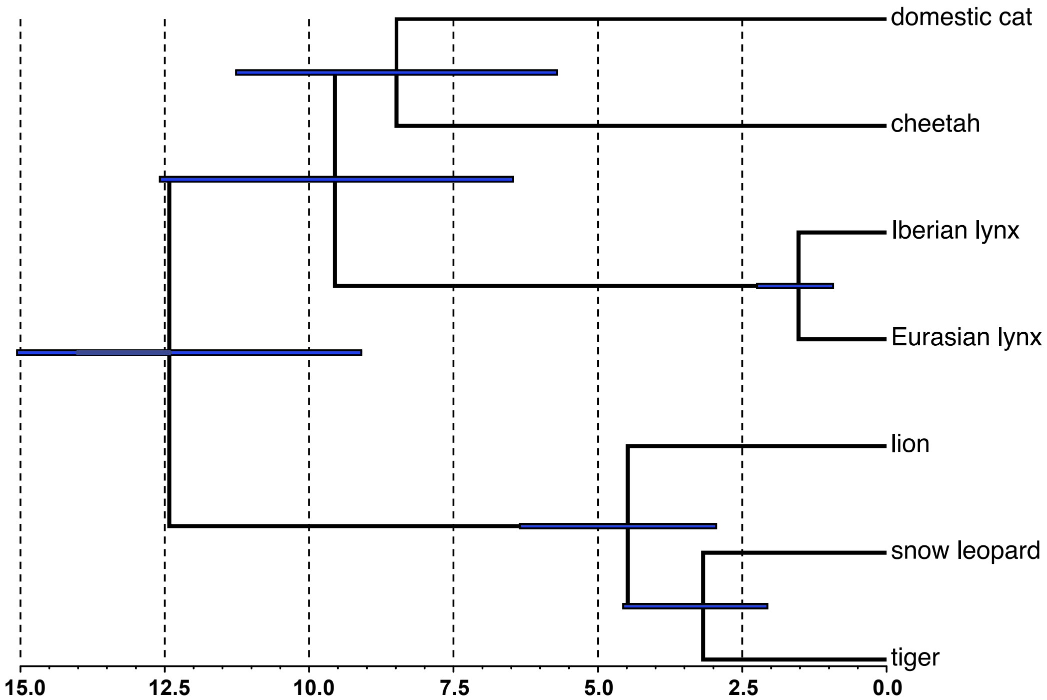

Figure S30. Dated Felidae phylogeny based on whole genome alignments. The topology is the most supported among those reconstructed from *Saguaro* partitions and is concordant with previous phylogenetic reconstructions. Bars on internal nodes represent the 95% confidence intervals for node dates (Table S25). Axis represents time in million years units.

Table S25. Divergence time estimates for nodes within Felidae calculated on windows of the genome alignment that reconstruct the species tree topology (Figure S30).

Node	Time (Mya)	95%_low	95%_high
tiger/snow leopard	3.18	2.06	4.56
lion/(tiger+snow leopard)	4.49	2.95	6.36
domestic cat/cheetah	8.49	5.71	11.26
Iberian lynx/Eurasian lynx	1.52	0.93	2.24
Lynx(domestic cat+cheetah)	9.55	6.47	12.59
Base of Felidae	12.42	9.10	15.06

14 Positive selection

We looked for signatures of positive selection in the lynx lineage using a set of one-to-one orthologs generated in the phylogenomics analyses (Section 13). We selected 8 different species: *Panthera tigris, Felis catus, Lynx lynx, Lynx pardinus*, *Ailuropoda melanoleuca*, *Canis lupus familiaris, Homo sapiens* and *Mus musculus*. The set comprised 9,695 genes. We performed multiple sequence alignments with the software PRANK [151] which has been shown to be particularly accurate at handling insertions and deletions, resulting in a lower number of false positives in positive selection tests [152, 153]. We conducted a branch-site test of positive selection (PS) [143] using information from Timetree (www.timetree.org) for the input tree. This test is based on the detection of codons with an excess of non-synonymous substitutions in particular branches. It has a reasonable statistical power and low false-positive rates but it is also extremely sensitive to alignment errors [154]. We filtered out cases with more than one site with a probability of being under positive selection higher than 0.99 by the Bayes empirical Bayes (BEB) approach, as they typically corresponded to non-homologous stretches [153]. Internal branches were barely affected by this filtering, since they are more resilient to this kind of errors. We manually validated 100 lynx positive selection candidates (96 for *Lynx sp.* and 4 for *Lynx lynx*; Additional file 2, Datasheet S7).

We used Gitools [155] and annotations from Ensembl version 73 [156] to perform an enrichment analysis in the set of positively selected genes, obtaining

no significant results for either Gene Ontology terms or KEGG pathways (p-value > 0.05, False Discovery Rate correction BH [157]). We also collected lists of genes related to immune system or audition from NCBI genes but could see no general enrichment either.

However, inspection of the list of 100 candidates revealed that 21 of the validated genes were related to known human phenotypes, mostly diseases or syndromes recorded in OMIM (Additional file 2, Datasheet S7). We also found structures in Protein Data Bank (PDB) for another 22 of those 100 cases. For example, the gene LYPA23A017274P1 (DARS) is an extremely conserved protein where we have a histidine (H) in the lynx lineage while all the felids present an arginine (R) and the rest of mammals a glutamine (Q).

Sensory perception is thought to be particularly important for cats [158] and indeed we found two genes related to hearing in the list of positive selection candidates for the lynx branches: CACNA1D (LYPA23A015140P1) or MYO1F (LYPA23A022113P1). Mutations in these genes have been associated with deafness or hearing loss in humans [159, 160]. In addition, two vision-related genes were also under positive selection in lynxes: OPTC (LYPA23A008195P1) and GUCY2F (LYPA23A015393P1) [161].

15 Transposable elements dynamics

Transposable elements (TEs) are key players in the evolution of eukaryotic (and bacterial) genomes, shaping the organization and restructuration of chromosomes and acting as potent mutators [162, 163]. Recombination dynamics and TE accumulation are related to each other because selection prevents ectopic recombination and at the same time is weaker at regions of low recombination rates. Consequently, TEs are expected to accumulate at regions of low recombination [164], as reported in *Drosophila [165]*. Although the argument is appealing and intuitive, opposing results have been reported for *C. elegans* [166]. Differences in the type of reproductive system (high inbreeding in *C. elegans*) and in the effective population sizes (high in *Drosophila*) have been proposed to account for the reported discrepancies [167]. Indeed, bottlenecks and inbreeding leads to high levels of homozygosity, reducing the effective rate

of recombination [168] and the strength of selection, so we could expect TE activity to increase in bottlenecked or inbred populations. However, the impact of inbreeding on TE accumulation is not clear yet, as contradictory results have been reported for different species [169, 170] and simulations [167, 171, 172]. Here we analyse and compare data from cat, tiger, and two lynx species (Iberian and Eurasian) to assess the impact of demographic bottlenecks, inbreeding, and reduced strength of purifying selection on TE dynamics.

15.1 Identification of species-specific SINE and LINE insertions from pairwise genome alignments of tiger, cat and lynx

TEs were annotated using RepeatMasker version open-4.0.1 [17], using the library of *Felis catus* and the sensitive search strategy (Section 3.3). Genomes of lynx, cat and tiger were pairwise aligned using LAST [121] with the aim of identifying orthologous regions between them (Section 12). We analyzed unambiguously aligned regions for each pair of species (lynx-cat, lynx-tiger, cat-tiger) to identify strongly supported gaps. Every gap in which a particular TE covered at least 95% of the gap, 99% of that TE was within the gap, and in which target-site duplications (TSDs) were detected at each gap boundary, was considered as a species-specific TE insertion. TSD were defined by obtaining -25/+15 and -15/+25 bps around the start and end site coordinates of the TE, respectively. Then, both sequences were compared to each other with BLAST and we required that L*P/100 was greater than 6, where L is the length of the alignment and P the percentage of identity. This procedure allowed the identification of short interspersed element (SINE) and long interspersed element (LINE) insertions, as they leave clear TSDs of size ~20 bps.

Based on the analysis of pairwise genome alignments we defined 7 sets of TE insertions: L_T, L_C, C_L, C_T, T_L, T_C, and LC_T, where L, C, and T indicate lynx, cat, and tiger, respectively. The first letter identifies the species in which the TE insertion was detected, whereas the suffix identifies the reference species in the pairwise genome alignment. The set LC_T corresponds to C_T minus C_L, and was used to represent those TEs that inserted in the last common ancestor of lynx and cat after the divergence with tiger.

When we compared lynx and cat, we identified 15,097 (366) and 10,687 (298) specific SINE (LINE) insertions in each species, respectively (sets L_C and C_L). The lynx-tiger comparison yielded even larger differences: 16,665 SINE (466 LINE) insertions in lynx (L_T) compared to 6,876 (391) insertions in tiger (T_L; Table S26). Hence, lynx shows higher activity of both SINE and LINE elements than cat and tiger. Active SINE elements in lynx correspond to types SINEC_Fc (14,196 lynx-specific insertions compared to cat), SINEC_Fc2 (866) and SINEC_Fc3 (33) types, whereas active LINEs belong to L1_Fc (316), L1-2_Fc (38), L1_Carn2 (2), and L1_Felid (2) types (Table S27).

Table S26. Number of species-specific/shared TEs (SINEs and LINEs) as estimated from the analysis of pairwise genome alignments. Each dataset identifies a target (first letter) and reference (subscript) species, where L, C and T stand for lynx, cat and tiger, respectively.

Set of TEs	SINEs		LINEs	
	TE Insertions	Shared TEs	TE Insertions	Shared TEs
L_C	15,097	1,435,628	366	1,192,406
C_L	10,687	1,442,499	298	1,198,474
L_T	16,665	1,449,061	466	1,211,737
T_L	6,876	1,449,057	391	1,217,480
T_C	5,765	1,398,763	380	1,176,509
C_T	9,955	1,405,174	308	1,175,636

To analyze the accumulation of TEs along the branches of the tree that relates lynx, cat and tiger, we relied on the pairwise comparisons between lynx and cat and between tiger and cat, i.e. on sets L_C, C_L, LC_T, C_T, and T_C. Every TE insertion was mapped onto the cat genome to analyze the patterns of insertion within genes (see below). Results shown in the main figure 2 represent the accumulation of TEs along the lineages of lynx, cat, and tiger since the last common ancestor of the three.

We also evaluated the reliability of our approach to identify TE insertions. One would expect that TEs shared between lynx and tiger should also be present in cat. Similarly, one would expect that lynx- or cat-specific TE insertions should not appear as shared with tiger. A large departure of this expected pattern could indicate some problem with the methodology. However, this was not the case as we found that almost all of the lynx TEs that are shared with tiger (i.e. they are part of an alignment of orthologous regions) are also shared with cat (2,940,547

out 2,970,782, 98.98%), whereas, very few lynx-specific TEs identified in the cat-lynx comparison are shared with tiger (239 out of 15,463, 1.49%). By comparing the set of lynx specific TE insertions with respect to tiger (17,133) against the set of TEs shared by lynx and cat, we were able to identify 1,887 TEs that most likely inserted in an ancestor of cat and lynx not shared with tiger.

Table S27. Activity of TEs in lynx, cat and tiger as estimated from pairwise genome alignment comparisons. Background frequencies are based on the number of occurrences in the genome.

	Lynx-tiger comparison (L_T and T_L sets)				Lynx-tiger comparison (L_C and C_L sets)			
TE	Lynx-specific TEs	Genomic background (%)	Tiger-specific TEs	Genomic background (%)	Lynx-specific TEs	Genomic background (%)	Cat-specific TEs	Genomic background (%)
SINEC_Fc	14,957	153,083 (4.46%)	3,538	46,631 (1.39%)	14,196	153,083 (4.46%)	9,888	137,863 (4.07%)
SINEC_Fc2	1,679	573,169 (16.70%)	3,238	589,658 (17.63%)	866	573,169 (16.70%)	778	564,075 (16.63%)
L1_Fc	413	64,352 (1.87%)	335	51,650 (1.54%)	316	64,352 (1.87%)	253	48,369 (1.43%)
L1-2_Fc	45	43,692 (1.27%)	49	33,680 (1.01%)	38	43,692 (1.27%)	39	33,979 (1.00%)
SINEC_Fc3	29	383,230 (11.16%)	9	382,478 (11.44%)	33	383,230 (11.16%)	21	377,395 (11.13%)
L1_Felid	4	7,808 (0.23%)	2	7,812 (0.23%)	--	--	--	--
SINE_LR	--	--	76	1,994 (0.06%)	--	--	--	--
SINE_OM	--	--	5	786 (0.02%)	--	--	--	--
L1_Carn2	--	--	--	--	5	62,017 (1.81%)	2	62,715 (1.85%)
L1_Felid	--	--	--	--	2	7,808 (0.23%)	1	7,950 (0.23%)

15.2 Identification of species-specific endogenous retroviruses insertions

To determine the activity of endogenous retroviruses (ERV) in lynx, we relied on a combined approach based on synteny analyses and phylogenetic reconstruction. First, we annotated the set of endogenous retroviruses in lynx, cat and tiger. To reconstruct full ERVs we post-processed RepeatMasker results and searched for pairs of long-terminal repeats (LTRs) that: 1) were of same type; 2) were on same strand; and 3) at least 50% of the LTR-enclosed sequence

corresponded to ERV fragments of the same family and orientation. Finally, when ERV candidates overlapped, we retained only one of them. By doing this, we were able to reconstruct 1,776, 1,895 and 1,940 full-ERV candidates in lynx, cat and tiger, respectively. We built a phylogenetic tree for all these ERVs using the BioNJ method [173]. Given the difficulties in aligning ERVs (they are large, fragmented, and display different genetic organizations), evolutionary distances were estimated by averaging the percentage of identities of all BLAST [46] non-overlapping local alignments. Percentages of identities (observed distances) were corrected with the Jukes-Cantor evolutionary model [174] and, when no similarity existed we arbitrarily set the distance to 0.5. The resulting tree was midpoint-rooted (Figure S31) and then traversed from root to tips to identify monophyletic groups constituted by two or more ERV from the same species, i.e. likely species-specific ERV expansions. We found 1, 14, and 30 monophyletic clusters in which only lynx, cat, and tiger ERVs were present, respectively. These clusters correspond to 2, 70, 95 ERVs in lynx, cat, and tiger respectively. The three largest clusters correspond to cat and contain 19, 14 and 11 ERVs. The first cluster contains ERVs of type ERV1-1_FCa, whereas the second and third contain ERV1-3_FCa. In tiger, we observed expansions of the same types of ERVs found in cat, although tiger-expanded ERVs differ in that they use to have LTR2_FC as LTRs. Overall, most expansions, including the two lynx-specific ERVs, correspond to the ERV1 class, although one ERV2 cluster was identified in each cat (ERV2-4_FCa-LTR/ERV2-4_FCa-I, 2 members) and tiger (LTR1_FC/ERV2-3_FC-I, 6 members). According to the phylogenetic tree, in most cases ERV expansions probably arose by the insertion of a new RV, although in some others the expansion seems to be related to the reactivation of an already inserted ERV. To summarize, our results indicate that, in contrast to cat and tiger, in lynx there has been null or very low activity of ERVs.

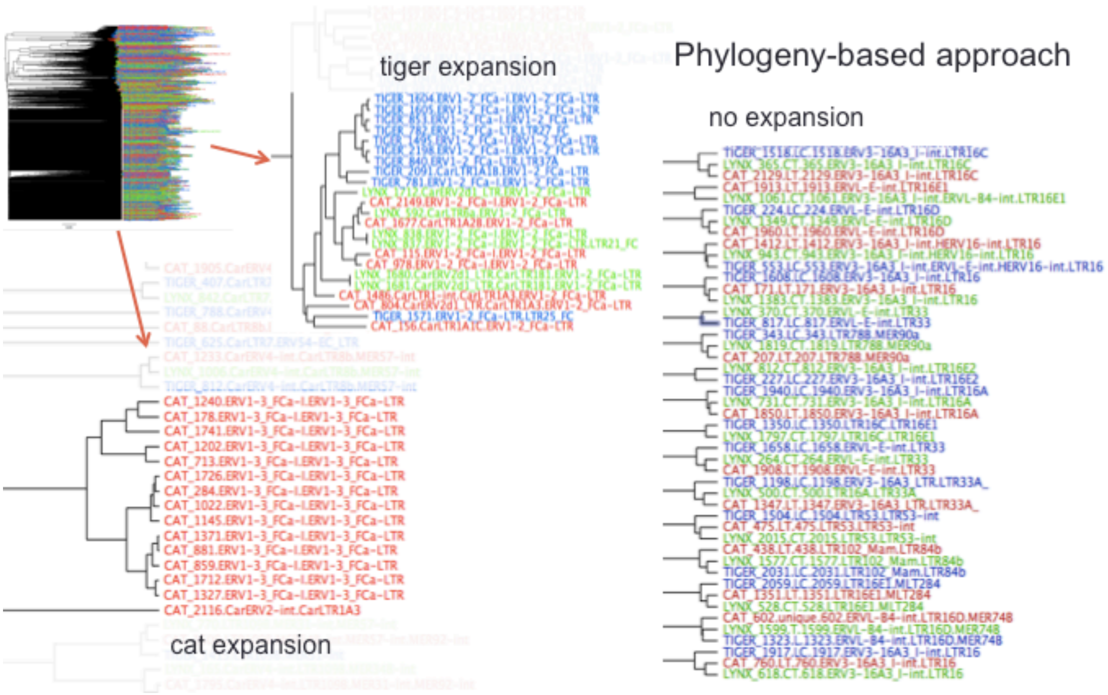

Figure S31. Examples of species-specific ERV expansions identified as single-species monophyletic clusters in phylogenetic analyses.

15.3 Analyses of TE insertions within genes

To do the analysis of orientation and proportion of within-gene TE insertions, we relied on the sets C_L, L_C, T_C, and LC_T. We translated lynx gene coordinates to cat genome coordinates, and all the analyses of TE insertions within genes were done with this same set of genes (except in the case of ERVs), to avoid possible biases related to different gene annotation qualities across species.

Overall, and consistent with patterns reported for other genomes [175], LINEs and ERVs, are particularly depleted within introns, and when present, tend to be in antisense orientation with respect to the gene (Table S28). However, LINE insertions significantly depart from this general trend and the sense/antisense bias is lost after the lynx-cat divergence, as 57 out of 112 within-gene LINE insertions are found in sense orientation in lynx ($p=0.004$, Fisher's exact test; genome-background frequency of LINE sense insertions is 0.37). This may be a sign of less effective purifying selection. No other TE class showed patterns of insertions within genes in sense orientation significantly different from the background in any of the three species. The proportion of SINE insertions within

genes was also larger in lynx (37.1% vs 35.5%, P-value=5.7e-05) than in other lineages.

Table S28. Proportion of insertion of LINEs within genes and proportion of these insertions that are in the same strand orientation than the gene (sense). Background frequencies are based on the number of occurrences of LINEs in the cat genome. Significant departures from background frequencies according to the Fisher's exact test: (*) p=0.004; (**) p=0.00006;

TE class	Comparison	Total insertions	Within genes (N)	Within genes (%)	In sense (N)	In sense (%)
LINEs	C, background	1,344,033	428,861	31.90%	160,255	37.40%
	C_L	298	83	27.90%	27	32.50%
	L_C	366	112	30.60%	57	50.9% *
	LC_T	166	42	25.30%	13	31.00%
	T_C	380	107	28.20%	34	31.80%
SINEs	C, background	1,639,966	582,084	35.50%	268,042	46.00%
	C_L	10,687	3,710	34.70%	1,690	45.60%
	L_C	15,097	5,597	37.1%**	2,633	47.00%
	LC_T	4,869	1,710	35.10%	791	46.30%
	T_C	5,765	2,067	35.90%	1,005	48.60%
ERVs/LTRs	C, background	1,895	279	14.72%	50	17.90%
	Cat	70	14	20.00%	3	21.40%
	Lynx	2	1	50%	0	0
	Tiger	96	12	12.50%	3	25.00%

15.4 Comparison of TE activity in *Lynx lynx* and *Lynx pardinus*

Since no assembly of the *Lynx lynx* genome is available, we relied on a different strategy to compare the activity of TEs in the two lynx species. We mapped PE reads of each species onto the cat genome (felCat5) and used RetroSeq [176] to call variant TE insertions. Our protocol included the following steps: read mapping, duplicates marking, TEs discovery and filtering. Reads were mapped using BWA v0.6.1 [108] with '-l 75' option for the 'align' command. The software PicardTools v1.60 was used to remove PCR duplicates from the BAM files. These two stages have been executed with the RUbioSeq [177] pipeline on an HPC system scheduled by SGE. Variant TEs were identified with RetroSeq [176]. The discovery phase was executed with the extra parameters '-refTEs <file> -align'. SINE, LINE and LTRs BED files were derived from RepeatMasker output and

based on our previous estimates of active TEs in lynx (see above). Results were filtered according to guidelines recommended by RetroSeq authors. First, calls very close to reference annotated repeat elements were removed. This was done using bedtools v2.16.1 [122] 'window' command with parameters '-w 100' for SINES, and '-w 200' for LINES and LTRs. Finally, we retained only those calls from the VCF file with the following INFO tags: 'FL=8 & GQ>=20'.

We developed an additional filter: first, we identified all confident TE insertions, and compiled the set of reads supporting each of them; then, we determined which read pair mapped close to the TE insertion and which was unmapped or mapped away; and finally, for each TE insertion we compared all the supporting unmapped reads against the collection of TEs with BLAST. This aimed to identify the TE subfamily and its insertion orientation. We retained only those TE insertions for which the orientation and class were unambiguously predicted, i.e. those cases in which at least 5 reads supported the TE insertion, at least 90% of these reads mapped to the same TE strand, and the class was the same as that originally predicted by RetroSeq (i.e. SINE or LINE). To assess the reliability of our predictions, we compared the orientation and subfamily assignments with those TE insertions identified in the genomic-alignment approach (see above). For 6,188 predictions, just 1 and 168 mismatches were observed for our orientation and subfamily assignments, respectively.

Next, we calculated the overlap between the assignments made by RetroSeq+filters for each lynx species and the results of the analysis based of genome alignments (Figure S32). Overall, the number of TE insertions identified with the genome alignment analysis pipeline is generally lower than the number identified with Retroseq+filter, possible due to the very conservative filters imposed in the former and/or to low specificity of RetroSeq predictions. 62% of the SINE (34% of LINE) insertions detected in the genome alignments were also detected with RetroSeq in at least one of the two lynx species.

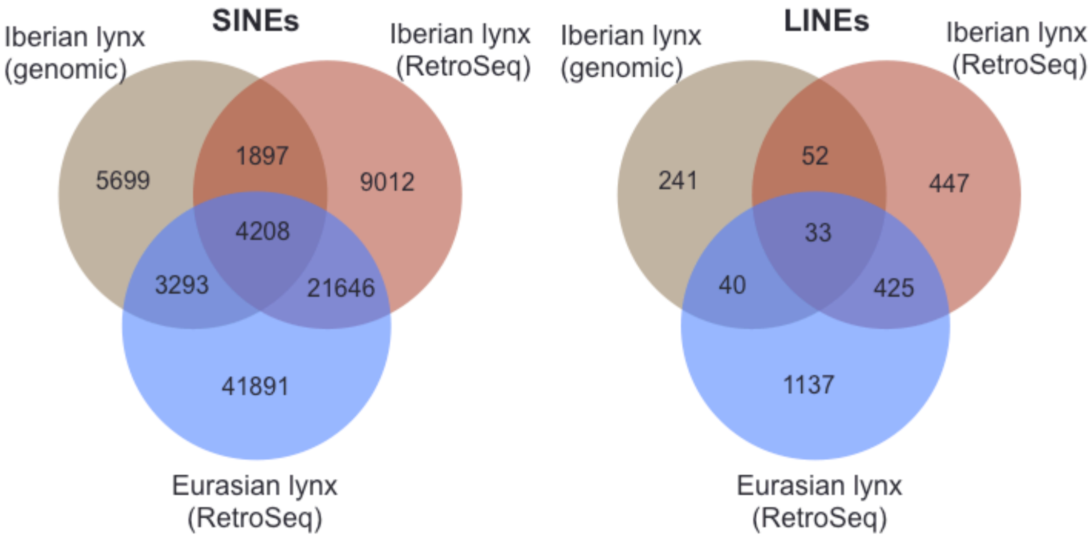

Figure S32. Venn diagrams showing overlap between the sets of species-specific SINEs (A) and LINEs (B) insertions that were identified based on genome alignments (Iberian lynx) or PE-reads mapping (Iberian and Eurasian lynx).

The Venn diagrams show a reasonably good overlap between the two methodologies, the genomic-alignment and reads-mapping approaches, hence reinforcing each other (Figure S32). The large overlap between the Iberian and Eurasian lynx indicates that many of the predicted TE insertions already occurred in a common ancestor, further supporting the reliability of the method.

Our results also indicate that SINEs and LINEs have been more active in *Lynx lynx* than in *Lynx pardinus*. We considered the possibility that the differences between the two lynx species were related to different sequencing qualities, but results remain unchanged after strict filtering of European lynx read data. Remarkably, the relative proportions of active TE subfamilies were very similar for the two lynx species and similar to those obtained based on the cat-lynx genome alignments. For instance, most SINE lynx species-specific insertions correspond to SINEC_Fc, which was also the most abundant Iberian lynx specific insertions in comparison to cat, in detriment of other subfamilies like SINEC_Fc2 and 3 which are more abundantly represented in the genome.

15.5 Patterns of variation in TE insertions in *Lynx pardinus* populations

A similar approach based on RetroSeq and our filtering method was applied to identify polymorphic TE insertions within the Iberian lynx species, although in this case reads were mapped onto the reference Iberian lynx genome. In addition, we rescued predictions that were not strongly supported for a given individual whenever the same prediction was strongly supported in other individual. Our filtering method (see above) was also modified to consider PE reads supporting each TE insertion from all individuals in conjunction. Results reliability was supported by the fact that TE insertion predictions recovered pretty well the population structure, both with SINE or LINE markers, as well as with smaller (random) subsets of these markers (see below).

A total of 7,131 potential polymorphic TEs were identified, corresponding to 6,905 SINEs and 226 LINEs. The relative proportions of TE subfamilies showing activity within the Iberian lynx populations are similar to those observed when Iberian and Eurasian lynx were compared to cat (data not shown), indicating that the same TE subfamilies that have been active since divergence from cat are still active or segregating in Iberian lynx. TE insertions were used as dominant markers for the calculation of diversity and differentiation statistics in GenAlEx v. 6.5 [178, 179]. Our results also show that variability within the Doñana population is much lower than in Sierra Morena (Table S29), and that the two populations are genetically differentiated (*PhiPT* **=** 0,261, *p*=0,007), in agreement to results based on SNP data.

Table S29. Diveristy statistics for TE insertions in Doñana (DON) and Sierra Morena (SMO) Iberian lynx populations. *Na* = No. of different alleles, *Ne* = No. of effective alleles, *I* = Shannon's Information Index, *He* = Expected Heterozygosity, *uHe* = Unbiased Expected Heterozygosity-

**Pop**		**N**	**Na**	**Ne**	**I**	**He**	**uHe**
**SMO**	**Mean**	6,000	1,525	1,350	0,344	0,220	0,240
	**SE**	0,000	0,010	0,004	0,003	0,002	0,002
**DON**	**Mean**	4,000	1,095	1,290	0,255	0,170	0,195
	**SE**	0,000	0,011	0,004	0,003	0,002	0,003

16 Substitution patterns

16.1 Identification and classification of potential substitutions along branches

We built a multiple alignment from the pairwise lynx-cat and tiger-cat genome alignments (see Section 12) and then applied the maximum parsimony criterion to infer the ancestral character states of lynx and cat (*Felinae*). The resulting multiple genome alignment was also used to evaluate the reliability of our variant calling strategy.

To identify substitutions and polarize mutations in Eurasian and Iberian lynxes we called variants using the RubioSeq pipeline [177] and the genome of cat (version 6.2, felcat5) as reference (Section 18.2). Based on the genotype of each lynx species, we selected all those sites, either variant or invariant with respect to cat, which were reliably predicted in both species in homozygosis (heterozygous sites were treated separately). The resulting dataset encompassed 2.15 billion genotyped base pairs. We selected those sites out of repeats and/or low-complexity regions, and within regions of orthology as established in the pairwise genome alignments of lynx, cat and tiger (this makes possible inferring ancestral character states). The final dataset contained 1,062,208,795 genotyped sites, and included 712,201 and 707,025 variants specific of Iberian (*L. pardinus*) and Eurasian (*L. lynx*) lynx, respectively, 9,687,075 variants shared by the two (substitutions occurring since the divergence of cat and lynxes until the divergence of Iberian and Eurasian lynxes), and 1,051,102,494 shared invariant sites. To evaluate the performance of the variant-calling and posterior filters, we counted how many sites were identified as variant in Iberian lynx (or in the lynx ancestor) but found as invariant in the Iberian lynx-cat pairwise genome alignment. Out of 10,399,276 variants predicted, just 2,805 (0.027%) were in disagreement. On the other hand, genome alignments revealed 150,026 variants (out of ~1 Gbp of aligned sites, 0.014%) that were predicted as invariant by RUbioSeq. Just 363 out 707,025 (0.051%) of the Eurasian lynx-specific variants were found as variant between Iberian lynx and cat at the alignment level. Summarizing, these comparisons strongly support

the reliability of our variant-calling strategy. It must be noted though that our approach is not appropriate to measure absolute evolutionary rates (a larger fraction of substitutions than of invariant sites did not pass our stringent filters) but is aimed to provide an appropriate framework to compare the evolutionary patterns among species.

Identified substitutions were used to estimate substitution rates, non-synonymous to synonymous substitution ratios (dN/dS), and weak-to-strong (mutations from A/T to G/C; hereafter W→S) substitution biases. The analysis of substitution rates is used to identify regions with differential rates of evolution in specific branches. Ratios of dN/dS were used to estimate the strength of purifying selection. High W→S biases are the main hallmark of GC-biased gene conversion (gBGC), a process by which repair mechanisms favour strong (G/C) over weak (A/T) heterozygous alleles during meiotic recombination [180, 181]. The action of gBGC has been linked to high mutation rates [182], increased genetic diversity [183], and fixation of slightly deleterious substitutions [184].

We translated cat genome coordinates to lynx scaffold coordinates (based on the genome alignments) for each substitution identified and annotated their effect on protein-coding genes based on the principal transcript isoforms identified with APPRIS [185] and using SnpEff v3.5 [186]. From the raw counts of non-synonymous and synonymous substitutions we estimated dN/dS ratios by assuming that ¾ of all of the sites are non-synonymous. This is an assumption that, although may not hold for single genes, is reasonable for large number of sites. Although biased codon-usage may alter the expected proportion of non-synonymous substitutions under equal nucleotide frequencies, the resulting dN/dS ratios are adequate for comparative purposes.

16.2 Identification of regions with differential rates of evolution in specific branches

Mutations were associated to each branch of the tree leading to cat, the lynx ancestor, and the Iberian and Eurasian lynxes. Mutational patterns, including rates and W→S bias, were condensed into 100 Kbp windows. Only those windows with at least 10,000 informative sites (Section 18.2) were retained.

Overall relative rates of evolution were estimated between: cat and the lynx ancestor (0.58, meaning that 58% of the divergence between the two accumulates in the former); Eurasian and Iberian lynx (0.50); and between cat and Iberian and Eurasian lynx (0.54 in each case). We took these relative rates as the expected proportion of successes in a binomial test (FDR-corrected) and identified windows whose proportions significantly deviated from the overall relative ratios. Resulting windows were analysed in terms of chromosome distribution, distance to telomeres, and W→S biases (Figure S33).

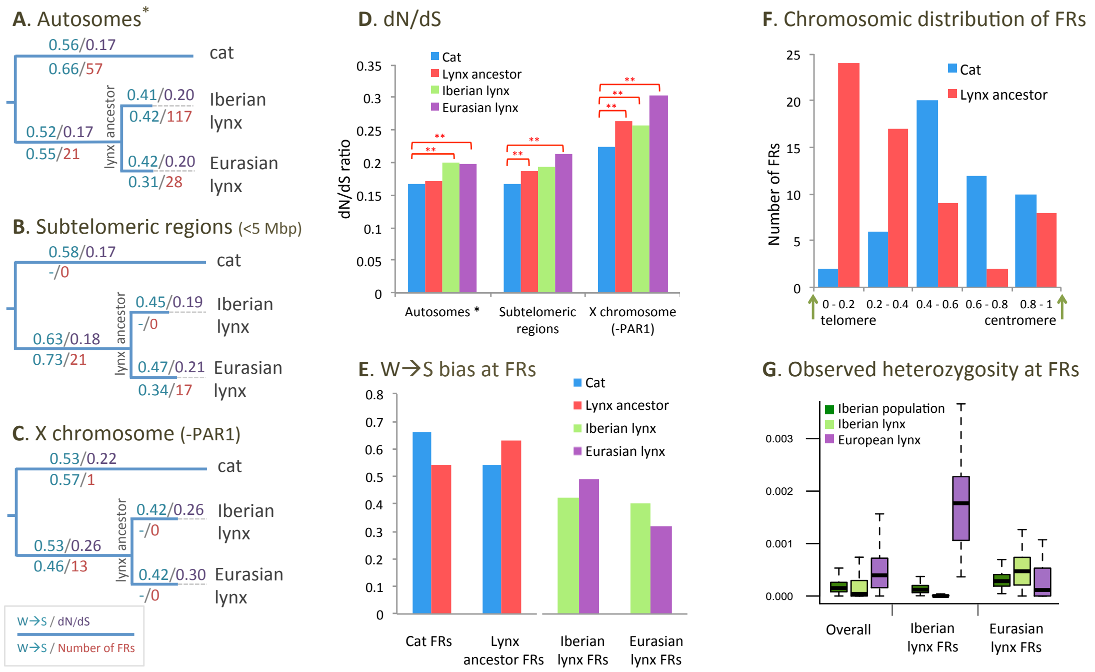

Figure S33. Mutational patterns along lynx and cat evolution. The W→S bias, dN/dS ratio and the number of evolutionary faster regions (FRs) are shown for each branch of the tree that relates cat, Iberian and Eurasian lynx for three different genomic regions: autosomal chromosomes (*: excluding subtelomeric regions and centromeres (**A**); 5 Mbp of subtelomeric regions (**B**); and sexual chromosome X (excluding first 6Mb corresponding to pseudoautosomal region 1; **C**). Numbers above branches indicate global W→S bias (blue) and dN/dS ratios (purple), and numbers below branches correspond to evolutionary FRs, indicating the W→S bias (blue) and the number of FRs (red). dN/dS ratios along each branch and at each chromosomal region (**D**), with significant statistical differences indicated by asterisks (p-val < 0.05, Fisher's exact test). Genome-wide W→S bias for each set of FRs (**E**). Chromosomal distribution of FRs identified in the cat versus lynx-ancestor comparison at different distances from telomeres (normalized to a 0-1 scale, 1 meaning centromeric location; **F**). Observed heterozygosity at FRs identified at the European versus Iberian lynx comparison (**G**).

16.3 Substitution patterns and rates along branches and chromosomes

Our results indicate that, on average, cat shows higher rates of neutral evolution, as its lineage accumulates 54% of the lynx-cat divergence. Remarkably, the W→S bias is much higher in cat than in lynx species, especially after the divergence of Iberian and Eurasian lynxes (Figure S33A-C; Table S30). On average, excluding autosomal telomeres and the X chromosome, the W→S bias accumulated along the cat lineage is 0.56, whereas in the branch leading to the lynx-ancestor it becomes lower (0.52), and then it is drastically reduced after the evolutionary split between Eurasian and Iberian lynxes (0.42; Figure S33A; Table S30).

Table S30. Mutational patterns in different chromosomal regions and lineages, including W→S bias at faster-evolving regions (FRs).

Genome-wide	Cat	Lynx ancestor	Iberian	Eurasian
**Subtelomeric regions**				
Substitutions	297647	232657	33084	43397
W→S bias	0.58	0.63	0.45	0.47
W→S bias lynx-ancestor FRs (21)	0.54	0.73	-	-
W→S bias Eurasian lynx FRs (17)	-	-	0.49	0.34
**Chromosome X (-PAR1)**				
Substitutions	172739	127408	25856	27458
W→S bias	0.53	0.53	0.43	0.42
W→S bias lynx-ancestor FRs (13)	0.44	0.46	-	-
**Rest of the genome**				
Substitutions	4932812	3588673	635875	618518
W→S bias	0.53	0.53	0.42	0.42
W→S bias lynx-ancestor FRs (21)	0.54	0.55	-	-
W→S bias cat FRs (58)	0.66	0.54	-	-
W→S bias Eurasian lynx FRs (28)	-	-	0.36	0.31
W→S bias Iberian lynx FRs (117)	-	-	0.42	0.49

Increased W→S ratios are the main hallmark of GC-biased gene conversion (gBGC), a process by which repair mechanisms favour strong (G/C) over weak (A/T) heterozygous alleles during meiotic recombination [180, 181]. The action of gBGC has been linked to high mutation rates [182], increased genetic diversity [183], and fixation of slightly deleterious substitutions [184]. As both inbreeding and genetic drift occurring in bottlenecked populations result in high levels of homozygosity, gBGC is ineffective under those situations. In fact, the patterns of mutation in selfing and out-crossing species of the genera *Arabidopsis* [187] and *Oryza* [188], as well as results from simulations [189], confirm the counter play that exists between inbreeding and gBGC. The occurrence of several important

bottlenecks along Iberian and Eurasian lynx histories, especially after their divergence (Section 9), has resulted in high homozygosity and low rates of gBGC. The weaker action of gBGC in lynx could also explain the higher rates of divergence along the cat lineage.

The hallmark of gBGC, i.e. high W→S bias and rate acceleration, has been related to male-driven recombinational processes in human and chimpanzee as it best correlates with male recombination rates [190, 191]. For instance, whereas gBGC is particularly frequent at subtelomeric regions, which are highly recombinogenic in males [192-195], it is scarce or absent at the X chromosome, which only recombines in females (to the exception of pseudo-autosomal regions, which show much larger W→S bias). Here we find these same trends: high W→S bias at subtelomeric regions and low W→S bias at the X chromosome along the branches corresponding to cat and lynx ancestor (Figure S33B-C). In contrast, these gBGC hallmarks are much weaker after divergence of Eurasian and Iberian lynx species, although a slightly higher W→S bias can be appreciated at subtelomeric regions.

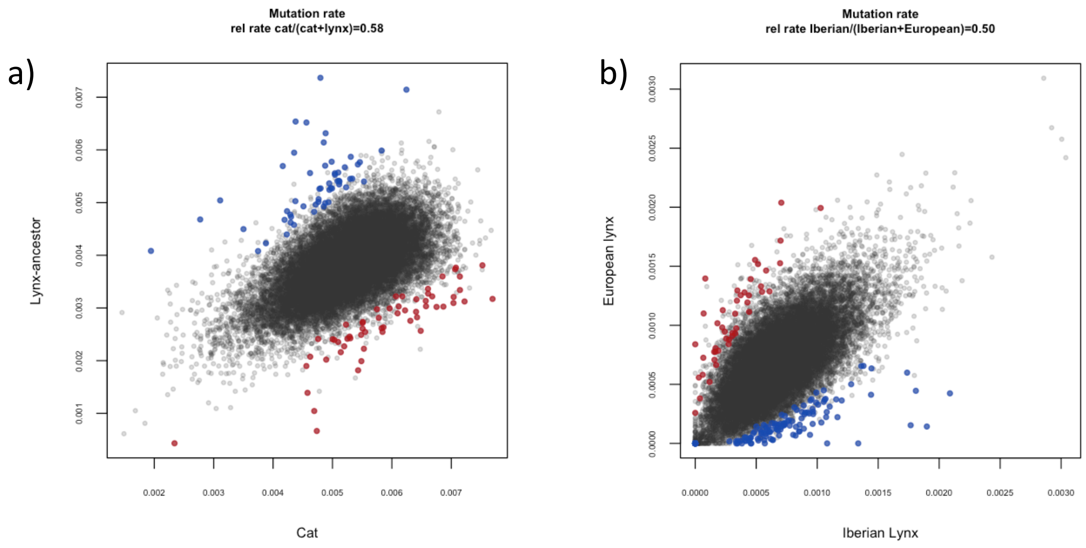

Figure S34. Windows identified as evolving faster than the genome average in the lynx-ancestor (blue) vs. cat (red) comparison (a) and in the Eurasian (red) and Iberian lynx (blue) comparison (b).

To further investigate the possible role of W→S bias and bBGC in determining lineage-specific divergence, we identified regions that evolve faster (FRs) in one

branch compared to its sister branch (Figure S34). Regarding the cat versus lynx-ancestor comparison, a significant fraction of lynx-ancestor FRs (55) map to subtelomeric regions (21) and the X chromosome (13), whereas cat FRs (58) distribute much more homogeneously among and along chromosomes (Figure S33F). Despite these topological differences, most of the identified FRs (42 out of 55 in lynx-ancestor, 50 out of 58 in cat) show W→S biases that are larger in the species showing faster rates and at the same time are larger than their genome-wide average (Figure S33E). Interestingly, X-chromosome FRs of lynx-ancestor behave differently than the rest, with low W→S bias values (0.46) that are even lower than the chromosome average (0.53), to the exception of one FR that maps to pseudo-autosomal region 1 and has a high W→S bias of 0.64, as could be expected given that this region recombines in males. Notably, Eurasian and Iberian lynx FRs, 46 and 117 in each, respectively, show the opposite trend, with lower W→S biases in the faster species, even lower that than the genome average (Figure S33A, E). These results further support that gBGC has been inefficient during the recent history of Iberian and Eurasian lynxes as, contrarily to what happened with most cat and lynx-ancestor FRs, regions with altered rates of evolution cannot be attributed to increased W→S bias.

The regions that we identified as faster correspond to regions with differential rates between pairs of species (or ancestral nodes), and contrarily to other studies [196], do not necessarily imply rate acceleration. In this regard, we observe a different behavior between the two sister-branches comparisons. Whereas all lynx-ancestor FRs (55), 43 out of 58 cat FRs, and 40 out of 46 Eurasian lynx FRs have substitution rates higher than the genome average, hence suggesting accelerated rates, only 68 out of 117 Iberian lynx FRs have substitution rates higher than its genome average.

If FRs are not related to recombination/gBGC in Eurasian and Iberian lynx species since their divergence, what is their origin? We wondered whether the same process that reduces the efficacy of gBGC (i.e. high levels of homozygosity) could explain the origin of FRs. To answer this, we calculated the observed heterozygosity in each lynx species, and then compared the Iberian and Eurasian lynx FRs. We found that heterozygosity in FRs is consistently much lower in the

species showing faster rates (Figure S33G), especially in the case of Iberian lynx FRs. Moreover, the levels of heterozygosity were higher and lower than the average in the slower and faster species, respectively. This strongly suggests that FRs correspond to regions with extreme loss of heterozygosity. We explicitly validated this hypothesis by focusing on those sites that are fixed variants with respect to cat in one lynx species, and at the same time are segregating in the other. The resulting set includes 93,293 sites that most likely were polymorphic in their last common ancestor but then became fixed in one of the two lynx species (note that sites being polymorphic in any of the two species were explicitly excluded from initial divergence analyses). Of these 93,293 sites, 70,518 (75.6%) and 22,775 (24.4%) became fixed in Iberian and Eurasian lynx, respectively. Our results are clear: in the Iberian lynx FRs 2,023 out of 2,049 ancestrally polymorphic sites (98.7%) became fixed in Iberian lynx, whereas in the Eurasian lynx FRs, 194 out of 233 ancestrally polymorphic sites (83.3%) became fixed in Eurasian lynx, hence indicating that faster rates are caused by increased fixation rates. Remarkably, the higher number of FRs (117 vs 46) and the degree of loss of ancestral heterozygosity indicate that Iberian lynx experienced stronger demographic bottlenecks than the Eurasian lynx, and agrees with genome–wide patterns of genetic diversity and other signs of genetic erosion (see below and Supplementary Notes 19-24).

To further study the relationship between loss of heterozygosity and substitution rates in lynx, we calculated for each window the relative proportions of heterozygous variants and fixed substitutions that are attributed to Iberian lynx with respect to Eurasian lynx. We found that the two ratios are negatively correlated along the genome (r=0.32, p-value<2.2e-16), implying that those regions in which Iberian lynx lost more heterozygosity with respect to Eurasian lynx have higher (relative) rates of substitution. It is important to note that substitution rates were calculated using only those sites that were (reliably) genotyped as homozygous in both lynx species.

Both inbreeding and gBGC have been reported to increase the probability of fixation of deleterious or slightly deleterious alleles [183, 184]. Indeed, increased homozygosity produces lower effective recombination rates [168] and an

increase in genetic drift is associated to a decreased power of selection. To gain further insights on this, we measured how the strength of purifying selection varied along each lineage and to what extent these variations may relate to gBGC, inbreeding, and/or distinct effective population sizes. We calculated approximate ratios (dN/dS) of substitution rates at non-synonymous (dN) and synonymous (dS) sites as a proxy to measure the strength of purifying selection. Our results indicate that dN/dS ratios are in general significantly higher in lynx than in cat branches. The difference reached significance in the Eurasian lynx branch at all three genomic compartments (Fisher's exact test p-values: 0,003, 2.3e-10 and 0,008 for subtelomeric regions, non-subtelomeric/non-pericentromeric autosomal regions and chromosome X, respectively), in the Iberian branch, significance was reached at non-subtelomeric/non-pericentromeric autosomal regions and chromosome X (Fisher's exact test p-values: 2.3e-12 and 0,011, respectively), and in the lynx ancestor branch significance was moderate at subtemoleric regions and the X chromosome (Fisher's exact test p-values: 0,016 and 0,011, respectively) (Figure S33C). Eurasian and Iberian lynx dN/dS ratios were not significantly different in any of the three compartments. Interestingly, in the X chromosome, dN/dS ratios are much higher that the genomic background in all lineages. Since gBGC is basically inefficient at X chromosomes, increased dN/dS ratios must reflect the lower effective population size of this chromosome [197]. The fact that X-chromosome dN/dS ratios are particularly high at Eurasian and Iberian lynx branches may reflect their even lower (species) effective population sizes (see below) and the effect of demographic bottlenecks. At subtelomeric regions, where gBGC is most active, one could expect higher dN/dS ratios than in the rest of the autosomes. This effect can be appreciated in the lynx-ancestor, where the dN/dS is significantly larger in subtelomeric regions than at other compartments (Fisher's p=0.012), and where a significantly large proportion of FRs (21 out of 55) concentrates.

17 Segmental duplications

We detected segmental duplications (SDs) in the genomes of 12 lynxes including 1 Eurasian lynx (*Lynx lynx*) and 11 Iberian lynxes (*Lynx pardinus*) both from Sierra Morena (7) and from Doñana (4) (Table S31).

Reference assembly. We downloaded the Fca-6.2 (UCSC felCat5) assembly from The UCSC Genome Browser (http://genome.ucsc.edu/) [198]. The 5,480 scaffolds either unplaced or labeled as random were concatenated into a single artificial chromosome. In addition to the repeats already masked in felCat5 with RepeatsMasker (www.repeatmasker.org) and Tandem Repeat Finder [199], we sought to identify and mask potential hidden repeats in the assembly. In order to do so, chromosomes were partitioned into 36-bps kmers (with adjacent kmers overlapping 5 bps) and these mapped against felCat5 using mrsFast (Hach et al., 2010). Then we masked positions in the assembly mapped by kmers with more than 20 placements in the genome, resulting in 5,942,755 bps additionally masked compared to the original masked assembly.

Preparation of the sequencing reads. Illumina 100-bps reads were mapped using BWA [108] (using as parameters 'bwa -q 15') to autosomes in the prepared version of the cat genome (see above) and duplicated reads were removed with SAMtools [146]. Successfully mapped reads in the resulting BAM files were then used to recover the original FASTQ files using the bam2fastq tool (http://gsl.hudsonalpha.org/information/software/bam2fastq). The final set of 100-bps reads were clipped to 36 bps-fragments but only retaining positions in the read with high quality, which was assessed with fastqc (http://www.bioinformatics.babraham.ac.uk/projects/fastqc/). Specifically, we retained positions 1–36 and 37–72 in all samples with the exception of the Candiles sample, in which we used positions 8–43 and 44–79.

Mapping and copy number estimation from read depth. The resulting 36-bps reads were then mapped to the prepared reference assembly (see above) using mrFast [200] (using as mapping parameters '–e 2'). mrCaNaVaR (version 3.0.1) [201] was used to estimate the copy number along the genome from the mapping read depth. Briefly, mean read depth per base pair is calculated in 1-

Kbp non-overlapping windows of non-masked sequence (that is, the size of a window will include any repeat or gap and thus the real window size may be actually larger than 1 Kbp). Importantly, because reads will not map to positions covering regions masked in the reference assembly read depth will be lower at the edges of these regions, which could underestimate the copy number in the subsequent step. To avoid this, the 36 bps flanking any masked region or gap were masked as well and thus not included within the defined windows. In addition, gaps >10 Kbps were not included within the defined windows. A read depth distribution is obtained through iteratively excluding windows with extreme read depth values relative to the normal distribution and the remaining windows are defined as control regions. The mean read depth in these control regions is considered to correspond to copy number equal to two and used to convert the read depth value in each window into a GC-corrected absolute copy number in each sample.

SDs calling. We called SDs in each individual as genomic regions in which the predicted copy number significantly exceeded diploidy while accounting for the technical variation in the copy number predictions across samples. Specifically, the 1-Kbp copy number distribution in control regions (Figure S35) was used to define two sample-specific gain cutoffs corresponding to the mean copy number plus 2 ('soft' cutoff) and 3 ('hard' cutoff) units of standard deviation (calculated not considering those windows exceeding the 1% highest copy number value). Note that as the mean copy number in the control regions is equal to two by definition, then the gain cutoffs will be largely influenced by the standard deviation. After excluding 1-Kbps windows with a predicted copy number greater than 100 copies, we conservatively defined a SD when the copy number was greater than the defined sample-specific cutoffs in 5 contiguous windows (allowing that at most 1 window exceed the 'soft' cutoff). Furthermore, we filtered out SDs shorter than 10 Kbps and with >85% of their size overlapping with repeats.

Analysis of shared and population-specific SDs. We excluded from this analysis the Sierra Morena Iberian lynx Candiles as it presented a notably distinct pattern of copy number predictions in the control regions compared to the rest of the

samples, showing a greater variation in the 1-Kbps copy number distribution (Figure S29). As a similar copy number distribution across samples is expected in control regions, in which no increased copy number is anticipated, we suspect that this observation in the Candiles sample arises from technical differences during the sequencing step instead of reflecting any biological meaning. Consequently, we excluded the Candiles sample to avoid that it distorted the between-groups comparisons.

Table S31. Length of duplicated sequence per sample

Species	Population	Sample	Length of duplicated sequence (bps)*
Lynx pardinus	Sierra Morena	A002	7,832,661
Lynx pardinus	Sierra Morena	A005	7,579,716
Lynx pardinus	Sierra Morena	A006	8,669,189
Lynx pardinus	Sierra Morena	A008	8,419,692
Lynx pardinus	Sierra Morena	B991	7,405,642
Lynx pardinus	Sierra Morena	B992	8,253,233
Lynx pardinus	Sierra Morena	Candiles	8,443,120
Lynx pardinus	Doñana	A003	7,849,502
Lynx pardinus	Doñana	A007	6,768,521
Lynx pardinus	Doñana	A009	7,357,074
Lynx pardinus	Doñana	A010	7,502,914
Lynx lynx	–	LynxLynx	7,571,394
All			11,554,996

*Based on SDs called on autosomes.

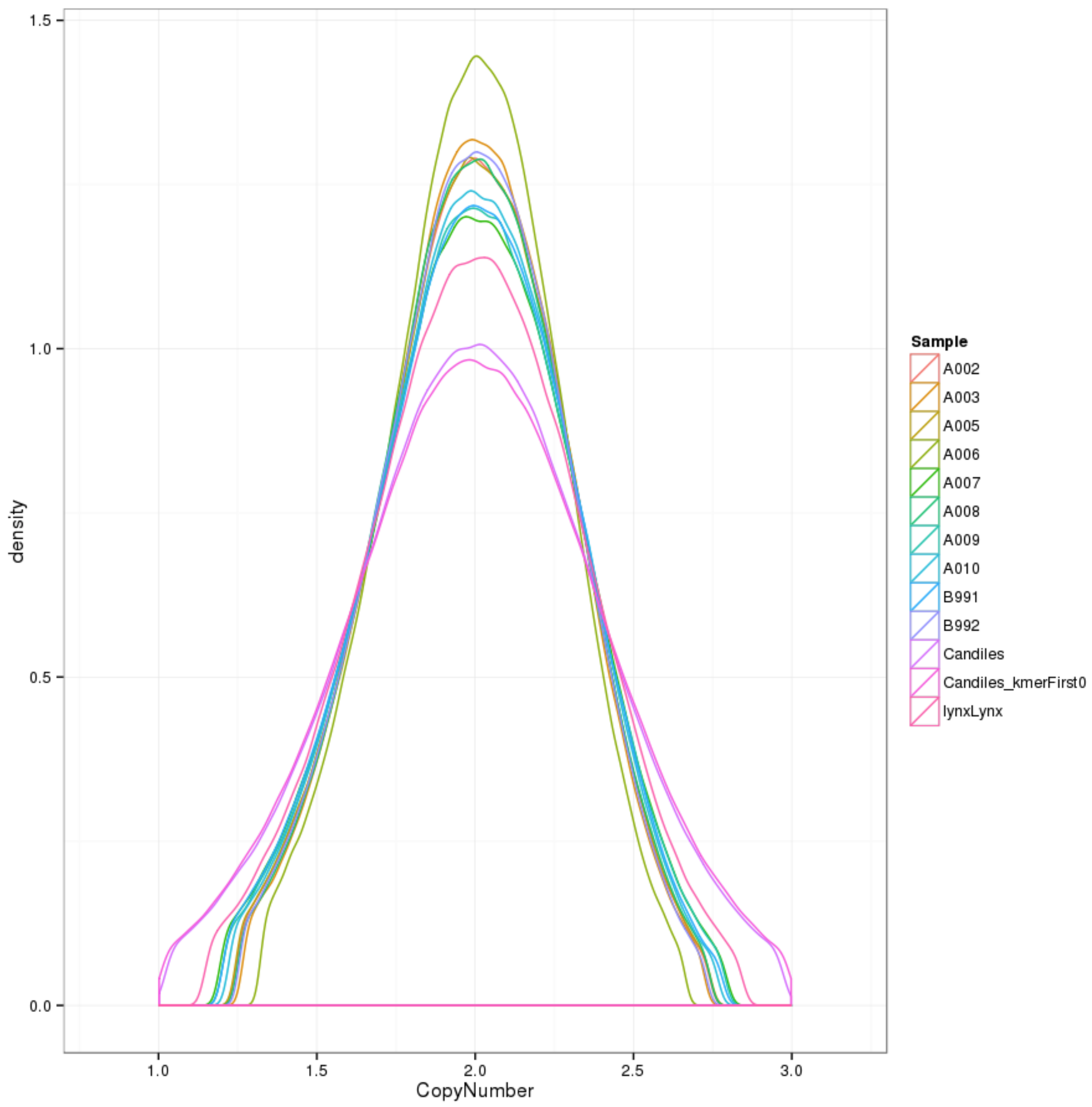

Figure S35. Predicted 1-Kbps copy number values in control regions. Shown is the distribution of copy number values in non-overlapping windows of 1 Kbps of non-repetitive sequence. The copy number in each window (in both control and non-control windows) was calculated as two times the read depth in the window divided by the median read depth in control regions. The Candiles sample ('Candiles') showed a broader variation in the distribution of predicted copy number values compared to the other samples, even when this was processed differently aiming to minimize such effect ('Candiles_kmerFirst0').

After generating the map of SDs in each of the 12 lynx individuals, we estimated that each genome harbors 6.77–8.67 Mbps (mean=7.80 Mbps) of duplicated sequence. This is a conservative estimate since it does not account for the location of the multiple copies and we used the most conservative methods by requiring five consecutive windows with depth of coverage higher than that expected for a diploid locus. When all SDs across samples were combined, we obtained that about 12 Mbps of sequence are within SDs in the lynx genome

(Table S31). Moreover, maps of SDs for each individual allowed us to compare the patterns of structural variation between the two Iberian lynx populations (Sierra Morena and Doñana) and the Eurasian lynx. We observed that the proportion of Iberian lynx SDs shared with the Eurasian lynx is high (7.04 Mbps, 67.50%) and similar in the two Iberian populations (Figure S36). On the other hand, 8.10 Mbps (77.66%) are shared by the two Iberian lynx populations, with a larger length of SDs being private to Sierra Morena (1.83 Mbps) than to Doñana (0.50 Mbps) (Figure S36), consistent with differences observed in SNP diversity (see Section 20).

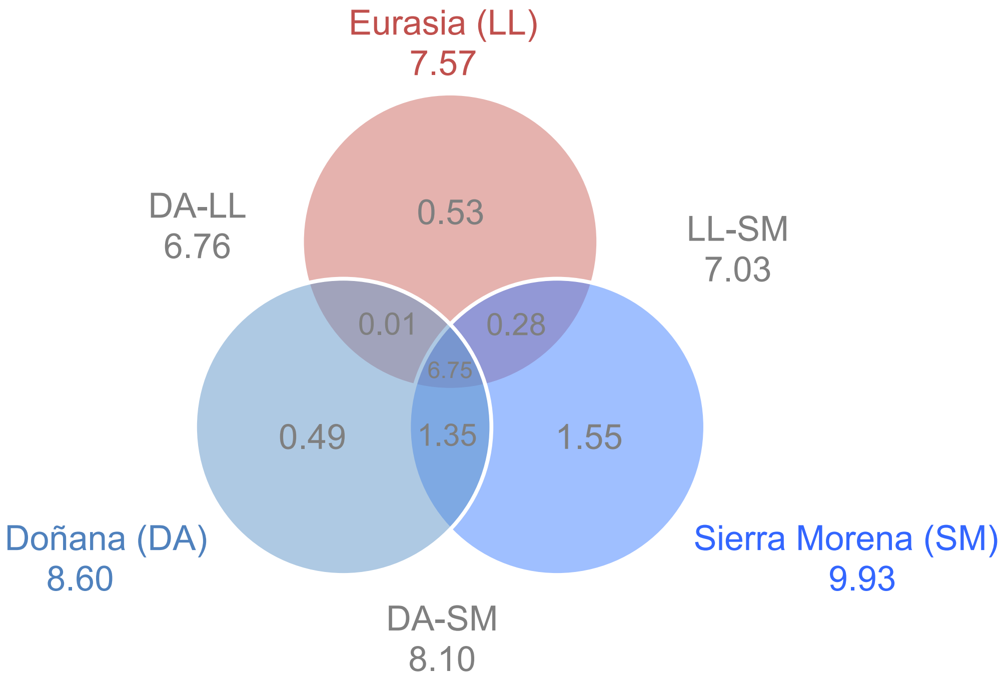

Figure S36. Overall sharing of SDs in the lynx populations. After defining the SDs in each lynx population, the Venn diagram shows the length of duplicated sequence (Mbps) shared between groups. SDs in each Iberian lynx population were defined as duplication events seen in at least one individual of the population (Sierra Morena, n = 6; Doñana, n = 4); note that SDs in the Eurasian lynx reflect those identified in the single sample included in this study. We show the total length of duplicated sequence (Mbps) in each population (colored numbers).

18 Variant discovery and genotype calling

18.1 Assembly-based approach

We applied the reference-free and assembly-based strategy for variant detection implemented in *Cortex_var* [202], on the Illumina PE reads of 11 males of Iberian

lynx (Table S2). The software builds a *de Bruijn* graph (e.g., [203]) for each sample and compares the resulting graphs to detect consensus sequences where there is enough statistical support for the existence of two alleles. The assembly and variant discovery was carried out with the *bubble caller* algorithm that looks for consensus motifs in the graph (*bubbles*) created by polymorphisms or repeats. If more than 10 samples are combined, it is also possible to distinguish repeat-induced bubbles from true variant sites by applying the *population filter* [204].

The kmer length parameter determines the sensitivity and proneness to error of the calls. We used a value of K=31, the same k-mer length used for variant detection with Cortex using Phase 2 1000 Genomes data (http://cortexassembler.sourceforge.net/cortex_phase2_recipe.pdf), which is expected to have a reasonable compromise between sensitivity (~80% sensitivity) and error rates. The following steps where performed at the Supercomputing Center of Galicia (CESGA, Santiago de Compostela):

1. Build a binary graph for each sample with a quality filtering of 5 and PCR duplicates removal. This step was performed with the *cortex_var-1.0.5.3* release.

2. Clean binary graph by removing low-coverage supernodes. The graphs were cleaned by removing those supernodes containing all interior nodes with coverage smaller than N. This coverage cut-off, N, is the coverage value right after the sequence error peak in the kmer coverage distribution. The cleaning step was also done using *cortex_var-1.0.5.3* release.

3. Variant and Genotype Calling. A genotype table was obtained for each variant, including a log-likelihood estimate for each genotype and a genotype call assigned to the one with the highest likelihood. Note that, the reference assembly was totally ignored during the calling, thus maintaining the complete avoidance of reference-bias (equal power for discovery of both alleles) and the calling of unnecessary and irrelevant variants where all samples differ from the reference. The total number of called variants during this step was 2,407,928.

4. Detection of variants with the population filter. The *population filter* implemented in an accompanying script called *Classify.R* uses information from multiple samples to distinguish polymorphisms from repeats or errors among the *bubbles* present in the *De Brujin* graph ([e.g. 205]). *Classify.R* estimates the log-likelihood of each of the possible causing patterns (variants, repeats and errors) for every *bubble*. In our case, 1.97 million variants were retained by the population-filter (version 1.0.5.14) out of the 2,407,928 raw calls obtained before (step 3).

5. Classification of variants and mapping filter. We used the script *process_calls.pl* distributed with cortex release 1.0.5.14 both to classify variants *a posteriori* and to map these variants to the Iberian lynx reference assembly (*lp23*). The script uses *Stampy* [206] to map each variant to the assembly with a minimum quality of 40 and classifies them by variant type (SNP, Indel…) based on the alignment of both alleles. Table S32, provides a full list of variants or polymorphisms found in the intra-specific (using 11 Iberian Lynx males) and inter-specific calls (adding the Eurasian lynx sample up) by cortex. While the cortex intra-specific SNPs were used for population genetic analyses, the inter-specific dataset was employed to estimate genome-wide heterozygosities. Additionally we have used the intersection of both to infer recent demography with *∂a∂i [107]*. The total number of SNPs detected in this call was approximately 1.4 million in Iberian lynx and 3.9 million using both lynx species (although they are mostly substitutions). After excluding putative substitutions, the intersection of both calls accounts for 1.2 million SNPs.

6. Confidence and completeness filters of cortex SNPs. Additionally, we applied several filters in order to obtain a reliable dataset for population genomics analyses. Cortex reports a confidence value for each variant and for each genotype call at that site, calculated as the difference between the maximum and the next largest log likelihood. Thus the higher is the confidence the less ambiguous the call. From the distribution of site and genotype confidence value obtained from the 1,443,758 SNPs passing cortex population and mapping filters (‘*cortex Lypa’* dataset, Table S32), we identified minimum

threshold values of 11 for the site confidence and 2 for the mean genotype confidence. As confidences are in log space, a site confidence of 11 means this is 10^{11} times more likely than the alternative and an average genotype confidence of 2 means that on average each genotype is 100 times more likely than the closest alternative. Finally, we restricted our dataset to sites for which all 11 samples could be reliably genotyped. The applied filters improved the distribution of confidence and coverage statistics by removing potential artefacts. The total number of SNPs meeting these criteria was 1,162,256 and from now onwards we will refer to this set as the *filtered cortex Lypa* dataset.

We have also applied these filters to the inter-specific dataset and the intersection keeping 3,619,487 and 1,041,728 SNPs respectively. Although the inter-specific cortex originated 3,619,487 biallelic sites, 1,957,006 sites were considered as putative substitutions because all Iberian lynx samples were homozygous for one allele and the Eurasian lynx sample was homozygous for the other allele. After filtering these sites, the remaining 1,662,481 SNPs were used to compare the genome-wide heterozygosity across samples (Section 20.3). For the demographic analyses with *∂a∂i [107]* (Section 10.2), we used a dataset that comprised 1,041,728 SNPs identified in both calls that were polymorphic in Iberian lynx.

Table S32. Number of variants that passed the population and mapping filters in *cortex_var* in calls performed for Iberian only (Lypa) or for both lynx species (Lypa+Lyly).

Variant Type	Cortex Lypa	Cortex Lypa+Lyly
SNP	1,443,758	3,930,529
SNP_FROM_COMPLEX	174,486	656,287
DEL	121,373	289,104
INS	121,871	282,126
INDEL_FROM_COMPLEX	15,158	65,771
INV_INDEL	39	131
Total passing the filters	1,876,685	5,223,948

SNP = "SNP"; SNP_FROM_COMPLEX = "SNP called from a cluster of phased SNPs or complex SNP/indel collection, split out for easier comparison with other SNP call sets"; DEL = "Deletion"; INS = "Insertion of novel sequence"; DEL_INV = "Deletion + Inversion"; INDEL = "Insertion-deletion"; INDEL_FROM_COMPLEX = "Insertion-deletion from a complex region"; INV_INDEL = "Inversion+indel".

18.2 Reference-based approach

The RUbioSeq suite [177] was used for the genotyping and variant identification analyses using a mapping-based approach. The protocol executed by RUbioSeq includes the following steps: read mapping, duplicate marking, GATK realignment, GATK calling, and results post-filtering. Reads were mapped using BWA (v0.6.1) customizing bwa 'align' command with '-l 75' option. PicardTools (v1.107) were used to remove duplicates from the BAM files. Genome Analysis Toolkit version 2.7-4-g6f46d11 was used for the realignment and calling steps. Genotype calling was performed with the UnifiedGenotyper function and “EMIT_ALL_SITES” option. The results were filtered to produce the final callsets using GATK VariantFiltration functions. Default RUbioSeq filtration applied to the variant sites is: "QD < 2.0 || FS > 60.0 || MQ < 40.0 || HaplotypeScore > 13.0 || MappingQualityRankSumTest < -12.5 || ReadPosRankSum < -8.0 || QUAL < 100". Variant sites that comply any of these filters were removed from the final set of called variants.

In order to generate genotype data for population genomics and species divergence analyses, variant discovery and genotype calls were performed for the two lynx species using either the Iberian lynx or the cat genome as reference (Table S33).

18.2.1 Joint Iberian lynx variant call against the Iberian lynx genome.

To generate a dataset for population genomics, the reads from 11 Iberian lynx individuals were combined into a joint multi-sample sites dataset. The joint multiexecution of RUbioSeq was enabled for this purpose. The samples were mapped against the Iberian lynx reference genome (lp23.fa). Invariant sites were called and those with a quality lower than 50 were removed from the dataset. In the case of variants, extra filters were used to remove sites with depth lower than 20 and higher than 669 (mean + 5*sd), non biallelic sites, sites that were called alternative homozygous in the reference genome individual, sites with less than 4 genotypes in each population (SMO and DON), sites where all individuals were called as heterozygotes and sites with one or more individual with Genotype Quality lower than 20.

18.2.2 Eurasian lynx variant call against Iberian lynx genome.

A set of callable and variant sites was also generated for the Eurasian lynx individual by mapping against the Iberian lynx genome (lp23.fa) as described above. Extra filtering steps were conducted to remove invariant sites with a quality lower than 50 and variant sites with coverage depth greater than 233 (mean + 5*sd) and low genotype quality (GQ < 20).

18.2.3 Eurasian lynx and Iberian lynx variant call against the cat genome.

Two sets of callable and variant sites were generated by mapping against the cat genome (v. 6.2, felcat5): one for the Eurasian lynx and another one for the Iberian lynx reference individual (Candiles). Extra post-filtering was applied to RUbioSeq's output to remove: invariant sites with quality lower than 50, variant sites with coverage depth greater than 173 and 282 for Eurasian and Iberian lynx, respectively, and genotype quality (GQ) lower than 20.

Table S33. Description of the four reference-based variant calling performed in this study.

Data	Reference	Depth	Invariant sites	Variant sites	Total callable sites
**All Iberian lynx (N=11)**	Lp23	302x	2,019,224,377	1,587,544	2,020,811,921
**Eurasian lynx**	Lp23	47x	2,291,115,126	5,820,814	2,296,935,940
**Eurasian lynx**	Cat 6.2	41x	2,193,634,924	26,171,339	2,219,806,263
**Iberian lynx (Candiles)**	Cat 6.2	80x	2,151,330,656	22,416,383	2,173,747,039

18.3 Comparison of reference- and assembly-based datasets

18.3.1 Variant overlap and genotype concordance

With the double aim of identifying unreliable sites and setting up an additional set of quality-filters, we compared the variants identified for the Iberian lynx population based on the multiexecution RUbioSeq analysis with those identified with CORTEX.

First, we compared raw variant calling results. We observed that in terms of the number of called sites, the set identified with RUbioSeq is 33% larger than that of CORTEX, with 80.3% of the sites identified by CORTEX overlapping with RUbioSeq, and 54% of the sites identified by RUbioSeq overlapping with CORTEX (Figure S37).

Focusing on the intersection between RUbioSeq and CORTEX we found that the two methods showed different genotyping biases. In those cases in which the two methods provided discordant genotypes, we found that RUbioSeq tended to genotype heterozygous sites which CORTEX genotyped as homozygous, and vice versa (Table S34). These differences resulted in a Non-reference discordant rate (NDR) of 7.04.

Based on the comparison of genotypes, we devised an additional set of filters to identify the most confident sites and remove false positives and unreliable genotypes (Supplementary Notes 18.2 and 18.3). This post-filtering removed 24.99% and 25.97% of the variants called by CORTEX and RUbioSeq, respectively. As a result of these additional filters, the intersection between RUbioSeq and CORTEX improved, increasing to 89.8% and 61.3% of the CORTEX and RUbioSeq sets of variants, respectively. Remarkably, the NDR decreased to 2.33%, indicating that the filters successfully reduced much of the genotyping bias of each software. (Table S34).

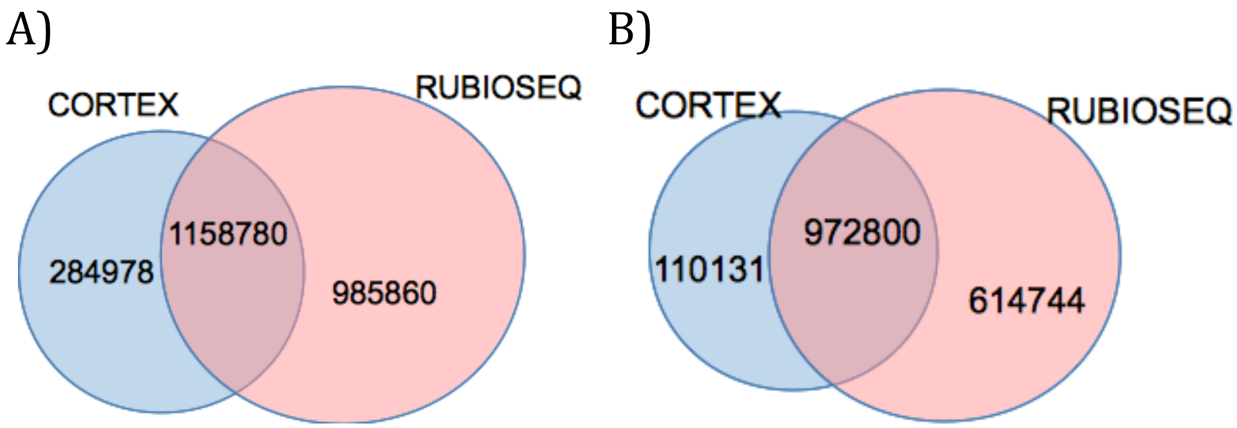

Figure S37. Venn diagrams comparing the variant sets identified by Cortex and RUbioSeq before (A) and after (B) the application of filters described in sections 16.2 and 16.3

Table S34. Comparison of the genotype calls made by Cortex and RubioSeq before and after the application of filters described in sections 16.2 and 16.3

		RUbioSeq vs. Cortex			Cortex vs. RubioSeq		
	**Variant Type**	**Number of mismatches**	**Number of matches**	**Discordance**	**Number of mismatches**	**Number of matches**	**Discordance**
**Before**	Hom_RR	8415	6889578	0.12%	206309	6889578	2.91%
	Het_RA	396672	2931891	11.92%	2524	2931891	0.09%
	Hom_AA	4658	2485185	0.19%	201355	2485185	7.49%
	Het_AA	443	0	0.00%	0	0	0.00%
**After**	Hom_RR	1730	5838711	0.03%	56374	5838711	0.96%
	Het_RA	110393	2677788	3.96%	735	2677788	0.03%
	Hom_AA	661	2046824	0.03%	55675	2046824	2.65%
	Het_AA	0	0	0.00%	0	0	0.00%

18.3.2 Allele Balance

For sequencing data, heterozygous SNPs should have an allele balance around 50%, meaning that 50% of the reads should support the reference allele while the other 50% of the reads should support the alternative allele. Thus the reads that support the reference (or alternative) allele should follow a binomial distribution (D, 0.5), where D is the average read depth. Skewness of the allele balance distribution might be due to cross-species contamination [e.g [110]], assembly-based artefacts not detected by Cortex or false positive SNP calls by Rubioseq.

Our results indicate that the allele balance of heterozygous calls and singletons follows a binomial distribution with mean and mode being approximately 0.5 in both the RUbioSeq and Cortex datasets (Table S35), although the two distributions are slightly different around the tails as reflected in the QQ-plots (Figure S38), indicating slightly larger allele imbalance in the RUbioSeq dataset. This difference reflects the different compromise between sensitivity and reliability reached by both methods. Consequently, Rubioseq is able to detect more heterozygous genotypes but some are based on calls with biased read coverage.

Table S35. Summary statistics of the allele balance for heterozygous genotypes (-H) and singletons (-S) in both datasets.

Caller	Min.	1st Qu.	Median	Mean	3rd Qu.	Max.
Rubioseq-H	0.05	0.44	0.51	0.51	0.58	0.94
Cortex-H	0.09	0.44	0.50	0.51	0.57	0.90
Rubioseq-S	0.09	0.44	0.50	0.51	0.57	0.87
Cortex-S	0.14	0.44	0.50	0.50	0.57	0.79

Min = minimum allele balance; 1st Qu = first quartile ; 3rd Qu = third Quartile; Max= maximum allele balance;

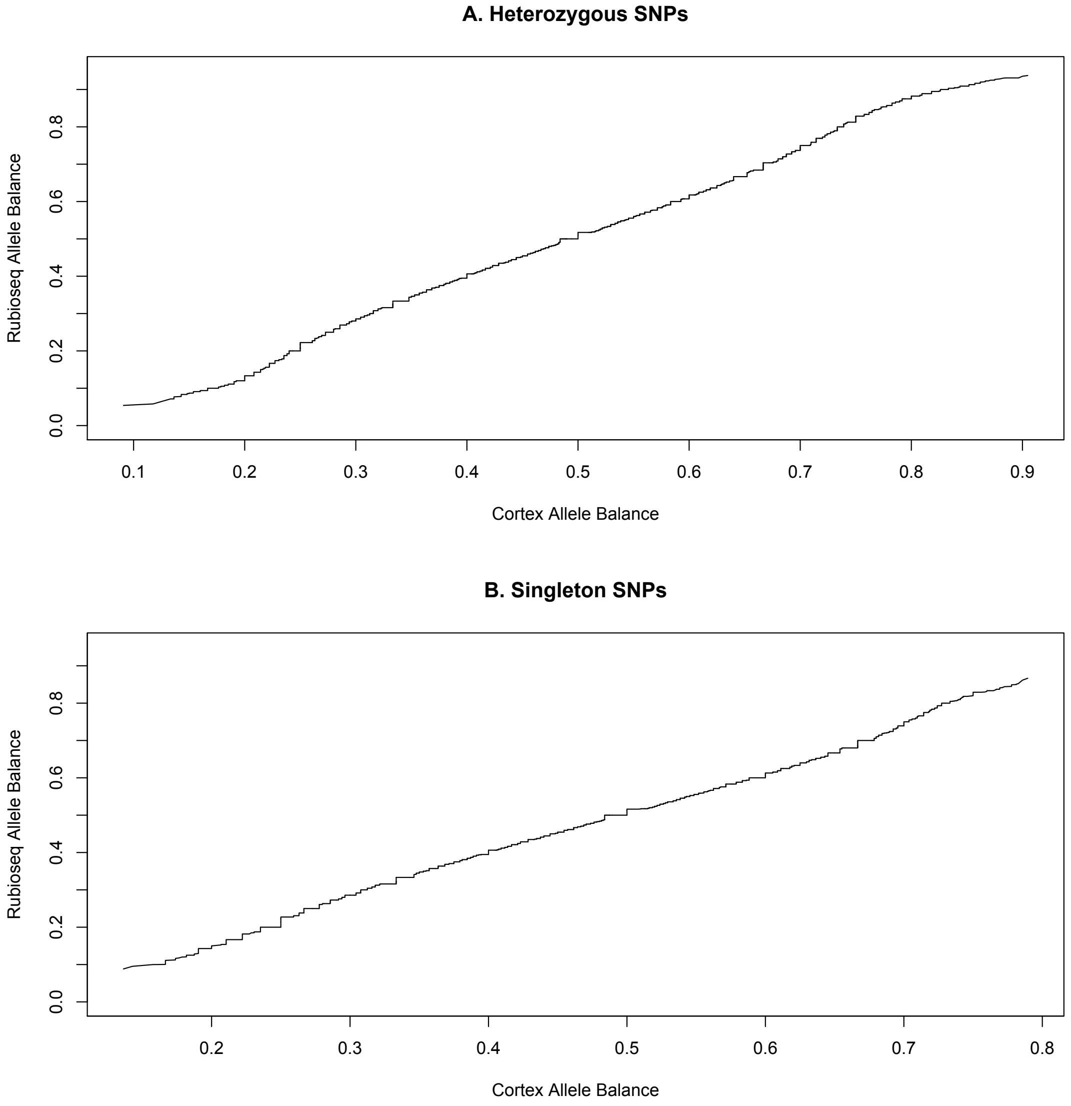

Figure S38. Quantile-quantile plot comparing the probability distribution of both dataset. A: comparison of allele balance distribution for all heterozygous calls. B: comparison of allele balance distribution for all singleton calls.

18.3.3 Minor Allele Counts

In order to better characterize possible biases affecting the population genomics analyses, we also examined the Minor Allele Count distribution for each dataset in the sample pool (Figure S39). As expected, the frequency spectrum of the cortex dataset shows a deficit of singletons in comparison to the RUbioSeq dataset. This in terms of subsequent analyses could have an impact in frequency-based estimates of population parameters and is likely to influence the delimitation of runs of homozygosity and linkage-disequilibrium blocks which are more likely to be broken with a more dense SNP dataset.

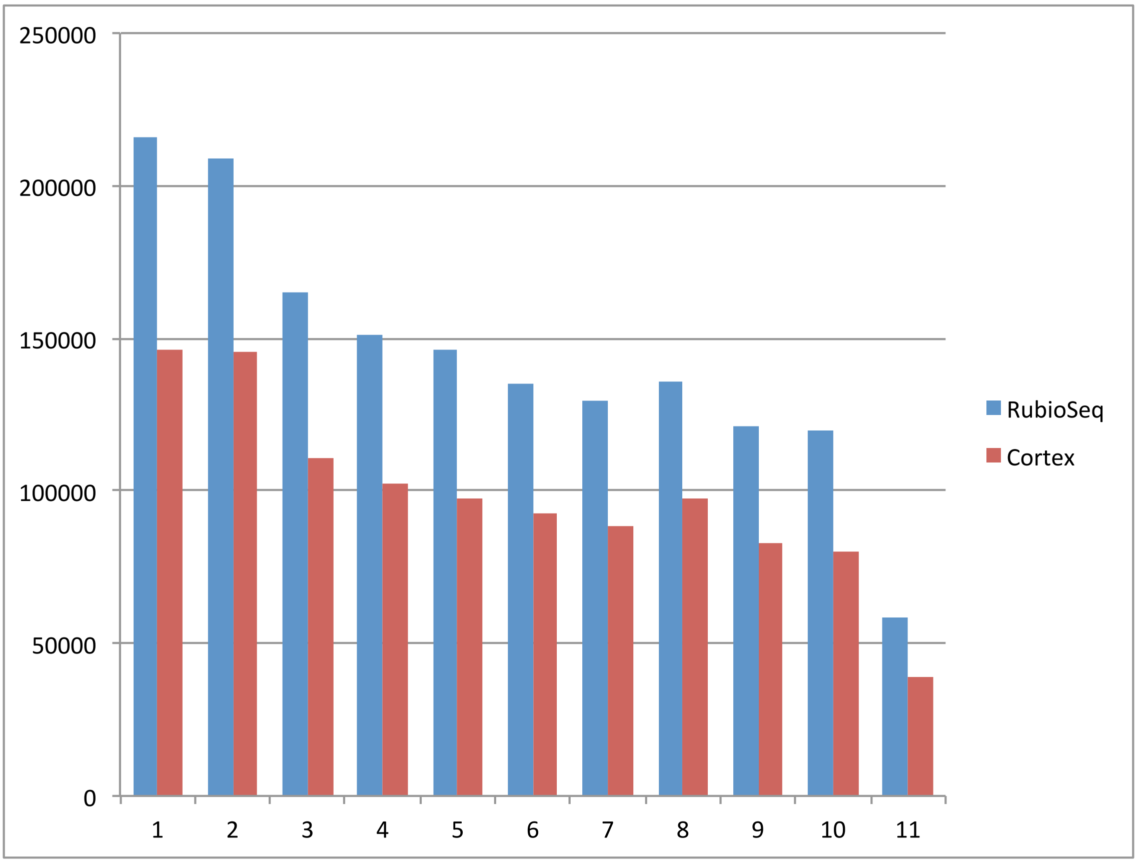

Figure S39. Comparison of Minor Allele Counts between RUbioSeq and Cortex SNPs

18.4 SNPs selection and validation

1536 SNPs were selected among Iberian lynx variants identified by both RUbioSeq and cortex and genotyped in an Illumina GoldenGate technology for

further validation. SNPs with more than two alleles and genotype confidences below 4 were discarded and a list of 1,082,931 SNPs was evaluated with the Illumina assay design tool, ADT (http://support.illumina.com/array/array_software/assay_design_tool.ilmn). ADT produces a score that is related to the likelihood an assay will convert. Only SNPs with an ADT score ≥ 0.8 and MAF ≥ 0.4 in the global Iberian lynx sample were considered. SNPs were chosen to be widely distributed along the genome. In a first round of selection we considered a minimum inter-SNP distance of 1 Mb, and a distance of 0.5 Mb from the end of the scaffolds. Thus in this first round, only scaffolds longer or equal to 1 Mb were considered. A second round of selection was performed reducing the inter-SNP distance to 0.6 Mb, then selecting them in scaffolds between 0,6 Mb and 1 Mb. When a genomic region harboured several SNPs meeting these criteria, preference was given to the one with the highest ADT score. Around half of the SNPs selected could be localised on the cat genome and these were distributed in all chromosomes at fairly homogeneous distances among them. We considered that this distribution met our initial criteria and all 1,536 SNPs were definitely incorporated into the assay design. The assay for the selected 1,536 SNPs was designed and manufactured by Illumina and used to genotype 384 Iberian lynx samples. 1494 markers rendered good genotypes, what translates into a conversion rate of 97,3%. This validated SNP set becomes a permanent resource for further population genetic studies and for the genetic monitoring and management of the species.

19 Runs of Homozygosity (ROH) and individual Inbreeding

Runs of Homozygosity (ROH) refer to contiguous blocks of homozygous genotypes in individual genomes. The extent and frequency of ROH inform on the ancestry of an individual and its population, with longer runs due to related parents transmitting identical haplotypes to their offspring, i.e. recent inbreeding, and shorter runs being indicative of older bottlenecks and inbreeding. Recent studies indicated that the proportion of an individual genome covered by long ROHs (F_{roh}) is likely to be the most powerful method for

detecting inbreeding effects from among several alternative estimates of F [207]. Here we have considered ROH of at least 1 Mbp long to estimate individual inbreeding (F_{roh}) in 11 Iberian lynx (Table S36) [110] and compare these estimates to those derived from the observed homozygosities and allele frequencies (F_{h}). Both, F_{h} and F_{roh} have been estimated with VCFTools v0.1.10 [208].

We first compared the distribution of the extent of the genome covered by ROH of different size classes in the different Iberian individuals (Figure S40). DON individuals showed a larger fraction of the genome covered by long ROH (> 1Mb) than all SMO individuals, with the exception of Candiles (A001), the inbred individual selected for the reference genome; this indicates a higher incidence of inbreeding due to recent consanguineous mating in this population. Medium length (100 Kb-1Mb) ROH are also more abundant in DON than in SMO, consistent with its lower effective size in the recent past, since the two populations became effectively isolated. Finally, the extent of the genome covered by shorter ROH (10Kb-100Kb) is similar in all individuals and probably reflects a shared history of low population size in the more distant past, when both populations were part of a single Iberian lynx metapopulation (Figure S40).

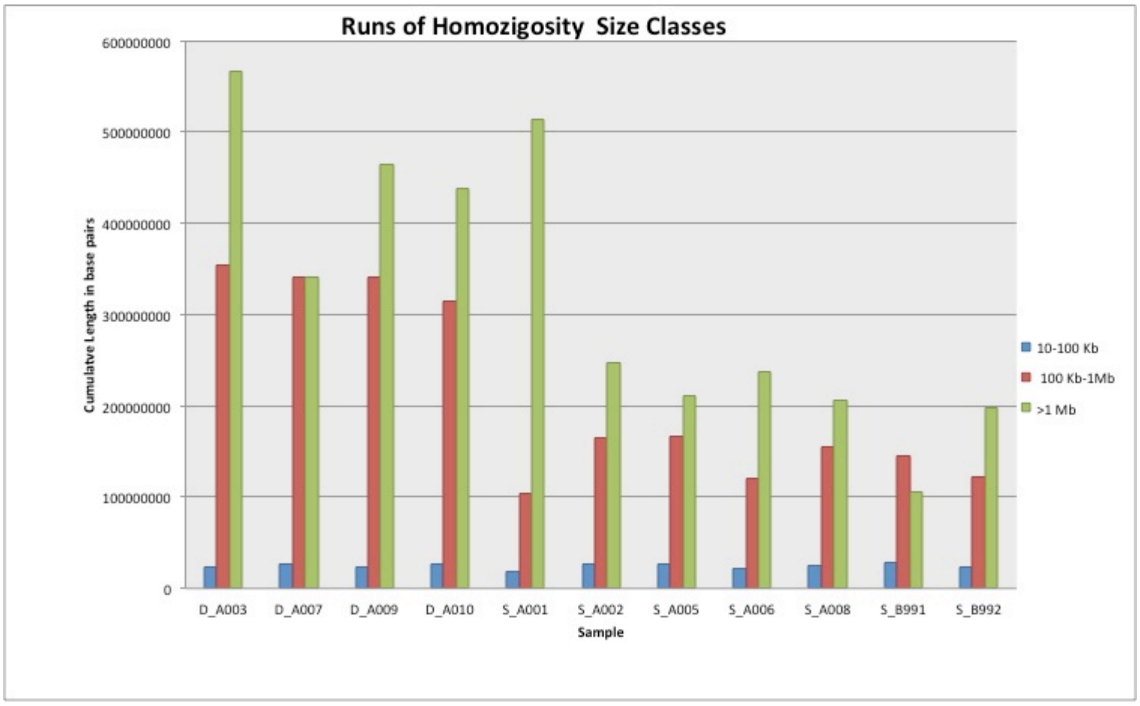

Figure S40. Accumulated length of the genome covered by ROH of different sizes in the Iberian lynx individuals. The letter *D* (Doñana) or *S* (Sierra Morena) preceding the individual code denotes the population of origin.

Estimates of individual inbreeding based on long (> 1Mb) ROHs were highly correlated with those obtained from allelic frequencies (Table S36, Figure S41; R^2=0.92, p-value=5.61x10^{-5}). The average F_h is 0.47 in DON and 0.06 in SMO (t-test, p = 9.3x10^{-9}), while the average F_{roh} is 0.32 in DON and 0.16 in SMO (t-test, p = 0.00211).

Table S36. Estimates of the inbreeding coefficient for each Iberian lynx individual. F_{roh} is based on the accumulated length of ROH at least 1Mbp long considering all scaffolds with at least 1Mb (total examined length of 1,516,323,743 bp). F_h is the allele-frequency based estimate obtained using VCFTools.

Individual	# ROHs	# ROH $_{>= 1Mb}$	Accumulated Length ROH $_{>= 1 Mb}$ (Mb)	F_{roh}	F_h
D_A003	2064	349	566.65	0.374	0.547
D_A007	2184	303	468.04	0.309	0.477
D_A009	2111	287	465.07	0.307	0.422
D_A010	2010	273	438.43	0.289	0.428
S_A001	1322	267	513.06	0.338	0.233
S_A002	1614	149	247.54	0.163	0.092
S_A005	1695	121	211.24	0.139	0.067
S_A006	1415	132	237.43	0.157	0.039
S_A008	1648	128	206.29	0.136	0.039
S_B991	1717	65	104.95	0.069	-0.029
S_B992	1386	113	197.60	0.130	-0.048

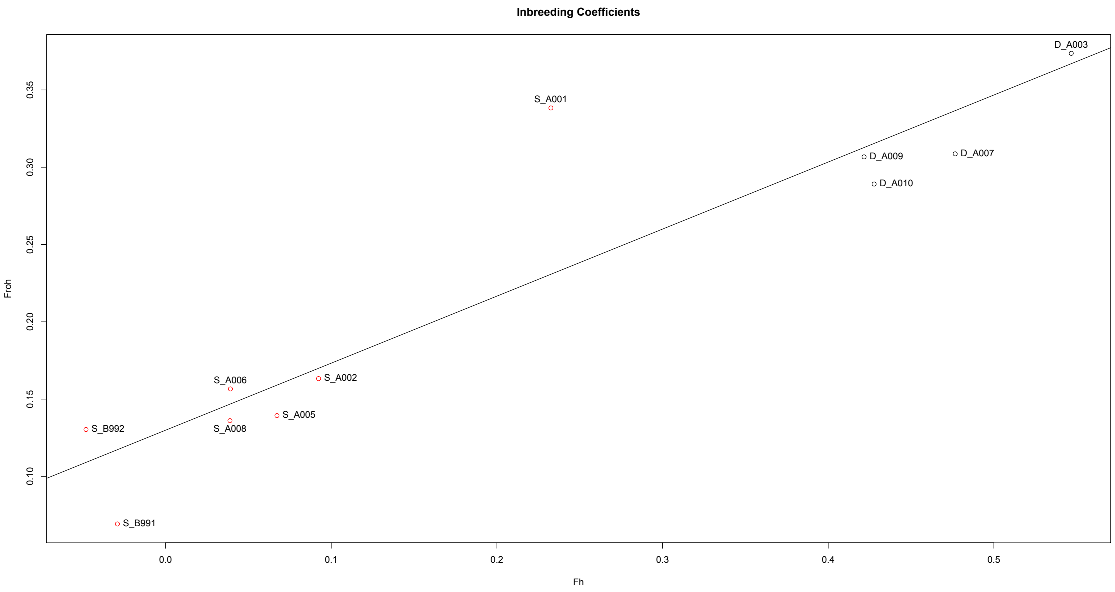

Figure S41. Correlation between F_{roh} and F_h estimates of individual inbreeding coefficient.

In order to find homozygous regions that may be associated with the low fitness of the species, we looked for overlapping ROH between individuals and populations. Using BEDtools 2.15.0 [122], we intersected the ROHs of all the Iberian lynx samples finding 1,590 homozygous regions common to all Doñana samples and 22 common to all Sierra Morena samples. To assess the possible functional impact of the homozygosity of these regions we used FatiGO [209] to evaluate if there is any significant functional enrichment in each transcript list. No GO term was found to be significantly enriched for genes within any of these high homozygosity regions.

20 Genomic averages of population genetics parameters

We have estimated genome-wide averages of genetic parameters for diversity and differentiation in the Iberian lynx based on two SNP datasets, Rubioseq and Cortex. All these analyses were conducted mainly using VCFTools v0.1.10 [208] and custom perl scripts.

20.1 Genetic relationships among individuals

In order to visualize the variation among genotypes in the Iberian lynx sample we used the *adegenet* package [210] to perform a Principal Component Analysis (PCA) on the 11 genotypes. The number of retained factors was two, as the first two *eigenvalues* explained approximately 50.6 % of the total variation. While the first axis neatly separates both populations, the second axis shows intra-population differences within Andújar, reflecting a higher genetic diversity than in Doñana (Figure S42a). This general pattern was confirmed by a neighbour-joining tree based on an Euclidean distance that takes into account the proportion of unshared alleles between individuals (Figure S42b). Results obtained for the cortex dataset are shown, but similar patterns were obtained with the RUbioSeq dataset.

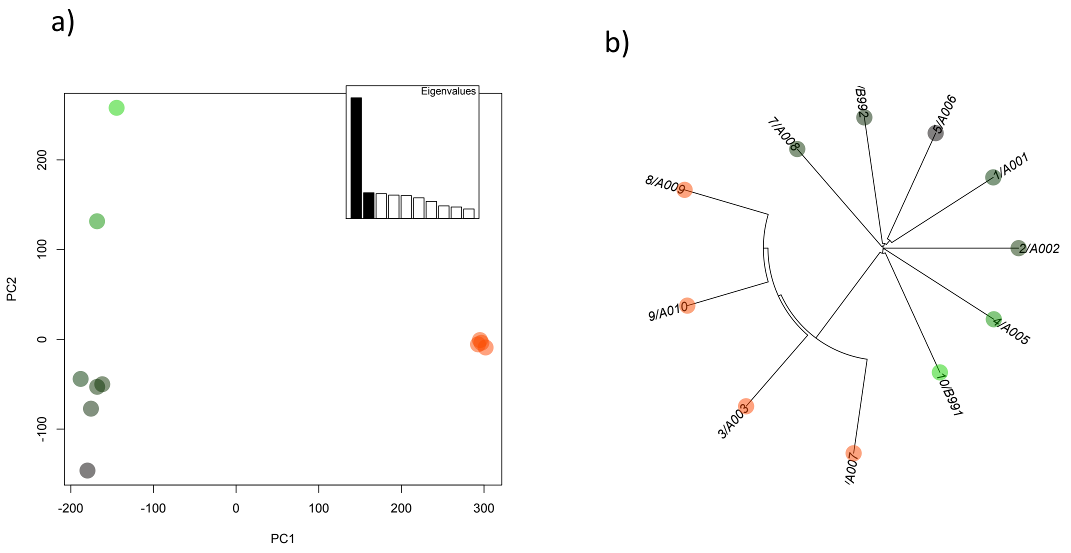

Figure S42. Patterns of variation among Iberian lynx individuals as depicted by FCA analyses (a) and Neigbour-Joining tree based on the Euclidean distance between individuals (b). Each dot represents an individual lynx, with green and orange individuals belonging to Andújar and Doñana populations, respectively.

20.2 Genetic differentiation

We have chosen the Weir & Cockerham's estimator of F_{ST}, the fixation index (F_{ST}) measuring the level of population differentiation on the basis of observed allele and heterozygote frequencies, as is more appropriate for small sample sizes [211, 212]. The level of genetic differentiation between Doñana and Andújar Iberian lynx populations is moderate to high and similar for the two datasets (F_{ST} = 0.22, SD= 0.28 for RubioSeq SNPs; F_{ST} = 0.23, SD= 0.29 for cortex SNPs; Figure S43). These SNP-based genome wide estimates are lower than a recent estimate based on 36 microsatellite loci [114] (F_{ST} = 0.42), although this value is still comprised within the 95% confidence interval of our estimates. Moderately high differentiation between the two remnant populations is consistent with a history of intense genetic drift and complete genetic isolation for the last few decades [114].

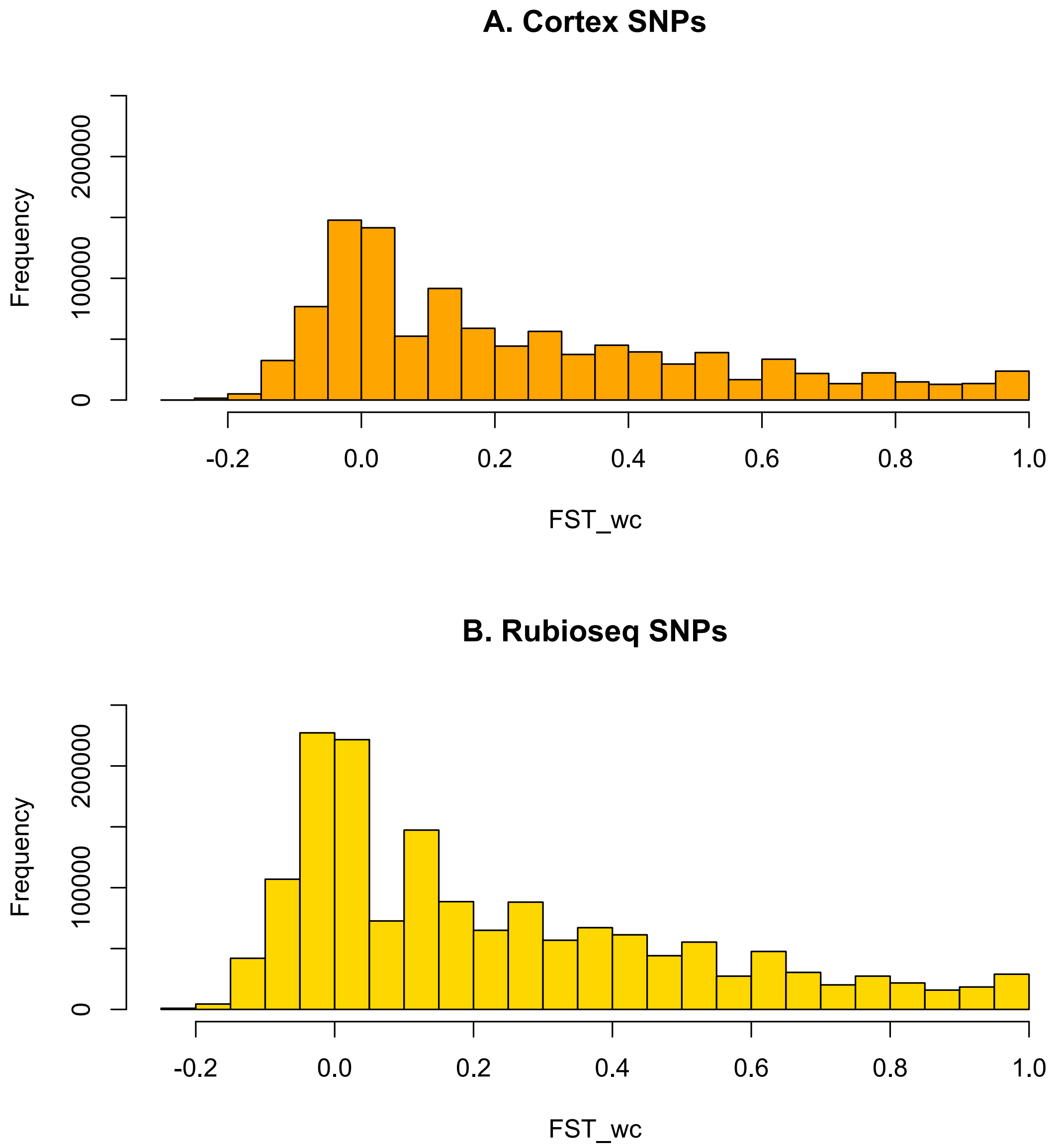

Figure S43. Distribution of Weir & Cockerham's F_{ST} for a) the cortex and b) the RUbioSeq datasets

20.3 Genetic diversity

Genetic diversity was measured using three different parameters: the number of segregating sites, the observed heterozygosity (*Ho*) and the expected heterozygosity (*He*) at segregating sites. From these parameters, we estimated the Watterson's estimator (W_Θ) of the population mutation rate ($\Theta = 4N_e\mu$), which is based on the observed number of segregating sites, and the observed and expected heterozygosity per site, the latter being equivalent to the nucleotide diversity (π), a second estimator of Θ. We report the estimates

obtained from both the RUbioSeq and Cortex SNPs, but the per site estimates required an estimate of the number of callable but invariant sites which was only available for the RUbioSeq dataset (2,020,145,224 total callable sites). In order to allow comparison with other species (see below), we also calculated the observed heterozyogosity rate (equivalent to the SNP density for individual genomes) with respect to the size of the lynx assembly (2,361,206,848 after removing ambiguous sites). Genetic parameters are estimated for each of the two remnant populations (Doñana, DON; Andújar-Sierra Morena, SMO) and for the species as a whole (Pool). As expected, diversity values per variant were slightly lower for the Cortex dataset in agreement with its more conservative call of heterozygous genotypes, while the trend among populations is consistent between both datasets. The lowest diversity values are observed in Doñana population, that shows just 56% and 53% of the observed and expected heterozygosity found in Sierra Morena, respectively (Table S37), once again reflecting its smaller size and longer time since isolation from other lynx populations.

Table S37. Genetic diversity statistics estimated with the two SNP datasets for the whole species (Pool) and for each of the two remnant populations of Doñana (DON) and Andújar (Sierra Morena, SMO)

	**RubioSeq SNPs**			**Cortex SNPs**		
	**Pool**	**SMO**	**DON**	**Pool**	**SMO**	**DON**
N (chromosomes)	22	14	8	22	14	8
Number of SNPs	1,587,509	1,383,709	625,552	1,072,340	937,143	420,712
Ho per variant	0.266	0.317	0.178	0.244	0.291	0.163
He per variant	0.336	0.316	0.167	0.337	0.312	0.163
Watterson's Θ (%) [2]	0.022	0.022	0.012	n.d.	n.d.	n.d.
Het. rate (%) [1]	0.018	0.021	0.012	0.011	0.013	0.007
Ho per site (%) [2]	0.021	0.025	0.014	n.d.	n.d.	n.d.
He per site (π) (%) [2]	0.026	0.025	0.013	n.d.	n.d.	n.d.

[1] assuming 2,361,206,848 sites (size of the assembly)
[2] assuming 2,021,732,768 callable sites

20.4 Comparison to other mammalian species

We re-estimated the observed heterozygosity rate using the 1,662,481 SNPs obtained with cortex for the pooled set of individuals, including the Eurasian lynx (inter-specific dataset), to allow the comparison between the diversity of

both lynx species. The average number of heterozygous SNPs per Mb observed for Iberian lynx was similar to that obtained using the Iberian lynx intraspecific variant calling (102 heterozygous SNPs per Mb), while for the Eurasian lynx we estimated a significantly larger, but still reduced heterozygosity rate of 279 SNPs/Mb. The Iberian lynx heterozygosity rate is only about one third (36.6%) of that present in Eurasian lynx (279 SNPs/Mb), similar to that estimated for the inbred domestic cat individual (121 SNPs/Mb; Figure 4C, main text), and lower than that of other severely endangered species like the snow leopard (*Panthera uncia*; 231 SNPs/Mb), the Tasmanian devil (*Sarcophilus harrisii*; 320 SNPs/Mb), the Amur tiger (*Panthera tigris altaica*; 486 SNPs/Mb), or the giant panda (*Ailuropoda melanoleuca*; 1120 SNPs/Mb). In fact, the Iberian lynx's is the lowest heterozygosity rate reported to date for any mammal, and is also lower than those reported for endangered avian species, including the crested ibis (*Nipponia nippon*; 430 SNPs/Mb) or the white-tailed eagle (*Haliaeetus albicilla*; 400 SNPs/Mb) [213]. Although these comparisons must be taken with caution because the methodological differences among studies (different filtering criteria, sequence read depth and sample sizes) can influence the reported estimates, they should still reflect gross differences among species diversity.

21 Variation and divergence at coding sequences

Low effective population size is expected to result in a reduction of diversity at both neutral and non-neutral sites, but it can additionally affect non-neutral variation through a decrease in the efficiency of natural selection. Such relaxation of purifying selection may lead to an increase in the frequency of slightly deleterious mutations, some of which can eventually reach fixation and contribute to the genetic load of the population. Increased genetic load in combination with high rates of inbreeding can compromise the viability of endangered species and populations. In order to assess the effect of bottlenecks in adaptive variation we analysed patterns of polymorphism and divergence at coding sequences in the Iberian lynx genome.

We focused on coding sequences (CDS) in regions of synteny to cat and used the variant sets defined with RUbioSeq in mappings to the Iberian lynx reference and to the domestic cat reference (Suplementary Section 16.2). We recorded the number of sites that are callable (*s*), the number of SNPs segregating in Iberian lynx populations (*P*), and the number of fixed differences with respect to cat (*D*). Synonymous and non-synonymous variants or substitutions as annotated in SNPeff were counted (P_s, P_n, D_s, D_n), and per-site synonymous and non-synonymous nucleotide diversity (π_S, π_N), and substitution rates (d_N, d_S) were approximated by assuming that ¾ of all of the sites are non-synonymous. Non-synonymous to synonymous ratios were then calculated for the counts of variants (P_N/P_S), their nucleotide diversity (π_N/π_S), and for the substitution rates (d_N/d_S). Finally, we calculated a neutrality index (*NI*), defined as *(Pn/Ps)/(Dn/Ds)*, to assess the direction and degree of departure from neutrality. Under the assumption that synonymous mutations are neutral, *NI* >1 indicates an excess of amino acid polymorphisms due to the relaxation of purifying selection, and *NI* < 1 indicates an excess of non-synonymous divergence due to the action of positive selection. Only CDSs with more than 200 callable sites were used. The genomic averages of π_S, π_N, d_N, and d_S were calculated by averaging across CDS while weighting by *s*, and genomic ratios were calculated from these averages. Genome-wide NI was calculated by summing the P_s, P_n, D_s and D_n across CDS for comparative purposes [214], but we also used the alternative measure NI_{TG}, a recently proposed estimator that avoids the biases associated to the classical *NI* [215].

Non-synonymous variants were almost as abundant as synonymous variants (*Pn/Ps*=0.90; Table S38). The genomic average of π_S was extremely low (0.028%), with 11,431 (81.5%) CDSs showing zero synonymous diversity, but the ratio π_N/π_S was quite high (0.29). Regarding divergence, the non-synonymous to synonymous ratio was lower for the number of observed fixed differences (*Dn/Ds*=0.64) than for variants (*Pn/Ps*=0.90), resulting in a genomic *NI* well above 1 (1.40); the unbiased NI_{TG} estimate was even higher (1.56). d_N/d_S ratios were relatively high (0.21), as also observed in the branch-specific analyses of substitution patterns (Supplementary Notes 16.3) and phylogenomic analyses (Section 13.7). Autosomes statistics were similar to global, however the X

chromosome yielded increased π_N/π_S, d_N/d_S, NI and NI_{TG} (0.38 and 0.30, 1.44 and 2.61, respectively; Table S38).

Table S38. Summary statistics of diversity and divergence in coding sequences in Iberian lynx.

	Global	Autosomes	ChrX
**CDS (n)**	14,028	13,550	476
**Sites (n)**	17,314,587	16,900,646	412,781
**πs (%)**	0.0275	0.0279	0.0095
**πN/πS**	0.286	0.285	0.378
d_N/d_S	0.212	0.210	0.301
**Pn/Ps**	0.90	0.89	1.30
**NI**	1.41	1.42	1.44
$\mathbf{NI_{TG}}$	1.56	1.56	2.61

These results are remarkably different from those obtained for species with large population sizes like *Drosophila simulans* [216], *Ciona intestinalis* [217], or rabbits, *Oryctolagus cuniculus* [218], but similar to values observed for humans [219], and closest to those reported for the endangered giant Galápagos tortoise [220]. It must be noted though that these two latter species still harbour much higher diversity than the Iberian lynx (Table S39). Although the comparison among species and studies is hampered by possible biases arising from the different criteria used for variant and gene filtering, methods of calculation, and sample size (studies with large samples are more likely to include low frequency non-synonymous variants), the large differences observed can be safely attributed to differences in demography and evolution among species.

Table S39. A comparison of genomic statistics of coding variation across species with different demography.

Species	Species profile	#genes	πs (%)	πN/πS	dN/dS
Ciona intestinalis	Highly abundant, invertebrate	1602	5.70	0.046	0.074
Drosophila simulans	Highly abundant, invertebrate	10996	3.27	0.085	0.115
Oryctolagus cuniculus algirus[1]	Abundant, mammal	3433	0.807	0.053	0.083
Homo sapiens	Old bottleneck	6530	0.164	0.241	0.229
Chelonoidis nigra[2]	Endangered island endemic, reptile	814	0.190	0.310	0.140
Lynx pardinus	Bottlenecked, mammal	14028	0.028	0.286	0.212

[1] autosomal, using unweighted averages across genes
[2] diversity data correspond to their A3 stringency

22 X chromosome versus autosomes genetic diversity

Comparisons of the genetic diversity between the X chromosome (X) and autosomes (A) can inform on historical demographic changes and on differences between processes affecting differently males and females [221]. In a scenario of constant population size and identical distribution of reproductive output between males and females, the effective population size (Ne) of X is expected to be three-quarters of that of the autosomes, a ratio that should translate to genetic diversity at mutation-drift equilibrium. Deviations from this ratio can be generated by sex-biases in migration, reproductive success or mutation rates, by differences in natural selection patterns due to the exposure of recessive X-linked variants in hemizygous males, and by changes in population size. In order to explore the possible impact of bottlenecks on chromosomal patterns of diversity in Iberian lynx we estimated the nucleotide diversity (π) normalized by divergence (D) in autosomes and the X chromosome for both intergenic (IGS) and synonymous coding sites (CDS). The normalization controls for variation in mutation rates, and the comparison between IGS and CDS regions allows the gauging of the relative contributions of neutral and selective forces.

We defined the set of coding and noncoding regions used for these analyses by applying a series of filters to exclude error-prone and genic regions. Regions filtered out included repeats generated by the RepeatMasker, low complexity regions identified by DustMasker, centromeres and telomeres along with 2 MB flanking regions, and pseudoautosomal region 1 (PAR1) in X chromosome (first 6Mb). Intergenic regions and CDS were delimited according to the current version of lp23 annotation (LYPA23C.PCG.ff.gff3). We considered only genomic regions with conserved synteny and correctly aligned to the domestic cat genome (felCat 6.2), so that we could assign lynx regions to specific chromosomal locations and obtain estimates of cat-lynx divergence. Finally, only callable sites (variants plus confident invariant sites as defined in the variant calling process) were considered.

With intergenic sites we delimited 10,432 windows with a minimum of 50K informative sites and a maximum physical size below 200Kb. For each of these windows we estimated the nucleotide diversity (π, the mean number of pairwise

differences per site) and the divergence to cat (D, the observed fraction of fixed differences). The ratio between the two (π/D) was used as a measure of diversity normalized by mutation rate. Synonymous nucleotide diversity was estimated for a total of 12,103 CDSs with more than 100 sites by assuming an overall synonymous to nonsynonymous ratio of ¼, and was normalized by the observed rate of synonymous substitutions (dS). Average estimates were obtained for autosomes and the X chromosome and for each of the three possible populations: global (N=11), Andújar (N=7) and Doñana (N=4). Standard errors were calculated by bootstrapping over windows or CDS as implemented in the *boot* package for R [222, 223], to account for the correlation among nearby sites due to linkage disequilibrium (LD).

The average X/A diversity ratios estimated for each Iberian lynx population were 0.35 (SE=0.02), 0.29 (SE=0.02), 0.38 (SE=0.03) for IGS, and 0.19 (SE=0.07), 0.21 (SE=0.08) and 0.13 (SE=0.07) for CDS (silent sites) for the global, Andújar and Doñana populations, respectively (Figure S44). IGS X/A diversity values in lynx are substantially lower than the 0.75 expected at equilibrium and lower than the lowest ratio reported for human populations (0.60-0.62 for Asian populations; [224]), and also lower than the ratios in Bornean Orang-Utan (0.50) or Indonesian–Malaysian *Macaca fascicularis* (0.54). Lynx ratios are, however, similar to those reported for Eastern gorillas (0.35) or Philippine *Macaca fascicularis* (0.29), which have been attributed to recent reductions in population size [110, 225].

These results show a strong reduction of diversity in the X chromosome diversity relative to autosomes in Iberian lynx, consistent with theoretical predictions for a recent demographic bottleneck. The observation that X/A diversity is even more reduced in CDS regions than in the IGS, suggests an additional contribution of a differential effect of purifying selection between autosomes and chromosome X through the exposure of deleterious recessive alleles in males, which may be more intense in the most inbred Doñana population.

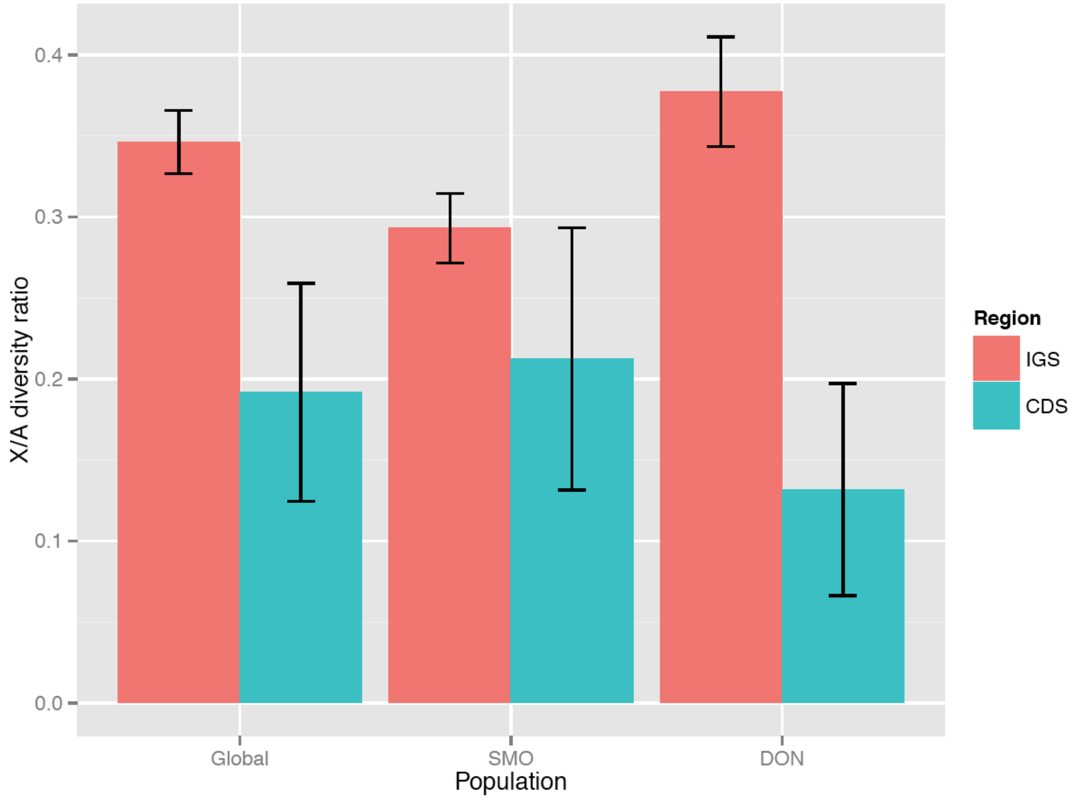

Figure S44. Autosomal and chromosome X ratios of normalized nucleotide diversity for intergenic (IGS) and coding (CDS) regions in the three Iberian lynx populations.

23 Patterns of diversity across the genome

23.1 Genomic windows with extremely low or high diversity within the Iberian lynx genome

Neutral theory predicts that the diversity at a neutral locus is determined by the mutation rate and the effective population size (i.e. genetic drift). Directional and purifying (background) natural selection reduce diversity at selected loci, while several forms of balancing selection can promote diversity values above those predicted under neutrality, and these effects can extend to linked loci through hitchhiking. Local genetic diversity is thus expected to vary along the genome as a result of the interplay between mutation, selection, genetic drift and recombination. In recently bottlenecked populations, the increase in genetic drift is expected to reduce diversity globally and randomly across regions, but ongoing balancing selection could temper, and positive and background selection

could enhance, the loss of diversity at selected loci and linked sites, especially in regions of low recombination beyond neutral expectations. In order to characterize genomic patterns of diversity in the Iberian lynx genome we identified genomic windows with extremely low or extremely high levels of heterozygosity, and then performed a GO-enrichment analysis on the overlapped genes.

A total of 2,021,732,768 callable sites (PASS and QUAL > 50) were identified with RUbioSeq. After removing all sites within repeats and low-complexity regions the total number of *informative sites* in the assembly was 1,123,189,703. We divided the genome into 150 Kb-long non-overlapping windows with at least 50,000 informative sites. For a finer-scale analysis we also defined windows of 20 Kb and at least 5,000 informative sites. For each window we calculated the Z-transformed per-site average of the observed heterozygosity (*H*) in each Iberian lynx population (Doñana, DON; Andújar-Sierra Morena, SMO) and in the species as a whole (POOL). The Z-transformation consists in expressing the observed parameter values in units of standard deviation from the global mean, as described below:

$$Z_H = \frac{H - E[H]}{sd[H]}$$

The genome-wide empirical distribution of the Z-transformed parameter was examined and all the windows with a Z value higher than the 99.9th or equal or lower than the 0.1th percentile were identified as outliers (Table S40). We then tested whether the genes within outlier windows were significantly enriched for particular cellular components, biological processes or molecular functions by performing a Gene Ontology enrichment analyses using FatiGO [209], as implemented in Babelomics 4.3 [226].

No GO term was significantly enriched among the genes included in the Doñana low Z_H windows, indicating that the large effect of drift in this population has been mostly random and has affected genes more or less uniformly. Contrarily, windows of low Z_H in both populations or in the species as a whole were significantly enriched for several GO terms, including some related to nerve and muscle development and function (Additional file 2, Datasheet S8). These might

correspond to regions most impacted by genetic drift, or regions of low recombination under strong selective constraint or recent positive selection. No GO terms were significantly enriched in SMO, but in both DON and POOL high Z_H outliers were enriched for genes for olfactory receptors (Additional file 2, Datasheet S8), what could indicate the action of balancing selection, although unrecognized duplications could be influencing this result, as discussed below.

Table S40. Genomic windows showing extreme diversity in Doñana (DON), Sierra Morena (SMO) or the species as a whole (POOL) and the number of genes they contain.

**Outlier**[1]	**150 Kb Windows**		**20 Kb Windows**	
	No. outliers	No. genes	No. outliers	No. genes
High Ho in DON	13	19	104	43
High Ho in POOL	13	21	104	36
High Ho in SMO	13	10	104	34
Low Ho in DON	2492	2898	3591	4319
Low Ho in POOL	100	102	487	486
Low Ho in SMO	123	122	535	518

[1]*High* means that the z-transformed value is above the 99.9th percentile of the z-empirical distribution of observed heterozygosity; *low,* that the z-value is equal or lower than the 0.1st percentile

23.2 Genomic windows of extreme interspecific difference in heterozygosity

To gain further insights on how the Iberian lynx decline has affected genomic variation across chromosomes or gene functions, we redid the diversity outlier analyses with the syntenic windows that were defined for the analyses of substitution patterns (Section 16). The use of syntenic windows allowed us to map lynx scaffolds onto cat chromosomes and analyse the patterns of diversity across chromosomes (Section 22) and/or chromosomal regions (Section 23). For this analysis we also included variation data obtained for the Eurasian lynx individual and focused on the detection of windows with highest or lowest heterozygosity difference between the two lynx species.

For autosomal windows with more than 10,000 informative sites we calculated the observed heterozygosity per site as the ratio of heterozygous positions to the total number of callable sites. We tried to identify those genomic regions that have been most and least impacted by genetic drift by comparing

heterozygosities between species (Iberian vs. Eurasian) or populations (Doñana vs. Andújar). We defined: i) windows with null heterozygosity in one species/population but non-null heterozygosity in the other, and ii) windows with extreme differences in standardized heterozygosity between species. We then tested whether these extreme windows were enriched at particular chromosomal regions (e.g. subtelomeric regions, delimited as <5 Mb from the end of chromosomes), genes or particular GO-terms.

Window-averaged observed heterozygosity largely varied between Iberian populations and between Iberian and Eurasian lynx. Variations were observed also among and along chromosomes. Global averages were highest for the Eurasian, and higher for Andújar than for Doñana Iberian individuals. The Iberian average heterozygosity (0.00020) was only 35.9% of that observed in the Eurasian individual (0.00057), and Doñana's (0.00013) was 54.7% of Andújar's (0.00024). We found wide variation among chromosomes, and subtelomeric regions showed higher average heterozygosity than the rest of the chromosome in both species (66.2% higher in Iberian, 45.1% higher in Eurasian), and the ratio of Iberian/Eurasian heterozygosity was larger for subtelomeric regions (0.406) than for the rest of the chromosome (0.355).

Thirty-one windows showed no variation in Iberian but non-zero variation in the Eurasian individual, and 65 and 4,935 windows had zero heterozygosity only in Andújar and Doñana, respectively. Although some of these regions could originate from Iberian lynx-specific selective sweeps, in view of the recent divergence and demographic history of the species, they are more likely to correspond to regions where genetic drift has driven the complete loss of genetic variation in the Iberian lynx or in any of its two remnant populations. These windows were not significantly enriched or depauparated with respect to the presence of genes, but tended to be under-represented in subtelomeric regions, although statistical significance is only reached in the Doñana population (Table S41).

We also analysed the distribution of the difference in standardized heterozygosity (i.e. heterozygosity expressed as units of standard deviation from the mean) between Eurasian and Iberian lynx ($\Delta Z_H = Z_{H\text{-}EL} - Z_{H\text{-}IL}$). For this

comparison we excluded windows with null heterozygosity in the Eurasian lynx individual, as this might correspond to identity-by-descent (IBD) regions generated by recent inbreeding and do not necessarily reflect species-wide heterozygosity. Windows within both 2.5% lowest ($Z_{H\text{-}EL} << Z_{H\text{-}IL}$) and 2.5% highest ΔZ_H ($Z_{H\text{-}EL} >> Z_{H\text{-}IL}$) were both significantly depleted of genes, and windows with smallest difference ($Z_{H\text{-}EL} << Z_{H\text{-}IL}$) were more likely to be in subtelomeric regions (Table S41).

Table S41. Syntenyc genomic windows with zero heterozygosity in both or each of the two Iberian lynx populations (DON, Doñana; SMO, Andújar-Sierra Morena), or extreme differences in standardized heterozygosity between Eurasian lynx (EL) and Iberian lynx (IL). The number of windows containing more that 10000 genic sites or falling within 5Mb of telomeres are reported, with asterisks denoting significant enrichment within the corresponding windows (t-test against the rest of the windows).

Windows	N	With genes (%)	Subtelomeric (%)
H=0 in IL (SMO and DON), H>0 in EL	31	17 (54.8)	1 (3.2)
H=0 in SMO, H>0 in DON and EL	65	27 (41.5)	2 (3.1)
H=0 DON, H>0 in SMO and EL	4935	2554 (51.8)	246 (5.0)*
Low ΔZ_H outlier (IL <<< EL)	718	310 (43.2)**	111 (15.5)**
High ΔZ_H outlier (IL >>> EL)	671	276 (41.1)**	49 (7.3)
Overall	20825	10495 (50.4)	1259 (6.0)

* p=.0003; **: p < 0.0001

Windows with extreme ΔZ_H were heterogeneously distributed among chromosomes and often found in clusters, defining extended regions of large heterozygosity difference between the two species (e.g. chrF1:17500000-18200000; Figure S45). In some cases high and low ΔZ_H windows were contiguous (e.g. chrE1:46300000-46500000; Figure S45).

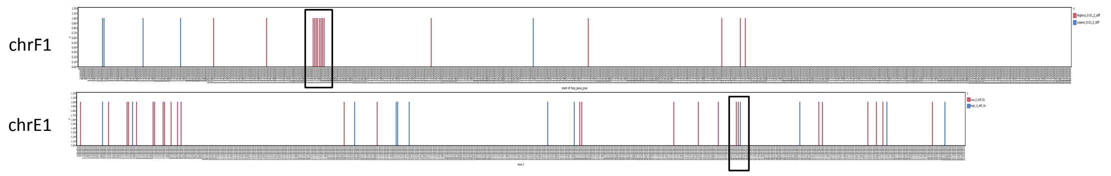

Figure S45. Chromosomal distribution of windows of extremely low (red) or high (blue) difference in standardized heterozygosity (ΔZ_H) between Iberian and Eurasian lynx. ChrF1 and chrE1 are shown to illustrate cases of clustered windows of low ΔZ_H and contiguous high and low ΔZ_H outliers (boxed).

A GO term enrichment analysis indicated that the windows with highest and lowest differences in heterozygosity between the two lynx species were both enriched for genes related to olfactory perception and G-protein signal transduction, while windows with smallest difference were additionally enriched for genes involved in amino acid and glucose transport and the regulation of triglyceride biosynthesis genes, among others (Additional file 2, Datasheet S8).

Higher relative heterozygosity in telomeres has been reported for several species, including humans [227], and the endangered western lowland gorillas [228]. Increased diversity in telomeres has been attributed to an increased recombination rate in these regions, and supports a widely observed correlation between recombination rate and local genetic diversity [229, 230]. In fact, it has been suggested that increased recombination would be positively selected as a mechanism to increase diversity in domesticated species [231], or otherwise small populations [232], specifically when a recombination driver allele is tightly linked to two or more genes with alleles under selection [233]. In support of this hypothesis, modifiers of recombination have been found among the genes with signatures of selection in domesticated species, including domestic cat [234]. Our observation of a higher level of retention of diversity in telomeres in the extremely bottlenecked Iberian lynx supports a prominent role of recombination in maintaining genetic diversity in small populations, either through an associated increase in mutation or through the reduction of the number of sites affected by hitchhiking following background or positive selection.

On the other hand, the olfactory-receptors form the largest multigene family in vertebrates and one of the most genetically diverse in the human genome. Olfactory receptors have been suggested to be under the influence of balancing selection [235], and they often come out as regions of high diversity in whole genome scans, even after partially controlling for paralogy (e.g. [236]). Increased diversity is supposed to enable the recognition of a wider range of odors, and the number of functional OR genes has been suggested to vary in relation to the importance of olfaction for the species, with a low number in human (358) and the highest in elephant (1948) [237], with intermediate numbers in tiger (713)

and domestic cat (667) [234]; in the Iberian lynx genome 935 genes are associated to the GO term "function: olfactory receptor activity ". Increased retention of diversity in Iberian lynx could thus be related to ongoing balancing selection on olfactory perception, although testing this hypothesis will require additional data and analyses.

It must be noted that some regions of extreme apparent heterozygosity may arise from segmental duplications (SD) that were collapsed in the assembly of the reference genome, and extreme differences between species may reflect species-specific duplications. Both telomeres and the olfactory-receptor gene family are characterized by a high incidence of duplications and copy-number variation, and the latter is organized in dense clusters dispersed on several chromosomes, [238, 239]. Windows including SD identified with respect to the cat genome (Section 17) showed indeed generally higher heterozygosity in both lynxes, and windows with SD exclusive of Eurasian lynx tended to show extreme heterozygosity values in this species. Note that while shared or Iberian-specific SD might or might not be collapsed in the Iberian reference genome, Eurasian-specific SDs will all be collapsed. Still, only 21 out of 718 Low ΔZ_H outlier windows (6 EL specific and 15 common to IL and EL), and 26 out of 671 high ΔZ_H windows (6 IL-specific, 14 EL-specific, and 26 both) contained regions identified as segmental duplications with respect to cat. More robust conclusions on the influence of collapsed SD on local heterozygosity will require the analyses of SD and CNV with respect to the reference Iberian lynx assembly on which the genotype calling was made.

24 Linkage disequilibrium

The pair-wise measure of linkage disequilibrium r^2 - the squared correlation coefficient between genotypes in each individual- was estimated between all the SNP in each scaffold and population independently with *VCFTools v0.1.10 [208]*, which provide the same estimate as PLINK [240]. In total we have used the 1, 587,544 SNPs called against lp23 using the RUbioSeq pipeline. We set a maximum threshold of 8,050 SNPs per scaffold to make the r^2 calculation computationally affordable. However, no SNPs were excluded because the

maximum number of SNPs was 8,009 SNPs for scaffold lp23.s00001, the longest with 13,188 Kb. Note that the r^2 estimate is quite robust for distances between SNPs below 2,000 Kb as they comprise 99.11% of the scaffolds (Figure 4D, main text; Figure S46).

We calculated the genomic mean and standard deviation of r^2 for different inter-SNPs distance classes. The genome-wide decay of r^2 was plotted as a function of distance and the point where r^2 reaches 50% of its maximum value was chosen as a comparison point between populations and species [241]. Our analysis indicates that the extent of LD corresponds to 185 Kb in SMO, at least twice the average of domestic cat breeds (96 Kb) and approaching that observed for the highly inbred Burmese cat (249-380 Kb) [241]. Our results suggest an even lower rate of LD decay in DON population, but r^2 might be overestimated due its small sample size. In order to evaluate this bias, we performed the same analysis for 10 random subsamples of 4 individuals from SMO. LD still decays more rapidly with distance and stabilizes at lower levels in these random subsamples of SMO populations, indicating that the two Iberian lynx populations differ in LD decay in accordance to differences in current effective population sizes.

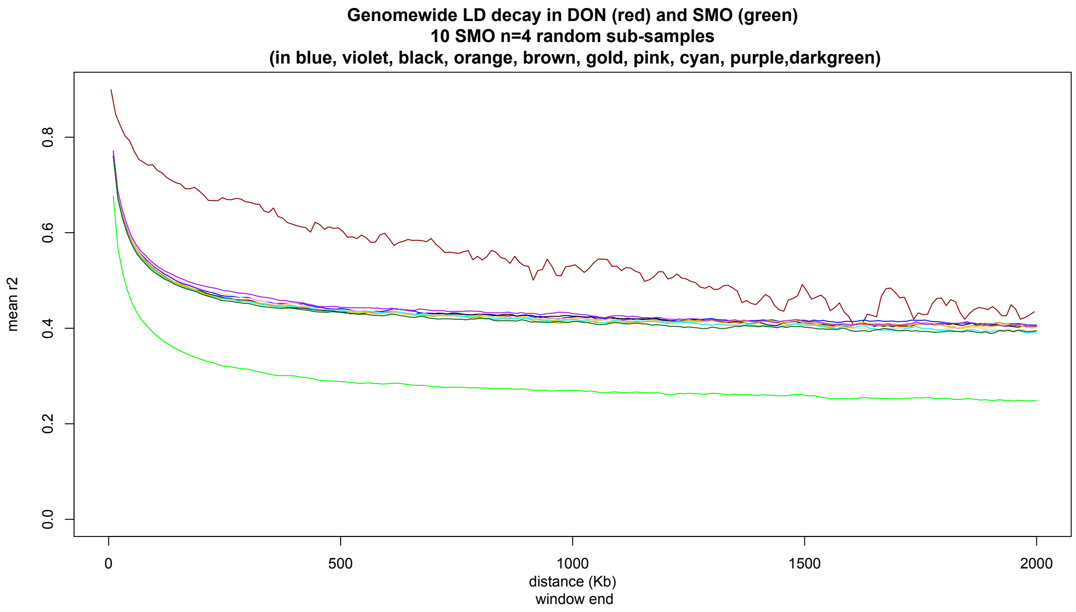

Figure S46. LD Decay of 10 random subsamples from Sierra Morena. The plot compares the LD decay estimated for Doñana (red) and Sierra Morena (green) with 10 random subsamples of size 4 drawn from Sierra Morena.

25 The Iberian lynx genome browser

To provide a common public entry point to all data generated by the project, we have made it accessible through an instance of JBrowse [242]. JBrowse is a genome browser that renders tracks by leveraging the presence of JavaScript and HTML5 in modern Internet browsers. While traditional genome browsers usually consist of heavy weight servers sending to client browsers precomputed images to be displayed, JBrowse's architecture allows for a lightweight server transmitting chunks of data to be processed by the client. In addition, client data visualization can be easily customized by specifying new CSS styles on the server. Interesting locations and track configurations can be straightforwardly shared with other users by sending them the URL the client is currently visualizing.

There is a number of tracks available for display, that can be used to obtain a fairly complete insight of the genome of *L. pardinus*:

1. Basic tracks: assembled reference sequence for *L. pardinus*, GC content, mappability [243], repeats (repeatmasker.org).
2. Assembly tracks. They serve the purpose of tracking the different stages of the assembly, and consist of alignments to the reference genome of contigs from whole-genome shotgun sequencing, scaffolds from fosmid-pool sequencing, merged scaffolds from fosmid-pool sequencing, super-scaffolds from large insert-size libraries, and super-scaffolds after rescaffolding with RNA-sequencing data.
3. Annotation tracks. Apart from the main reference annotation track, and a track showing predicted long non-coding RNAs (Section 3) several evidence-based tracks are available (alignment to the lynx genome of cDNAs, Cufflinks models, proteins, and cat ESTs.)
4. Synteny tracks. They display the synteny of the lynx genome with those of other felids (at the moment *F. catus*, and *P. tigris*) as represented by colinear chains of BLAT alignments (Supplemetary Section 9).
5. Variation track. It shows the variants (SNPs) found when comparing the

L. pardinus reference sequence to the re-sequencing data available for other lynx individuals (Supplemetary Section 16)

6. RNA-sequencing tracks. For each lynx tissue whose RNA has been sequenced (blood, brain, heart, kidney, liver, lung, muscle, pancreas, spleen, stomach and testes) two tracks are available. The first one displays the alignment coverage of RNA-sequencing spliced and unspliced reads, the second one the intron coverage provided by spliced reads. The tracks were generated with a RNA-sequencing pipeline based on GEM [7].

The Lynx genome browser portal can be publicly accessed at the URL http://denovo.cnag.cat/genomes/iberian_lynx

26 References

1. Palomares F, Rodríguez A, Revilla E, López-Bao JV, Calzada J: **Assessment of the conservation efforts to prevent extinction of the *Iberian lynx*.** *Conserv Biol* 2011, **25:**4-8.
2. Brown RM, Otero LJ, Brown GK: **Transfection screening for primary defects in the pyruvate dehydrogenase E1 alpha subunit gene.** *Hum Mol Genet* 1997, **6:**1361-1367.
3. Zhang HB, Scheuring CF, Zhang MP, Zhang Y, Wu CC, Dong JJ, Li YN: **Construction of BIBAC and BAC libraries from a variety of organisms for advanced genomics research.** *Nat Protoc* 2012, **7:**479-499.
4. Vinogradov AE: **Genome size and GC-percent in vertebrates as determined by flow cytometry: The triangular relationship.** *Cytometry* 1998, **31:**100-109.
5. Simpson JT, Wong K, Jackman SD, Schein JE, Jones SJ, Birol I: **ABySS: a parallel assembler for short read sequence data.** *Genome Res* 2009, **19:**1117-1123.
6. Boetzer M, Pirovano W: **Toward almost closed genomes with GapFiller.** *Genome Biol* 2012, **13:**R56.
7. Marco-Sola S, Sammeth M, Guigo R, Ribeca P: **The GEM mapper: fast, accurate and versatile alignment by filtration.** *Nat Methods* 2012, **9:**1185-1188.
8. Kent WJ: **BLAT - The BLAST-like alignment tool.** *Genome Res* 2002, **12:**656-664.

9. Parra G, Bradnam K, Korf I: **CEGMA: a pipeline to accurately annotate core genes in eukaryotic genomes.** *Bioinformatics* 2007, **23:**1061-1067.
10. Raineri E, Ferretti L, Esteve-Codina A, Nevado B, Heath S, Perez-Enciso M: **SNP calling by sequencing pooled samples.** *BMC Bioinformatics* 2012, **13:**e239.
11. Parra G, Bradnam K, Ning Z, Keane T, Korf I: **Assessing the gene space in draft genomes.** *Nucleic Acids Res* 2009, **37:**289-297.
12. Dobrynin P, Liu S, Tamazian G, Xiong Z, Yurchenko A, Krasheninnikova K, Kliver S, Schmidt-Kuntzel A, Koepfli K-P, Johnson W, et al: **Genomic legacy of the African cheetah, Acinonyx jubatus.** *Genome Biol* 2015, **16:**277.
13. Sindicic M, Gomercic T, Galov A, Polanc P, Huber D, Slavica A: **Repetitive sequences in Eurasian lynx (*Lynx lynx* L.) mitochondrial DNA control region.** *Mitochondrial DNA* 2012, **23:**201-207.
14. Li G, Davis BW, Raudsepp T, Wilkerson AJP, Mason VC, Ferguson-Smith M, O'Brien PC, Waters PD, Murphy WJ: **Comparative analysis of mammalian Y chromosomes illuminates ancestral structure and lineage-specific evolution.** *Genome Res* 2013, **23:**1486-1495.
15. Murphy WJ, Wilkerson AJP, Raudsepp T, Agarwala R, Schaffer AA, Stanyon R, Chowdhary BP: **Novel gene acquisition on carnivore Y chromosomes.** *PLoS Genet* 2006, **2:**353-363.
16. Wilkerson AJP, Raudsepp T, Graves T, Albracht D, Warren W, Chowdhary BP, Skow LC, Murphy WJ: **Gene discovery and comparative analysis of X-degenerate genes from the domestic cat Y chromosome.** *Genomics* 2008, **92:**329-338.
17. **RepeatMasker Open-4.0** [http://www.repeatmasker.org]
18. Mouse Genome Sequencing C, Waterston RH, Lindblad-Toh K, Birney E, Rogers J, Abril JF, Agarwal P, Agarwala R, Ainscough R, Alexandersson M, et al: **Initial sequencing and comparative analysis of the mouse genome.** *Nature* 2002, **420:**520-562.
19. Gibbs RA, Weinstock GM, Metzker ML, Muzny DM, Sodergren EJ, Scherer S, Scott G, Steffen D, Worley KC, Burch PE, et al: **Genome sequence of the Brown Norway rat yields insights into mammalian evolution.** *Nature* 2004, **428:**493-521.
20. Parra G, Agarwal P, Abril JF, Wiehe T, Fickett JW, Guigó R: **Comparative gene prediction in human and mouse.** *Genome Res* 2003, **13:**108-117.
21. Majoros WH, Pertea M, Salzberg SL: **TigrScan and GlimmerHMM: two open source ab initio eukaryotic gene-finders.** *Bioinformatics* 2004, **20:**2878-2879.
22. Stanke M, Waack S: **Gene prediction with a hidden Markov model and a new intron submodel.** *Bioinformatics* 2003, **19 Suppl 2:**ii215-225.
23. Wu TD, Watanabe CK: **GMAP: a genomic mapping and alignment program for mRNA and EST sequences.** *Bioinformatics* 2005, **21:**1859-1875.
24. Haas BJ, Salzberg SL, Zhu W, Pertea M, Allen JE, Orvis J, White O, Buell CR, Wortman JR: **Automated eukaryotic gene structure annotation using EVidenceModeler and the Program to Assemble Spliced Alignments.** *Genome Biol* 2008, **9:**R7.

25. Trapnell C, Williams BA, Pertea G, Mortazavi A, Kwan G, van Baren MJ, Salzberg SL, Wold BJ, Pachter L: **Transcript assembly and quantification by RNA-Seq reveals unannotated transcripts and isoform switching during cell differentiation.** *Nat Biotechnol* 2010, **28:**511-515.
26. Iwata H, Gotoh O: **Benchmarking spliced alignment programs including Spaln2, an extended version of Spaln that incorporates additional species-specific features.** *Nucleic Acids Res* 2012, **40:**e161.
27. Nawrocki EP, Kolbe DL, Eddy SR: **Infernal 1.0: inference of RNA alignments.** *Bioinformatics* 2009, **25:**1335-1337.
28. Griffiths-Jones S, Moxon S, Marshall M, Khanna A, Eddy SR, Bateman A: **Rfam: annotating non-coding RNAs in complete genomes.** *Nucleic Acids Res* 2005, **33:**D121-D124.
29. Esteve-Codina A, Kofler R, Palmieri N, Bussotti G, Notredame C, Pérez-Enciso M: **Exploring the gonad transcriptome of two extreme male pigs with RNA-seq.** *BMC Genomics* 2011, **12**.
30. Derrien T, Johnson R, Bussotti G, Tanzer A, Djebali S, Tilgner H, Guernec G, Martin D, Merkel A, Knowles DG, et al: **The GENCODE v7 catalog of human long noncoding RNAs: Analysis of their gene structure, evolution, and expression.** *Genome Res* 2012, **22:**1775-1789.
31. Notredame C, Higgins DG, Heringa J: **T-Coffee: A novel method for fast and accurate multiple sequence alignment.** *J Mol Biol* 2000, **302:**205-217.
32. Trapnell C, Williams BA, Pertea G, Mortazavi A, Kwan G, van Baren MJ, Salzberg SL, Wold BJ, Pachter L: **Transcript assembly and quantification by RNA-Seq reveals unannotated transcripts and isoform switching during cell differentiation.** *Nat Biotechnol* 2010, **28:**511-U174.
33. Parra G, Blanco E, Guigo R: **GeneID in *Drosophila*.** *Genome Res* 2000, **10:**511-515.
34. Morgulis A, Gertz EM, Schäffer AA, Agarwala R: **A fast and symmetric DUST implementation to mask low-complexity DNA sequences.** *J Comput Biol* 2006, **13:**1028-1040.
35. Zdobnov EM, Apweiler R: **InterProScan - an integration platform for the signature-recognition methods in InterPro.** *Bioinformatics* 2001, **17:**847-848.
36. Hunter S, Jones P, Mitchell A, Apweiler R, Attwood TK, Bateman A, Bernard T, Binns D, Bork P, Burge S, et al: **InterPro in 2011: new developments in the family and domain prediction database.** *Nucleic Acids Res* 2012, **40:**D306-D312.
37. Kanehisa M, Goto S, Sato Y, Furumichi M, Tanabe M: **KEGG for integration and interpretation of large-scale molecular data sets.** *Nucleic Acids Res* 2012, **40:**D109-D114.
38. Matthews L, Gopinath G, Gillespie M, Caudy M, Croft D, de Bono B, Garapati P, Hemish J, Hermjakob H, Jassal B, et al: **Reactome knowledgebase of human biological pathways and processes.** *Nucleic Acids Res* 2009, **37:**D619-D622.
39. Gotz S, Garcia-Gómez JM, Terol J, Williams TD, Nagaraj SH, Nueda MJ, Robles M, Talon M, Dopazo J, Conesa A: **High-throughput functional**

annotation and data mining with the Blast2GO suite. *Nucleic Acids Res* 2008, **36:**3420-3435.
40. Petersen TN, Brunak S, von Heijne G, Nielsen H: **SignalP 4.0: discriminating signal peptides from transmembrane regions.** *Nat Methods* 2011, **8:**785-786.
41. Moriya Y, Itoh M, Okuda S, Yoshizawa AC, Kanehisa M: **KAAS: an automatic genome annotation and pathway reconstruction server.** *Nucleic Acids Res* 2007, **35:**W182-W185.
42. Huerta-Cepas J, Capella-Gutiérrez S, Pryszcz LP, Denisov I, Kormes D, Marcet-Houben M, Gabaldon T: **PhylomeDB v3.0: an expanding repository of genome-wide collections of trees, alignments and phylogeny-based orthology and paralogy predictions.** *Nucleic Acids Res* 2011, **39:**D556-D560.
43. Harrow J, Frankish A, González JM, Tapanari E, Diekhans M, Kokocinski F, Aken BL, Barrell D, Zadissa A, Searle S, et al: **GENCODE: The reference human genome annotation for The ENCODE Project.** *Genome Res* 2012, **22:**1760-1774.
44. Flicek P, Amode MR, Barrell D, Beal K, Brent S, Carvalho-Silva D, Clapham P, Coates G, Fairley S, Fitzgerald S, et al: **Ensembl 2012.** *Nucleic Acids Res* 2012, **40:**D84-D90.
45. Rodríguez JM, Maietta P, Ezkurdia I, Pietrelli A, Wesselink JJ, López G, Valencia A, Tress ML: **APPRIS: annotation of principal and alternative splice isoforms.** *Nucleic Acids Res* 2013, **41:**D110-D117.
46. Altschul SF, Madden TL, Schaffer AA, Zhang JH, Zhang Z, Miller W, Lipman DJ: **Gapped BLAST and PSI-BLAST: a new generation of protein database search programs.** *Nucleic Acids Res* 1997, **25:**3389-3402.
47. Emanuelsson O, Brunak S, von Heijne G, Nielsen H: **Locating proteins in the cell using TargetP, SignalP and related tools.** *Nat Protoc* 2007, **2:**953-971.
48. López G, Maietta P, Rodríguez JM, Valencia A, Tress ML: **firestar-advances in the prediction of functionally important residues.** *Nucleic Acids Res* 2011, **39:**W235-W241.
49. López G, Valencia A, Tress ML: **firestar - prediction of functionally important residues using structural templates and alignment reliability.** *Nucleic Acids Res* 2007, **35:**W573-W577.
50. López G, Valencia A, Tress M: **FireDB - a database of functionally important residues from proteins of known structure.** *Nucleic Acids Res* 2007, **35:**D219-D223.
51. Rose PW, Beran B, Bi CX, Bluhm WF, Dimitropoulos D, Goodsell DS, Prlic A, Quesada M, Quinn GB, Westbrook JD, et al: **The RCSB Protein Data Bank: redesigned web site and web services.** *Nucleic Acids Res* 2011, **39:**D392-D401.
52. Hildebrand A, Remmert M, Biegert A, Soding J: **Fast and accurate automatic structure prediction with HHpred.** *Proteins* 2009, **77:**128-132.
53. Punta M, Coggill PC, Eberhardt RY, Mistry J, Tate J, Boursnell C, Pang N, Forslund K, Ceric G, Clements J, et al: **The Pfam protein families database.** *Nucleic Acids Res* 2012, **40:**D290-D301.

54. Jones DT: **Improving the accuracy of transmembrane protein topology prediction using evolutionary information.** *Bioinformatics* 2007, **23:**538-544.
55. Kall L, Krogh A, Sonnhammer ELL: **A combined transmembrane topology and signal peptide prediction method.** *J Mol Biol* 2004, **338:**1027-1036.
56. Viklund H, Elofsson A: **Best alpha-helical transmembrane protein topology predictions are achieved using hidden Markov models and evolutionary information.** *Protein Sci* 2004, **13:**1908-1917.
57. Pruitt KD, Harrow J, Harte RA, Wallin C, Diekhans M, Maglott DR, Searle S, Farrell CM, Loveland JE, Ruef BJ, et al: **The consensus coding sequence (CCDS) project: Identifying a common protein-coding gene set for the human and mouse genomes.** *Genome Res* 2009, **19:**1316-1323.
58. Quesada V, Ordoñez GR, Sánchez LM, Puente XS, López-Otín C: **The Degradome database: mammalian proteases and diseases of proteolysis.** *Nucleic Acids Res* 2009, **37:**D239-D243.
59. Puente XS, Sánchez LM, Overall CM, López-Otín C: **Human and mouse proteases: A comparative genomic approach.** *Nat Rev Genet* 2003, **4:**544-558.
60. Ordoñez GR, Puente XS, Quesada V, López-Otín C: **Proteolytic systems: constructing degradomes.** *Met Mol Biol* 2009, **539:**33-47.
61. Puente XS, López-Otín C: **A genomic analysis of rat proteases and protease inhibitors.** *Genome Res* 2004, **14:**609-622.
62. Ordoñez GR, Hillier LW, Warren WC, Grutzner F, López-Otín C, Puente XS: **Loss of genes implicated in gastric function during platypus evolution.** *Genome Biol* 2008, **9:**R81.
63. Puente XS, Sánchez LM, Overall CM, López-Otín C: **Human and mouse proteases: a comparative genomic approach.** *Nat Rev Genet* 2003, **4:**544-558.
64. Quesada V, Velasco G, Puente XS, Warren WC, López-Otín C: **Comparative genomic analysis of the zebra finch degradome provides new insights into evolution of proteases in birds and mammals.** *BMC Genomics* 2010, **11:** 220.
65. Puente XS, Gutiérrez-Fernández A, Ordoñez GR, Hillier LW, López-Otín C: **Comparative genomic analysis of human and chimpanzee proteases.** *Genomics* 2005, **86:**638-647.
66. Locke DP, Hillier LW, Warren WC, Worley KC, Nazareth LV, Muzny DM, Yang SP, Wang Z, Chinwalla AT, Minx P, et al: **Comparative and demographic analysis of orang-utan genomes.** *Nature* 2011, **469:**529-533.
67. López-Otín C, Bond JS: **Proteases: multifunctional enzymes in life and disease.** *J Biol Chem* 2008, **283:**30433-30437.
68. Mikkelsen TS, Hillier LW, Eichler EE, Zody MC, Jaffe DB, Yang SP, Enard W, Hellmann I, Lindblad-Toh K, Altheide TK, et al: **Initial sequence of the chimpanzee genome and comparison with the human genome.** *Nature* 2005, **437:**69-87.
69. Warren WC, Clayton DF, Ellegren H, Arnold AP, Hillier LW, Kunstner A, Searle S, White S, Vilella AJ, Fairley S, et al: **The genome of a songbird.** *Nature* 2010, **464:**757-762.

70. Warren WC, Hillier LW, Marshall Graves JA, Birney E, Ponting CP, Grutzner F, Belov K, Miller W, Clarke L, Chinwalla AT, et al: **Genome analysis of the platypus reveals unique signatures of evolution.** *Nature* 2008, **453:**175-183.
71. Bryce SD, Lindsay S, Gladstone AJ, Braithwaite K, Chapman C, Spurr NK, Lunec J: **A novel family of cathepsin L-like (CTSLL) sequences on human chromosome 10q and related transcripts.** *Genomics* 1994, **24:**568-576.
72. Lombardi G, Burzyn D, Mundinano J, Berguer P, Bekinschtein P, Costa H, Castillo LF, Goldman A, Meiss R, Piazzon I, Nepomnaschy I: **Cathepsin-L influences the expression of extracellular matrix in lymphoid organs and plays a role in the regulation of thymic output and of peripheral T cell number.** *J Immunol* 2005, **174:**7022-7032.
73. Schurigt U, Eilenstein R, Gajda M, Leipner C, Sevenich L, Reinheckel T, Peters C, Wiederanders B, Brauer R: **Decreased arthritis severity in cathepsin L-deficient mice is attributed to an impaired T helper cell compartment.** *Inflammation Res* 2012, **61:**1021-1029.
74. Simmons G, Gosalia DN, Rennekamp AJ, Reeves JD, Diamond SL, Bates P: **Inhibitors of cathepsin L prevent severe acute respiratory syndrome coronavirus entry.** *Proc Natl Acad Sci USA* 2005, **102:**11876-11881.
75. Hood CL, Abraham J, Boyington JC, Leung K, Kwong PD, Nabel GJ: **Biochemical and structural characterization of cathepsin L-processed Ebola virus glycoprotein: implications for viral entry and immunogenicity.** *J Virol* 2010, **84:**2972-2982.
76. Bosch BJ, Bartelink W, Rottier PJ: **Cathepsin L functionally cleaves the severe acute respiratory syndrome coronavirus class I fusion protein upstream of rather than adjacent to the fusion peptide.** *J Virol* 2008, **82:**8887-8890.
77. Gallwitz M, Hellman L: **Rapid lineage-specific diversification of the mast cell chymase locus during mammalian evolution.** *Immunogenetics* 2006, **58:**641-654.
78. Griebel T, Zacher B, Ribeca P, Raineri E, Lacroix V, Guigo R, Sammeth M: **Modelling and simulating generic RNA-Seq experiments with the flux simulator.** *Nucleic Acids Res* 2012, **40:**10073-10083.
79. Ramskold D, Wang ET, Burge CB, Sandberg R: **An abundance of ubiquitously expressed genes revealed by tissue transcriptome sequence data.** *PLoS Comp Biol* 2009, **5:**e1000598.
80. Brawand D, Soumillon M, Necsulea A, Julien P, Csardi G, Harrigan P, Weier M, Liechti A, Aximu-Petri A, Kircher M, et al: **The evolution of gene expression levels in mammalian organs.** *Nature* 2011, **478:**343-348.
81. Robinson MD, McCarthy DJ, Smyth GK: **edgeR: a Bioconductor package for differential expression analysis of digital gene expression data.** *Bioinformatics* 2010, **26:**139-140.
82. Mortazavi A, Williams BA, Mccue K, Schaeffer L, Wold B: **Mapping and quantifying mammalian transcriptomes by RNA-Seq.** *Nat Methods* 2008, **5:**621-628.
83. Huerta-Cepas J, Capella-Gutiérrez S, Pryszcz LP, Marcet-Houben M, Gabaldon T: **PhylomeDB v4: zooming into the plurality of**

evolutionary histories of a genome. *Nucleic Acids Res* 2014, **42:**D897-D902.

84. Bolstad BM, Irizarry RA, Astrand M, Speed TP: **A comparison of normalization methods for high density oligonucleotide array data based on variance and bias.** *Bioinformatics* 2003, **19:**185-193.
85. Tarazona S, Garcia-Alcalde F, Dopazo J, Ferrer A, Conesa A: **Differential expression in RNA-seq: A matter of depth.** *Genome Res* 2011, **21:**2213-2223.
86. Dobin A, Davis CA, Schlesinger F, Drenkow J, Zaleski C, Jha S, Batut P, Chaisson M, Gingeras TR: **STAR: ultrafast universal RNA-seq aligner.** *Bioinformatics* 2013, **29:**15-21.
87. Müller K, Koster S, Painer J, Söderberg A, Gavier-Widèn D, Brunner E, Dehnhard M, Jewgenow K: **Testosterone production and spermatogenesis in free-ranging Eurasian lynx (*Lynx lynx*) throughout the year.** *Eur J Wildl Res* 2014**:**1-9.
88. Martínez F, Manteca X, Pastor J: **Retrospective study of morbidity and mortality of captive Iberian lynx (*Lynx pardinus*) in the *ex situ* conservation programme (2004-june 2010).** *J Zoo Wildl Med* 2013, **44:**845-852.
89. Nenadic O, Greenacre M: **Correspondence analysis in R, with two- and three-dimensional graphics: The ca package.** *J Stat Softw* 2007, **20**.
90. Opgen-Rhein R, Strimmer K: **From correlation to causation networks: a simple approximate learning algorithm and its application to high-dimensional plant gene expression data.** *Bmc Systems Biology* 2007, **1:**e37.
91. Schafer J, Strimmer K: **A shrinkage approach to large-scale covariance matrix estimation and implications for functional genomics.** *Stat Appl Genet Molec Biol* 2005, **4:**Artn 32.
92. Wissler L, Gadau J, Simola DF, Helmkampf M, Bornberg-Bauer E: **Mechanisms and dynamics of orphan gene emergence in insect genomes.** *Genome Biol Evol* 2013, **5:**439-455.
93. Dujon B: **The yeast genome project: What did we learn?** *Trends Genet* 1996, **12:**263-270.
94. Khalturin K, Anton-Erxleben F, Sassmann S, Wittlieb J, Hemmrich G, Bosch TCG: **A novel gene family controls species-specific morphological traits in *Hydra*.** *PLoS Biol* 2008, **6:**2436-2449.
95. Toll-Riera M, Bosch N, Bellora N, Castelo R, Armengol L, Estivill X, Albà MM: **Origin of primate orphan genes: A comparative genomics approach.** *Mol Biol Evol* 2009, **26:**603-612.
96. Altschul SF, Gish W, Miller W, Myers EW, Lipman DJ: **Basic Local Alignment Search Tool.** *J Mol Biol* 1990, **215:**403-410.
97. Kaessmann H: **Origins, evolution, and phenotypic impact of new genes.** *Genome Res* 2010, **20:**1313-1326.
98. Toll-Riera M, Rado-Trilla N, Martys F, Albà MM: **Role of low-complexity sequences in the formation of novel protein coding sequences.** *Mol Biol Evol* 2012, **29:**883-886.
99. Tautz D, Domazet-Loso T: **The evolutionary origin of orphan genes.** *Nat Rev Genet* 2011, **12:**692-702.

100. Wootton JC: **Nonglobular domains in protein sequences - Automated segmentation using complexity-measures.** *Comput Chem* 1994, **18:**269-285.
101. Kielbasa SM, Wan R, Sato K, Horton P, Frith MC: **Adaptive seeds tame genomic sequence comparison.** *Genome Res* 2011, **21:**487-493.
102. Mailund T, Dutheil JY, Hobolth A, Lunter G, Schierup MH: **Estimating divergence time and ancestral effective population size of Bornean and Sumatran orangutan subspecies using a coalescent hidden Markov model.** *PLoS Genet* 2011, **7:**e1001319.
103. Mailund T, Halager AE, Westergaard M, Dutheil JY, Munch K, Andersen LN, Lunter G, Prufer K, Scally A, Hobolth A, Schierup MH: **A new isolation with migration model along complete genomes infers very different divergence processes among closely related great ape species.** *PLoS Genet* 2012, **8:**e1003125.
104. Johnson WE, Eizirik E, Pecon-Slattery J, Murphy WJ, Antunes A, Teeling E, O'Brien SJ: **The Late Miocene radiation of modern Felidae: A genetic assessment.** *Science* 2006, **311:**73-77.
105. Cho YS, Hu L, Hou H, Lee H, Xu J, Kwon S, Oh S, Kim H-M, Jho S, Kim S, et al: **The tiger genome and comparative analysis with lion and snow leopard genomes.** *Nat Commun* 2013, **4:**2433.
106. Li H, Durbin R: **Inference of human population history from individual whole-genome sequences.** *Nature* 2011, **475:**493-U484.
107. Gutenkunst RN, Hernandez RD, Williamson SH, Bustamante CD: **Inferring the joint demographic history of multiple populations from multidimensional SNP frequency data.** *PLoS Genet* 2009, **5:**e1000695.
108. Li H, Durbin R: **Fast and accurate short read alignment with Burrows–Wheeler transform.** *Bioinformatics* 2009, **25:**1754-1760.
109. DePristo MA, Banks E, Poplin R, Garimella KV, Maguire JR, Hartl C, Philippakis AA, del Angel G, Rivas MA, Hanna M, et al: **A framework for variation discovery and genotyping using next-generation DNA sequencing data.** *Nat Genet* 2011, **43:**491-+.
110. Prado-Martínez J, Sudmant PH, Kidd JM, Li H, Kelley JL, Lorente-Galdos B, Veeramah KR, Woerner AE, O'Connor TD, Santpere G, et al: **Great ape genetic diversity and population history.** *Nature* 2013, **499:**471-475.
111. Groenen MAM, Archibald AL, Uenishi H, Tuggle CK, Takeuchi Y, Rothschild MF, Rogel-Gaillard C, Park C, Milan D, Megens HJ, et al: **Analyses of pig genomes provide insight into porcine demography and evolution.** *Nature* 2012, **491:**393-398.
112. Orlando L, Ginolhac A, Zhang G, Froese D, Albrechtsen A, Stiller M, Schubert M, Cappellini E, Petersen B, Moltke I, et al: **Recalibrating Equus evolution using the genome sequence of an early Middle Pleistocene horse.** *Nature* 2013, **499:**74-78.
113. Hwang DG, Green P: **Bayesian Markov chain Monte Carlo sequence analysis reveals varying neutral substitution patterns in mammalian evolution.** *Proc Natl Acad Sci USA* 2004, **101:**13994-14001.
114. Casas-Marce M, Soriano L, López-Bao JV, Godoy JA: **Genetics at the verge of extinction: insights from the Iberian lynx.** *Mol Ecol* 2013, **22:**5503-5515.

115. Seabright M: **Use of proteolytic-enzymes for mapping of structural rearrangements in chromosomes of man.** *Chromosoma* 1972, **36:**204-&.
116. Wursterhill DH, Centerwall WR: **The interrelationships of chromosome-banding patterns in canids, mustelids, hyena, and felids.** *Cytogenet Cell Genet* 1982, **34:**178-192.
117. Nie W, Wang J, Su W, Wang D, Tanomtong A, Perelman PL, Graphodatsky AS, Yang F: **Chromosomal rearrangements and karyotype evolution in carnivores revealed by chromosome painting.** *Heredity* 2012, **108:**17-27.
118. O'Brien JT, Menninger JC, Nash WG: *Atlas of Mammalian Chromosomes.* Hoboken, NJ: John Wiley & Sons, Inc; 2006.
119. Wursterhill DH, Gray CW: **Interrelationships of chromosome-banding patterns in procyonids, viverrids, and felids.** *Cytogenet Cell Genet* 1975, **15:**306-331.
120. Wursterhill DH, Gray CW: **Giemsa-banding patterns in chromosomes of 12 species of cats (*Felidae*).** *Cytogenet Cell Genet* 1973, **12:**377-397.
121. Kiełbasa SM, Wan R, Sato K, Horton P, Frith MC: **Adaptive seeds tame genomic sequence comparison.** *Genome Res* 2011, **21:**487-493.
122. Quinlan AR, Hall IM: **BEDTools: a flexible suite of utilities for comparing genomic features.** *Bioinformatics* 2010, **26:**841-842.
123. Huerta-Cepas J, Capella-Gutiérrez S, Pryszcz LP, Denisov I, Kormes D, Marcet-Houben M, Gabaldón T: **PhylomeDB v3.0: an expanding repository of genome-wide collections of trees, alignments and phylogeny-based orthology and paralogy predictions.** *Nucleic Acids Res* 2011, **39:**D556-560.
124. Edgar RC: **MUSCLE: a multiple sequence alignment method with reduced time and space complexity.** *BMC Bioinformatics* 2004, **5:**113-113.
125. Katoh K, Kuma K-i, Toh H, Miyata T: **MAFFT version 5: improvement in accuracy of multiple sequence alignment.** *Nucleic Acids Res* 2005, **33:**511-518.
126. Lassmann T, Frings O, Sonnhammer ELL: **Kalign2: high-performance multiple alignment of protein and nucleotide sequences allowing external features.** *Nucleic Acids Res* 2009, **37:**858-865.
127. Landan G, Graur D: **Heads or tails: a simple reliability check for multiple sequence alignments.** *Mol Biol Evol* 2007, **24:**1380-1383.
128. Wallace IM, O'Sullivan O, Higgins DG, Notredame C: **M-Coffee: combining multiple sequence alignment methods with T-Coffee.** *Nucleic Acids Res* 2006, **34:**1692-1699.
129. Capella-Gutiérrez S, Silla-Martínez JM, Gabaldón T: **trimAl: a tool for automated alignment trimming in large-scale phylogenetic analyses.** *Bioinformatics* 2009, **25:**1972-1973.
130. Gil M, Zanetti MS, Zoller S, Anisimova M: **CodonPhyML: fast maximum likelihood phylogeny estimation under codon substitution models.** *Mol Biol Evol* 2013, **30:**1270-1280.
131. Huerta-Cepas J, Dopazo J, Gabaldón T: **ETE: a python Environment for Tree Exploration.** *BMC Bioinformatics* 2010, **11:**24-24.

132. Huerta-Cepas J, Dopazo H, Dopazo J, Gabaldón T: **The human phylome.** *Genome Biol* 2007, **8:**R109-R109.
133. Gabaldon T: **Large-scale assignment of orthology: back to phylogenetics?** *Genome Biol* 2008, **9:**235.
134. Huerta-Cepas J, Capella-Gutiérrez S, Pryszcz LP, Marcet-Houben M, Gabaldón T: **PhylomeDB v4: zooming into the plurality of evolutionary histories of a genome.** *Nucleic Acids Res* 2014, **42:**D897-902.
135. Huerta-Cepas J, Gabaldón T: **Assigning duplication events to relative temporal scales in genome-wide studies.** *Bioinformatics* 2011, **27:**38-45.
136. Al-Shahrour F, Minguez P, Tárraga J, Medina I, Alloza E, Montaner D, Dopazo J: **FatiGO +: A functional profiling tool for genomic data. Integration of functional annotation, regulatory motifs and interaction data with microarray experiments.** *Nucleic Acids Res* 2007, **35**.
137. Slater GS, Birney E: **Automated generation of heuristics for biological sequence comparison.** *BMC Bioinformatics* 2005, **6:**e31.
138. Stamatakis A: **RAxML-VI-HPC: Maximum likelihood-based phylogenetic analyses with thousands of taxa and mixed models.** *Bioinformatics* 2006, **22:**2688-2690.
139. Felsenstein J: **Phylip: phylogeny inference package (version 3.2).** *Cladistics* 1989, **5:**164-166.
140. Wehe A, Bansal MS, Burleigh JG, Eulenstein O: **DupTree: a program for large-scale phylogenetic analyses using gene tree parsimony.** *Bioinformatics* 2008, **24:**1540-1541.
141. Kumar S, Hedges SB: **Timetree2: Species divergence times on the iPhone.** *Bioinformatics* 2011, **27:**2023-2024.
142. Sanderson MJ: **r8s: Inferring absolute rates of molecular evolution and divergence times in the absence of a molecular clock.** *Bioinformatics* 2003, **19:**301-302.
143. Yang Z: **PAML 4: Phylogenetic analysis by maximum likelihood.** *Mol Biol Evol* 2007, **24:**1586-1591.
144. Li G, Davis BW, Eizirik E, Murphy WJ: **Phylogenomic evidence for ancient hybridization in the genomes of living cats (Felidae).** *Genome Res* 2016, **26:**1-11.
145. Li G, Hillier LW, Grahn RA, Zimin AV, David VA, Menotti-Raymond M, Middleton R, Hannah S, Hendrickson S, Makunin A, et al: **A High-Resolution SNP Array-Based Linkage Map Anchors a New Domestic Cat Draft Genome Assembly and Provides Detailed Patterns of Recombination.** *G3: Genes|Genomes|Genetics* 2016, **6:**1607-1616.
146. Li H, Handsaker B, Wysoker A, Fennell T, Ruan J, Homer N, Marth G, Abecasis G, Durbin R, Proc GPD: **The Sequence Alignment/Map format and SAMtools.** *Bioinformatics* 2009, **25:**2078-2079.
147. Korneliussen TS, Albrechtsen A, Nielsen R: **ANGSD: Analysis of Next Generation Sequencing Data.** *BMC Bioinformatics* 2014, **15**.
148. Abyzov A, Urban AE, Snyder M, Gerstein M: **CNVnator: An approach to discover, genotype, and characterize typical and atypical CNVs from**

family and population genome sequencing. *Genome Res* 2011, **21:**974-984.
149. Zamani N, Russell P, Lantz H, Hoeppner MP, Meadows JRS, Vijay N, Mauceli E, di Palma F, Lindblad-Toh K, Jern P, Grabherr MG: **Unsupervised genome-wide recognition of local relationship patterns.** *BMC Genomics* 2013, **14**.
150. Stamatakis A: **RAxML version 8: a tool for phylogenetic analysis and post-analysis of large phylogenies.** *Bioinformatics* 2014, **30:**1312-1313.
151. Loytynoja A, Goldman N: **An algorithm for progressive multiple alignment of sequences with insertions.** *Proc Natl Acad Sci USA* 2005, **102:**10557-10562.
152. Jordan G, Goldman N: **The effects of alignment error and alignment filtering on the sitewise detection of positive selection.** *Mol Biol Evol* 2012, **29:**1125-1139.
153. Villanueva-Cañas JL, Laurie S, Albà MM: **Improving Genome-Wide Scans of Positive Selection by Using Protein Isoforms of Similar Length.** *Genome Biol Evol* 2013, **5:**457-467.
154. Fletcher W, Yang ZH: **The effect of insertions, deletions, and alignment errors on the branch-site test of positive selection.** *Mol Biol Evol* 2010, **27:**2257-2267.
155. Pérez-Llamas C, López-Bigas N: **Gitools: Analysis and visualisation of genomic data using interactive heat-maps.** *Plos One* 2011, **6:**e19541.
156. Flicek P, Ahmed I, Amode MR, Barrell D, Beal K, Brent S, Carvalho-Silva D, Clapham P, Coates G, Fairley S, et al: **Ensembl 2013.** *Nucleic Acids Res* 2013, **41:**D48-D55.
157. Benjamini Y, Hochberg Y: **Controlling the false discovery rate - a practical and powerful approach to multiple testing.** *J Roy Stat Soc Ser B* 1995, **57:**289-300.
158. Heffner HE, Heffner RS: **Audition.** In *Handbook of research methods in experimental psychology.* Edited by Davis SF. Malden, MA: Blackwell Pub.; 2003: 413-440: *Blackwell handbooks of research methods in psychology*].
159. Zadro C, Alemanno MS, Bellacchio E, Ficarella R, Donaudy F, Melchionda S, Zelante L, Rabionet R, Hilgert N, Estivill X, et al: **Are MYO1C and MYO1F associated with hearing loss?** *BBA-Mol Basis Dis* 2009, **1792:**27-32.
160. Satheesh SV, Kunert K, Ruttiger L, Zuccotti A, Schonig K, Friauf E, Knipper M, Bartsch D, Nothwang HG: **Retrocochlear function of the peripheral deafness gene Cacna1d.** *Hum Mol Genet* 2012, **21:**3896-3909.
161. Hunt DM, Buch P, Michaelides M: **Guanylate cyclases and associated activator proteins in retinal disease.** *Mol Cell Biochem* 2010, **334:**157-168.
162. Deininger PL, Moran JV, Batzer MA, Kazazian HH: **Mobile elements and mammalian genome evolution.** *Curr Opin Genet Dev* 2003, **13:**651-658.
163. Kidwell MG, Lisch DR: **Perspective: transposable elements, parasitic DNA, and genome evolution.** *Evolution* 2001, **55:**1-24.
164. Charlesworth D, Wright SI: **Breeding systems and genome evolution.** *Curr Opin Genet Dev* 2001, **11:**685-690.
165. Rizzon C, Marais G, Gouy M, Biémont C: **Recombination rate and the distribution of transposable elements in the *Drosophila melanogaster* genome.** *Genome Res* 2002, **12:**400-407.

166. Duret L, Marais G, Biémont C: **Transposons but not retrotransposons are located preferentially in regions of high recombination rate in Caenorhabditis elegans.** *Genetics* 2000, **156:**1661-1669.
167. Morgan MT: **Transposable element number in mixed mating populations.** *Genet Res* 2001, **77:**261-275.
168. Nordborg M: **Linkage disequilibrium, gene trees and selfing: an ancestral recombination graph with partial self-fertilization.** *Genetics* 2000, **154:**923-929.
169. Wright SI, Agrawal N, Bureau TE: **Effects of recombination rate and gene density on transposable element distributions in *Arabidopsis thaliana*.** *Genome Res* 2003, **13:**1897-1903.
170. Wright SI, Le QH, Schoen DJ, Bureau TE: **Population dynamics of an Ac-like transposable element in self-and cross-pollinating *Arabidopsis*.** *Genetics* 2001, **158:**1279-1288.
171. Charlesworth D, Charlesworth B: **Transposable elements in inbreeding and outbreeding populations.** *Genetics* 1995, **140:**415.
172. Wright SI, Schoen DJ: **Transposon dynamics and the breeding system.** In *Transposable Elements and Genome Evolution.* Edited by McDonald JF: Springer; 2000: 139-148
173. Gascuel O: **BIONJ: An improved version of the NJ algorithm based on a simple model of sequence data.** *Mol Biol Evol* 1997, **14:**685-695.
174. Jukes TH, Cantor CR: **Evolution of protein molecules.** In *Mammalian protein metabolism, III.* Edited by Munro HN. New York: Academic Press; 1969: 21-132
175. Zhang Y, Romanish MT, Mager DL: **Distributions of transposable elements reveal hazardous zones in mammalian introns.** *PLoS Comp Biol* 2011, **7:**e1002046.
176. Keane TM, Wong K, Adams DJ: **RetroSeq: transposable element discovery from next-generation sequencing data.** *Bioinformatics* 2013, **29:**389-390.
177. Rubio-Camarillo M, Gómez-López G, Fernández JM, Valencia A, Pisano DG: **RUbioSeq: a suite of parallelized pipelines to automate exome variation and bisulfite-seq analyses.** *Bioinformatics* 2013, **29:**1687-1689.
178. Peakall R, Smouse PE: **GenAlEx 6.5: genetic analysis in Excel. Population genetic software for teaching and research-an update.** *Bioinformatics* 2012, **28:**2537-2539.
179. Peakall R, Smouse PE: **GENALEX 6: genetic analysis in Excel. Population genetic software for teaching and research.** *Mol Ecol Notes* 2006, **6:**288-295.
180. Galtier N, Piganeau G, Mouchiroud D, Duret L: **GC-content evolution in mammalian genomes: the biased gene conversion hypothesis.** *Genetics* 2001, **159:**907-911.
181. Duret L, Galtier N: **Biased gene conversion and the evolution of mammalian genomic landscapes.** *Annu Rev Genomics Hum Genet* 2009, **10:**285-311.
182. Katzman S, Kern AD, Pollard KS, Salama SR, Haussler D: **GC-biased evolution near human accelerated regions.** *PLoS Genet* 2010, **6:**e1000960.

183. Kent CF, Minaei S, Harpur BA, Zayed A: **Recombination is associated with the evolution of genome structure and worker behavior in honey bees.** *Proc Natl Acad Sci USA* 2012, **109:**18012-18017.
184. Galtier N, Duret L, Glemin S, Ranwez V: **GC-biased gene conversion promotes the fixation of deleterious amino acid changes in primates.** *Trends Genet* 2009, **25:**1-5.
185. Rodríguez JM, Maietta P, Ezkurdia I, Pietrelli A, Wesselink J-J, López G, Valencia A, Tress ML: **APPRIS: annotation of principal and alternative splice isoforms.** *Nucleic Acids Res* 2013, **41:**D110-D117.
186. Cingolani P, Platts A, Wang LL, Coon M, Nguyen T, Wang L, Land SJ, Lu XY, Ruden DM: **A program for annotating and predicting the effects of single nucleotide polymorphisms, SnpEff: SNPs in the genome of *Drosophila melanogaster* strain w(1118); iso-2; iso-3.** *Fly* 2012, **6:**80-92.
187. Marais G, Charlesworth B, Wright SI: **Recombination and base composition: the case of the highly self-fertilizing plant *Arabidopsis thaliana*.** *Genome Biol* 2004, **5:**R45-R45.
188. Muyle A, Serres-Giardi L, Ressayre A, Escobar J, Glémin S: **GC-biased gene conversion and selection affect GC content in the *Oryza* genus (rice).** *Mol Biol Evol* 2011, **28:**2695-2706.
189. Glemin S: **Surprising fitness consequences of GC-biased gene conversion: I. Mutation load and inbreeding depression.** *Genetics* 2010, **185:**939-959.
190. Dreszer TR, Wall GD, Haussler D, Pollard KS: **Biased clustered substitutions in the human genome: The footprints of male-driven biased gene conversion.** *Genome Res* 2007, **17:**1420-1430.
191. Webster MT, Smith NG, Hultin-Rosenberg L, Arndt PF, Ellegren H: **Male-driven biased gene conversion governs the evolution of base composition in human alu repeats.** *Mol Biol Evol* 2005, **22:**1468-1474.
192. Jensen-Seaman MI, Furey TS, Payseur Ba, Lu Y, Roskin KM, Chen C-F, Thomas Ma, Haussler D, Jacob HJ: **Comparative recombination rates in the rat, mouse, and human genomes.** *Genome Res* 2004, **14:**528-538.
193. Yu A, Zhao C, Fan Y, Jang W, Mungall AJ, Deloukas P, Olsen A, Doggett NA, Ghebranious N, Broman KW: **Comparison of human genetic and sequence-based physical maps.** *Nature* 2001, **409:**951-953.
194. Kong A, Gudbjartsson DF, Sainz J, Jonsdottir GM, Gudjonsson SA, Richardsson B, Sigurdardottir S, Barnard J, Hallbeck B, Masson G: **A high-resolution recombination map of the human genome.** *Nat Genet* 2002, **31:**241-247.
195. Broman KW, Murray JC, Sheffield VC, White RL, Weber JL: **Comprehensive human genetic maps: individual and sex-specific variation in recombination.** *Am J Hum Genet* 1998, **63:**861-869.
196. Pollard KS, Salama SR, King B, Kern AD, Dreszer T, Katzman S, Siepel A, Pedersen JS, Bejerano G, Baertsch R: **Forces shaping the fastest evolving regions in the human genome.** *PLoS Genet* 2006, **2:**e168.
197. Charlesworth B: **Effective population size and patterns of molecular evolution and variation.** *Nat Rev Genet* 2009, **10:**195-205.

198. Tamazian G, Simonov S, Dobrynin P, Makunin A, Logachev A, Komissarov A, Shevchenko A, Brukhin V, Cherkasov N, Svitin A, et al: **Annotated features of domestic cat - Felis catus genome.** *GigaScience* 2014, **3:**13.
199. Benson G: **Tandem repeats finder: a program to analyze DNA sequences.** *Nucleic Acids Res* 1999, **27:**573-580.
200. Hach F, Sarrafi I, Hormozdiari F, Alkan C, Eichler EE, Sahinalp SC: **mrsFAST-Ultra: a compact, SNP-aware mapper for high performance sequencing applications.** *Nucleic Acids Res* 2014, **42:**W494-W500.
201. Alkan C, Kidd JM, Marques-Bonet T, Aksay G, Antonacci F, Hormozdiari F, Kitzman JO, Baker C, Malig M, Mutlu O, et al: **Personalized copy number and segmental duplication maps using next-generation sequencing.** *Nat Genet* 2009, **41:**1061-U1029.
202. Iqbal Z, Caccamo M, Turner I, Flicek P, McVean G: **De novo assembly and genotyping of variants using colored de Bruijn graphs.** *Nat Genet* 2012, **44:**226-232.
203. Compeau PEC, Pevzner PA, Tesler G: **How to apply de Bruijn graphs to genome assembly.** *Nat Biotechnol* 2011, **29:**987-991.
204. Auton A, Fledel-Alon A, Pfeifer S, Venn O, Segurel L, Street T, Leffler EM, Bowden R, Aneas I, Broxholme J, et al: **A fine-scale chimpanzee genetic map from population sequencing.** *Science* 2012, **336:**193-198.
205. Abbott R, Albach D, Ansell S, Arntzen JW, Baird SJE, Bierne N, Boughman JW, Brelsford A, Buerkle CA, Buggs R, et al: **Hybridization and speciation.** *J Evol Biol* 2013, **26:**229-246.
206. Lunter G, Goodson M: **Stampy: A statistical algorithm for sensitive and fast mapping of Illumina sequence reads.** *Genome Res* 2011, **21:**936-939.
207. Keller MC, Visscher PM, Goddard ME: **Quantification of inbreeding due to distant ancestors and its detection using dense single nucleotide polymorphism data.** *Genetics* 2011, **189:**237-U920.
208. Danecek P, Auton A, Abecasis G, Albers CA, Banks E, DePristo MA, Handsaker RE, Lunter G, Marth GT, Sherry ST, et al: **The variant call format and VCFtools.** *Bioinformatics* 2011, **27:**2156-2158.
209. Al-Shahrour F, Díaz-Uriarte R, Dopazo J: **FatiGO: a web tool for finding significant associations of Gene Ontology terms with groups of genes.** *Bioinformatics* 2004, **20:**578-580.
210. Jombart T, Ahmed I: **adegenet 1.3-1: new tools for the analysis of genome-wide SNP data.** *Bioinformatics* 2011, **27:**3070-3071.
211. Weir BS, Cockerham CC: **Estimating F-Statistics for the analysis of population structure.** *Evolution* 1984, **38:**1358-1370.
212. Willing EM, Dreyer C, van Oosterhout C: **Estimates of Genetic Differentiation Measured by F-ST Do Not Necessarily Require Large Sample Sizes When Using Many SNP Markers.** *Plos One* 2012, **7:**e42649.
213. Li S, Li B, Cheng C, Xiong Z, Liu Q, Lai J, Carey H, Zhang Q, Zheng H, Wei S, et al: **Genomic signatures of near-extinction and rebirth of the crested ibis and other endangered bird species.** *Genome Biol*.
214. Smith NG, Eyre-Walker A: **Adaptive protein evolution in *Drosophila*.** *Nature* 2002, **415:**1022-1024.

215. Stoletzki N, Eyre-Walker A: **Estimation of the Neutrality Index.** *Mol Biol Evol* 2011, **28:**63-70.
216. Begun DJ, Holloway AK, Stevens K, Hillier LW, Poh YP, Hahn MW, Nista PM, Jones CD, Kern AD, Dewey CN, et al: **Population genomics: Whole-genome analysis of polymorphism and divergence in *Drosophila* simulans.** *PLoS Biol* 2007, **5:**2534-2559.
217. Tsagkogeorga G, Cahais V, Galtier N: **The population genomics of a fast evolver: High levels of diversity, functional constraint, and molecular adaptation in the tunicate *Ciona intestinalis*.** *Genome Biol Evol* 2012, **4:**852-861.
218. Carneiro M, Albert FW, Melo-Ferreira J, Galtier N, Gayral P, Blanco-Aguiar JA, Villafuerte R, Nachman MW, Ferrand N: **Evidence for widespread positive and purifying selection across the European rabbit (*Oryctolagus cuniculus*) genome.** *Mol Biol Evol* 2012, **29:**1837-1849.
219. Bustamante CD, Fledel-Alon A, Williamson S, Nielsen R, Hubisz MT, Glanowski S, Tanenbaum DM, White TJ, Sninsky JJ, Hernandez RD, et al: **Natural selection on protein-coding genes in the human genome.** *Nature* 2005, **437:**1153-1157.
220. Loire E, Chiari Y, Bernard A, Cahais V, Romiguier J, Nabholz B, Lourenco JM, Galtier N: **Population genomics of the endangered giant Galapagos tortoise.** *Genome Biol* 2013, **14:**R136.
221. Pool JE, Nielsen R: **Population size changes reshape genomic patterns of diversity.** *Evolution* 2007, **61:**3001-3006.
222. Davinson AC, Hinkley DV: *Bootstrap methods and their application.* Cambridge: Univ. Press; 1997.
223. Canty A, Ripley B: **boot: Bootstrap R (S-Plus) Functions. R package version 1.3-11.**; 2014.
224. Arbiza L, Gottipati S, Siepel A, Keinan A: **Contrasting X-linked and autosomal diversity across 14 human populations.** *The American Journal of Human Genetics*, **94:**827-844.
225. Osada N, Nakagome S, Mano S, Kameoka Y, Takahashi I, Terao K: **Finding the factors of reduced genetic diversity on X chromosomes of *Macaca fascicularis*: Male-driven evolution, demography, and natural selection.** *Genetics* 2013, **195:**1027-+.
226. Medina I, Carbonell J, Pulido L, Madeira SC, Goetz S, Conesa A, Tarraga J, Pascual-Montano A, Nogales-Cadenas R, Santoyo J, et al: **Babelomics: an integrative platform for the analysis of transcriptomics, proteomics and genomic data with advanced functional profiling.** *Nucleic Acids Res* 2010, **38:**W210-W213.
227. Altshuler D, Durbin RM, Abecasis GR, Bentley DR, Chakravarti A, Clark AG, Collins FS, De la Vega FM, Donnelly P, Egholm M, et al: **A map of human genome variation from population-scale sequencing.** *Nature* 2010, **467:**1061-1073.
228. Scally A, Yngvadottir B, Xue YL, Ayub Q, Durbin R, Tyler-Smith C: **A genome-wide survey of genetic variation in gorillas using reduced representation sequencing.** *Plos One* 2013, **8:**e65066.
229. Hellmann I, Mang Y, Gu ZP, Li P, De La Vega FM, Clark AG, Nielsen R: **Population genetic analysis of shotgun assemblies of genomic sequences from multiple individuals.** *Genome Res* 2008, **18:**1020-1029.

230. Nachman MW: **Variation in recombination rate across the genome: evidence and implications.** *Curr Opin Genet Dev* 2002, **12:**657-663.
231. Ross-Ibarra J: **The evolution of recombination under domestication: A test of two hypotheses.** *Am Nat* 2004, **163:**105-112.
232. Otto SP, Barton NH: **Selection for recombination in small populations.** *Evolution* 2001, **55:**1921-1931.
233. Coop G, Przeworski M: **An evolutionary view of human recombination.** *Nat Rev Genet* 2007, **8:**23-34.
234. Montague MJ, Li G, Gandolfi B, Khan R, Aken BL, Searle SMJ, Minx P, Hillier LW, Koboldt DC, Davis BW, et al: **Comparative analysis of the domestic cat genome reveals genetic signatures underlying feline biology and domestication.** *Proc Natl Acad Sci USA* 2014, **111:**17230-17235.
235. Alonso S, López S, Izagirre N, de la Rua C: **Overdominance in the human genome and olfactory receptor activity.** *Mol Biol Evol* 2008, **25:**997-1001.
236. Esteve-Codina A, Kofler R, Himmelbauer H, Ferretti L, Vivancos AP, Groenen MAM, Folch JM, Rodríguez MC, Pérez-Enciso M: **Partial short-read sequencing of a highly inbred Iberian pig and genomics inference thereof.** *Heredity* 2011, **107:**256-264.
237. Niimura Y, Matsui A, Touhara K: **Expansion and contraction of olfactory receptor gene families during mammalian evolution: Comparative analysis among 13 Eutherians.** *Genes Genet Syst* 2013, **88:**383-383.
238. Riethman H: **Human telomere structure and biology.** *Annu Rev Genomics Hum Genet* 2008, **9:**1-19.
239. Niimura Y: **Olfactory receptor multigene family in vertebrates: From the viewpoint of evolutionary genomics.** *Curr Genomics* 2012, **13:**103-114.
240. Purcell S, Neale B, Todd-Brown K, Thomas L, Ferreira MAR, Bender D, Maller J, Sklar P, de Bakker PIW, Daly MJ, Sham PC: **PLINK: A tool set for whole-genome association and population-based linkage analyses.** *Am J Hum Genet* 2007, **81:**559-575.
241. Alhaddad H, Khan R, Grahn RA, Gandolfi B, Mullikin JC, Cole SA, Gruffydd-Jones TJ, Haggstrom J, Lohi H, Longeri M, Lyons LA: **Extent of linkage disequilibrium in the domestic cat, *Felis silvestris catus*, and its breeds.** *Plos One* 2013, **8:**e53537.
242. Skinner ME, Uzilov AV, Stein LD, Mungall CJ, Holmes IH: **JBrowse: a next-generation genome browser.** *Genome Res* 2009, **19:**1630-1638.
243. Derrien T, Estelle J, Marco Sola S, Knowles DG, Raineri E, Guigo R, Ribeca P: **Fast computation and applications of genome mappability.** *PloS one* 2012, **7:**e30377.
